# Dinutuximab Beta Added to Temozolomide-Based Chemotherapy for Children With Relapsed and Refractory Neuroblastoma: Results of the ITCC-SIOPEN BEACON Immuno Phase II Trial

**DOI:** 10.1200/JCO-25-01868

**Published:** 2025-12-12

**Authors:** Juliet C. Gray, Rebekah Weston, Cormac Owens, Adela Canete, Marion Gambart, Bram De Wilde, Karsten Nysom, Natasha van Eijkelenburg, Ruth Ladenstein, Aurora Castellano, Nicolas U. Gerber, Lynley V. Marshall, Giuseppe Barone, Alba Rubio-San-Simon, Antony Ng, Sucheta Vaidya, Soledad Gallego, Guy Makin, G. A. Amos Burke, Anthony McCarthy, Dermot Murphy, C. Michel Zwaan, Ricardo López-Almaraz, Sarah Jannier, Estelle Thebaud, Nadege Corradini, Dan Yeomanson, Lisa Howell, Deborah A. Tweddle, Martin Elliott, Dave Hobin, Dominique Valteau-Couanet, Gudrun Schleiermacher, Pascal Chastagner, Anne Sophie Defachelles, Benedicte Brichard, Sally George, Louis Chesler, Jennifer Laidler, Charlotte Firth, Grace Holt, Veronica Moroz, Andrew D.J. Pearson, Simon Gates, Keith Wheatley, Pam Kearns, Lucas Moreno

**Affiliations:** ^1^ Centre for Cancer Immunology, University of Southampton, Southampton, United Kingdom; ^2^ Cancer Research UK Clinical Trials Unit (CRCTU), University of Birmingham, Birmingham, United Kingdom; ^3^ Our Lady's Children's Hospital, Dublin, Republic of Ireland; ^4^ Unidad de Oncologia Pediatrica, Institute de Investigación Sanitaria La Fe, Valencia, Spain; ^5^ Hôpital des Enfants, Toulouse, France; ^6^ Universitair Hospital Gent, Gent, Belgium; ^7^ Copenhagen University Hospital, Rigshospitalet, Copenhagen, Denmark; ^8^ Princess Máxima Centre for Paediatric Oncology, Utrecht, The Netherlands; ^9^ Erasmus MC-Sophia Children's Hospital, Rotterdam, The Netherlands; ^10^ Department of Studies and Statistics for Integrated Research and Projects, Department of Paediatrics, St Anna Children's Hospital and Children's Cancer Research Institute, Medical University of Vienna, Vienna, Austria; ^11^ Bambino Gesù Children's Hospital, Rome, Italy; ^12^ Department of Oncology, University Children's Hospital, Zürich, Switzerland; ^13^ The Royal Marsden Hospital, Institute of Cancer Research, London, United Kingdom; ^14^ Great Ormond Street Hospital, London, United Kingdom; ^15^ Hospital del Niño Jesus, Madrid, Spain; ^16^ Bristol Royal Hospital for Children, Bristol, United Kingdom; ^17^ Hospital Vall d'Hebron, Barcelona, Spain; ^18^ Royal Manchester Children's Hospital, Manchester, United Kingdom; ^19^ Cancer Research UK Clinical Trials Unit, University of Birmingham, Birmingham, United Kingdom; ^20^ Royal Belfast Hospital for Sick Children, Belfast, United Kingdom; ^21^ Al Jalila Children's Speciality Hospital, Dubai, United Arab Emirates; ^22^ Hospital Universitario Cruces, Biobizkaia Health Research Institute, Barakaldo, Spain; ^23^ Hôpital de Hautepierre, Strasbourg, France; ^24^ Department of Pediatric Oncology, Centre Hospitalier Universitaire, Nantes, France; ^25^ Department of Pediatric Oncology, Pediatric Haematology and Oncology Institute, Léon Bérard Center, Lyon, France; ^26^ Sheffield Children's Hospital, Sheffield, United Kingdom; ^27^ Alder Hey Children's Hospital, Liverpool, United Kingdom; ^28^ Royal Victoria Infirmary Newcastle and Translational & Clinical Research Institute, Newcastle University, Newcastle upon Tyne, United Kingdom; ^29^ Leeds General Infirmary, Leeds, United Kingdom; ^30^ Birmingham Women and Children's Hospital, Birmingham, United Kingdom; ^31^ Gustave Roussy, Villejuif, France; ^32^ Institut Curie, Paris, France; ^33^ Hôpital d'Enfants, Nancy, France; ^34^ Centre Oscar Lambret, Lille, France; ^35^ Cliniques Universitaires Saint Luc, Brussels, Belgium; ^36^ NIHR Birmingham Biomedical Research Centre, Institute of Cancer and Genomic Sciences, College of Medical and Dental Sciences, University of Birmingham, Birmingham, United Kingdom

## Abstract

**PURPOSE:**

Outcomes for children with relapsed and refractory high-risk neuroblastoma (RR-HR-NBL) remain dismal. Here, we investigate addition of the anti-GD2 monoclonal antibody, dinutuximab beta (dB), to temozolomide (T)-based chemotherapy.

**MATERIALS AND METHODS:**

Patients with RR-HR-NBL were randomly assigned in a 1:2 ratio to receive chemotherapy alone or chemotherapy with dB, given concurrently as a 7-day infusion (10 mg/m^2^/24 h). The trial had a factorial design, with some patients also randomly assigned between chemotherapy regimens (T *v* T-topotecan [TTo]). Crossover to dB with To/cyclophosphamide was allowed for patients randomly assigned to chemotherapy alone with disease progression (PD). The primary outcome was best objective response (complete or partial) rate (overall response rate [ORR]) during six cycles of treatment. Progression-free (PFS), overall survival (OS), and safety were secondary outcomes.

**RESULTS:**

Sixty-five patients were randomly assigned to chemotherapy alone (3 T, 19 TTo) or with dB (6 dBT, 37 dBTTo). The median age was 4 years; 28 and 37 patients had refractory and relapsed diseases, respectively. Baseline characteristics were balanced between arms. The ORR was 30.2% (13 of 43) and 18.2% (4 of 22) in dB and non-dB arms, the median PFS was 11.1 months (95% CI, 4.3 to 15.5) for dB patients and 3.8 months (95% CI, 1.9 to 7.9) for non-dB patients, respectively. The median OS was 25.7 months (95% CI, 11.4 to not reached [NR]) for dB patients and 17.1 months (95% CI, 7.6 to 54.6) for non-dB patients (upper 95% CI, NR in dB arm). Thirteen of 22 patients in the non-dB arm crossed over to dB with cyclophosphamide/To because of PD. Neurotoxicity was more common in the dB arm (grade 1 and 2: 26% *v* 9%, grade 3: 2.3% *v* 4.5%), but other toxicities were similar.

**CONCLUSION:**

Within a randomized phase II setting, results observed with addition of dB to T-based chemotherapy in RR-HR-NB warrant further evaluation.

## INTRODUCTION

Neuroblastoma is a rare pediatric malignancy, which accounts for a disproportionately high number of childhood cancer deaths.^1,2^ Approximately half of patients have high-risk disease (high-risk neuroblastoma [HR-NBL]), based on internationally agreed risk factors.^3,4^ Despite intensive multimodal therapies, over 50% of these patients relapse or have disease refractory to frontline chemotherapy (relapsed and refractory HR-NBL [RR-HR-NBL]). The outcome for RR-HR-NBL remains poor, with 5-year survival rates <15%-20%.^5-7^

**CONTEXT**

**Key Objective**
The outcome for children with relapsed and refractory neuroblastoma is poor, and new therapeutic combinations are urgently needed. This study aimed to test if the addition of the anti-GD2 monoclonal antibody dinutuximab beta (dB) to a chemotherapy backbone can be used to improve outcomes in these patients.
**Knowledge Generated**
Sixty-five patients were randomly assigned to chemotherapy alone or chemotherapy with dB. This provided phase II evidence supporting the activity of dB-based chemoimmunotherapy in patients with relapsed and refractory neuroblastoma.
**Relevance *(S. Bhatia)***
Findings from this study can be used to develop clinical trials to provide definitive evidence of efficacy of dB-based chemoimmunotherapy in patients with relapsed and refractory neuroblastoma.*
*Relevance section written by *JCO* Associate Editor Smita Bhatia, MD, MPH, FASCO.

Several salvage chemotherapy regimens have been tested, mainly in single-arm phase II trials, with a wide range of response rates (0% to 64%).^5,7,8^

Anti-GD2 antibody therapy, given alone or with cytokines, improves outcome when given after consolidation therapy to patients with HR-NBL.^9,10^ More recently, several, mostly nonrandomized, studies have reported encouraging response rates, in both relapse/refractory and up-front settings, when anti-GD2 is given in conjunction with chemotherapy.^11-13^

The BEACON-Neuroblastoma trial, which opened in 2013, tested three backbone chemotherapy regimens (temozolomide [T], irinotecan-T, topotecan [To]-T) and addition of bevacizumab, which has been reported separately.^14^ When the bevacizumab random assignment completed in 2019, the trial was amended (BEACON-Immuno amendment) to test addition of an anti-GD2 antibody, dinutuximab beta (dB), to the chemotherapy backbone. Here, we report the results of this random assignment.

## MATERIALS AND METHODS

### Patients

Patients age 1 to ≤21 years with confirmed RR-HR-NBL were eligible regardless of the number of episodes of relapse. Refractory disease was defined as inadequate response to frontline therapy using SIOPEN criteria at the time of enrollment: three or more spots on metaiodobenzylguanidine (I^123^ MIBG) scan (with or without persistence of bone marrow disease). Measurable (as per RECIST 1.1) or evaluable disease (uptake on I-123 mIBG or fluorodeoxyglucose-positron emission tomography [FDG-PET] scan) was required for study entry. Previous treatment with T or chemo-immunotherapy was not allowed, but previous anti-GD2 or To was permitted.

Eligible patients had adequate performance status, organ and bone marrow function, and appropriate washout periods for previous therapies. Nonbleeding CNS lesions were permissible. Full eligibility criteria are provided in the trial protocol.^15^ All patients, parents, and/or legal guardians provided written informed consent and assent where appropriate.

### Trial Design and Interventions

BEACON-Immuno (ClinicalTrials.gov identifier: NCT02308527) was an open-label, randomized phase II trial. The dB random assignment was initially conducted with the ongoing T versus TTo chemotherapy random assignment following a 2 × 2 factorial design. This chemotherapy random assignment closed in January 2020 following an Independent Data Monitoring Committee (IDMC) recommendation, because of futility of T alone, after which all patients received the TTo backbone. Patients were randomly assigned with a 2:1 ratio, in favor of dB plus chemotherapy versus chemotherapy alone. dB was delivered as a concurrent 7-day continuous infusion (10 mg/m^2^/24 h) with each cycle of chemotherapy. Trial arms and details of chemotherapy doses are provided in Appendix Table A1 (online only), as well as the trial protocol.

Trial treatment was given for up to six cycles, each lasting 4 weeks. After six cycles, patients with responding or stable disease were allowed to continue trial treatment with chemotherapy only, for up to 12 cycles. Random assignment was stratified according to disease status: early relapse (<18 months from initial diagnosis), late relapse (≥18 months from initial diagnosis) or refractory disease, and the presence of measurable/evaluable disease, using minimization.

Patients allocated to receive chemotherapy alone, who had received at least two cycles of trial therapy, and whose disease relapsed/progressed within 30 months of starting trial treatment were allowed to cross over to dB given with cyclophosphamide and To.^16^

### Outcome Measures and Assessments

The primary outcome measure was objective response (OR; complete or partial) during the first six courses of trial therapy. Response was evaluated using RECIST 1.1 for patients with measurable disease.^17^ For those with only evaluable disease, response was evaluated using the 2017 International Neuroblastoma Response Criteria (INRC).^18^ For primary analysis, partial and complete responses (partial remission [PR] and clinical remission) were considered as OR.

Secondary outcome measures were toxicity of the regimens (Common Terminology Criteria Adverse Events [AEs] v4.0), progression-free survival (PFS), and overall survival (OS).

Disease was assessed at baseline and after every two cycles. Cross-sectional imaging (computed tomography/magnetic resonance imaging) of areas of suspected disease and I-123 mIBG scans (with calculated SIOPEN scores) were performed at each time point. Patients with mIBG nonavid disease were followed with FDG-PET scans. Bilateral bone marrow aspirates and trephine biopsies were mandatory at baseline and repeated every two cycles in patients with bone marrow involvement at study entry or under clinical suspicion of progression. All patients underwent CNS imaging at study entry.

The primary end point of response was evaluated using local investigator-based assessments entered in the study case report form (CRF). Before the preparation of this report, the lead investigators (J.G., L.M.) performed a review of all tumor assessments to detect inconsistencies between the results of investigations and overall response evaluation or between time points, which were clarified by the site investigators and updated on the trial database. Central review of imaging and bone marrow samples has not yet been performed.

As the 2017 INRC only became available during the trial, in 2024, using tumor measurements, bone marrow evaluations, and SIOPEN scores from the CRF, the lead investigators calculated the response per INRC for all patients, and upon agreement with site investigators, this was added to the database. For this post hoc INRC analysis, PR and CR (but not minor responses) were considered OR. This analysis is provided separately to facilitate clarity and comparability with other contemporaneous clinical trials.

### Statistical Design and Analysis

The statistical methods used were as outlined in the predefined statistical analysis plan, unless otherwise stated. Efficacy data were analyzed on an intention-to-treat basis, and safety data were reported for all patients who received at least one dose of a trial drug. The sample size for the dB random assignment was calculated to obtain a 20% increase in response rate with addition of dB, assuming a control arm response rate of 25%. A sample size of 64 patients in total was calculated to provide an 80% power with a one-sided *P* value of .23. The sample size was calculated to power a chi-squared test; however, at the time of the analysis, it was found that the assumptions for this had not been met, so the analysis was performed using a Fisher's exact test, assumptions for which were met. The sample size calculation was performed using sample size tables for clinical studies software. The calculation assumed no dropouts.

For the primary analysis of response, one-sided Fisher's exact tests were used to compare responses (CR/PR) between random assignment arms. Secondary analysis of response used generalized linear models with binomial distribution and log link to produce risk ratios with 80% CIs for dB treatment with response (responder or not) as an outcome variable and randomized treatment allocation (dB or not) as an independent variable with adjustment for the allocation in the other random assignment(s), disease status (early/late relapse, relapsed/refractory), and measurable/evaluable disease. Nonevaluable patients were included as nonresponders for the primary response analysis.

For PFS and OS, Cox regression models were fitted for the random assignment, with adjustment for the allocation in the other random assignment(s), disease status (early relapsed/late relapsed/refractory), and measurable/evaluable disease. The general rule of “10 events per df” was considered, and, of note, because of the limited number of deaths, the adjusted Cox model of OS may lead to overfitting. Hazard ratios (HRs) with 95% CIs were calculated for survival end points. The Cox proportional hazards (PHs) assumption was tested graphically using log(-log) plots to confirm that the assumption was not violated for PFS or OS. Interactions between dB and trial chemotherapy arms were explored by producing forest plots to test the level of heterogeneity, described by *I*^2^.

Database lock for the analyses presented here occurred on February 10, 2025. Analyses were performed using Stata Version 18.0.

### Trial Oversight

This trial was performed in accordance with the principles of the Declaration of Helsinki and Good Clinical Practice. The trial was designed by the Trial Management Group and Sponsor (University of Birmingham), in collaboration with the Innovative Therapies for Children with Cancer and European Neuroblastoma Research Network (SIOPEN) cooperative groups and overseen by an IDMC. The trial obtained first competent authority and ethics committee approval in the United Kingdom (West Midlands—Coventry and Warwickshire Research Ethics Committee, reference 13/WM/0023) and then obtained competent authority and ethics committee approval in all the countries where it opened. EUSA Pharma provided dB and gave advice on protocol development but had no role in trial conduct or data analysis.

## RESULTS

### Screening and Random Assignment

**FIG 1.**
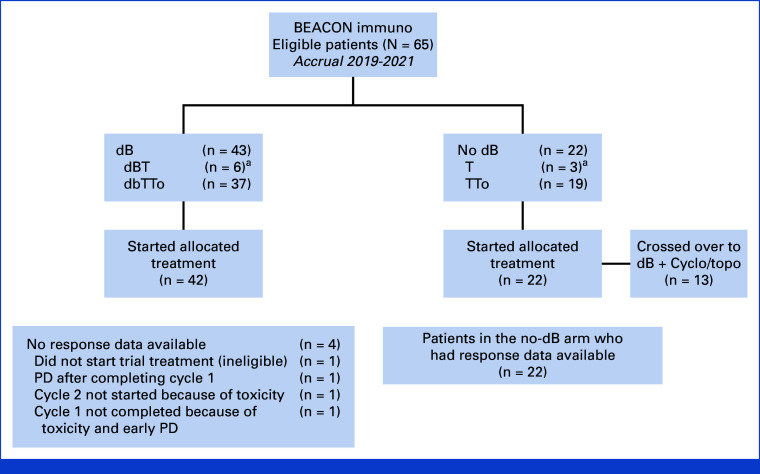
CONSORT diagram of the BEACON Immuno trial. ^a^The trial IDMC implemented an urgent safety measure in January 2020 stopping the To random assignment as outcomes for patients receiving T alone were inferior. From then on, all patients were randomly assigned to TTo or DbTTo. dB, dinutuximab beta; dBT, dB temozolomide; dBTTo, dB temozolomide-topotecan; IDMC, Independent Data Monitoring Committee; PD, disease progression; T, temozolomide; TTo, temozolomide-topotecan.

Between August 2019 and February 2021, 65 patients were randomly assigned from 29 sites in seven European countries. Forty-three patients were allocated to receive dB, and 22 to receive chemotherapy alone (Fig 1). As the concurrent chemotherapy random assignment (T *v* TTo) closed soon after the start of the dB random assignment, most patients (56 of 65) received TTo.

### Patient Characteristics

Age at enrollment ranged from 1 to 18 years (median 4 years; Table 1). Twenty-eight (43%) patients had refractory neuroblastoma, and 37 (57%) had relapsed neuroblastoma; 51 (78.5%) had measurable disease, and 14 (21.5%) had evaluable disease; 19 (29%) had *MYCN* amplification; 24 (37%) had received previous anti-GD2. The median follow-up for survivors was 3.9 years (IQR, 3.7-4.3 years).

**TABLE 1.** Characteristics of Patients in dB Random Assignment

Characteristic	No dB (n = 22)	dB (n = 43)	Overall (N = 65)	*P*
Median age, years (range)	4 (1-17)	4 (1-18)	4 (1-18)	.378
Age, years, No. (%)				.155
<2	3 (13.5)	1 (2)	4 (6)	
2 to <6	14 (64)	26 (61)	40 (62)	
6 to <12	2 (9)	12 (28)	14 (22)	
12 to <18	3 (13.5)	3 (7)	6 (9)	
≥18	0 (0)	1 (2)	1 (1)	
Initial INSS stage, No. (%)				.205
1	0 (0)	0 (0)	0 (0)	
2	3 (13.5)	1 (2)	4 (6)	
3	0 (0)	3 (7)	3 (5)	
4	18 (82)	32 (75)	50 (77)	
4S	1 (4.5)	2 (5)	3 (4.5)	
Not known	0 (0)	5 (11)	5 (7.5)	
*MYCN* amplification, No. (%)	6 (27)	13 (30)	19 (29)	.846
Present	15 (68)	29 (68)	44 (68)	
Absent to not known	1 (5)	1 (2)	2 (3)	
Induction chemotherapy, No (%)				1.000
COJEC	12 (54.5)	22 (51.2)	34 (52.3)	
COJEC + TVD	4 (18.2)	6 (14)	10 (15.4)	
COJEC + TVD + other	1 (4.6)	2 (4.6)	3 (4.6)	
COJEC + other	1 (4.6)	2 (4.6)	3 (4.6)	
N7	0 (0)	2 (4.6)	2 (3.1)	
Other	3 (13.6)	7 (16.3)	10 (15.4)	
Missing	1 (4.6)	2 (4.6)	3 (4.6)	
Previous major surgery, No. (%)	12 (55)	30 (70)	42 (65)	.255
High-dose chemotherapy with autologous stem-cell rescue, No. (%)	8 (36)	22 (51)	30 (46)	.257
Previous radiotherapy, No. (%)	9 (41)	18 (42)	27 (42)	.941
Previous anti-GD2 therapy, No. (%)	6 (27)	18 (42)	24 (37)	.249
PS, No. (%)				.524
Lanksy/Karnofsky				
90-100	19 (86.5)	30 (70)	49 (75)	
50-80	2 (9)	11 (25)	13 (20)	
Missing	1 (4.5)	2 (5)	3 (5)	
Disease status, No. (%)				.956
Refractory	9 (41)	19 (44)	28 (43.1)	
Early relapse	9 (41)	16 (37.2)	25 (38.4)	
Late relapse	4 (18.2)	8 (18.6)	12 (18.4)	
Patients with two or more relapse(s), No. (%)	3 (14)	3 (7)	6 (10)	.385
Disease evaluation at study entry, No. (%)				.268
Measurable disease	19 (86.4)	32 (74)	51 (78.5)	
Evaluable disease	3 (13.6)	11 (26)	14 (21.5)	

NOTE. There were no significance differences in the baseline characteristics between the arms; *P* values were calculated using *χ*^2^ (or Fisher's exact [if cell count <5]) tests for all categorical variables and the Wilcoxon rank-sum test for median age.

Abbreviations: COJEC, cisplatin, vincristine, carboplatin, etoposide, and cyclophosphamide; dB, dinutuximab beta; INSS, International Neuroblastoma Staging System; PS, performance status; TVD, topotecan, vincristine, doxorubicin.

Four patients (6.2%), all randomly assigned to receive dB, did not have response data available; one patient did not commence trial treatment as the patient no longer met eligibility criteria, one patient stopped treatment after cycle 1 because of disease progression (PD), and two patients did not continue beyond cycle 1 because of toxicity. For primary analysis, all these patients were considered nonresponders.

### Response Rate for dB Random Assignment

The overall response rate (ORRs) were 30.2% (13 of 43) in patients receiving dB with chemotherapy and 18.2% (4 of 22) in patients receiving chemotherapy alone (Table 2). Fisher's exact test produced a one-sided *P* value of .23. This did not pass the predefined failure threshold of the random assignment (one-sided *P* > .23 for primary analysis). The unadjusted risk ratio for response was 1.66 (80% CI, 0.87 to 3.19) or 1.42 (80% CI, 0.74 to 2.72), when adjusted for To administration, disease status, and measurable/evaluable disease, giving one-side *P* values of .16 (unadjusted) and .24 (adjusted), respectively.

**TABLE 2.** Evaluation of Best Responses by RECIST (patients with measurable disease) and INRC (patients with evaluable disease) in Patients Allocated to Receive No dB or dB

Best Response	No dB (n = 22), No. (%)	+ dB (n = 43), No. (%)
CR	1 (4.6)	5 (11.6)
PR	3 (13.6)	8 (18.6)
MR	0 (0)	2 (4.7)
SD	12 (54.5)	16 (37.2)
Progressive disease	6 (27.3)	8 (18.6)
Not evaluable	0 (0)	4 (9.3)
OR rate (PR + CR)	4 (18.2)	13 (30.2)

Abbreviations: CR, complete response; dB, dinutuximab beta; INRC, International Neuroblastoma Response Criteria; MR, minor response; OR, objective response; PR, partial response; SD, stable disease.

### Analysis of Response Per INRC

In addition, after calculating INRC response status for all patients on the trial, best ORR was analyzed by INRC only, taking best response by INRC for all patients and including patients with best response of CR and PR as responders (Appendix Table A2). The ORR was 27.9% (12 of 43) in patients receiving dB and 13.6% (3 of 22) in patients receiving chemotherapy alone.

### Crossover to Chemo-Immunotherapy From the Chemotherapy-Only Arm

Thirteen of 22 patients randomly assigned to receive chemotherapy alone subsequently crossed over to receive chemoimmunotherapy (dB + cyclophosphamide/To) because of PD. Patient characteristics for these patients are shown in Appendix Table A3.

### Survival Outcomes for dB Random Assignment

**FIG A1.**
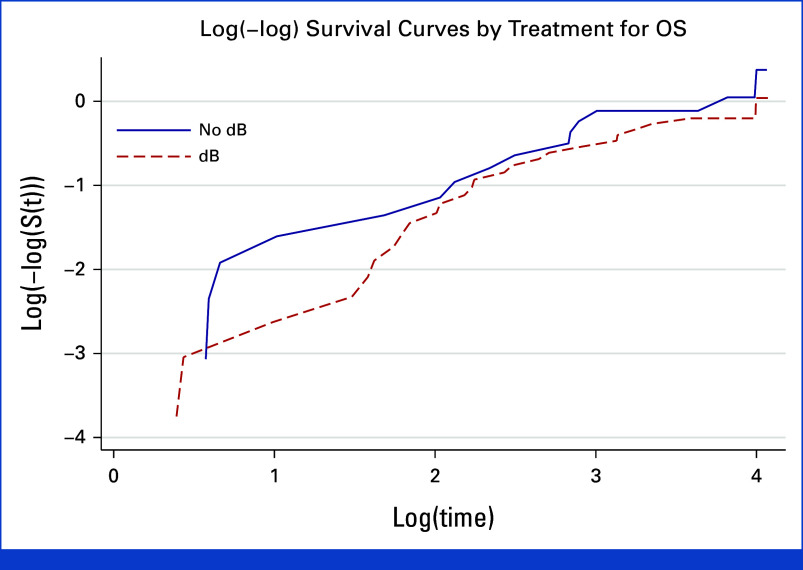
Forest plots for the dB random assignment. *I*^2^ indicates the level of heterogeneity, which is equal to 0, indicating that no significant interaction was observed. dB, dinutuximab beta; OS, overall survival.

**FIG A2.**
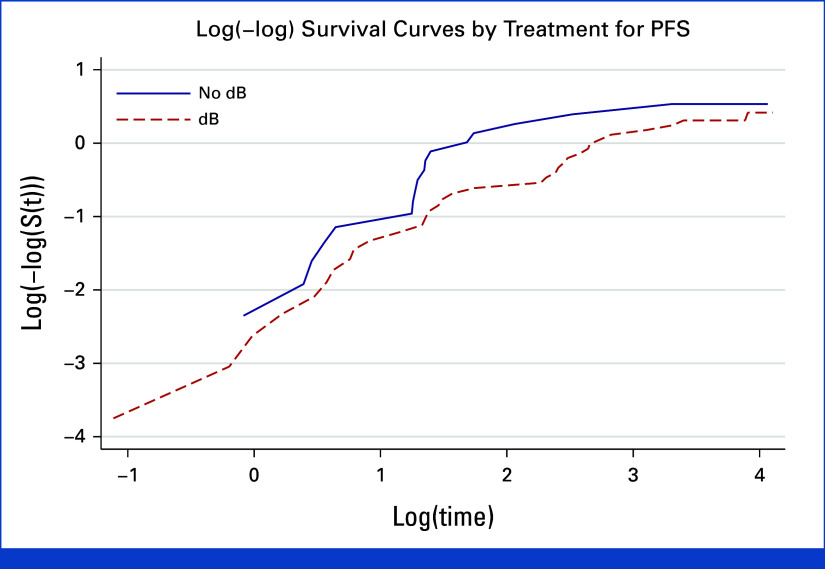
Log(-log) survival curves for OS for dinutuximab random assignment. dB, dinutuximab beta; OS, overall survival; PFS, progression-free survival.

The PHs assumption was tested for the Cox PH model of PFS and OS and was not violated; diagnostic log(-log) plots are presented in Appendix Figures A1 and A2, respectively.

**FIG 2.**
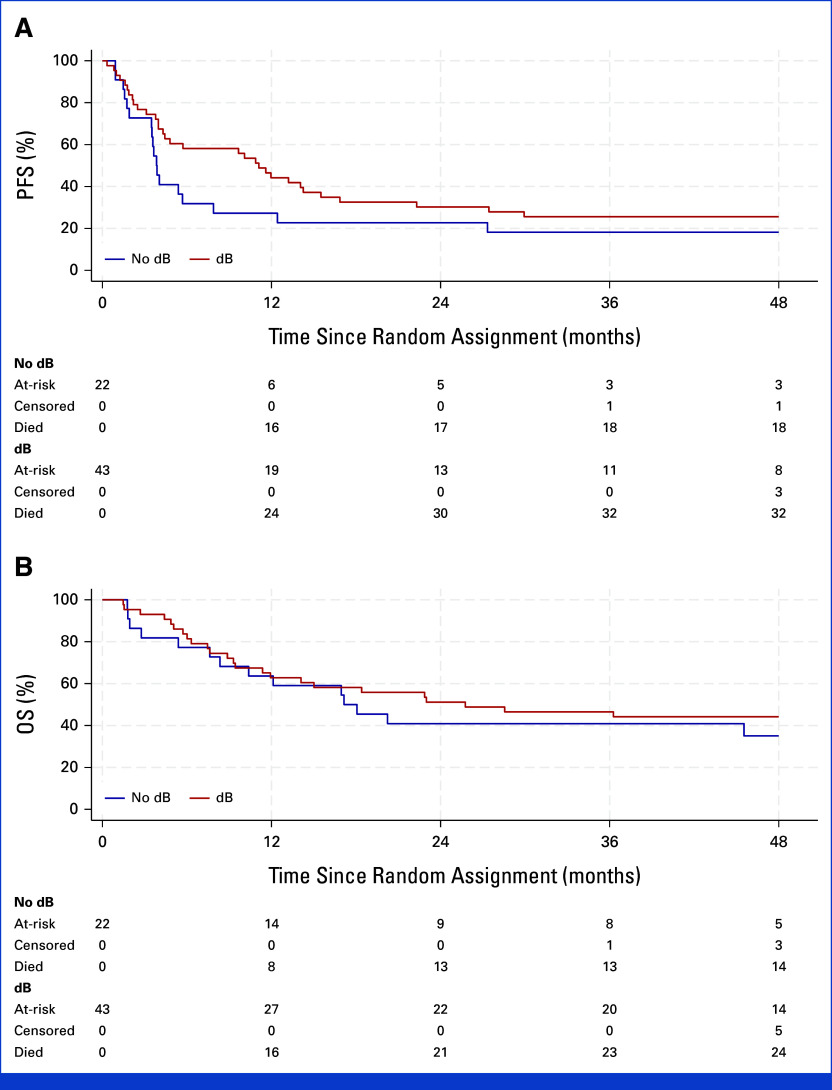
Survival analysis in the BEACON Immuno trial. (A) PFS and (B) OS for patients randomly assigned to no dB versus dB. The risk table shows the number of patients at risk, who have died or who have been censored at each time point. dB, dinutuximab beta; OS, overall survival; PFS, progression-free survival.

The HR for PFS was 0.80 (95% CI, 0.44 to 1.46; *P* = .48) when adjusted for To administration, disease status, and measurable/evaluable disease. The 1-year PFS was 44% for patients randomly assigned to receive dB, compared with 27% for patients receiving chemotherapy alone (Fig 2A). The median PFS was 11.1 months (95% CI, 4.3 to 15.5) for dB patients and 3.8 months (95% CI, 1.9 to 7.9) for non-dB patients.

OS was not significantly different between random assignment arms (Fig 2B), with an HR of 1.01 (95% CI, 0.53 to 1.96; *P* = .97) when adjusted for To allocation and stratification factors. The 1-year OS was 63% and 64% for dB and non-dB patients, respectively. The median OS was 25.7 months (95% CI, 11.4 to ) for dB and 17.1 months (95% CI, 7.6 to 54.6) for non-dB patients. However, 13 of 22 patients in the no-dB arm had crossed over, because of PD/relapse, to receive dB with cyclophosphamide/To, making this comparison uninformative.

### Potential Interactions

**FIG A3.**
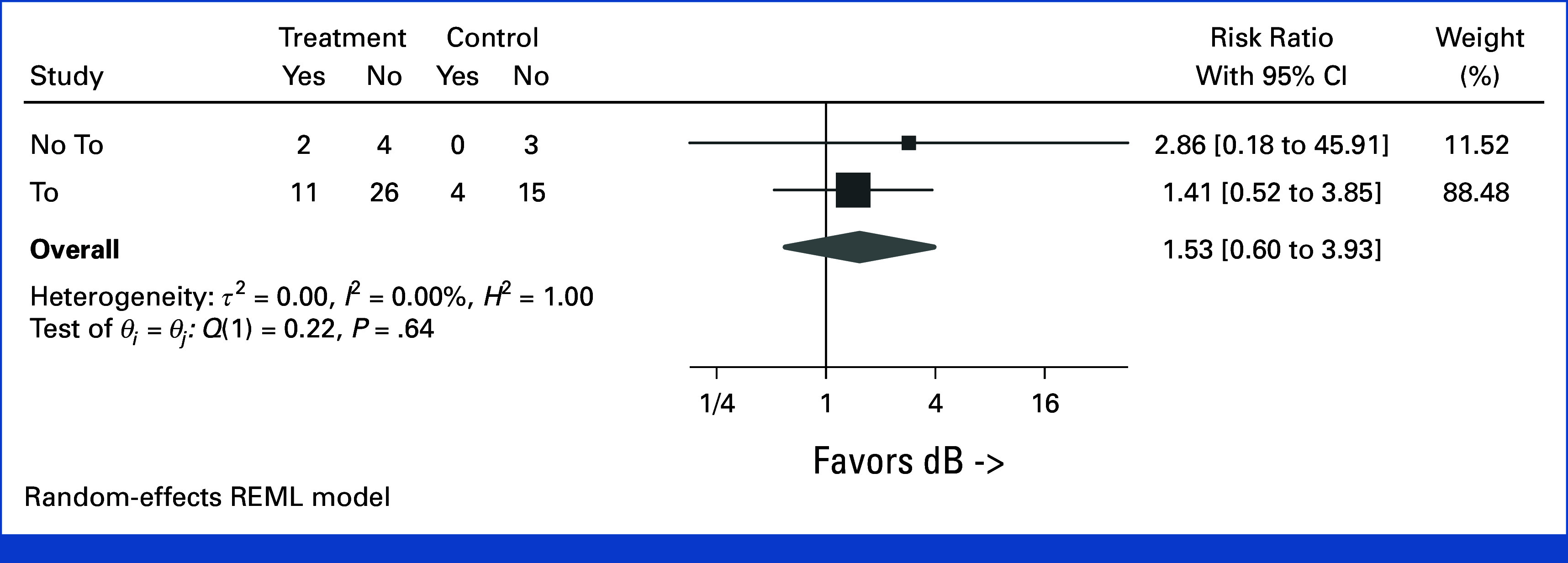
Log(-log) survival curves for PFS for dB random assignment. dB, dinutuximab beta; PFS, progression-free survival; REML, residual maximal likelihood; To, topotecan.

The trial design assumed no interaction between dB and the different chemotherapy regimens. At trial completion, interactions were explored by heterogeneity tests between individual arms (Appendix Fig A3) and no significant interaction was observed.

### Safety of Administration of dB Plus Chemotherapy

Grade 3 or worse AEs are shown in Table 3. dB given with chemotherapy did not increase the incidence of AEs, other than neurologic toxicities. Neurologic toxicities were mostly mild, with grade 1 and 2 toxicities in 26% of patients receiving dB and 9% of patients receiving chemotherapy alone (Table 4). One patient receiving dB developed grade 3 myelitis, which had improved to grade 2 after stopping dB. One patient receiving chemotherapy alone experienced a grade 3 seizure. Notably, pain was well-controlled in most patients, with only 16.0% of dB patients and 9.0% of non-dB patients experiencing grade 3 pain.

**TABLE 3.** Grade ≥3 CTCAE According to dB Random Assignment Maximum Grade Experienced

CTCAE Category	dB Random Assignment (maximum grade experienced: grade 3 and above AEs), No. of AEs (% of patients experiencing AE in the arm)		
	No dB (T, TTo; n = 22)	dB (dBT, dBTTo; n = 43)	Overall (N = 65)
Investigations	30 (73)	55 (58)	85 (63)
Platelet count decreased	14 (64)	13 (30)	27 (45)
Neutrophil count decreased	9 (41)	13 (30)	22 (34)
White blood cell decreased	3 (14)	7 (16)	10 (17)
Lymphocyte count decreased	3 (14)	7 (16)	10 (15)
GGT increased	0 (0)	5 (12)	5 (11)
Alanine aminotransferase increased	0 (0)	6 (14)	6 (9)
Aspartate amino transferase increased	1 (4.5)	4 (9)	5 (8)
Blood and lymphatic system disorders	17 (55)	22 (40)	39 (45)
Anemia	11 (50)	13 (30)	24 (38)
Febrile neutropenia	5 (23)	7 (16)	12 (20)
Leukocytosis	0 (0)	1 (2)	1 (1.5)
Disseminated intravascular coagulation	0 (0)	1 (2)	1 (1.5)
Thrombotic thrombocytopenic purpura	1 (4.5)	0 (0)	1 (1.5)
Metabolism and nutrition disorders	2 (9)	8 (19)	10 (15)
Hypokalemia	1 (4.5)	3 (7)	4 (6)
Hyperglycemia	0 (0)	1 (2)	1 (1.5)
Anorexia	1 (4.5)	1 (2)	2 (3)
Hypoglycemia	0 (0)	1 (2)	1 (1.5)
Tumor lysis syndrome	0 (0)	1 (2)	1 (1.5)
Undernutrition	0 (0)	1 (2)	1 (1.5)
GI disorders	6 (18)	10 (16)	16 (17)
Vomiting	1 (4.5)	3 (7)	4 (6)
Abdominal pain	2 (9)	3 (7)	5 (8)
Nausea	0 (0)	2 (4.5)	2 (3)
Diarrhea	1 (4.5)	1 (2)	2 (3)
Enterocolitis	0 (0)	1 (2)	1 (1.5)
Ascites	1 (4.5)	0 (0)	1 (1.5)
Intra-abdominal hemorrhage	1 (4.5)	0 (0)	1 (1.5)
Infections and infestations	8 (36)	9 (16)	17 (23)
Catheter-related infection	1 (4.5)	2 (4.5)	3 (6)
Sepsis	1 (4.5)	2 (4.5)	3 (4.5)
Pharyngitis	0 (0)	1 (2)	1 (1.5)
Upper respiratory infection	0 (0)	3 (7)	3 (4.5)
Infectious enterocolitis	0 (0)	1 (2)	1 (1.5)
Bladder infection	1 (4.5)	0 (0)	1 (1.5)
Lower respiratory tract infection	2 (9)	0 (0)	2 (3)
Bacterial infection	1 (4.5)	0 (0)	1 (1.5)
Central venous line infection	1 (4.5)	0 (0)	1 (1.5)
Device-related infection	1 (4.5)	0 (0)	1 (1.5)
General disorders and administration site conditions	2 (9)	10 (23)	12 (18.5)
Pain	1 (4.5)	5 (12)	6 (9)
Fever	1 (4.5)	3 (7)	4 (8)
Noncardiac chest pain	0 (0)	1 (2)	1 (1.5)
Lower limb pain	0 (0)	1 (2)	1 (1.5)
Respiratory, thoracic, and mediastinal disorders	3 (14)	5 (9)	8 (11)
Hypoxia	2 (9)	3 (7)	5 (8)
Epistaxis	0 (0)	1 (2)	1 (1.5)
Dyspnea	1 (4.5)	1 (2)	2 (1.5)
Nervous system disorders	1 (4.5)	1 (2)	2 (3)
Myelitis	0 (0)	1 (2)	1 (1.5)
Seizure	1 (4.5)	0 (0)	1 (1.5)
Musculoskeletal and connective tissue disorders	2 (4.5)	2 (4.5)	4 (4.5)
Arthralgia	0 (0)	1 (2)	1 (1.5)
Back pain	1 (4.5)	1 (2)	2 (3)
Neck pain	1 (4.5)	0 (0)	1 (1.5)
Vascular disorders	1 (4.5)	3 (7)	4 (6)
Capillary leak syndrome	0 (0)	1 (2)	1 (1.5)
Hypotension	1 (4.5)	1 (2)	2 (3)
Visceral arterial ischemia	0 (0)	1 (2)	1 (1.5)
Skin and subcutaneous tissue disorders	0 (0)	2 (4.5)	2 (3)
Maculopapular rash	0 (0)	1 (2)	1 (1.5)
Urticaria	0 (0)	1 (2)	1 (1.5)
Surgical and medical procedures	0 (0)	1 (2)	1 (1.5)
Broken central line	0 (0)	1 (2)	1 (1.5)
Injury, poisoning, and procedural complications	0 (0)	1 (2)	1 (1.5)
Aortic injury	0 (0)	1 (2)	1 (1.5)
Immune system disorders	0 (0)	1 (2)	1 (1.5)
Allergic reaction	0 (0)	1 (2)	1 (1.5)
Reproductive system and breast disorders	1 (4.5)	0 (0)	1 (1.5)
Vaginal hemorrhage	1 (4.5)	0 (0)	1 (1.5)
Total No. of AEs	73	130	203

Abbreviations: AE, adverse event; CTCAE, Common Terminology Criteria AE; dB, dinutuximab beta; dBT, dB temozolomide; dBTTo, dB temozolomide-topotecan; GGT, γ-glutamyl transferase; TTo, temozolomide-topotecan.

**TABLE 4.** Neurologic AEs, All CTCAE Grades

Maximum CTCAE Grade Experienced	No. of AEs (No. of patients experiencing AE grade)	
	No dB (n = 22)	+dB (n = 43)
Grade 1		
Somnolence	1 (1)	4 (4)
Dizziness	0	3 (3)
Neuralgia	0	1 (1)
Headache	0	2 (2)
Paresthesia/dysesthesia	0	2 (2)
Dysarthria	1 (1)	0
Depressed level of consciousness	1 (1)	0
Total	3 (2)	12 (9)
Grade 2		
Neuralgia	0	1 (1)
Lethargy	0	1 (1)
Headache	0	1 (1)
Paresthesia	0	1 (1)
Nystagmus	0	1 (1)
Total	0	5 (5)
Grade 3		
Myelitis	0	1 (1)
Seizure	1 (1)	0
Total	1 (1)	1 (1)
Overall	4 (3)	18 (12)

NOTE. Maximum grade experienced.

Abbreviations: AE, adverse event; CTCAE, Common Terminology Criteria AE; dB, dinutuximab beta.

## DISCUSSION

The BEACON Immuno trial is the largest randomized trial to date testing addition of anti-GD2 antibody to chemotherapy in patients with RR-HR-NBL. In the context of an established multiarm multistage, factorial design, international cooperative group academic trial, with an ongoing chemotherapy random assignment, to our knowledge, it is the first randomized study to investigate the addition of dB to chemotherapy. Response rates were 30.2% in patients receiving dB and 18.2% in those receiving chemotherapy alone (adjusted risk ratio, 1.42 [80% CI, 0.74 to 2.72]). The median PFS with chemo-immunotherapy was 11.1 months (95% CI, 4.3 to 15.5) for dB patients compared with 3.8 months (95% CI, 1.9 to 7.9) for non-dB patients (adjusted HR, 0.80 [95% CI, 0.44 to 1.46]; *P* = .48). As 13 of 22 (59%) patients in the chemotherapy-alone arm crossed over to alternative chemoimmunotherapy (dB + cyclophosphamide/To) at further relapse/progression, analysis of OS was uninterpretable. Despite this limitation, the crossover was considered essential in the trial design to ensure acceptability to parents and clinicians as nontrial access to chemo-immunotherapy was not possible in many European countries.

Previous single-arm studies of dB chemoimmunotherapy, including prospective clinical trials and retrospective studies, have reported response rates of 36%-64%^19-21^ in RR-HR-NBL.^19-21^ Other studies have reported similar results with other anti-GD2 antibodies; the US Children's Oncology Group ANBL1221 study reported an ORR of 41.5% and a 1-year PFS of 67.9% in patients administered dinutuximab with irinotecan, T, and granulocyte-macrophage colony-stimulating factor.^12^ In contrast to the BEACON Immuno random assignment, the ANBL1221 study was limited to patients experiencing first relapse of neuroblastoma. In addition, the ANBL1221 study included a more intensive and longer anti-GD2 dosing schedule, with 70 mg/m^2^ (17.5 mg/m^2^ infusion per day on four successive days given every 21 days) dinutuximab for up to 17 cycles, as compared with 70 mg/m^2^ (as a seven-day continuous infusion) dinutuximab beta given every 28 days for just six cycles in BEACON Immuno. Similarly, Lerman et al^22^ reported an ORR of 49% (71 of 146) and a 1-year PFS of 50% in a large, retrospective, cohort study. Broadly similar response rates have also been reported when the humanized anti-GD2 antibodies Hu14.18K322A and naxitamab are combined with chemotherapy.^23,24^

The absolute ORR difference of 12% reported here is smaller than the targeted 20% difference, and the response rates observed are lower than those reported in the above studies. However, the corresponding *P* value of .23 is at the threshold of significance by design, and therefore, it is believed that this result represents a clinically meaningful signal. The one-sided *P* value threshold of .23 was prespecified to maintain an 80% power under a 2:1 random assignment and reflects a common statistical approach in exploratory phase II oncology studies with feasibility constraints.

Furthermore, comparing efficacy across different studies is challenging, given the small potentially heterogenous patient populations. The influence of different inclusion criteria (eg, restricted to first relapse), longer trial treatment duration, and different dosing schedules is difficult to assess. The heterogenous nature of the patient populations highlights the importance of randomized trials, but as illustrated in this study, the rarity of the patient population (even in a pan-European trial) limits recruitment and the ability to obtain statistically significant results in a timely manner.

The factorial design of this trial allowed different questions to be addressed simultaneously. While advantageous in a rare disease, this means that patients did not all receive the same chemotherapy backbone, and interactions between arms cannot be completely excluded.

The 2017 INRC only became available during the trial, and the primary analysis used RECIST 1.1 to assess patients with measurable disease. RECIST has limitations in fully assessing the extent of neuroblastoma, and a subsequent analysis of all patients' response according to 2017 INRC was therefore performed. The results of this were broadly similar to the primary analysis but should be interpreted in the context of a post hoc analysis. Despite these limitations, to our knowledge, this is the first randomized study of chemo-immunotherapy using dB, the most widely used anti-GD2 antibody outside of the United States, and the results of this trial will potentially broaden global access to chemo-immunotherapy. The fact that the analysis of PFS shows a clear short-term improvement, but that survival beyond this is broadly similar, highlights the need for longer chemo-immunotherapy, more potent chemo-immunotherapy combinations, or improved consolidation options, such as CAR-T-cell therapy.^25^

Importantly, dB given with chemotherapy was generally well-tolerated. Although neurologic toxicities were more common in patients receiving dB, most were mild and consistent with the known toxicity profile.^10,26^ Notably, pain was generally well-controlled, with only 16.0% of dB patients experiencing grade 3 pain. The incidence of other AEs was similar in both arms. While this study did not include quality of life or patient-reported outcomes, the overall toxicity burden of this chemoimmunotherapy in this population of heavily pretreated patients appears to be acceptable. A parallel biological study, BEACON-BIO, using samples obtained from patients recruited to the trial, is ongoing and will correlate genomics, immune parameters, biomarkers of resistance, and pharmacokinetics with clinical outcomes.

Before the dB random assignment, the BEACON trial investigated the addition of bevacizumab to the chemotherapy backbone. These results, reported separately, suggest benefit of bevacizumab, with the combination of bevacizumab, irinotecan, and T (BIT) appearing particularly promising (the ORR was 30%, and the 1-year PFS was 67%).^14^ The recently opened BEACON-2 platform trial will directly compare BIT with dB chemo-immunotherapy in patients with first relapse and will also test combining both bevacizumab and dB with chemotherapy in patients with subsequent relapses. In the BEACON-2 trial, the chemoimmunotherapy has been intensified (given every 3 weeks rather than every 4 weeks) and prolonged to up to 12 cycles.

In conclusion, within a randomized phase II setting, results observed with addition of dB to T-based chemotherapy in RR-HR-NB warrant further evaluation. Further clinical trials are required to provide definitive evidence of efficacy and to evaluate in comparison with other combinational therapies.

## Protocols





## Data Availability

A data sharing statement provided by the authors is available with this article at DOI https://doi.org/10.1200/JCO-25-01868.

## CLINICAL TRIAL INFORMATION

NCT02308527 (BEACON Immuno)

## APPENDIX

**TABLE A1.** Summary of dB Random Assignment Trial Treatment

Arm	Drug and Dose, Dose Frequncy	Frequency of Cycles
Arm T		
Days 1-5	T: 200 mg/m^2^ once daily for 5 days PO	Every 4 weeks
Arm dBT		
Day 1-7	dB: 10 mg/m^2^/d 24 hours continuous IV infusion	Every 4 weeks
Days 1-5	T: 200 mg/m^2^/ once daily for 5 days PO	Every 4 weeks
Arm TTo		
Days 1-5	T: 150 mg/m^2 ^once daily for 5 days PO	Every 4 weeks
Days 1-5	To: 0.75 mg/m^2^ once daily for 5 days IV	Every 4 weeks
Arm dBTTo		
Day 1-7	dB: 10 mg/m^2^/d 24 hours continuous IV infusion	Every 4 weeks
Days 1-5	T: 150 mg/m^2 ^once daily for 5 days PO	Every 4 weeks
Days 1-5	To: 0.75 mg/m^2^ once daily for 5 days IV	Every 4 weeks

Abbreviations: dB, dinutuximab beta; dBT, dB temozolomide; dBTTo, dB temozolomide-topotecan; IV, intravenous; PO, orally; T, temozolomide; TTo, temozolomide-topotecan.

**TABLE A2.** Evaluation of Best Responses by INRC (CR, PR, MR, SD, PD) in Patients Allocated to Receive No dB or dB

Best Response INRC Only	No dB (n = 22), No. (%)	+dB (n = 43), No. (%)
CR	1 (4.6)	5 (11.6)
PR	2 (9.1)	7 (16.3)
MR	5 (22.7)	8 (18.6)
SD	8 (36.4)	10 (23.3)
Progressive disease	6 (27.3)	8 (18.6)
Not evaluable	0 (0)	5 (11.6)
OR rate (PR + CR)	3 (13.6)	12 (27.9)

Abbreviations: CR, complete response; INRC, International Neuroblastoma Response Criteria; MR, minor response; OR, objective response; PD, disease progression; PR, partial response; SD, stable disease.

**TABLE A3.** Characteristics of Patients Allocated to Receive Chemotherapy Alone Who Crossed Over to Receive Chemo-Immunotherapy With To/Cyclophosphamide Because of PD.

Patient Characteristic	Overall (n = 13)
Trial treatment allocated, No. (%)	
T	2 (15)
T and To	11 (85)
Median age, years (range)	4 (1-12)
Age, years, No. (%)	
<2	1 (8)
2 to <6	9 (69)
6–12	3 (23)
Disease status	
Refractory	5 (38)
Early relapse	4 (31)
Late relapse	4 (31)
Disease evaluation at study entry, No. (%)	
Measurable disease	13 (100)
Evaluable disease	0
Median No. of cycles received before crossover (range)	4 (2-8)
Cycles of allocated trial treatment received, No. (%)	
2	2 (15)
4	5 (38)
5	1 (8)
6	4 (31)
8	1 (8)
Median time from the start of trial treatment to PD/relapse, days (range)	115 (52-827)

Abbreviations: dB, dinutuximab beta; T, temozolomide; PD, disease progression.
