# Supplementary material for: Dinutuximab Beta Added to Temozolomide-Based Chemotherapy for Children With Relapsed and Refractory Neuroblastoma: Results of the ITCC-SIOPEN BEACON Immuno Phase II Trial
Source: J Clin Oncol. 2025 Dec 12;44(3):176–87. doi: 10.1200/JCO-25-01868 (PMC12794700; doi:10.1200/JCO-25-01868)
Supplement: Supplementary file 2 [file jco-44-176-s002.pdf]

A randomised phase IIb trial of BEvACizumab added to Temozolomide ± IrinOtecan for children with refractory/relapsed Neuroblastoma

BEACON-Neuroblastoma

Version 8.0 dated 07-Mar-2023

Dinutuximab beta amendment

Coordinating Sponsor:	University of Birmingham
Sponsor Protocol Number:	RG_ 11-087
CAS Code:	BN2008
EudraCT Number:	2012-000072-42
ISRCTN Reference Number:	40708286
ITCC Number:	032
Roche Study Reference Number:	MO28245
Email:	beacon@trials.bham.ac.uk

This application is supported by the facilities funded through Birmingham Science City: Translational Medicine Clinical Research Infrastructure and Trials Platform, an Advantage West Midlands (AWM) funded project which forms part of the Science City University of Warwick and University of Birmingham Research Alliance.

SIGNATURE PAGE

BEACON-Neuroblastoma Trial Protocol – Version 8.0 07 Mar 2023
This protocol has been approved by:

Name:	Dr Lucas Moreno	Trial Role:	Chief Investigator
Signature:		Date:	09 MAR 2023

This protocol describes the BEACON-Neuroblastoma trial and provides information about procedures for patients taking part in the BEACON-Neuroblastoma trial. The protocol should not be used as a guide for treatment of patients not taking part in the BEACON-Neuroblastoma trial.

AMENDMENTS

The following amendments and/or administrative changes have been made to this protocol since the implementation of the first approved version

Amendment number	Date of amendment	Protocol version number	Type of amendment	Summary of amendment
SA 1	29-Jan-2013	2.0	Substantial Amendment	Introduction of the recommendation of weekly monitoring of blood counts for all patients receiving irinotecan.<br>Addition of planned vaccination with live vaccination to exclusion criteria and prohibited medications section.
N/A	23-Apr-2013	2.0a	Non-Substantial Amendment	ITCC Number has been corrected.<br>Roche Study Reference Number and ISRCTN Reference Number have been added.<br>Contact details for Plasma & Tumour Angiogenesis-Related Biomarkers have been amended.<br>Table numbers have been corrected.<br>Addition of guidance for research bone marrow sampling in Schedule of Activities table and sections 7.4.2.2 and 7.5.2.
N/A	01-Jul-2013	2.0b	Non-Substantial Amendment	Addition of paragraph to sections 7.2, 7.6.1 – 7.6.4 detailing arrangements for handling dose modifications for Irinotecan ± Temozolomide for patients receiving Bevacizumab.<br>Discontinuation rules for osteonecrosis of the jaw and eye disorders added to table 13 in section 7.6.4.<br>Correction to table number references in section 7.6.3.<br>Clarification made in section 13.4.1 concerning Planned Interim Analysis.
SA 3	06-Oct-2014	4.0	Substantial Amendment	Changes to the Trial Personnel section of the protocol to include the addition of contact details for Denmark and Ireland Lead Investigators.<br>Amendments to reflect the changes in study sampling requirements to Trial Synopsis, Schedule of Activities table and sections 1.2.6, 2.1, 2.2, 5.1, 5.2, 7.3, 7.4.2 and 7.5.<br>Amendment to exclusion criteria in Trial Synopsis and section 4.2.<br>Changes to the Schedule of Activities table to include the addition of an echocardiogram to be performed at screening and Tanner staging at screening and yearly in follow up.<br>Changes to the time line for measuring renal function prior to commencing treatment in the Trial Synopsis, Schedule of Activities and section 4.1.

				Option to fax emergency randomisation removed. Telephone only in section 6.2<br>Changes to guidelines in section 7.2 for dose calculation in patients whose weight exceeds the 98th centile for age.<br>Removal of enhanced data collection for Adverse Events of Special Interest (AESI) in section 7.6 and 9.1.2.<br>Addition of option to extend treatment delay with agreement from Sponsor in Section 7.6.1<br>Addition of necrotising fasciitis as an adverse event requiring bevacizumab discontinuation in section 7.6.4.<br>Changes to section 7.10 concerning the documenting of concomitant medications in patient medical notes and administration of bisphosphonates.<br>Changes to section 9.1 regarding reporting of laboratory adverse events.<br>Clarification on the arrangements for Follow Up Form completion for patients who do not require further follow up visits in section 11.<br>Changes to bevacizumab and irinotecan preparation and dispensing guidelines in sections 8.2.4 and 8.3.3.<br>Clarification on fasting arrangements prior to temozolomide administration added to section 8.4.3.<br>Changes to events that should be reported on an Expected SAR Form in section 9.1.3.1.<br>Clarification on SAEs that should be reported to F.Hoffman-La Roche Ltd in section 9.2.6<br>Addition of Trial Management Group meeting frequency in section 14.4.<br>Changes to the wording of irinotecan randomisation in section 13.4.2.<br>Addition of guidelines for dose reduction and discontinuation of temozolomide for liver toxicity in tables 8, 9, 10 & 11.<br>Reference to the National Coordinating Centres has been changed to National Co-Sponsor throughout.<br>Reference to Sponsor has been changed to Coordinating Sponsor.
SA 4	06-Oct-2014	4.0	Substantial Amendment	Change of Chief Investigator to Professor Pamela Kearns. Change of Principal Investigator at Royal Marsden Hospital to Dr Sucheta Vaidya. No changes made to the Protocol version.
SA 5	30-Jul-2015	5.0	Substantial Amendment	Chief Investigator and UK Lead Investigator changed to Dr Lucas Moreno<br>Switzerland details added<br>Schedule of events table amended for End Of Treatment clarity

				Topotecan added to the study as a new trial question and 2 new randomisation arms. The following sections are amended accordingly:<br>Synopsis (Primary Objectives, sample size, Trial Duration, Trial therapy)<br>Section 1 Background and rationale (Trial rationale)<br>Section 3 Trial design (Randomisation)<br>Section7.1 and 7.2 Treatment details<br>Section7.6.1 Dose modifications Table 6 amended, Tables 11 and 12 added<br>Section 8.4 Pharmaceutical Information<br>Section 13 Statistical considerations<br><br>The following changes were made to the Eligibility:<br>Inclusion criteria – further details regarding birth control<br>Exclusion criteria – Defined wash out period following prior IMP according to IMP half-life or 14 days.<br>Lifestyle guidelines - further details regarding birth control<br><br>Section 7.6 Dose modifications Figure 1 – reference to “chemo” changed to Temozolomide/Irinotecan/Topotecan” for clarity<br>Section 8.2.4 Reference to “chemo” removed for clarity<br>Section 7.6.4 AEs requiring Bevacizumab discontinuation – additional AEs added following Bevacizumab IB v22 Addendum<br><br>Additional mRNA and exploratory sampling.<br><br>The requirement for confirmatory scans was removed from the Schedule of Activities and Response assessment section 7.4.3.<br>Lead Investigator for France amended to Dr Marion Gambart<br>Minor wording corrections and clarifications
N/A	23-Sep-2015	5.0a	Non-Substantial Amendment	Schedule of events table corrected
SA 6	16-Jan-2019	6.0a	Substantial amendment	Introduction of two new treatment arms (dinutuximab beta) for additional 64 patients<br>Addition of eligibility criteria, schedule of events, treatment details, duration, cross over and dose modification details for new dinutuximab beta arms<br>Adaptation of objectives, trial design, supporting treatment, pharmaceutical information and statistical consideration sections with new, relevant information.

				Minor wording corrections and clarifications<br>Version amended from 6.0 to 6.0a to add. Additional rationale to update typographical errors.)
N/A	11-Apr-2019	6.0b	Non-Substantial Amendment	Trial Synopsis: Clarification of recruitment targets<br>Clarification of Section 10 title: “Dinutuximab beta and topotecan randomisations”<br>Clarification that not all biological studies will be open at any one time (Section 10.2 and 15.5)<br>Minor wording corrections and clarifications
SA 7	07-Feb-2020	7.0	Substantial Amendment	Urgent Safety Measure – implemented on 28th January 2020<br>Closure of Temozolomide (T) and Dinutuximab beta and Temozolomide (dBT) arms with immediate effect.<br>Section 1.1 Background<br>Section 1.2.3 Benefit Risk assessment<br>Section 3.1 Randomisation<br>Section 10 Headings changed<br>Section 10.3 Trial therapy<br>Update of contact details
SA 22	07-Mar-2023	8.0	Substantial Amendment	Change of definition of End of Trial (Section 21). Protocol previously defined two stages of end of trial (6 months after last patient completes treatment and 12 months after last data capture after 5 years follow up). This has been combined into one End of Trial definition: 6 months after last patient last visit (i.e. after 5 years follow up)<br>Also addition of option to email SAE form (Section 18.2.1.2)

TRIAL PERSONNEL

Chief Investigator: **Dr Lucas Moreno**

Dr. Lucas Moreno, MD, PhD
Director
Paediatric Oncology & Haematology Division
Vall d'Hebron Barcelona Hospital Campus
Passeig de la Vall d'Hebron, 119-129,
08035, Barcelona, Spain
☎ +34 93 489 3000
🖷 +34 93 489 4060
✉ lucas.moreno@vhebron.net

Co-Investigators: Professor Keith Wheatley

Professor of Clinical Trials
Cancer Research UK Clinical Trials Unit (CRCTU)
Institute of Cancer and Genomic Sciences
College of Medical and Dental Sciences
University of Birmingham
Birmingham, UK
B15 2TT
☎ +44 (0)121 415 9119
✉ k.wheatley@bham.ac.uk

Dr Juliet Gray
Associate Professor and Consultant in Paediatric Oncology
Southampton Children's Hospital
Tremona Road
Southampton
SO16 6YD
☎ +44 (0) 790 1507929
✉ juliet.gray@uhs.nhs.uk

Exploratory Biomarkers Dr Gudrun Schleiermacher

Senior Scientist
Institute Curie
26 rue d'Ulm 75248 Paris cedex 05
France
☎ +33 (0)1 56 24 45 50
🖷 +33 (0)1 56 24 66 30
✉ gudrun.schleiermacher@curie.net

Professor Louis Chesler
Paediatric Tumour Biology Team
Institute of Cancer Research and Royal Marsden Hospital
(University of London), Downs Road, Sutton
Surrey, UK
SM2 5PT
☎ +44 (0) 208 722 4035

✉ louis.chesler@icr.ac.uk

Functional Imaging Study:

Professor Andrew Peet
Institute of Child Health
University of Birmingham
Whittall Street
Birmingham, UK
B4 6NH
☎ +44 (0) 121 333 8234
🖷 +44 (0) 121 333 8241
✉ a.peet@bham.ac.uk

Dr Dow-Mu Koh
Consultant Radiologist in Functional Imaging
Royal Marsden Hospital
Downs Road,
Sutton, Surrey, UK
SM2 5PT
☎ +44 (0) 208 6613857
✉ dow-mu.koh@icr.ac.uk

Professor Martin Leach
Co-Director, Cancer Research UK and EPSRC Centre for Cancer Imaging, Director, NIHR Clinical Research Facility
Deputy Head, Division of Radiotherapy and Imaging
Institute of Cancer Research and Royal Marsden Hospital (University of London), Downs Road, Sutton
Surrey, UK
SM2 5PT
☎ +44 (0 208 661 3338
✉ Martin.Leach@icr.ac.uk

Molecular Monitoring mRNA Study:

Professor Sue Burchill
Leeds Institute of Cancer & Pathology
St. James University Hospital
Beckett Street
Leeds, UK
LS9 7TF
☎ +44 (0) 113 206 5873
🖷 +44 (0) 113 242 9886
✉ S.A.Burchill@leeds.ac.uk

Professor Walter Gregory
Clinical Trials Research Unit (CTRU)
University of Leeds Clinical Trials Research House
71-75 Clarendon Road
Leeds
LS2 9PH
☎ +44 (0) 113 343 1489
🖷 +44 (0) 113 343 1471

✉ W.M.Gregory@leeds.ac.uk

Trial Statistician:

Miss Grace Holt
Cancer Research UK Clinical Trials Unit (CRCTU)
Institute of Cancer and Genomic Sciences
College of Medical and Dental Sciences
University of Birmingham
Birmingham, UK
B15 2TT
☎ +44 (0)121 414 8328
🖶 +44 (0)121 414 3700
✉ G.C.Holt@bham.ac.uk

Trial Coordinator & Trial Office:

Miss Punam Mistry
Children's Cancer Trials Team
Cancer Research UK Clinical Trials Unit (CRCTU)
Institute of Cancer and Genomic Sciences
College of Medical and Dental Sciences
University of Birmingham
Birmingham, UK
B15 2TT
☎ +44 (0)121 414 3788
🖶 +44 (0)121 414 9520
✉ beacon@trials.bham.ac.uk

Randomisation Service:

Provided by the CRCTU at the University of Birmingham

Randomisation should be performed by sites online at:
https://www.cancertrials.bham.ac.uk/BEACONLive

In case of any problems with online randomisation, randomisation can be performed over the phone by the CRCTU on:
☎ 0800 371 969 or +44 (0)121 414 3366

SAE Reporting:

SAEs should be faxed to the
BEACON-Neuroblastoma Trial Office,
CRCTU,
University of Birmingham, UK
🖶 + 44 (0)121 414 9520 or +44 (0)121 414 3700

National Coordinating Investigators:

Austria – Lead Investigator:
Prof Dr Ruth Ladenstein
St. Anna Children's Hospital and CCRI /Studies and Statistics
Department for Integrated Research and Projects (S²IRP)
Kinderspitalgasse 6, Zimmermannplatz 10
A-1090 Vienna
Austria
☎ +43-1-40470-4750
🖶 +43-1- 40470- 7430
✉ ruth.ladenstein@ccri.at

Belgium Lead Investigator:
Prof Genevieve Laureys
Ghent University Hospital
9000 Ghent
De Pinterlaan 185
Belgium
☎ +32 93 32 34 48
✉ genevieve.laureys@uzgent.be

Denmark – Lead Investigator:
Dr Karsten Nysom
Dept. of Paediatrics & Adolescent Medicine
Rigshospitalet
Blegdamsvej 9
DK2100 Copenhagen
Denmark
☎ +45 35 45 08 09
🖷 +45 35 45 50 55
✉ Karsten.nysom@regionh.dk

France – Lead Investigator:
Dr Marion Gambart
Unité d'Hémato-Oncologie
Hôpital des Enfants
330, avenue de Grande Bretagne
TSA 70034
31059 Toulouse Cedex France
☎ +33 (0)5 34 55 86 11
🖷 +33 (0)5 34 55 86 12
✉ gambart.m@chu-toulouse.fr

Germany - Lead Investigator:
Dr. Simone Hettmer
Zentrum für Kinder- und Jugendmedizin
UNIVERSITÄTSKLINIKUM FREIBURG
Mathildenstr. 1, 79106 Freiburg
Germany
☎ +49 761 270-43000
🖷 +49 761 270-45180
✉ simone.hettmer@uniklinik-freiburg.de

Ireland – Lead Investigator:
Dr Cormac Owens
Our Lady's Children's Hospital
Crumlin Road, Crumlin
Dublin 12
Ireland
☎ +35314096659
🖷 +35313453041
✉ Cormac.owens@olchc.ie

Italy – Lead Investigator:
Dr. Aurora Castellano
U.O.Oncoematologia Ospedale Pediatrico Bambino Gesù Pzza S. Onofrio 4
00165 Roma
Italy
☎ +39 06 68592957-2678
🖷 +39 06 68592826
✉ aurora.castellano@opbg.net

Netherlands – Lead Investigator:
Dr. C Michel Zwaan
Erasmus Medical Center
Sophia's Children's Hospital
Dr. Molewaterplein 60
3015 GJ Rotterdam
☎ +31 (0) 10 703 6691
🖷 +31(0) 10 703 6681
✉ c.m.zwaan@erasmusmc.nl

Spain – Lead Investigator:
Dr. Victoria Castel
Instituto de Investigación Sanitaria
Unidad de Oncología Pediátrica
Hospital Universitario La Fe
Bulevar Sur, S/N
46026 Valencia
Spain
☎ +34 963862758 Ext 50040
🖷 +34 963494416
✉ castel_vic@gva.es

Switzerland – Lead Investigator:
Dr. Nicolas Gerber
University Children's Hospital,
Steinwiesstrasse 75,
CH-8032 Zurich,
Switzerland
☎ +41 44 266 31 17
🖷 +41 44 266 34 61
✉ Nicolas.gerber@kispi.uzh.ch

UK – Lead Investigator:
Dr Lucas Moreno
Honorary Research Fellow
University of Birmingham
Birmingham, UK
B15 2TT
☎ +44 (0)121 414 3788
🖷 +44 (0)121 414 9520
✉ lucas.moreno@vhebron.net, lmorenom@ext.cnio.es

TRIAL SYNOPSIS

Title
A randomised phase IIb trial of bevacizumab added to temozolomide ± irinotecan for children with refractory/relapsed neuroblastoma – BEACON-Neuroblastoma Trial

Trial Design
A phase II, randomised, open label, international multicentre 3x2 factorial trial. The dinutuximab beta amendment did utilise a 2x2 factorial design it will now be a simple two-way randomisation.

Objectives

Primary:
- To test whether bevacizumab added to a backbone chemotherapy regimen (temozolomide, irinotecan + temozolomide or temozolomide + topotecan) demonstrates activity in children with relapsed or refractory neuroblastoma
- To test whether the addition of irinotecan to temozolomide increases the activity of chemotherapy in children with relapsed or refractory neuroblastoma
- To test whether the addition of topotecan to temozolomide increases the activity of chemotherapy in children with relapsed or refractory neuroblastoma (“topotecan randomisation”)
- To test whether dinutuximab beta added to a backbone chemotherapy regimen (temozolomide or temozolomide + topotecan) demonstrates activity in children with relapsed or refractory neuroblastoma (“dinutuximab beta randomisation”)

Secondary:
- To evaluate the safety of the regimens

Tertiary:
- To undertake preliminary evaluation of the changes in magnetic resonance imaging (MRI) derived functional imaging biomarkers of angiogenesis
- To undertake preliminary evaluation of the role of circulating mRNA levels for tyrosine hydroxylase (TH), paired-like homeobox 2b (PHOX2B) and doublecortin (DCX) as prognostic/predictive biomarkers in this refractory/relapsed setting
- To undertake a preliminary evaluation of the role of tumour molecular profiles in blood and archival tumour tissue profiles as prognostic and predictive biomarkers
- To undertake a preliminary evaluation of biomarkers of response to anti-GD2 therapy (Fc/KIR polymorphisms, Antibody Dependant Cell-Mediated Cytotoxicity (ADCC) and Anti-Drug Antibodies (ADAs) and of dinutuximab beta pharmacokinetics (PK)

Outcome Measures
Primary Endpoint:
- Best response (Complete Response [CR] or Partial Response [PR]) [1] at any time during the first 6 cycles of trial treatment
- For the bevacizumab part 2 only: Progression-free survival (PFS)

Secondary Endpoints:
- Safety of the regimens: Incidence and severity of Adverse Events (AE)s
- PFS
- Overall survival (OS)
- Event-free survival (EFS)

Exploratory/Tertiary Endpoints:

- Changes in (MRI) derived functional imaging biomarkers of angiogenesis measured by quantitative dynamic contrast enhanced (DCE) MRI: primary biomarkers will be the transfer constant K^{trans} [min^{-1}] and initial area under the gadolinium uptake curve from 0 to 60 seconds ($IAUGC_{60}$, mM Gd min) and secondary biomarkers will be tumour apparent diffusion coefficient (ADC, 10^{-6} cm^2 s^{-1}), native T1 and T2 relaxation times (ms) and transverse relaxation rate R^{2*}
- Changes in circulating mRNA levels for TH, PHOX2B and DCX in bone marrow and blood samples
- Pilot descriptive study of angiogenesis and neuroblastoma markers that may include O6-methylguanine-methyltransferase (MGMT) status, immunohistochemistry and immunofluorescence markers on tumour samples (such as microvessel density (MVD), CD31, Ki67, NRP1, VEGFR-1, VEGFR-2, C-KIT), DNA/RNA extraction from tissue sections for tumour mutation screening and tumour expression profiling
- A preliminary correlation of the different biomarkers [Fc/KIR polymorphisms, Antibody – Dependent Cellular Toxicity (ADCC), and Anti-Drug Antibodies (ADAs)] will be made with parameters of anti-tumour activity (response rate, PFS and OS). PK parameters (dinutuximab beta trough levels) for this chemo-immunotherapy regimen will be described.

Patient Population

Children and young adults aged 1 to 21 years of age with relapsed/refractory neuroblastoma.

Sample Size

Approximately 224 patients, including 160 for the bevacizumab randomisation and 64 for the dinutuximab beta amendment.

Trial Duration

8 years of patient recruitment, 5 years of patient follow up

Abbreviations

ADA	ANTI-DRUG ANTIBODIES
ADCC	ANTIBODY – DEPENDENT CELL-MEDIATED CYTOTOXICITY
AE	ADVERSE EVENT
AESI	ADVERSE EVENT OF SPECIAL INTEREST
AFSAPPS	COMPETENT AUTHORITY FOR FRANCE
ALT	ALANINE AMINOTRANSFERASE
ANC	ABSOLUTE NEUTROPHIL COUNT
APPT	ACTIVATED PARTIAL THROMBOPLASTIN TIME
ASCT	AUTOLOGOUS STEM CELL TRANSPLANTATION
AST	ASPARTATE AMINOTRANSFERASE
AUC	AREA UNDER THE CURVE
AR	ADVERSE REACTION
BIT	BEVACIZUMAB + IRINOTECAN + TEMOZOLOMIDE ARM
BM	BONE MARROW
BP	BLOOD PRESSURE
BSA	BODY SURFACE AREA
BT	BEVACIZUMAB + TEMOZOLOMIDE ARM
BTTo	BEVACIZUMAB + TEMOZOLOMIDE + TOPOTECAN ARM
CI	CHIEF INVESTIGATOR
CIs	CONFIDENCE INTERVALS
COG	CHILDREN'S ONCOLOGY GROUP
CNS	CENTRAL NERVOUS SYSTEM
CR	COMPLETE RESPONSE
CRF	CASE REPORT FORM
CR UK	CANCER RESEARCH UK
CRCTU	CANCER RESEARCH UK CLINICAL TRIALS UNIT (UNIVERSITY OF BIRMINGHAM)
CRN	CLINICAL RESEARCH NETWORK
CSR	CLINICAL STUDY REPORT
CT	COMPUTERISED TOMOGRAPHY
CTC	COMMON TERMINOLOGY CRITERIA
CTCAE	COMMON TERMINOLOGY CRITERIA FOR ADVERSE EVENTS
CXR	CHEST X-RAY
dBT	DINUTUXIMAB BETA + TEMOZOLOMIDE ARM
dBTTo	DINUTUXIMAB BETA + TEMOZOLOMIDE + TOPOTECAN ARM
DCX	DOUBLECORTIN
DLT	DOSE LIMITING TOXICITY
DMC	DATA MONITORING COMMITTEE
DNA	DEOXYRIBONUCLEIC ACID
ECHO	ECHOCARDIOGRAM
ECOG	EASTERN COOPERATIVE ONCOLOGY GROUP
EFS	EVENT FREE SURVIVAL
EMA	EUROPEAN MEDICINES AGENCY
ERDC	ELECTRONIC REMOTE DATA CAPTURE
EOT	END OF TREATMENT
FFPE	FORMALIN-FIXED PARAFFIN EMBEDDED
GCP	GOOD CLINICAL PRACTICE
G-CSF	GRANULOCYTE COLONY STIMULATING FACTOR

GFR	GLOMERULAR FILTRATION RATE
GM-CSF	GRANULOCYTE-MONOCYTE COLONY STIMULATING FACTOR
GGT	GAMMA-GLUTAMYL TRANSPEPTIDASE
GP	GENERAL PRACTITIONER
GPOH	GERMAN SOCIETY FOR PAEDIATRIC ONCOLOGY & HAEMATOLOGY
HR	HEART RATE
IB	INVESTIGATOR BROCHURE
ICF	INFORMED CONSENT FORM
ICH	INTERNATIONAL CONFERENCE ON HARMONISATION
IMP	INVESTIGATIONAL MEDICINAL PRODUCT
INR	INTERNATIONAL NORMALISED RATIO
INRC	INTERNATIONAL NEUROBLASTOMA RESPONSE CRITERIA
INRG	INTERNATIONAL NEUROBLASTOMA RISK GROUP
INSS	INTERNATIONAL NEUROBLASTOMA STAGING SYSTEM
IRF	INSTITUTIONAL REVIEW BOARD
ISF	INVESTIGATOR SITE FILE
IT	IRINOTECAN + TEMOZOLOMIDE ARM
ITCC	INNOVATIVE THERAPIES FOR CHILDREN WITH CANCER
IV	INTRAVENOUS
MGMT	O6-METHYGUANINE METHYLTRANSFERASE
MIBG	META-IODO-BENZYL-GUANIDINE
MHRA	MEDICINES AND HEALTHCARE PRODUCTS REGULATORY AGENCY
MRD	MINIMAL RESIDUAL DISEASE
MRI	MAGNETIC RESONANCE IMAGING
MSKCC	MEMORIAL SLOAN KETTERING CANCER CENTRE
MTD	MAXIMUM TOLERATED DOSE
MYCN	MYELOCYTOMATOSIS VIRAL RELATED ONCOGENE
NANT	NEW AGENTS FOR NEUROBLASTOMA THERAPY
NCI	NATIONAL COORDINATING INVESTIGATOR
NCS	NATIONAL CO-SPONSOR
NR	NO RESPONSE
OS	OVERALL SURVIVAL
OTC	OVER THE COUNTER
PCP	PNEUMOCYSTIS CARNI PNEUMONITIS
PD	PROGRESSSIVE DISEASE
PFS	PROGRESSION FREE SURVIVAL
PHOX2B	PAIRED-LIKE HOMEOBOX2B
PI	PRINCIPAL INVESTIGATOR
PIS	PATIENT INFORMATION SHEET
PK	PHARMACOKINETICS
PMA	POPULATION-MODELLING ANALYSIS
PRES	POSTERIOR REVERSIBLE ENCEPHALOPATHY SYNDROME
PO	ORALLY
PPTP	PAEDIATRIC PRECLINICAL TESTING PROGRAM
PR	PARTIAL RESPONSE
REC	RESEARCH ETHICS COMMITTEE
RECIST	RESPONSE EVALUATION CRITERIA IN SOLID TUMOURS
RNA	RIBONUCLEIC ACID

RTKI	RECEPTOR TYROSINE KINASE INHIBITORS
RT-qPCR	REVERSE TRANSCRIPTASE QUANTITATIVE POLYMERASE CHAIN REACTION
SAE	SERIOUS ADVERSE EVENT
SAR	SERIOUS ADVERSE REACTION
SCT	STEM CELL TRANSPLANT
SD	STABLE DISEASE
SFOP	FRENCH SOCIETY OF PAEDIATRIC ONCOLOGY
SIOPEN	INTERNATIONAL SOCIETY PAEDIATRIC ONCOLOGY EUROPEAN NEUROBLASTOMA GROUP
SNP	SINGLE NUCLEOTIDE POLYMORPHISM
SPC	SUMMARY OF PRODUCT CHARACTERISTICS
SUSAR	SUSPECTED UNEXPECTED SEVERE ADVERSE REACTION
SWFI	STERILE WATER FOR INJECTION
T	TEMOZOLOMIDE ARM
TH	TYROSINE HYDROXYLASE
TMA	TISSUE MICROARRAY
TMG	TRIAL MANAGEMENT GROUP
TSC	TRIAL STEERING COMMITTEE
TTo	TEMOZOLOMIDE + TOPOTECAN ARM
TVD	TOPOTECAN, VINCRISTINE & DOXORUBICIN
UAR	UNEXPECTED ADVERSE REACTION
UKCCSG	UNITED KINGDOM CHILDREN'S CANCER STUDY GROUP
ULN	UPPER LIMIT OF NORMAL
VTE	VENOUS THROMBO-EMBOLISM
VEGF	VASCULAR ENDOTHELIAL GROWTH FACTOR
VGPR	VERY GOOD PARTIAL RESPONSE
WMA	WORLD MEDICAL ASSOCIATION

FORMULAE

Mosteller formula:

BSA (m²) =

$$BSA[m^2] = \sqrt{\frac{bodyweight[kg] \cdot bodyheight[cm]}{3600}}$$

Table of Contents

Trial Synopsis **12**
Title 12
Trial Design 12
Objectives 12
Outcome Measures 12
Patient Population 13
Sample Size 13
Trial Duration 13
Abbreviations 14
1. Background and Rationale **22**
1.1 Background 22
1.1.1 Background for the dinutuximab beta amendment 28
1.2 Trial Rational 29
1.2.1 Justification for design 29
1.2.2 Rationale for patient population 30
1.2.3 Benefit-risk assessment 31
1.2.4 Rationale for the selected backbone schedules: Temozolomide, irinotecan + temozolomide and temozolomide + topotecan 31
1.2.5 Rationale for dosing schedule of bevacizumab 32
1.2.6 Rationale for evaluating chemo-immunotherapy in the BEACON-Neuroblastoma Trial 32
1.2.7 Rationale for dosing schedule of dinutuximab beta 33
1.2.8 Rationale for the use of biomarker studies 34
1.3 Relevance and future importance 35
2. Objectives and Outcome Measures **36**
2.1 Objectives 36
2.2 Outcome Measures 37
3. Trial Design **37**
3.1 Randomisation 37
3.2 Duration of treatment 38
3.3 Frequency and duration of follow-up 38
4. Eligibility **39**
4.1 Lifestyle guidelines 39
5. Schedule of activities **39**
6. Screening and Consent **40**
6.1 Informed Consent 40
6.2 Screening 41
7. Trial Entry **42**
7.1 Procedure for online patient randomisation 42
7.2 Emergency Randomisation 42
8. Treatment Details **43**
8.1 Definition of Investigational Medicinal Products (IMPs) 43
9. Bevacizumab randomisation **43**
9.1 Eligibility criteria for the bevacizumab randomisation 43
9.1.1 Inclusion criteria for the bevacizumab randomisation 43
9.1.2 Exclusion criteria for the bevacizumab randomisation 44
9.2 Schedule of activities for the bevacizumab randomisation 45

9.3 Trial Therapy (bevacizumab randomisation) ... 49
9.3.1 Bevacizumab randomisation trial treatment ... 49
9.4 Treatment Schedule ... 51
9.4.1 Day 1 of Cycle 1 ... 51
9.4.2 Day 1 of subsequent cycles ... 51
9.4.3 Post Cycle 6 (For patients continuing to Cycle 7-12) ... 52
9.4.4 End of Treatment ... 52
9.4.5 Treatment Duration ... 52
9.5 Dose Modifications for the bevacizumab randomisation ... 53
9.5.1 Dose Modifications for AEs due to chemotherapy - for the bevacizumab randomisation ... 56
9.5.2 Bevacizumab – Infusion-related Reaction/Infusional Site Extravasation Management Guidelines ... 62
9.5.3 Bevacizumab - Treatment Delays ... 63
9.5.4 Bevacizumab - Discontinuation ... 64
9.5.5 Bevacizumab - Toxicity Management guidelines ... 65
9.6 Central Venous Access Device (CVAD) ... 67
10 Dinutuximab beta and topotecan randomisations ... 68
10.1 Eligibility for the dinutuximab beta randomisation ... 68
10.1.1 Inclusion criteria for the dinutuximab beta randomisation ... 68
10.1.2 Exclusion criteria for the dinutuximab beta randomisation ... 69
10.2 Schedule of events for the dinutuximab beta and topotecan randomisations ... 70
10.3 Trial therapy (dinutuximab beta and topotecan randomisations) ... 74
10.3.1 Dinutuximab beta and topotecan trial treatment ... 75
10.4 Treatment Schedule ... 76
10.4.1 Day 1 of Cycle 1 ... 76
10.4.2 Day 1 of subsequent cycles ... 76
10.4.3 Post Cycle 6 (For patients continuing to Cycle 7-12 on chemotherapy only) ... 77
10.4.4 End of Treatment ... 77
10.4.5 Treatment Duration ... 78
10.4.6 Cross-over ... 78
10.5 Dose Modifications – dinutuximab beta and topotecan randomisations ... 79
10.5.1 Dose modifications for dinutuximab beta specific toxicities ... 79
10.5.2 Dose modifications for haematological toxicity ... 81
10.5.3 Dose modifications for hepatic toxicity ... 82
11 Treatment Compliance ... 84
12 Supportive Treatment ... 84
12.1 Nausea and Vomiting ... 84
12.2 Growth Factors ... 84
12.3 Fever and neutropenia ... 84
12.4 Blood products ... 84
12.5 Pneumocystis jirovecii pneumonia (PJP) prophylaxis ... 84
12.6 Management of side effects caused by non-selective NSAIDs as cyclooxygenase (COX) type I and II inhibitors ... 84
12.7 Supportive care during Dinutuximab beta infusion ... 85
12.7.1 Pain Management ... 85
12.7.2 Prevention of dinutuximab beta related infusion reactions ... 86
13 Concomitant Medication ... 87
14 Assessments ... 87

14.1 Response assessment ... 88
15 Biomarkers ... 88
15.1 Blood sampling safety ... 88
15.2 MRI-derived functional imaging biomarkers of angiogenesis ... 90
15.3 Molecular monitoring mRNA ... 90
15.4 Neuroblastoma exploratory biomarker analyses ... 90
15.5 Sample Collection ... 92
15.5.1 Peripheral blood samples ... 92
15.5.2 Bone Marrow Samples ... 92
15.5.3 Archival tumour samples ... 92
16 Patient Follow Up ... 93
16.1 Patient Withdrawal ... 93
17 Pharmaceutical Information ... 94
17.1 Definition of Investigational Medicinal Product ... 94
17.2 Bevacizumab ... 94
17.2.1 Bevacizumab - Drug Supply ... 94
17.2.2 Bevacizumab - Ordering ... 94
17.2.3 Bevacizumab - Formulation, Packaging and Labelling ... 94
17.2.4 Bevacizumab - Preparation and Dispensing ... 95
17.2.5 Compatibility information ... 95
17.2.6 Bevacizumab - Administration ... 95
17.2.7 Bevacizumab – Accountability ... 96
17.2.8 Bevacizumab - Destruction ... 96
17.3 Cyclophosphamide ... 96
17.4 Dinutuximab beta ... 96
17.5 Irinotecan ... 96
17.5.1 Irinotecan - Drug Supply ... 96
17.5.2 Irinotecan - Formulation, Packaging and Labelling ... 96
17.5.3 Irinotecan - Preparation and Dispensing ... 97
17.5.4 Compatibility information ... 97
17.5.5 Irinotecan - Administration ... 97
17.6 Temozolomide ... 97
17.6.1 Temozolomide – Drug Supply ... 97
17.6.2 Temozolomide - Formulation, Packaging and Labelling ... 97
17.6.3 Temozolomide - Administration ... 97
17.7 Topotecan ... 98
17.7.1 Topotecan - Drug Supply ... 98
17.7.2 Topotecan - Formulation, Packaging and Labelling ... 98
17.7.3 Topotecan - Preparation and Dispensing ... 98
17.7.4 Topotecan - Compatibility information ... 98
17.7.5 Topotecan - Administration ... 99
18 Adverse Event Reporting ... 99
18.1 Reporting Requirements ... 99
18.1.1 Adverse Events (AE) ... 99
18.1.2 AESIs of Bevacizumab ... 99
18.1.3 Serious Adverse Advents (SAE) ... 99
18.1.4 Reporting period ... 100
18.2 Reporting Procedure ... 100

18.2.1 Site ... 100
18.2.2 Trial Office ... 102
18.2.3 Reporting to the Competent Authority and main Research Ethics Committee ... 102
18.2.4 Investigators ... 102
18.2.5 Data Monitoring Committee ... 102
18.2.6 Manufacturer of Investigational Medicinal Product ... 102
19 Data Handling and Record Keeping ... 103
19.1 Data Collection ... 103
19.2 Archiving ... 103
20 Quality Management ... 103
20.1 Site Set-up and Initiation ... 103
20.2 On-site Monitoring ... 104
20.3 Central Monitoring ... 104
20.4 Audit and Inspection ... 104
20.5 Notification of Serious Breaches ... 104
21 End of Trial Definition ... 105
22 Statistical Considerations ... 105
22.1 Trial Design ... 105
22.2 Definition of Outcome Measures ... 106
22.2.1 Primary ... 106
22.2.2 Secondary ... 106
22.2.3 Exploratory/Tertiary ... 106
22.3 Sample Size ... 106
22.4 Interim and Main Analyses of Outcome Measures ... 108
22.4.1 Planned Interim Analyses ... 108
22.4.2 Main Analysis ... 108
22.5 Stopping Guidelines ... 110
23 Trial Organisational Structure ... 110
23.1 Coordinating Sponsor ... 110
23.2 Co-Sponsor Centres ... 110
23.3 Relationship of trial committees ... 112
23.4 Trial Management Group ... 112
23.5 Trial Steering Committee ... 112
23.6 Data Monitoring Committee ... 112
23.7 Finance ... 113
23.8 NIHR CRN Portfolio ... 113
24 Ethical Considerations ... 113
25 Confidentiality and Data Protection ... 114
26 Insurance and Indemnity ... 115
27 Publication Policy ... 116
28 Reference List ... 117
Appendix 1 – WMA Declaration of Helsinki ... 124
Appendix 2 - Definition of Adverse Events ... 127
Appendix 3 - Common Toxicity Criteria Grading ... 129
Appendix 4 – RECIST Criteria 1.1 ... 130
Appendix 5 – Tumor Response at Metastatic Soft Tissue and Bone Sites (Park et al. 2017) ... 133
Appendix 6 - CURIE & SIOPEN scoring methods for neuroblastoma ... 134
Appendix 7 – Temozolomide Dosing ... 136

Appendix 8 – Blood Pressure Levels by Age and Height Percentile for Children and Adolescents ... 139
Appendix 9 – Height for Age Chart - Girls ... 144
Appendix 10 – Height for Age Chart - Boys ... 149
Appendix 11 – Lansky and Karnofsky/ECOG Scales ... 154
Appendix 12 – Tanner Staging ... 156
Appendix 13 – Clinical studies of anti-GD2 therapies in combination with chemotherapy ... 157

LIST OF TABLES

Table 1 - Second line chemotherapy regimens tested in phase II in relapsed or refractory neuroblastoma since 2000 ... 23
Table 2: Comparison of toxicity of regimens in relapsed or refractory neuroblastoma ... 24
Table 3 Preclinical studies using VEGF inhibitors in neuroblastoma ... 25
Table 4: IMPs in the BEACON-Neuroblastoma Trial ... 43
Table 5: Treatment Schedule ... 49
Table 6: Dose levels for dose adjustments ... 56
Table 7 - Arm T - Dose reduction and discontinuation rules ... 57
Table 8: Arm BT - Dose reduction and discontinuation rules ... 57
Table 9: Arm IT - Dose reduction and discontinuation rules ... 58
Table 10: Arm BIT - Dose reduction and discontinuation rules ... 59
Table 11: Arm TTo - Dose reduction and discontinuation rules ... 60
Table 12: Arm BTTo - Dose reduction and discontinuation rules ... 61
Table 13: Bevacizumab - Infusion-related reaction/infusional site extravasation management guidelines ... 62
Table 14: Adverse Events requiring bevacizumab treatment delay ... 64
Table 15: Adverse Events requiring discontinuation of bevacizumab ... 64
Table 16: Bevacizumab- toxicity management guidelines ... 65
Table 17: Treatment Schedule in the BEACON-Neuroblastoma - dinutuximab beta randomisation ... 75
Table 18: Cross-over treatment overview ... 78
Table 19: Dose modification for dinutuximab beta specific toxicities ... 79
Table 20: Dose levels for dose adjustments in the dinutuximab beta randomisation ... 81
Table 21: Dose reduction and discontinuation rules for AEs attributed to chemotherapy – All arms as applicable ... 81
Table 22: Dose modifications for hepatic toxicity in the dinutuximab beta randomisation ... 82
Table 23: EMA Recommendations for Trial related Blood Loss ... 89
Table 24: BEACON dinutuximab beta amendment: Blood volumes for immune monitoring ... 90
Table 25: Scenario probabilities based on probability-based Bayesian approach (n=60 per arm) ... 107

LIST OF FIGURES

Figure 1: Classification of Adverse Events in the BEACON-Neuroblastoma Trial (bevacizumab randomisation) ... 55
Figure 2 Dinutuximab beta amendment trial therapy overview ... 74
Figure 3: Probability plots for irinotecan and topotecan questions ... 109
Figure 4: Relationship of Trial Committees ... 112
Figure 5: The CURIE Method Figure 6: The SIOPEN Method ... 134

1. BACKGROUND AND RATIONALE

1.1 Background

Neuroblastoma and Relapse Studies

Neuroblastoma is the most common extracranial solid tumour in childhood and the principal cause of death due to cancer in infancy. It is also, after domestic accident, the second most frequent cause of mortality in children. More than 1200 cases/year are diagnosed in USA and Europe (Maris et al. 2007; Maris 2010; Gatta et al. 2009). Half of those cases are considered high-risk disease (metastatic/*MYCN* amplified) (Maris et al. 2007). With the use of intensive chemotherapy, surgery, myeloablative chemotherapy with haematopoietic stem cell rescue, radiotherapy and differentiating therapy with 13-cis-retinoic acid, long-term survival for children with high-risk neuroblastoma has moderately improved over the past 30 years, but in long-term reports, overall survival is still below 50% (Pearson et al. 2008; Matthay et al. 2009). The recent introduction of immunotherapy into the multimodal treatment of neuroblastoma has shown promising results with improvements in 2-year event free survival (EFS) of up to 20% after the addition of the anti-GD2 monoclonal antibody ch14.18 with interleukin-2 and GM-CSF, although it remains to be established the long-term benefit of this modality where late relapses have been described (Yu et al. 2010; Simon et al. 2011).

Up to 60% of children with high risk neuroblastoma will experience relapse with current therapies. In metastatic neuroblastoma, 10-year OS was 2% after relapse and 1.5% after progression according to Italian Registry data (Garaventa et al. 2009). In INRG, a database with outcomes from 8800 children with neuroblastoma treated worldwide, 5-year overall survival (OS) after relapse was 8% for non-infants with relapsed metastatic neuroblastoma and 4% for those with MYCN amplification (London et al. 2011). There is an unmet need to develop new therapeutic strategies and test new agents in children with neuroblastoma.

In addition to relapsed neuroblastoma, there are some patients that remain refractory to current front-line conventional chemotherapy and also require novel therapies. Since the International Neuroblastoma Response Criteria (INRC) were established in the late 80's and reviewed in the 90's (Brodeur et al. 1988; Brodeur et al. 1993) very few clinical trials have reported the outcome of patients according to response to front-line treatment. The long-term report of the German NB97 and NB90 trials described that 5-year event-free survival was 54.9% for those patients achieving complete responses (CR), 47% for those achieving CR or very good partial responses (VGPR) and 37% for those in partial response (PR) after induction (Simon et al. 2011). Moreover, several reports assessing metastatic response using MIBG scintigraphy scans have shown an unfavourable prognostic impact of poor response in MIBG scans after induction chemotherapy and several semi-quantitative scores have been described to assess MIBG scans (Yanik et al. 2010; Lewington, Bar Sever, and Lynch 2009; Ady et al. 1995; Matthay, Edeline, et al. 2003). Using the Curie score after induction therapy in patients enrolled on Children's Oncology Group (COG) A3973 clinical trial at frontline, Yanik et al. described how 3-year EFS was 8.3% for those with a score >5 vs. 41.5% for those with a score ≤5 and this difference was more striking in those cases of MYCN amplified disease (Yanik et al. 2010).

In Europe, up to 28 countries follow the front-line treatment in the SIOPEN HR-NBL1 study which has demonstrated in two randomised questions the benefit of the addition of G-CSF to avoid infections during induction chemotherapy and the benefit of busulfan-melphalan conditioning over carboplatin-etoposide-melphalan for myeloablative chemotherapy with stem cell support (Ladenstein et al. 2008; Ladenstein et al. 2011). Up to 30% of the patients have had suboptimal responses to induction chemotherapy and second-line regimen Topotecan Vincristine and Doxorubicin (TVD) requiring further chemotherapy (Ladenstein et al. 2010). In this trial, 3-year EFS of 49% has been reported for those

receiving busulfan-melphalan, showing that still, more than 50% of children with high-risk neuroblastoma experience relapse.

A number of second-line strategies have been tested in children with refractory or relapsed neuroblastoma over the past thirty years including chemotherapy, immunotherapy or targeted radionucleide therapy. Most published clinical trials have concentrated on response as an endpoint and there is a lack of data on median survival. The interpretation of those studies remains very difficult given that the populations studied were heterogeneous, with different proportions of refractory/relapsed patients, numbers of prior treatments, different response criteria being used, prognostic factors were not considered and most studies were single-arm non-randomised studies The majority of these studies have only reported best responses during treatment, without data on median time-to-progression, PFS or OS; endpoints that might be more reflective of patient benefit. Overall, objective response rates (CR + VGPR + PR) seem to range from 10 to 25%, and 25 to 50% experience disease stabilisation (SD). Overall, patients with refractory disease seem to benefit more than those with relapsed disease. Some of those regimens have been taken forward to front-line, for example topotecan-cyclophosphamide is now used at induction by Children's Oncology Group following the results of a non-randomised pilot study (London et al. 2010) and topotecan-vincristine-doxorubicin is now used in the frontline HR-NBL1 trial by SIOPEN.

Table 1 - Second line chemotherapy regimens tested in phase II in relapsed or refractory neuroblastoma since 2000

Regimen (reference)	Collaborative Group		Responses (Number of patients)	Response Rate (%)		Comments
				CR +PR	SD	
Temozolomide (Rubie et al. 2006)	UKCCSG/SFOP		5/25 CR, VGPR, PR<br>7/25 SD/NR<br>3/25 MR	20%	40% (SD/NR/MR)	
Irinotecan (Vassal et al. 2008)	SFOP/UKCCSG		0/37 CR, PR<br>5/37 SD	0%	13%	
Temozolomide/ Irinotecan (Kushner et al. 2006; Bagatell et al. 2011)	MSKCC		3/39 CR,PR,<br>5/39 SD	7.7%	12.8%	
	COG		8/55 CR, PR<br>29/55 SD	15%	53%	
Topotecan/ Temozolomide, phase II (Di Giannatale et al. 2012)	ITCC		9/38 CR, PR<br>21/38 MR/SD	24%	55%	1-year PFS 45%
Topotecan/ Vincristine/ Doxorubicin (Garaventa et al. 2003)	SIOPEN		16/25 CR, PR<br>4/25 SD	64%	16%	already in HR-NBL1
Topotecan/ cyclophosphamide vs. topotecan alone (London et al. 2010)	COG	TOPO /CYCL	24/87 CR, PR<br>15/87 MR	27.5%	17% (MR)	improved PFS (p=0.029)
		TOPO	17/89 CR, PR<br>12/89 MR	19%	13.5% (MR)	

Topotecan/ etoposide (Simon, Langler, Berthold, et al. 2007)	GPOH	17/36 (CR, PR)<br>SD n/a	47%	n/a	
Topotecan/ cyclophosphamide/ etoposide (Simon, Langler, Harnischmacher, et al. 2007)	GPOH	19/31 (CR, VGPR, PR)<br>1/31 SD	61%	3%	

Results of an ITCC (Innovative Therapies for Children with Cancer) single arm phase II trial of the combination of topotecan and temozolomide (TOTEM) have been recently become available. In this study, efficacy was achieved if more than 10/36 objective responses (complete + partial responses) after 2 cycles were seen. In the study, 7/38 responses were seen after 2 cycles (18%), but there were 9 complete and partial responses in 38 patients recruited (24%) during all treatment, with a median duration of response of 8.5 months. In addition, overall tumour control rate (responders plus stable disease) was 79%. Finally, 12-month progression free survival was 45%. The toxicity profile was acceptable. (Di Giannatale et al. 2012).

Taken together, these results (similar response rate, prolonged disease stabilisation and lower incidence of febrile neutropaenia [and hence hospital admissions] and non-haematological toxicity than temozolomide and irinotecan) warrant evaluation of temozolomide + topotecan in a randomised phase II trial.

Table 1 summarizes efficacy data from published phase II studies in relapsed or refractory neuroblastoma evaluating temozolomide, irinotecan, topotecan or combinations and Table 2 reports most common grade 3-4 toxicities in these mentioned trials.

Table 2: Comparison of toxicity of regimens in relapsed or refractory neuroblastoma

**Regimen**	**Gr 3-4 Haematological toxicities (%)**			**Gr 3-4 Non-haematological toxicities (%)**	
	Thrombocytopaenia	Neutropaenia	Anaemia	Febrile neutropaenia	Other
Temozolomide (SFOP/UKCCSG)	16	12	9	8	
Irinotecan-temozolomide (COG)	13	34	15	22	Nausea + vomiting 11<br>Diarrhoea 5
Topotecan-temozolomide (ITCC)	71	89	58	8	ALT/AST elevation 11

With the introduction of molecularly targeted agents into paediatric oncology, a number of small molecules and monoclonal antibodies targeting cell signalling pathways are now undergoing, and completing phase I testing and it is envisioned that the addition of those agents to second-line regimens will improve outcome for this poor prognosis population. It is crucial then, to identify the best backbone chemotherapy regimen to combine with novel agents within the next decade.

Angiogenesis in neuroblastoma

Neuroblastoma is a highly vascular tumour (Canete et al. 2000; Rossler et al. 2008). It has been shown that a high level of expression of angiogenesis factors VEGF (Vascular Endothelial Growth Factor) A and B is associated with poor prognosis (Rossler et al. 2008; Canete et al. 2000; Jakovljevic et al. 2009).

Strong preclinical evidence suggests that angiogenesis inhibition with several Receptor Tyrosine Kinase Inhibitors (RTKI) or monoclonal antibodies produces anti-tumour responses *in vitro* and *in vivo* (Rossler et al. 2008; Maris et al. 2008; Segerstrom et al. 2006; Sims et al. 2008).

Bevacizumab improved delivery and efficacy of chemotherapy in neuroblastoma models (Dickson et al. 2007) and treatment with bevacizumab alone (Segerstrom et al. 2006) or combined with topotecan decreased tumour growth in neuroblastoma xenografts (Dickson et al. 2007). The combination of bevacizumab with topotecan showed enhanced preclinical activity (Kim et al. 2002). Bevacizumab also improved the delivery and efficacy of chemotherapy in neuroblastoma xenografts (Dickson et al. 2007; Kim et al. 2002; Zhen et al. 2010; Patterson et al. 2011b).

Moreover, pre-clinical testing of other agents such as VEGF receptor small molecule tyrosine kinase inhibitors vandetanib (Beaudry et al. 2008), cediranib (Maris et al. 2008) or axitinib (Rossler et al. 2011) showed promising results producing an antiangiogenic effect (downregulation of VEGFR2, decreased microvessel density) with significant tumour growth delay. Other molecularly targeted agents may also exert anti-tumour activity via inhibition of angiogenesis such as MDM2 inhibitors or PI3K/AKT/mTOR inhibitors (Patterson et al. 2011a; Chanthery et al. 2012; Roche). Table 3 summarises the available data on preclinical testing of angiogenesis inhibitors in neuroblastoma.

Table 3 Preclinical studies using VEGF inhibitors in neuroblastoma

Agent (reference)	Results
Bevacizumab (Sims et al. 2008; Dickson et al. 2007)	Prolonged survival of mice with neuroblastoma xenografts and decreased tumour burden<br>Bevacizumab improved the delivery of chemotherapy
Bevacizumab + topotecan (Dickson et al. 2007)	Synergistic effect in neuroblastoma xenografts when topotecan was administered after bevacizumab
Vandetanib (Beaudry et al. 2008)	Tumour growth inhibition in neuroblastoma xenografts after vandetanib, while no anti-tumour effect seen with bevacizumab alone
Axitinib (Rossler et al. 2011)	Tumour growth delay in neuroblastoma xenografts
Cediranib (Maris et al. 2008)	Paediatric preclinical testing programme (PPTP), tumour growth delay in 5/6 neuroblastoma xenografts

Bevacizumab

Bevacizumab is a recombinant humanised monoclonal antibody against VEGF that blocks the binding of VEGF to its receptors. It has been used in a large number of adult patients during extensive phase III investigations and post-marketing authorisation (see bevacizumab Summary of Product Characteristics [SPC] (Roche)) as single agent, or in combination, with chemotherapy or radiotherapy. Studies in adults have established the safety profile with manageable toxicity as a single agent and in combination

including experience with irinotecan in high grade glioma. In a small proportion of patients the toxicity has been severe (hypertension, gastrointestinal perforation, thrombosis, haemorrhage, and proteinuria).

Adverse events of special interest (AESIs) have been defined as adverse drug reactions that have been observed across clinical trials in which bevacizumab has been used either as monotherapy or in combination with chemotherapy. These are:-

- Hypertension
- Proteinuria
- Wound healing complication
- Bleeding/haemorrhage
- Thromboembolic events (arterial and venous)
- Fistula
- Gastrointestinal perforation
- Congestive heart failure (CHF)
- Posterior Reversible Encephalopathy Syndrome (PRES)

Despite early concerns about cerebral bleeding with bevacizumab therapy, relatively few reports of this complication have emerged in brain tumour patients receiving bevacizumab even though central nervous system (CNS) haemorrhage can occur spontaneously in patients with malignant glioma. Amongst the 167-patient Phase II study of bevacizumab with or without irinotecan, 2 patients (2.4%) who received single-agent bevacizumab experienced Grade 1 intracranial haemorrhage, and 3 patients (3.8%) who received bevacizumab plus irinotecan experienced Grades 1, 2, and 4 intracranial haemorrhage (Gururangan et al. 2010).

An increase in survival for several types of adult cancers (colorectal, breast, ovarian or non-small cell lung cancer) (Roche) have been demonstrated.

Bevacizumab in children

A phase I single agent evaluation of bevacizumab (Glade Bender et al. 2008) and a phase II study in combination with irinotecan for high grade and diffuse pontine gliomas (Gururangan et al. 2010) have been completed in paediatrics while there are currently more than ten trials using bevacizumab in combination in children with solid tumours. To date, frequent toxicities reported in children have been infusion reactions, proteinuria, rash, hypertension or haematological toxicity. Severe grade 3-4 toxicities have been rare, consisting of central nervous system (CNS) events (ischaemia/haemorrhage), hypertension, proteinuria, haematological toxicity and fatigue:

COG conducted a Phase I trial (Study AVF2771s) of bevacizumab in children with refractory extracranial solid tumours. The primary aims included determining the maximum-tolerated dose (MTD) or recommended Phase II (RP2D) dose through use of a restricted dose escalation scheme based on clinically efficacious doses in adults, defining dose limiting toxicities (DLTs) and other toxicities and describing bevacizumab pharmacokinetics (PK) in children. Secondary aims included assessment of bevacizumab anti-tumour activity and exploration of potential biomarkers of anti-angiogenesis (Glade Bender et al. 2008).

Overall, bevacizumab therapy was well tolerated in these paediatric patients and had an acceptable toxicity profile when administered at doses of 5, 10, or 15 mg/kg every 2 weeks. Eighteen of the 21 patients were fully assessable (i.e., completed one course) for toxicity. A total of 67 courses were administered (median, 3 courses per patient; range, 1−16 courses). Although no DLTs were encountered, the following non−dose-limiting, (Grade 1−2 toxicities) with a possible, probable, or definite relationship to protocol therapy were observed in more than 10% of the patients assessable for toxicity: infusional reaction (n = 3), rash (n = 3), mucositis (n = 2), proteinuria (n = 3), thrombocytopenia (n = 2), or leukopenia (n = 6). None of these non-DLTs required the discontinuation of therapy. A statistically significant increase in systolic and diastolic blood pressure (BP), not meeting Common Terminology

Criteria for Adverse Events version 3 (CTCAE v3) paediatric-specific criteria for hypertension, were observed in the majority of children (11 of 16 with complete documentation, with a median increase of 6 mmHg for systolic and 9 mmHg for diastolic pressures) irrespective of dose level. Rare but serious AEs (SAEs) seen in adults, including haemorrhage and arterial thromboembolism, were not observed. Bevacizumab was not thought to have contributed to the cause of death in the 14 patients who died during the study.

Of the 52 currently available case reports covering all paediatric age subsets, bevacizumab was prescribed mostly to patients with CNS malignancies; therefore, not unexpectedly, most of the reported SAEs belong to the neurology category of the CTCAE v3 (cerebral ischemia, cerebrovascular accident, intracranial haemorrhage, convulsion, hemiparesis/hemiplegia, and headache). Since the reported clinical conditions are commonly encountered in patients undergoing treatment for CNS tumours, it is understandable that the investigators were not able to determine whether these SAEs represented adverse drug reactions. A very small number of children with relapsed neuroblastoma treated with bevacizumab have been reported: among two cases in the COG phase I study (Glade Bender et al. 2008) and two cases treated on compassionate use in Austria (Benesch et al. 2008), one patient in the latter report experienced a prolonged partial response.

Paediatric experience with other VEGF-targeted therapies is extremely limited. Only vandetanib, pazopanib and cediranib have completed phase I investigation in children (Broniscer et al. 2010; Fox et al. 2010; Glade Bender et al. 2013) with pazopanib being currently explored in the phase 2 setting.

During the conduct of the BEACON-Neuroblastoma trial, several phase 2 trials using bevacizumab in paediatric cancers have been reported in sarcoma, high grade glioma and osteosarcoma (Chisholm et al. 2017; Grill et al. 2018; Navid et al. 2017). A phase 2 single centre-single arm study performed at MSKCC (Memorial Sloan Kettering Cancer Centre) in New York and has studied the combination of bevacizumab with irinotecan and temozolomide chemotherapy. This study allowed prior therapy with irinotecan temozolomide. Three complete responses were reported among 33 patients with median progression-free survival of 7.7±1.7 months. 25/33 patients had received irinotecan+ temozolomide before (Modak et al. 2017). Another study led by NANT (New Agents for Neuroblastoma Therapy) consortium is a single arm study of bevacizumab in combination with metronomic chemotherapy (oral cyclophosphamide and zoledronic acid) and is currently ongoing with no results available. Neither of these is randomised. The BEACON-Neuroblastoma trial differs markedly from the MSKCC study for the following reasons: i) it is a randomised study, ii) patients with prior exposure to irinotecan or temozolomide are excluded, iii) a different schedule of irinotecan-temozolomide is used, and iv) it has embedded biomarker studies that will assess relevant biological hypotheses to elucidate the role of antiangiogenic therapy in neuroblastoma.

Paediatric pharmacology

In the above-mentioned COG phase I study, AVF2771s (Glade Bender et al. 2008), bevacizumab was administered as a 30−90 minute infusion on Days 1 and 15 of a 28-day course, with no interruptions between courses. On the basis of adult data, the starting dose was 5 mg/kg, with cohort escalations to 10 and 15 mg/kg. The final dose level was expanded to include at least 3 children younger than 6 years of age. Of the 21 patients enrolled, 20 (10 males, 10 females; median age, 13 years; range, 1–21 years) were assessable at dose levels of 5 mg/kg (n = 3), 10 mg/kg (n = 3), and 15 mg/kg (n = 14). Because serum concentration-time data fitted a one-compartment model in 3 patients and a two-compartment open model in 5 patients, data were analysed by non-compartmental methods to provide comparable PK estimates for all patients. The serum exposure to bevacizumab, as measured by area under the concentration-time curve (AUC), appeared to increase in proportion to dose. The median clearance was 4.1 mL/d/kg (range, 3.1−15.5 mL/d/kg), and the median t½ was 11.8 days (range, 4.4−14.6 days). Although there is some evidence of a sex difference in bevacizumab PK for adult patients, the patient numbers precluded the investigators from performing a similar analysis in children.

With a limited duration of sampling, non-compartmental analysis is more likely to reflect the transition-phase half-life (from the initial predominate distribution phase into the predominant elimination phase) than the elimination-phase half-life. For this reason, a population-modelling analysis (PMA) was performed, and the resultant estimated bevacizumab PK parameters were as follows: the median clearance was 3.25 mL/day/kg (range, 1.58–10.5 mL/day/kg), and the median t½, 22.0 days (range, 10.0–41.8 days) (Clinical Study Report AVF2771s).

Results of Bevacizumab and Irinotecan Randomisation.

Entry to the irinotecan and bevacizumab randomisations was completed in July 2018 and February 2019 respectively. The initial results on objective (complete or partial) responseand toxicity were reported at ASCO 2019 (irinotecan)(Moreno et al. 2019) and ESMO 2019 (bevacizumab)(Moreno L 2019).

In brief, for the irinotecan randomisation, 121 patients were randomised. Ten patients randomised to irinotecan (17%) and 14 patients randomised to no irinotecan (24%) responded. The risk ratio for response was= 0.70, with 95% credible interval 0.32 to 1.44. The probability that the risk ratio for responsewas >1.0 was 17%, meaning that irinotecan + temozolomide did not show greater activity than temozolomide. Patients randomised to irinotecan experienced more grade 3-4 diarrhoea (12 vs. 0 grade 3-4 AEs) and other GI toxicities (17 vs. 3 grade 3-4 AEs) than those randomised to no irinotecan.

For the bevacizumab randomisation, 160 patients were randomised. Twenty one patients (27%) randomised to bevacizumab and thirteen patients (17%) randomised to no bevacizumab responded. The risk ratio for response was 1.62 [one-sided 80% CI = 1.24-∞]. The success criterion for bevacizumab for response was met. Patients randomised to bevacizumab experienced more grade 3-4 myelotoxicity: anaemia (20 vs. 8 episodes), neutropaenia (31 vs 24) and thrombocytopaenia (27 vs. 21) and GI toxicities (25 vs. 14 episodes). There were 18 episodes of proteinuria , 5 of them were grade 3-4 (all cases-all grades of proteinuria occurred in bev patients).

The data on PFS and OS for the irinotecan and bevacizumab randomisations require longer follow-up before the definitive analyses are performed. Analyses available in January 2020 triggered the Urgent Safety Measure that is discussed in section 1.2.3.

1.1.1 Background for the dinutuximab beta amendment

GD2 is a ganglioside which is an excellent target in neuroblastoma. It is almost universally expressed on neuroblastoma cells, but has a relatively limited distribution (neurons, skin melanocytes and peripheral pain fibres) on normal tissues (Yang and Sondel 2010). After the ANBL0032 study by the US Children's Oncology Group was reported, immunotherapy with the anti-GD2 monoclonal antibody dinutuximab together with GM-CSF and interleukin-2 (IL-2) was introduced as consolidation treatment after myeloablative chemotherapy in high risk neuroblastoma (Yu et al. 2010). Dinutuximab (Unituxin, United Therapeutics) has FDA approval, but is not available in Europe.

Dinutuximab beta (Quarziba, Eusa Pharma) is closely related to dinutuximab. The two antibodies have identical amino acid sequences, but are produced in different cell lines. This results in differing glycosylation patterns, which confer some differences in biological properties (Zeng et al. 2005). Therefore, although the antibodies have similar specificity for GD2, they should be considered as distinct agents. Dinutuximab beta received marketing authorisation from the EMA in 2017 ('Dinutuximab beta Apeiron Assessment report' 2017). Dinutuximab beta has been most widely used in Europe, in the context of the trials conducted by the European Neuroblastoma Research Network (SIOPEN) where it has been given as consolidation therapy, with and without adjuvant IL-2. In patients with relapsed or refractory neuroblastoma, a SIOPEN phase II single arm trial of dinutuximab beta given as a long-term infusion with IL-2 as consolidation therapy showed a 40% objective clinical response rate and a 2-year overall survival of 64% (Lode NH 2016).

Historically controlled analyses conducted for both frontline and relapsed/refractory patient populations in SIOPEN trials support the possibility of benefit with the addition of dinutuximab beta to maintenance therapy.

Chemo-immunotherapy: There is emerging evidence of the benefit of the combination of anti-GD2 therapy with chemotherapy (so called "chemo-immunotherapy").

Preclinical evaluation of the combination of dinutuximab with chemotherapy has shown that neutrophils have significant anti-tumor effects against neuroblastomas in vitro but only in presence of dinutuximab. Moreover, chemotherapy in combination with dinutuximab and GM-CSF achieved significant activity in vitro and in vivo, doubling median survival time and led to PFS in a high-tumor burden metastatic xenogeneic neuroblastoma model (Yeo and Asgharzadeh, Advances in Neuroblastoma Research 2018)

In the COG ANBL1221 trial, patients were randomised to receive either irinotecan-temozolomide-temsirolimus (an mTOR inhibitor) or irinotecan-temozolomide-dinutuximab-GM-CSF, to ascertain whether either combination met the minimum defined clinical activity to warrant further investigation. Mody et al. (Mody et al. 2017) reported a 53% response rate (5 complete responses and 4 partial responses; 95% CI 29–77) in 17 patients treated with dinutuximab plus GM-CSF in combination with chemotherapy at first relapse or with refractory disease. One-year PFS was 77% (95% CI: 56-97) and overall survival 88% (95% CI: 72-100). An update on this trial was presented at ASCO in 2018, where results in an expansion cohort of 53 patients receiving dinutuximab plus GM-CSF with irinotecan and temozolomide, were reported. In this group, there were 21 objective responses (40%), with 11 (21%) complete responses (Mody R 2018).

Another anti-GD2 antibody – hu14.18K322A – combined with induction chemotherapy potentially shows benefit when used upfront in newly diagnosed patients; in a single-institution non-randomised study conducted at St. Jude, a response rate of 76% was seen, higher than the 40% seen in historical controls (Furman WL 2016), (Federico et al. 2017).

Despite this promising clinical activity, the mechanism of action of the apparent benefits of giving anti-GD2 monoclonal antibody and chemotherapy are not clear, and published pre-clinical studies are limited. In vitro testing indicates that chemotherapy may sensitise neuroblastoma cells to anti-GD2 mediated apoptosis (Kowalczyk et al. 2009). Combining monoclonal antibodies which target tumour antigens with chemotherapy is widely used in other tumours (e.g. rituximab, herceptin) (Hiddemann et al. 2005), (Slamon DJ 2001). Furthermore, the broader benefits of chemotherapy on the tumour immune environment and the potential for combining conventional agents with immunotherapy are being increasingly recognised (Emens and Middleton 2015). To date, no clinical trial has tested dinutuximab beta in combination with chemotherapy. In this study, chemo-immunotherapy with dinutuximab beta will be evaluated for the first time in a randomised trial. Also, this trial is the first using a long-term infusion schedule in combination with chemotherapy to reduce toxicities associated with shorter-term infusions.

For chemo-immunotherapy, the key study by Mody et al. (Mody et al. 2017) showed no unacceptable toxicities aside from one case of grade 4 hypoxia in a patient with rapidly progressive thoracic disease. The most common grade 3 or worse adverse events were pain (seen in 44% of patients), hypokalaemia (38%), neutropenia (25%), thrombocytopenia (25%), anaemia (25%), fever and infection (25%), and hypoxia (25%). The St. Jude study (Federico et al. 2017) showed good tolerability of hu14.18K322A in combination with chemotherapy in the upfront setting. No dose-reductions for the monoclonal antibody were required, although the infusion time was extended in 47% of patients following development of a cough, hypoxia and/or hypotension.

A list of clinical studies of anti-GD2 therapies in combination is given in Appendix 13.

1.2 Trial Rational

1.2.1 Justification for design

There is a lack of randomised clinical trials in neuroblastoma: there has been only one large randomised study in frontline therapy addressing the role of induction regimens (Pearson et al. 2008), and few addressing other components of frontline therapy such as high dose chemotherapy (Pritchard et al. 2005; Matthay et al. 1999; Berthold et al. 2005), immunotherapy (Yu et al. 2010) or 13-cis-retinoic acid (Matthay et al. 1999; Yu et al. 2010).

More importantly, in the relapsed setting where new therapies are tested prior to being explored in phase III trials of frontline treatment, there have been few randomised phase II studies published (London et al. 2010). This lack of reliable evidence leads to the widespread use of agents that may not be effective (e.g. irinotecan) or may add unnecessary toxicity.

A randomised Phase II design is needed to ensure that patients allocated to the experimental treatments are similar to those in the control group, thereby avoiding problems of interpretation to which previous non-randomised studies in this disease area have been subjected. Randomisation controls for selection factors and will allow an unbiased estimate of the differences between arms at the end of the trial.

Currently there is no standard chemotherapy for children with relapsed/refractory neuroblastoma after first line treatment and both irinotecan-temozolomide and temozolomide alone have been used. The use of topotecan has been investigated in phase II studies previously but not in direct comparison with temozolomide. The factorial design used in this trial will allow the benefit of a new agent (bevacizumab) to be evaluated as well as evidence on the role of irinotecan and topotecan. The factorial design will also mean fewer patients will be enrolled in one single trial than would be required for multiple different trials. A Phase II trial is needed to obtain initial evidence of activity before proceeding, if appropriate, to a Phase III trial that will evaluate efficacy.

The best backbone chemotherapy regimen will be used to combine with new molecularly targeted agents. Considering the large number of potential molecularly targeted agents, novel clinical trial designs are required to test agents efficiently.

Following a review of the Phase II data for the bevacizumab randomisation (referred to henceforth as Bevacizumab part 1) by the independent Data Monitoring Committee and the Trial Steering Committee TSC), the Trial Management Group (TMG) decided that this randomisation should continue to 160 patients. This extension continues as a Phase II trial (referred to henceforth as Bevacizumab part 2), but with PFS as the primary outcome measure. The long-term outcome PFS – along with OS – is a more relevant measure than short-term response to patients and parents. However, at the start of BEACON, there was insufficient information on PFS in relapsed/refractory neuroblastoma to be able to do a sample size calculation, so response was used as the primary outcome (although it was not known whether response is a valid surrogate for long-term outcome). Data from BEACON is now available with which to perform such a calculation, so PFS can be used as the primary outcome in Bevacizumab part 2.

1.2.2 Rationale for patient population

The trial will include the representative age group that suffers from relapsed/refractory neuroblastoma: children and young people aged 1 to 21 years old that have failed prior treatment. Performance status and bone marrow function cut-off values are those representative for the patient population where bone marrow involvement is frequent and are consistent with other phase II studies performed within COG or Europe. Metastases in the central nervous system (CNS) can occur in children with neuroblastoma particularly in the relapsed setting in up to 5% of patients (Matthay, Brisse, et al. 2003). Bevacizumab has been extensively employed in the treatment of adults and also in children with brain tumours such as high grade glioma or medulloblastoma (Gururangan et al. 2010; Friedman et al. 2009). Besides, a number of clinical trials are currently evaluating bevacizumab in several paediatric brain tumours. For this reason, children with CNS metastases will be allowed to participate in the BEACON-Neuroblastoma study provided there are no clinical signs or radiological features of bleeding lesions in the CNS. A brain CT/MRI scan will be required at screening in order to detect and monitor any CNS metastases. Patients with bleeding metastases will be excluded.

1.2.3 Benefit-risk assessment

There is wide experience of managing toxicity for all the agents being tested in BEACON-Neuroblastoma and this will minimise the risks to patients. Given that severe toxicity can occur with the use of bevacizumab in adult and paediatric patients, significant effort has been made to put in place guidance to be used in the event of toxicity.

The protocol includes dose adjustments, reductions and delays where relevant. Patients with pre-existing organ toxicity or significant bone marrow failure will not be allowed in the study. Robust standard operating procedures will be implemented for pharmacovigilance across this multi-centre international trial.

There is insufficient information on the long-term side effects of all components of BEACON-Neuroblastoma, especially for bevacizumab which has been used in fewer paediatric patients, although when used in poor-risk populations no concerns have been raised to date (Benesch et al. 2008; Glade Bender et al. 2008; Gururangan et al. 2010).

A Data Monitoring Committee (DMC) will monitor toxicity at regular intervals in order to minimise exposure to toxic treatments.
The initial results of the bevacizumab and irinotecan randomisations for PFS and OS in the BEACON-Neuroblastoma trial were reviewed and discussed by the TMG.In brief, the results suggest that, considering just the primary comparisons (irinotecan vs. not, and bevacizumab vs. not), irinotecan is effective and bevacizumab just meets the Phase II success criterion in terms of PFS However, there is some, but not conclusive, evidence of a potential positive interaction between irinotecan and bevacizumab, which would mean that patients receiving the three drug arm (bevacizumab, irinotecan and temozolomide) derive more benefit than those on either bevacizumab + temozolomide or irinotecan + temozolomide. This interaction is statistically uncertain and it is unlikely that longer follow-up or further analyses will help clarify this interpretation, though analysis of the topotecan-containing arms may.
While the interpretation of these results is complex, it is clear that the temozolomide only arm is inferior in terms of PFS and OS.

The TMG agreed that these results do have an impact on patient safety (in terms of inferior survival) in the ongoing randomisation with dinutuximab beta and, hence, considered that future patients entered into the trial should not be allocated to temozolomide chemotherapy alone. These results were discussed with the independent DMC and the TSC. As a result, the TMG decided to close the temozolomide chemotherapy arms (temozolomide and dinutuximab beta with temozolomide) in the current randomisation with immediate effect via the notification of an Urgent Safety Measure on 28 January 2020 to the UK Competent Authority (MHRA) and Ethics Committee. The randomisation will continue as temozolomide and topotecan vs. dinutuximab beta, temozolomide and topotecan, under the same conditions as before (2:1 randomisation ratio favouring chemo-immunotherapy and cross-over for patients whoprogress on temozolomide and topotecan).

The TMG also recommended that trial patients currently receiving temozolomide or dinutuximab beta, and temozolomide at the point of implementation and their patients should be approached to dicsuss the available results. Further treatment at the investigators discretion however the TMG supported the option of switching to a temozolomide and topotecan based regimen.

1.2.4 Rationale for the selected backbone schedules: Temozolomide, irinotecan + temozolomide and temozolomide + topotecan

Temozolomide and irinotecan + temozolomide are the combinations selected for evaluation as they have shown the most promising results with tolerable side effects and are widely used internationally. The irinotecan + temozolomide combination is widely considered a “standard” treatment for relapsed or refractory neuroblastoma, yet there is no good evidence that this regimen is any better than single agent temozolomide: two studies of irinotecan + temozolomide have reported response rates of 8% and 15%, while a single study of temozolomide has reported a response rate of 20%. Hence, there is no justification for the use of irinotecan + temozolomide as the standard backbone in this trial and a randomisation between irinotecan + temozolomide and temozolomide has been incorporated in order to obtain unbiased evidence on the role of irinotecan.

Temozolomide + topotecan has been shown in a recent non-randomised study (TOTEM) (Di Giannatale et al. 2012) to result in positive response rates of 24%, clinical benefit ratio of 79% and 1-year progression free survival of 42% and a favourable toxicity profile. Topotecan has been added to this randomised study to obtain to provide supporting data for the use of topotecan in the treatment of neuroblastoma in children.

The dose of temozolomide as single agent for paediatric solid tumours has consistently followed the Stupp regimen with a recommended schedule of 200 mg/m^2 daily orally for five days every 28 days (Rubie et al. 2006). Different schedules of irinotecan + temozolomide have been used in neuroblastoma and other tumour types. When combined with irinotecan, it has been shown that the tolerated dose of temozolomide for this heavily pre-treated population is 100 mg/m^2 every three weeks (i.e. equivalent to 133 mg/m^2 every four weeks) (Bagatell et al. 2009). Studies using higher doses of temozolomide have found excessive toxicity that led to delays in the administration of chemotherapy (Kushner et al. 2006). Although irinotecan was initially used at lower and more protracted doses (10 mg/m^2 per dose, five days a week for two weeks every three weeks) (Bagatell et al. 2009), there is evidence in children with rhabdomyosarcoma that giving higher doses over one week, every three weeks, does not impact on toxicity or efficacy, but is less burdensome for the patients and their families (Mascarenhas et al. 2010). Therefore, the proposed arms, temozolomide and irinotecan-temozolomide, utilise the most internationally accepted schedules.

In June 2018, the irinotecan randomisation (temozolomide vs. irinotecan + temozolomide) was closed having reached its target recruitment of 120 patients. The topotecan randomisation (temozolomide vs. temozolomide + topotecan) will continue through the dinutuximab beta amendment.

1.2.5 Rationale for dosing schedule of bevacizumab

Given the difficulties of a four-arm study testing two backbone chemotherapy regimens, one given four-weekly (temozolomide) and the other given three-weekly, the PK data and previous experience in adult studies suggested that a 5 mg/kg/week schedule of bevacizumab would achieve therapeutic levels without significant differences from 10 mg/kg/biweekly for those receiving 4-weekly chemotherapy vs. 15 mg/kg/3-weekly for those receiving 3-weekly chemotherapy.

1.2.6 Rationale for evaluating chemo-immunotherapy in the BEACON-Neuroblastoma Trial

The key elements supporting the clinical testing of novel targeted therapies in paediatric cancers, particularly neuroblastoma, have been described in two European consensus publications by the Innovative Therapies for Children with Cancer consortium (ITCC) [Moreno EODD 2017, Moreno Nat Rev Clin Onc 2017]. In this case, the combination of chemotherapy with anti-GD2 therapy with dinutuximab beta, all preclinical and clinical conditions required for its phase 2 evaluation in relapsed & refractory neuroblastoma are present, as described next.

- Less than 8% of children with relapsed high risk neuroblastoma achieve long term survival (London J Clin Oncol 2011). There is a great unmet need for new therapies in this field and a lack of therapeutic options.

- GD2 is an excellent therapeutic target in neuroblastoma: it is present in most neuroblastoma cells and absent in most human tissues, and there are clinically available monoclonal antibodies targeting GD2.
- There are multiple examples of successful combination of monoclonal antibodies with chemotherapy showing enhanced results, for example rituximab (anti-CD20) in combination with chemotherapy in mature B-Non Hodgkin Lymphomas (e.g. Meinhardt A. 2010 Phase II Window Study on Rituximab in Newly Diagnosed Pediatric Mature B-Cell Non-Hodgkin's Lymphoma and Burkitt Leukemia.).
- Preclinical evaluation of the combination of dinutuximab with chemotherapy has shown that neutrophils have significant anti-tumor effects against neuroblastomas in vitro but only in presence of dinutuximab. Moreover, chemotherapy in combination with dinutuximab and GM-CSF achieved significant activity in vitro and in vivo, doubling median survival time and led to progression-free survival in a high-tumor burden metastatic xenogeneic neuroblastoma model (Yeo and Asgharzadeh, ANR 2018)
- Early clinical trials conducted in the US with two different anti-GD2 monoclonal antibodies (with dinutuximab and hu14.18K322A) in combination with chemotherapy, also early experience with dinutuximab beta in Germany have shown unprecedented positive results

This, taken together with the poor outcome and high unmet need for novel therapies in this patient group, form a robust rationale for this clinical trial proposal.

- NCT01576692. Hu14.18K322A in combination with topotecan/cyclophosphamide, irinotecan/temozolomide or ifosfamide/carboplatin/etoposide in relapsed & refractory patients, n=13. Response rate 61.5%, 1-year PFS 77% (St. Jude pilot trial).
- NCT01857934. Hu14.18K322A in combination with topotecan/cyclophosphamide, cyclophosphamide/doxorubicin/vincristine or cisplatin/etoposide in children as induction for newly diagnosed high-risk neuroblastoma, n=20 Response rate 80%. (St.Jude Pilot trial)
- NCT01767194. Dinutuximab plus GM-CSF with irinotecan-temozolomide N=34 (17 patients treated with dinutuximab). Median progression-free survival was 0.25 years for arm A [irinotecan-temozolomide-temsirolimus] compared to 2.14 years for arm B, [irinotecan-temozolomide-dinutuximab], (US Children's Oncology Group randomised phase 2 trial)
- Compassionate use program. Dinutuximab beta plus induction chemotherapy in relapsed & refractory high- risk neuroblastoma, n=16, best objective response rate 50% (German pilot study).

Details of the studies are provided in Appendix 13.

1.2.7 Rationale for dosing schedule of dinutuximab beta

Continuous infusion:

The SIOPEN HR-NBL-1 trial initially included delivery of dinutuximab beta as a short (5 x 8 hour) infusion regimen (in a similar manner to the published COG trial of dinutuximab (Yu et al. 2010), whilst the current SIOPEN Phase II LTI study and the subsequent amendment of HR-NBL1 trial (so-called R4 randomisation) has delivered the same dose of the antibody as a continuous 10 day (240 hour) infusion. The latter schedule has resulted in improved tolerability, with a reduction in Grade 3 and 4 toxicities, reduced pain scores as well as reduced IV morphine use and favourable pharmacokinetics (Ladenstein R 2015). Dosing of 10mg/m^2/day as a continuous infusion over 10 days in 5-week cycles was found to be tolerable without intravenous opiates in the majority of patients. This schedule can be tolerated by many patients in an outpatient setting, delivered via an ambulatory pump, particularly when it is given without IL2. On the basis of the current evidence, continuous infusion, at this rate, is considered by the SIOPEN group to be the standard infusion schedule of dinutuximab beta. This method of continuous infusion will therefore be used for this current study.

Dose of Dinutuximab beta:

In the Mody et al. (Mody et al. 2017) chemo-immunotherapy study combining dinutuximab and irinotecan + temozolomide, a lower dose of dinutuximab was used compared to when it had been given with cytokines in children with minimal residual disease (17.5 mg/m^2 per day for 4 days, compared to 25 mg/m^2 for 4 days). Similarly, the Furman et al. (Furman WL 2016) study, combining Hu14.18k322A, chemotherapy used cytokines, used a lower dose of antibody than the recommended phase II dose (40mg/m^2 per day for 4 days compared to recommended dose of 60mg/m^2/day for 4 days. For this study, in view of the fact that continuous infusion of dinutuximab beta is well tolerated at 10mg/m^2/day, we propose to use the same daily dose, but reduce the number of infusion days compared to how it has been previously given (7 days total compared to 10 days). This should achieve good pharmacokinetics of the antibody during the period of chemotherapy exposure (Siebert, Eger, et al. 2016) but will largely avoid delivering the antibody during the period of significant neutropenia. It will also minimize the amount of time patients need to stay in hospital. Total antibody delivery per cycle will be 70% of 'standard', non-chemo-immunotherapy dose, compared to 60% in the Mody and Furman studies. However, cycles will be given every 28 days, together with the chemotherapy courses, as compared to every 35 days for schedules when the antibody is given alone/with cytokines in the maintenance setting.

Timing of dinutuximab beta:

In the Furman and Mody studies, the anti-GD2 antibodies (dinutuximab and hu14.18K322A respectively) were given concurrently with the chemotherapy. Therefore, in this trial, dinutuximab beta administration will also start on day 1 of the chemotherapy cycles, given that temozolomide and temozolomide + topotecan are commonly used in the outpatient setting and are usually well tolerated.

Inclusion of cytokines:

In both the Mody and Furman studies, cytokines (GM-CSF in both studies, plus IL-2 in the St. Jude one) were given in addition to the anti-GD2 antibody and chemotherapy. All patients received the cytokines so it is not clear whether they contributed to efficacy. The SIOPEN HR-NBL-1 and LTI studies have both shown that the addition of IL-2 contributes significantly to toxicity, but clear evidence in terms of efficacy has not been demonstrated. Therefore, in view of the fact that cytokines would increase the risk of toxicity, without any clear evidence supporting the addition of any of them, IL-2 is not included in the current protocol.

Anti-drug antibodies:

A number of patients in the study will have received anti-GD2 antibody previously and may have developed an anti-drug antibody (ADA) response. The incidence of neutralising levels of ADA (with the potential to reduce circulating dinutuximab beta levels) following dinutuximab beta is reported to be 7.5% (Siebert, Eger, et al. 2016). Samples will therefore be collected from patients to assess for development of ADA, but this will not be used as an exclusion criterion and a result will not be required prior to randomisation.

1.2.8 Rationale for the use of biomarker studies

Biomarkers have been defined as biological characteristics that can be objectively measured and evaluated as an indicator of normal biological, pathological processes, or pharmacological responses to a therapeutic intervention (Naylor 2003).

Three main types of biomarkers have been defined (Sawyers 2008):

- Prognostic biomarkers, give information on prognostic features helping to distinguish between cases with good or poor outcome, independent of treatment.
- Predictive biomarkers assess the probability that a patient will benefit from a particular treatment.
- Pharmacological/Pharmacodynamic (PD) biomarkers, measure effects of the drug on the tumour. PD biomarkers are of major value in making "go-no-go" decisions and assess performance of new drugs. PK biomarkers can also be included in this category.

The use of biomarkers will accelerate and improve drug development (Tan et al. 2009; Yap et al. 2010; Garrido-Laguna, Hidalgo, and Kurzrock 2011). In this study, a comprehensive biomarker evaluation has been developed taking into account all considerations for research in the paediatric population. Sampling times and blood volumes have been minimised as well as invasive investigations. Samples and images will only be collected when required as per standard of care for the patients, and volumes of blood will comply with guidance by the European Medicines Agency (EMA 2008).

Different pharmacological and predictive biomarkers relevant to the neuroblastoma population and to the use of angiogenesis inhibitors will be piloted in children.

Molecular monitoring mRNA– Detection of neuroblastoma target mRNAs in blood and bone marrow will be studied for the first time in the relapsed/refractory setting. This assay has been evaluated frontline as a prognostic indicator and to monitor minimal residual disease (MRD) and is currently being validated in HR-NBL-1/SIOPEN (Viprey et al. 2007; Burchill et al. 2001).

MRI-derived functional imaging biomarkers of angiogenesis – This study will also implement functional imaging to assess angiogenesis inhibition for extracranial malignancies in a multi-centre setting for the first time in Europe. Dynamic contrast-enhanced MRI (DCE MRI) has already been used in adult studies of angiogenesis inhibitors and therefore will be explored in this trial in a selected number of centres (Rosen and Schnall 2007; Liu et al. 2005).

Exploratory biomarker assays performed whole blood, plasma and tumour samples -Samples will be taken at study entry, during treatment and at end of treatment. These may include, but are not limited to:

- Tumour molecular profiling- gene expression and DNA sequencing will be performed to explore possible associations between candidate molecular predictors and clinical outcome.
- Mutant circulating DNA- mutant circulating DNA for driver mutations will be assessed as a potential surrogate marker for tumour burden or treatment efficacy.
- Exploratory biomarker analyses on blood samples will include identifying potential markers of prognosis or response to new therapies, and the biology of neuroblastoma.
- Pharmacokinetics of dinutuximab beta. With the evaluation of dinutuximab beta in combination with chemotherapy, using an adaptation of the long-term infusion regimen (as explained in the rationale for the dosing schedule of dinutuximab beta section), it is necessary to conduct a limited evaluation of basic pharmacokinetic parameters of dinutuximab beta when given in combination with chemotherapy.

Exploratory biomarker assays related to immunotherapy with dinutuximab beta:

- Fcgamma and KIR receptor genotype have been described as potential biomarkers of response to antiGD2 antibodies in the maintenance setting (Erbe et al. 2018). In this study, their role as predictors of response to chemo-immunotherapy will be evaluated.
- ADA. Antidrug antibodies have been described in different proportions in the different antiGD2 antibodies developed. The presence and development of ADA during treatment with chemo-immunotherapy in this trial will be collected retrospectively, but without clinical implications.
- ADCC (antibody dependent cell-mediated cytotoxicity) has also been shown in studies using antiGD2 antibodies in the maintenance setting as a potential pharmacodynamics and predictive biomarker, and it will be collected here in the chemo-immunotherapy setting.

1.3 Relevance and future importance

The proposed study is part of the SIOPEN European collaborative strategy for the development of new agents for neuroblastoma. Other themes of research within this strategy include immunotherapy (a

Phase I/II of long-term infusion anti-GD2 immunotherapy) and radionuclide therapy (LUDO, a phase II study of Lutetium Dotatate for refractory neuroblastoma). Those studies are complementary and aim to evaluate different components of the current multimodal treatment for high risk neuroblastoma. Additionally, strong links with COG mean that the study results will be shared with the relevant North American phase II studies.

Furthermore, the results of the current frontline phase III trial High Risk Neuroblastoma Study 1 of SIOP – Europe HRNBL-1/SIOPEN will soon be available. The results of BEACON-Neuroblastoma will feed into the European frontline therapy for the upcoming SIOPEN phase III trial where the most promising combinations will be tested in larger patient groups.

2. OBJECTIVES AND OUTCOME MEASURES

2.1 Objectives

Primary:

- To test whether bevacizumab added to a backbone chemotherapy regimen (temozolomide, irinotecan + temozolomide or temozolomide + topotecan) demonstrates activity in children with relapsed or refractory neuroblastoma
- To test whether the addition of irinotecan to temozolomide increases the activity of chemotherapy in children with relapsed or refractory neuroblastoma
- To test whether the addition of topotecan to temozolomide increases the activity of chemotherapy in children with relapsed or refractory neuroblastoma (“topotecan randomisation”)
- To test whether dinutuximab beta added to a backbone chemotherapy regimen (temozolomide or temozolomide + topotecan) demonstrates activity in children with relapsed or refractory neuroblastoma (“dinutuximab beta randomisation)

Secondary:

- To evaluate the safety of the regimens

Tertiary:

- To undertake preliminary evaluation of the changes in magnetic resonance imaging (MRI) derived functional imaging biomarkers of angiogenesis
- To undertake preliminary evaluation of the role of circulating mRNA levels for tyrosine hydroxylase (TH), paired-like homeobox 2b (PHOX2B) and doublecortin (DCX) as prognostic/predictive biomarkers in this refractory/relapsed setting
- To undertake a preliminary evaluation of the role of tumour molecular profiles in blood and archival tumour tissue including pharmacogenomic profiles as prognostic and predictive biomarkers in children with neuroblastoma
- To undertake a preliminary evaluation of biomarkers of response to anti-GD2 therapy (Fc/KIR polymorphisms, ADCC, and ADAs) and of dinutuximab beta pharmacokinetics (PK)
- Pilot descriptive study of neuroblastoma markers that may include: O6-methylguanine-methyltransferase (MGMT) status, immunohistochemistry and immunofluorescence markers on tumour samples (such as microvessel density (MVD), CD31, Ki67, NRP1, VEGFR-1, VEGFR-2, C-KIT), DNA/RNA extraction from tissue sections for tumour mutation screening and tumour expression profiling
- A preliminary correlation of the different biomarkers [Fc/KIR polymorphisms, Antibody – Dependent Cellular Toxicity (ADCC), and Anti-Drug Antibodies (ADAs)] will be made with parameters of anti-tumour activity (response rate, PFS and OS). PK parameters (dinutuximab beta trough levels) for this chemo-immunotherapy regimen will be described

2.2 Outcome Measures

Primary Endpoint:

- Best response (Complete Response [CR], or Partial Response [PR](Brodeur et al. 1988) at any time during the first 6 cycles of trial treatment
- For the bevacizumab part 2 only: Progression-free survival (PFS)

Secondary Endpoints:

- Safety of the regimens: Incidence and severity of Adverse Events (AE)
- PFS
- Event-free survival (EFS)
- Overall survival (OS)

Exploratory/Tertiary Endpoints:

- Changes in magnetic resonance imaging (MRI) derived functional imaging biomarkers of angiogenesis measured by quantitative dynamic contrast enhanced (DCE) MRI: primary biomarkers will be the transfer constant K^{trans} [min^{-1}] and initial area under the gadolinium uptake curve from 0 to 60 seconds ($IAUGC_{60}$, mM Gd min) and secondary biomarkers will be tumour apparent diffusion coefficient (ADC, 10^{-6} cm^2 s^{-1}), native T1 and T2 relaxation times (ms) and transverse relaxation rate R^{2*}
- Changes in circulating mRNA levels for TH, PHOX2B and DCX in bone marrow and blood samples

3. TRIAL DESIGN

This is an international open-label, randomised, multicentre phase II trial of temozolomide ± irinotecan or temozolomide ± topotecan, with or without bevacizumab or dinutuximab beta, for the treatment of patients with relapsed or refractory neuroblastoma. The study will evaluate the safety and activity of these combinations.

3.1 Randomisation

Patients will be registered into the trial and randomised at the same time to one of the following six arms (hereinafter referred to as the “bevacizumab randomisation”):

T: Temozolomide
BT: Bevacizumab + Temozolomide (closed 07-Feb-2019)
IT: Irinotecan + Temozolomide (closed in June 2018)
BIT: Bevacizumab + Irinotecan + Temozolomide (closed in June 2018)
TTo: Temozolomide + Topotecan
BTTo: Bevacizumab + Temozolomide + Topotecan (closed 07-Feb-2019)

The bevacizumab randomisation closed to recruitment on 07 February 2019.

Following completion of the bevacizumab randomisation (approximately 160 patients), 64 additional patients will be registered into the trial and randomised at the same time to one of the following four arms (hereinafter referred to as the “dinutuximab beta randomisations”):

T: Temozolomide
dBT: Dinutuximab beta + Temozolomide
TTo: Temozolomide + Topotecan
dBTTo: Dinutuximab beta + Temozolomide + Topotecan

Following review and discussion by the TMG of the PFS and OS results of the bevacizumab and irinotecan randomisations the evidence shows that T alone is inferior. Therefore, from 28 January 2020, patients will be randomised to one of the following two arms:

TTo: Temozolomide + Topotecan

dBTTo: Dinutuximab beta + Temozolomide + Topotecan

Randomisation will be via a secure on-line computer-based system at the CRCTU, University of Birmingham, UK and patients will be allocated in a 2:1 ratio favouring dinutuximab beta. Minimisation will be used to ensure balance across the arms for the important prognostic factors as described by London et al. (London et al. 2011): a) refractory disease, early relapse (<18 months), late relapse (≥18 months) and b) measurable versus evaluable disease (i.e. disease evaluated according to RECIST versus disease detectable only by MIBG scanning with or without bone marrow involvement as detected by local morphology). The number of patients within each country is expected to be small; therefore it was considered not practical to stratify the randomisation by country.

3.2 Duration of treatment

Patients will receive treatment for 6 courses, lasting 24 weeks depending on the arm of the trial that they are randomised to.

Patients with a response (CR, PR) or stable disease (SD) while on the BEACON-Neuroblastoma trial will receive 6 cycles of trial treatment. If the patient has achieved a satisfactory response (i.e. CR, PR or SD) with acceptable toxicity, treatment may be extended beyond 6 cycles (up to 12 cycles) after discussion with the Coordinating Sponsor and the CI.
Patients randomised to receive dinutuximab beta who continue past 6 cycles will receive a chemotherapy only (without dinutuximab beta) treatment regimen beyond cycle 6.

3.3 Frequency and duration of follow-up

A follow-up visit should be scheduled 90 ± 15 days after the last study drug administration. In case of sustained adverse events (AEs), additional follow up study visits will be scheduled according to the severity of the AEs. Frequency of study visits and length of follow-up will need to be discussed with the Coordinating Sponsor and the CI. New onset AEs occurring within 90 days from end of trial treatment will be recorded and followed-up after discussion with the Coordinating Sponsor.

For patients who experience relapse or receive further treatment, after the follow-up visit, the local physician will enter the survival and relapse status of the patient on the Follow Up Form at follow up time points until death, for at least 5 years after registration.

Patients who do not relapse or start further treatment will have 3-monthly (±15 days) follow-up visits for the first year after discontinuation of study therapy and then 6-monthly (±15 days) for at least 5 years after registration.

4. ELIGIBILITY

Please refer to Section 9.1 for eligibility criteria for the bevacizumab randomisation and to Section 10.1 for eligibility criteria for the dinutuximab beta randomisation.

4.1 Lifestyle guidelines

Patients with reproductive potential must agree to use an adequate method of birth control during the period of therapy, i.e. with a failure rate of less than 1% per year. Men should be advised not to father a child up to 6 months after receiving the last dose. Women of childbearing potential should be advised to use effective contraception to avoid pregnancy up to 6 months after the last dose of study treatment. Effective contraceptive methods include implants, injectables combined oral contraceptives, intrauterine device (IUD or coil), sexual abstinence or vasectomised partner.

5. SCHEDULE OF ACTIVITIES

Please refer to Section 9.2 for the schedule of activities for the bevacizumab randomisation and to Section 10.2 for the schedule of activities for the dinutuximab beta and topotecan randomisation.

6. SCREENING AND CONSENT

6.1 Informed Consent

It is the responsibility of the Principal Investigator or co-investigator (if the responsibility has been delegated by the Principal Investigator as captured on the Site Signature and Delegation Log) to obtain written informed consent for each patient before performing any trial related procedure. A Patient/Parent Information Sheet (PIS) is provided to facilitate this process. Investigators must ensure that they adequately explain the objectives of the trial, trial treatment and potential benefits and hazards to the patient of taking part in the trial. It must be clearly explained to the patient/parents that participation in the biomarker studies (tumour profiling, exploratory biomarkers, molecular monitoring and DCE-MRI) is mandatory given the scientific relevance of the biomarker studies, with the exception of the blood DNA sample which is optional. The blood volumes will comply with EMA guidance for research in children. The time points for the biomarker studies will not require additional visits to hospital. No additional procedures under sedation/general anaesthesia will be performed. The Investigator should also stress that the patient, parents or legal guardian are free to refuse to take part and can withdraw from the trial at any time. The patient, parents or legal guardian should be given ample time, (e.g. usually at least overnight), to read the PIS and to discuss their participation with others outside of the site research team. The patient/parent must be given an opportunity to ask questions which should be answered to their satisfaction. The right of the patient and parents or legal guardian to refuse to participate in the trial without giving a reason must be respected.

If the patient, parent or legal guardian expresses an interest in their/child participating in the trial they should be asked to sign and date one copy of the latest approved version of the Informed Consent Form. The Investigator or designate must then sign and date the form on the same day as the patient/parent/legal guardian. Written assent will also be obtained from patients under the age of 16 years wherever it is possible to do so using the relevant section on the Informed Consent Form. For those children who are not able to read, write or understand the Informed Consent Form where it is deemed appropriate for them to provide assent, the clinician will explain the study and obtain verbal assent where possible and this will be documented in the medical notes. A copy of the Informed Consent Form should be given to the parent or patient (if over 16 years), a copy should be filed in the medical notes, and the original(s) placed in the Investigator Site File (ISF). Once the patient has been entered into the trial, the patient's trial number should be entered on the Informed Consent Form maintained in the ISF. In addition, if the patient/their parents has/have given explicit consent and permissible by local laws, a copy of the signed Informed Consent Form must be sent to the National Co-Sponsor's Trial Office for review.

Details of the informed consent discussions should be recorded in the patient's medical notes, this should include date of, and information regarding, the initial discussion, the date consent was given, with the name of the trial and the version number of the Patient Information Sheet and Informed Consent Form. Throughout the trial the patient and parent or legal guardian should have the opportunity to ask questions about the trial and any new information that may be relevant to the patient's continued participation should be shared with them in a timely manner. On occasion it may be necessary to re-consent the patient, for example if new information becomes available or an amendment is made to the protocol that might affect the patient's participation in the trial. In this case the process above should be followed and the patient's and parents or legal guardian's right to withdraw from the trial respected.

Electronic copies of the Patient Information Sheets and Informed Consent Forms are available from the Trial Office and should be printed or photocopied onto the headed paper of the local institution. Details of all patients approached about the trial should be recorded on the Patient Screening/Enrolment Log.

With the patients/parents prior consent their General Practitioner (GP)/Primary Physician should also be informed that they are taking part in the trial. A GP Letter is provided electronically for this purpose.

6.2 Screening

No trial specific procedure should be carried out prior to signing the consent form for this study, (see section 6.1). All patients who have been consented as part of screening must undergo the following assessments/procedures 28 days prior to randomisation and as indicated below:

- Imaging of measurable or evaluable disease (MRI (preferred) or CT including the brain, MIBG scan (or ^{18}-FDG PET/CT scans if MIBG negative disease) and bilateral bone marrow aspirates and trephines assessed by local morphology) must have been performed within 4 weeks prior receiving the first dose of trial treatment. Screening tumour assessment should be recorded on the Tumour Assessment CRF, recording measurable disease as per RECIST 1.1 (target and non-target lesions), mIBG (or PET) according to the SIOPEN score and bone marrow assessment (report of bilateral aspirates and trephines). For those patients having MRI scanning, images will also be obtained for MRI-derived functional imaging biomarkers of angiogenesis
- Echocardiogram should be performed within 4 weeks of randomisation. If the patient has had an echocardiogram within 12 weeks of eligibility assessment and has not received anthracyclines or cardiotoxics then this echocardiogram can be used for screening and does not need to be repeated for this study.
- Complete medical history (including past medical history, concurrent medical events and dates of all previous anticancer treatment) within 4 weeks prior to dosing
- Full clinical examination (including vital signs, weight and standing height) and performance status at screening and within 24 hours prior to receiving the first dose of trial treatment. Sitting height will also be measured at screening. Where possible, a stadiometer should be used to measure height. Where this may be difficult in particularly young children, standard measurement methods may be used. Menstrual status (regularity of menstruation) in females of child bearing potential should be assessed at screening, at post Cycle 6 (or at end of treatment if the patient discontinues early), and during follow up. Tanner staging should be performed within 4 weeks prior to receiving the first dose of trial treatment and during follow up
- Haematology [includes Haemoglobin (Hb), white blood cells (WBC), neutrophil count, lymphocytes and platelets] at screening and within 72 hours prior to dosing. If more than 72 hours elapse from screening to initiation of trial therapy, they should be repeated
- Biochemistry (includes sodium, potassium, calcium, urea, creatinine, total protein, albumin, bilirubin, alkaline phosphatase (ALK PHOS), GGT, ALT or AST must be done at screening and within 72 hours prior to dosing
- A calculated GFR (radioisotope or 24 hour urine calculated creatinine clearance) must be carried out in patients with serum creatinine $\geq$ 1.5 ULN for age within 7 days of randomisation
- A urine (preferred) or serum pregnancy test will be done on girls who are post-menarche within 72 hours prior to cycle 1 dosing. Results of the tests are needed to determine patient eligibility and testing may need to be repeated so that result from the pregnancy test are available for within 72 hours before cycle 1 dosing
- Bilateral bone marrow samples at screening for the molecular monitoring analysis of mRNA RT-qPCR (TH, PHOX2B, DCX) **(Please refer to most recent version of Laboratory Manual before taking samples)**
- A blood sample for the molecular monitoring analysis of mRNA RT-qPCR (TH, PHOX2B, DCX) taken at the same time as the bone marrow examination
- A whole blood sample taken for DNA analysis at baseline (optional to patient/parent)
- A blood sample for exploratory biomarkers should be collected before study drug administration
- Available archival tumour sample to be identified prior to starting study medications. If a sample is not available, the local physician must agree the potential study entry with the Coordinating Sponsor and the CI always **before** starting trial medication. The archival tumour sample will be

shipped on a regular basis as arranged with the Sponsor. Please see Laboratory Manual for further details

- Any ongoing adverse events from previous treatment and concomitant medications must be documented and continued to be assessed throughout the study. Records of ongoing concomitant medication will not be collected on the CRF, but should be available for reporting when an SAE occurs

Separate screening tests for the different randomisations
For the bevacizumab randomisation only:

- Clotting (including INR and APTT) at screening and within 72 hours prior to dosing
- Proteinuria test (Early morning urine dipstick and/or protein/creatinine ratio) at screening and within 24 hours of dosing. If there are signs of proteinuria (>2+), a sample must be sent for determination of albumin/protein and creatinine in urine and the albumin or protein/creatinine ratio or 24 hour urinary protein excretion must be calculated. This will also be repeated within 24 hours prior to each bevacizumab injection or the start of cycle 1
- An X-ray of the left wrist/hand. The Greulich-Pyle atlas should be used to assess bone age

For the dinutuximab beta randomisation only:

- Oxygen saturation, visual acuity and Chest X Ray (CXR) are required at screening

7. TRIAL ENTRY

7.1 Procedure for online patient randomisation

Randomisation for the trial should be performed by sites using the online electronic data capture system. Informed consent should be obtained prior to any trial-related procedures.

Once a paper Eligibility Checklist has been completed, in order to randomise a patient, the online Randomisation Form must be completed. Randomisation of patients can be achieved by logging on to:
https://www.cancertrials.bham.ac.uk/BEACONLive

This program will confirm eligibility and allocate treatment via a computerised minimisation algorithm, developed by the CRCTU.

A copy of the Randomisation Confirmation Report and the patient's trial number (TNO) should be printed and retained in the Investigator Site File (ISF) and patient's notes. The TNO should be written on the Informed Consent Form filed in the ISF and used on all serious adverse event (SAE) forms and correspondence relating to that patient.

In addition where possible a copy of the patient's Informed Consent Form must be sent in the post to the National Co-Sponsor's Trial Office.

7.2 Emergency Randomisation

In case of any problems with online randomisation, a paper Eligibility Checklist and paper Randomisation Form should be completed. These details can be phoned through to the BEACON-Neuroblastoma Trial Office at the Cancer Research UK Clinical Trials Unit (CRCTU), University of Birmingham, UK, or to the dedicated Randomisation line using the number below:

RANDOMISATION
(09:00 to 17:00 GMT, Monday to Friday)
☎ +44 (0) 121 414 3366

8. TREATMENT DETAILS

8.1 Definition of Investigational Medicinal Products (IMPs)

The following drugs are IMPs in this trial:

Table 4: IMPs in the BEACON-Neuroblastoma Trial

IMP	Formulation
Bevacizumab	Infusion
Cyclophosphamide	Infusion
Dinutuximab beta	Infusion
Irinotecan	Infusion
Temozolomide	Capsule
Topotecan	Infusion

9. BEVACIZUMAB RANDOMISATION

9.1 Eligibility criteria for the bevacizumab randomisation

9.1.1 Inclusion criteria for the bevacizumab randomisation

Disease specific

- Histologically proven neuroblastoma as per International Neuroblastoma Staging System (INSS) (Brodeur et al. 1988) definition
- Relapsed or refractory neuroblastoma
  - Relapsed: any relapsed or progressed high-risk neuroblastoma
  - Refractory high risk disease: Lack of adequate response to frontline therapy that precludes the patient from proceeding to consolidation therapies (e.g. myeloablative chemotherapy)
- Measurable disease by cross sectional imaging (RECIST) or evaluable disease (uptake on MIBG scan with or without bone marrow histology). Patients with only bone marrow detectable disease (bone marrow aspirate or trephine) are NOT eligible for the study

General

- Age ≥1 to ≤21 years
- Informed consent from patient, parent or guardian

Performance and organ function

- Performance Status:
  - Lansky ≥ 50%, Karnofsky ≥ 50% or ECOG ≤3
  - (Patients who are unable to walk because of paralysis, but who are able to sit upright unassisted in a wheelchair, will be considered ambulatory for the purpose of assessing performance score)
- Life expectancy of ≥12 weeks
- Bone marrow function (prior to 72 hours of planed randomisation date):
  - No bone marrow disease:
    - Platelets ≥ 75 x 109/L (unsupported for 72 hours)
    - ANC ≥ 0.75 x 109/L (no G-CSF support for 72 hours)
    - Haemoglobin > 7.5 g/dL (transfusions allowed)
  - Bone marrow disease:
    - Platelets ≥ 50 x109/L (unsupported for 72 hours)
    - ANC ≥0.5 x 109/L (no G-CSF for 72 hours)
    - Haemoglobin > 7.5 g/dL (transfusions allowed)
  - Renal function (prior to 7 days of randomisation date):

    - Absence of clinically significant proteinuria (early morning urine dipstick ≤2+). When the dipstick urinalysis shows a proteinuria > 2+, a protein: creatinine (Pr/Cr) ratio must be < 0.5 or a 24 hour protein excretion must be < 0.5g
    - Serum creatinine ≤1.5 ULN for age, if higher, a calculated GFR (radioisotope or 24 hour urine calculated creatinine clearance) must be ≥ 60 ml/min/1.73 m2
- Liver function (prior to 72 hours of randomisation date): AST or ALT ≤2.5 ULN and total bilirubin ≤1.5 ULN. In case of liver metastases, AST or ALT ≤5 ULN and total bilirubin ≤2.5 ULN
- Cardiac function, measured using echocardiogram prior to 4 weeks of randomisation date or 12 weeks if patient has not received anthracyclines or cardiotoxics. Shortening fraction ≥29% on echocardiogram
- Coagulation, patients not on anticoagulation must have an INR ≤1.5 and APTT ≤1.5 ULN for age. Anticoagulation is permitted as long as the INR or APTT is within therapeutic limits (according to the medical standard of the institution) and the patient has been on a stable dose of anticoagulants for at least two weeks at the time of study enrolment.
- Blood pressure below 95th centile for age and sex. Use of antihypertensive medication is permitted
- Males or females of reproductive potential may not participate unless they agree to use an adequate method of birth control, i.e. with a failure rate of less than 1% per year, (e.g. implants, injectables, combined oral contraceptives, IUDs, sexual abstinence or vasectomised partner), for the duration of study therapy and for up to 6 months after the last dose of trial drugs. A negative urine (preferred) or serum pregnancy test must be obtained within 72 hours prior to dosing in females who are post-menarche.

9.1.2 Exclusion criteria for the bevacizumab randomisation

- Previous treatment with bevacizumab, temozolomide, irinotecan or any combination of these drugs
- Known hypersensitivity to:
    - Any study drug or component of the formulation
    - Chinese hamster ovary products or other recombinant human or humanised antibodies
    - Dacarbazine
- Prior severe arterial thrombo-embolic events (e.g. cardiac ischemia, cerebral vascular accident, peripheral arterial thrombosis)
- Any ongoing arterial thrombo-embolic events
- Patient less than (at point of planned date of randomisation):
    - 48 hours post bone marrow aspirate/trephine
    - 48 hours post central line insertion
    - Four weeks post major surgery
    - One week post core biopsy
    - Two weeks from prior chemotherapy
    - Six weeks from prior craniospinal radiotherapy or MIBG therapy and two weeks from radiotherapy to the tumour bed
    - Eight weeks from prior myeloablative therapy with haematopoietic stem cell rescue (autologous stem cell transplant)
    - Three months from prior allogeneic stem cell transplant
    - 14 days or 5 half-lives (whichever occurs later) from last administration of an IMP in an IMP-trial
    - 6 months from presentation of lung haemorrhage/haemoptysis
- Bleeding metastases (patients with CNS metastases can be enrolled as long as the metastases are not bleeding)
- Invasion of major blood vessels
- Use of enzyme inducing anticonvulsants within 72 hours of randomisation

- History or evidence of inherited bleeding diathesis or significant coagulopathy at risk of bleeding (i.e. in the absence of therapeutic anticoagulation)
- History of abdominal fistula, gastrointestinal perforation, intra-abdominal abscess or active gastrointestinal bleeding within 6 months prior to study enrolment
- Current chronic intestinal inflammatory disease/bowel obstruction
- Intolerance to galactose and fructose, lactase deficiency, and/or defect of absorption of galactose and fructose
- Pregnant or lactating patient
- Any uncontrolled medical condition that poses an additional risk to the patient (i.e. haemoptysis, non-healing, bone fracture, wound/ulcer)
- Low probability of treatment compliance

9.2 Schedule of activities for the bevacizumab randomisation

Protocol Activity	Screen / Baseline	Pre Dosing (All Cycles)	Post Cycles 2, 4 & 9 (Up to 7 days prior to starting the next cycle)	Post Cycle 6 & End of Treatment[16] (28 days after Cycle 6 dose and Last Dose given)	Follow Up[17]
Informed Consent/Assent	X				
Medical History	X				
Physical Examination and Performance status[1]	X	X		X	X
Weight and Height[2]	X	X		X	X
Sitting Height[2]	X			X	X
**Laboratory Tests**					
Haematology[3]	X	X		X	X
Blood Chemistry[3]	X	X		X	X
Clotting[3]	X	X		X	X
Pregnancy Test[4]	X			X	
Urinalysis[5]	X	X		X	X
Glomerular Filtration Rate (GFR[6])	X				
**Tumour Assessment**					
MRI or CT Brain[7]	X				
MRI (preferred) or CT scan of tumour[7]	X		X	X	
MIBG[7] or ^{18}FDG PET/CT Scan	X		X	X	
Bilateral Bone Marrow Aspirate and Trephine[7]	X		X	X	

Biomarkers					
	**Screen / Baseline**	**Pre Dosing (All Cycles)**	**Post Cycles 2, 4 & 9 (Up to 7 days prior to starting the next cycle)**	**Post Cycle 6 & End of Treatment[16] (28 days after Cycle 6 dose and Last Dose given)**	**Follow Up[17]**
Blood for analysis of mRNA RT-qPCR (TH, PHOX2B, DCX)[8]	X		X (excluding Post Cycle 9)	X	
Bone marrow aspirate for analysis of mRNA RT-qPCR (TH, PHOX2B, DCX)[8]	X				
Blood sample for exploratory biomarkers[9]	X		X (excluding Post Cycle 9)	X	
MRI-Derived Functional Imaging Biomarkers[10]	X		X (excluding Post Cycle 9)	X	
Whole blood sample for DNA[11]	X				
Tumour Molecular Profiling (Formalin Fixed Paraffin Embedded) & Frozen tissue [12]	X				
**Miscellaneous**:					
X-Ray left wrist[13]	X			X	X
Echocardiogram[14]	X				
Tanner staging[15]	X			X	X
Menstrual Status[15]	X			X	X
Adverse Events	**X** (assess throughout study)				
Concomitant Medications	**X** (assess throughout study)				

1. Performance status will be reported using the Lansky scale for 1-12 year olds and using the Karnofsky/ECOG scale for older patients. Performance status and physical examination (including BP, HR and Temperature) will be performed at screening/baseline and within 24 hours prior to each cycle in all treatment arms. Physical examination will also be performed prior to each injection of bevacizumab in arms BIT, BT and BTTo. Both examinations will also be performed at the end of treatment visit, and at each follow up visit (see footnote 17). Menstrual status (regularity of menstruation) in females of child bearing potential should be assessed at screening, end of treatment and during follow up.
2. Patient's weight will be measured at screening, on day 1 of each cycle prior to dosing and at the end of treatment. Body height (standing or lying length where age-appropriate) will be measured at screening, on day 1 of each cycle prior to dosing and at the end of treatment. Body height (sitting length) will be measured at screening, and at the end of treatment visit. Where possible, a stadiometer should be used. Where this may be difficult in particularly young children, standard measurement methods may be used. After end of treatment, weight, sitting height and standing height will be performed at each follow up visit (see footnote 17).
3. Haematology, biochemistry and clotting blood tests must be done at screening and within 72 hours prior to cycle 1 dosing. Unless required by the investigator, safety tests will not be repeated at cycle 1, day 1 prior to dosing. Beginning in cycle 2 and continuing for cycles 3, 4, 5, and 6, pre-dose activities can be done within 72 h pre-dose. It is recommended that blood counts are monitored weekly for all patients. Additional safety assessments may be done according to institutional standard of care. These tests must also be performed at end of treatment and subsequent follow up visits. Haematology includes - haemoglobin (Hb), white blood cells (WBC) with differential count, neutrophils lymphocytes and platelets. Biochemistry includes - sodium, potassium, calcium, urea, creatinine,

total protein, albumin, bilirubin, alkaline phosphatase (ALK PHOS), GGT, LDH, ALT or AST. Clotting includes INR and APTT.

4. For girls who are post-menarchal, a urine (preferred) or serum pregnancy test will be done at screening (within 72 hours of dosing) and end of treatment. If the results are inconclusive, a repeat test must be performed using a urine sample. In addition, a pregnancy test will be done whenever one menstrual cycle is missing during treatment or a potential pregnancy is suspected. Girls who become pregnant while on study will be discontinued immediately and the outcome of the pregnancy will be followed. Pregnancy tests may be repeated during study if required by hospital regulations and/or Research Ethics Committees (RECs).
5. Urinalysis: A Urinalysis test (early morning urine dipstick and/or protein/creatinine ratio) will be performed at screening and within 24 hours prior to each bevacizumab injection and the start of each cycle of treatment. This will also be repeated at the end of treatment and at each follow up visit. If there are signs of proteinuria, a sample must be sent for determination of albumin/protein and creatinine in urine, and the albumin or protein/creatinine ratio must be calculated.
6. Calculated GFR (radioisotope or 24 hour urine calculated creatinine clearance) should be carried out at screening up to 7 days prior to eligibility assessment in patients with a level of serum creatinine $\geq$ 1.5 ULN for age
7. Tumour assessments: (Must comply with RECIST 1.1). The minimum requirement is a cross sectional image of site of measurable disease by MRI (preferred) or CT, performed within 4 weeks prior to receiving the first dose of trial treatment. A scan including the brain (either CT or MRI) must also be performed at screening to assess patient's eligibility with regard to the presence of bleeding brain metastases. At baseline, MIBG scans (or ^{18}-FDG PET/CT scan if MIBG negative disease) and bilateral bone marrow aspirates and trephine are to be done within 4 weeks prior to receiving the first dose of trial treatment. Bone marrow assessments will be done in all patients at screening and will only be repeated after 2, 4 and 6 courses if positive at screening or clinically indicated (until they become negative). Patients with negative bone marrow assessments at screening will not have repeated BM samples. To ensure comparability, the same scan, equipment, method and technique used during baseline should be consistently used throughout the study. Additional investigations of possible metastatic sites should be done upon presentation of signs and symptoms. Tumour lesions previously irradiated will be considered measurable only if progressing. Scans should be completed up to 7 days prior to cycles 3 and 5; following cycle 6; and at end of treatment if the patient discontinues treatment early. Response assessment will be performed with the same modalities used at screening (MRI/CT/MIBG/bone marrow histology as appropriate). Tumour assessments do not have to be repeated where the following cycle is delayed due to toxicity.
8. **Please refer to current BEACON-Neuroblastoma Laboratory Manual before taking samples.** Blood samples will be obtained at baseline, post Cycles 2, 4 and 6 for the molecular monitoring analysis of mRNA by qRT-PCR for TH, PHOX2B and DCX in all patients. Bone Marrow samples (right and left) for the measurement of circulating neuroblastoma mRNA should also be taken from all patients at screening.
9. Blood samples for the evaluation of exploratory biomarkers will be collected at baseline, post cycle 2, post cycle 4 and post cycle 6 (samples can be collected the same day as starting the next cycle, but have to be collected before study drug administration). Samples will be taken at EOT if the patient discontinues treatment early. Samples will be taken from all patients.
10. MRI-derived functional imaging biomarkers will only be performed in those patients that are assessed routinely with MRI. Functional imaging will only be performed in patients with i) tumour measuring at least 2cm in the minimum axial diameter and ii) tumour location not prone to motion artefacts (diaphragmatic surface, paracardiac region, adjacent to major arteries). Patients with metallic implants near the tumour or contraindications to MR imaging will not be eligible.
11. A blood sample for constitutional DNA analysis will also be performed at baseline. Samples for constitutional DNA analyses are optional.
12. Tumour molecular profiling: The evaluation of angiogenesis related biomarkers will be performed on samples of tumour tissue collected at the time of diagnosis or during frontline therapy and then the point of relapse if a biopsy/surgery is performed for clinical reasons (this sample is mandatory for study entry, if no tumour is available this will be discussed with the Coordinating Sponsor and the Chief Investigator **before** study entry). Archival tumour tissue either as paraffin embedded or frozen material will be collected.
13. Left hand x-ray, including the wrist should be done at screening, post cycle 6 or EOT if the patient discontinues treatment early; and then yearly until the patients relapses or starts further treatment for up to five years. Depending on the discretion of the investigator (i.e., bone pain) a more frequent monitoring could be done (i.e. every 6 months). Note: if baseline x-ray shows evidence of growth-plate fusion, no further left hand/wrist x-ray will be necessary. The Greulich-Pyle atlas should be used to assess bone age.
14. An echocardiogram should be performed within 4 weeks of randomisation. If the patient has had an echocardiogram within 12 weeks of eligibility assessment and has not received anthracyclines or cardiotoxics then this echocardiogram can be used for screening and does not need to be repeated for this study.
15. Tanner staging and menstrual status (if applicable) should be completed at screening within 4 weeks of commencing trial treatment (Appendix 12 – Tanner Staging). For patients undergoing follow up visits following end of treatment, Tanner staging and menstrual status should be collected annually.
16. Response assessment of the Primary Endpoint takes place Post Cycle 6. Patients completing treatment at cycle 6 will have an End of Treatment visit approximately 28 $\pm$7 days after the Cycle 6 dose. Patients may continue to receive further treatments following Cycle 6, following discussion with the Coordinating Sponsor and CI. Patients receiving cycles 7-12 will have the same clinical and laboratory assessments at each cycle and have a response assessment within 1 week of starting cycles 7 and 10. NB- The Post cycle 6 assessments may be used as the Pre

Cycle 7 tests. Patients who discontinue treatment early (prior to cycle 6) will have an End of Treatment visit approximately 28 days after the last dose given.

17. A follow up visit will be scheduled 90 ± 15 days after the last dose of trial treatment, unless the patient withdraws consent or receives another treatment. Patients that develop relapse or start further therapy will not undergo further follow up visits but will still have survival and disease status data collected for them at the time points for follow up visits. For those patients that remain relapse-free and do not start any further therapy, follow up visits will be done 3-monthly for one year and 6-monthly for up to 5 years. Additional follow up visits will be scheduled, as needed to monitor any sustained unresolved, treatment emergent adverse events.

9.3 Trial Therapy (bevacizumab randomisation)

Dosing of the trial drugs will take place every 3-4 weeks depending on the arm of the study that the patient is randomised to. Patients with a response (CR, PR) or stable disease (SD) while on the BEACON-Neuroblastoma trial will receive 6 cycles of trial treatment. If the patient has achieved a satisfactory response (i.e. CR, PR or SD) with acceptable toxicity, treatment can be extended beyond 6 cycles (up to 12 cycles) after discussion with the Coordinating Sponsor and the Chief Investigator.
The trial drugs to be given per cycle are listed according to trial arm below:

	Day 1	Day 2-5	Day 15	Day 22	Day 29
Drug schedule on **temozolomide-**based arms<br>*Treatment duration: 6 cycles = 24 weeks, response assessed every 2 cycles*					
T	Temozolomide 200 mg/m^2 po	Temozolomide 200 mg/m^2 po			Day 1 of next cycle starts
BT	Bevacizumab 10 mg/kg iv Temozolomide 200 mg/m^2 po	Temozolomide 200 mg/m^2 po	Bevacizumab 10 mg/kg iv		Day 1 of next cycle starts
Drug schedule on **irinotecan + temozolomide**-based arms<br>*Treatment duration: 6 cycles = 18 weeks, response assessed every 2 cycles*					
IT	Irinotecan 50 mg/m^2 iv Temozolomide 100 mg/m^2 po	Irinotecan 50 mg/m^2 iv Temozolomide 100 mg/m^2 po		Day 1 of next cycle starts	
BIT	Bevacizumab 15 mg/kg iv Irinotecan 50 mg/m^2 iv Temozolomide 100 mg/m^2 po	Irinotecan 50 mg/m^2 iv Temozolomide 100 mg/m^2 po		Day 1 of next cycle starts	
Drug schedule on **temozolomide + topotecan**-based arms<br>*Treatment duration: 6 cycles = 24 weeks, response assessed every 2 cycles*					
TTo	Temozolomide 150 mg/m^2 po Topotecan 0.75 mg/m^2/day iv	Temozolomide 150 mg/m^2 po Topotecan 0.75 mg/m^2/day iv			Day 1 of next cycle starts
BTTo	Bevacizumab 10 mg/kg iv Temozolomide 150 mg/m^2 po Topotecan 0.75 mg/m^2/day iv	Temozolomide 150 mg/m^2 po Topotecan 0.75 mg/m^2/day iv	Bevacizumab 10 mg/kg iv		Day 1 of next cycle starts

9.3.1 Bevacizumab randomisation trial treatment

Trial drugs will be administered to patients within this trial according to the treatment arm that the patient is allocated to:

Table 5: Treatment Schedule

For each treatment arm in the BEACON-Neuroblastoma trial, a cycle consists of:

**Arm T**		
Days 1 – 5	Temozolomide 200 mg/m^2/d PO	Every 4 weeks
**Arm BT**		
Day 1 and day 15	Bevacizumab 10mg/kg IV	Every 4 weeks
Days 1 – 5	Temozolomide 200 mg/m^2/d PO	Every 4 weeks
**Arm IT**		
Days 1 – 5	Irinotecan 50 mg/m^2/d IV	Every 3 weeks
Days 1 – 5	Temozolomide 100 mg/m^2/d PO	Every 3 weeks
**Arm BIT**		
Day 1	Bevacizumab 15 mg/kg IV	Every 3 weeks
Days 1 – 5	Irinotecan 50 mg/m^2/d IV	Every 3 weeks
Days 1 – 5	Temozolomide 100 mg/m^2/d PO	Every 3 weeks
**Arm TTo**		
Days 1 – 5	Temozolomide 150 mg/m^2/d PO	Every 4 weeks
Days 1 – 5	Topotecan 0.75 mg/m^2/d IV	Every 4 weeks
**Arm BTTo**		
Day 1 and day 15	Bevacizumab 10 mg/kg IV	Every 4 weeks
Days 1 – 5	Temozolomide 150 mg/m^2/d PO	Every 4 weeks
Days 1 – 5	Topotecan 0.75 mg/m^2/d IV	Every 4 weeks

Bevacizumab: If allocated to arm BT, BIT or BTTo the dose of bevacizumab will be 10 or 15 mg/kg given intravenously. Bevacizumab should be administered prior to chemotherapy. The total dose will be rounded to the nearest 5mg.

- Bevacizumab MUST not be administered within 48 hours of a minor surgical procedure (e.g. bone marrow exam or insertion of central venous access device [CVAD]) or within 28 days of a major surgery. In all cases, the investigator should verify that there are no wound healing complications before the administration of bevacizumab.
- In case of toxicity related to bevacizumab, the drug MUST be stopped and not dose reduced. See section 9.5.3 and 9.5.4 for criteria. In case of a prolonged dose interruption, the local physician will discuss with the Coordinating Sponsor and the medical team the appropriateness of re-starting treatment with bevacizumab, given that 4 to 6 weeks will be needed to achieve steady-state concentrations again and there is risk of subsequent toxicity related to bevacizumab. For those patients that are near completion of the treatment course it will be advisable to stop bevacizumab permanently.

Temozolomide: The dose of temozolomide will be 100, 150 or 200 mg/m^2/day orally depending on the allocated arm. Where possible, doses will be rounded to the nearest 5mg as per the table in Appendix 7 – Temozolomide Dosing.

Irinotecan: The dose of irinotecan will be 50 mg/m^2/day. Irinotecan will be given intravenously over 1 hour on days 1-5, at least one hour following the administration of temozolomide. The total administered dose of chemotherapy may be rounded up or down to the nearest dose that can be accurately measured.

Topotecan: The dose of topotecan will be 0.75 mg/m²/day. Topotecan will be given intravenously over 30mins on days 1-5, at least one hour following the administration of temozolomide. The administered dose of chemotherapy may be rounded up or down to the nearest dose that can be accurately measured.

NOTE: The toxicity profiles of Bevacizumab and temozolomide ± irinotecan/topotecan are different. They should be considered separately and any necessary treatment modification to one element of the study treatment should not cause a change or delay to the other element. This is particularly important with respect to Bevacizumab which takes 4 – 6 weeks to achieve steady state levels. Any treatment delay will require steady state levels to be re-established (See Section 9.5.3)

The patient's height and weight (BW) must be measured prior to each cycle of treatment, and BSA should be calculated using the Mosteller formula.

Mosteller Formula: $$BSA[m^2] = \sqrt{\frac{bodyweight[kg] \cdot bodyheight[cm]}{3600}}$$

Bevacizumab is prescribed per kg, temozolomide, irinotecan and topotecan are prescribed per body surface area (BSA). **The same dose should be given to the patient every cycle, using the doses calculated using the screening or Cycle 1 BW and BSA, UNLESS the calculated dose(s) have changed by ≥10% from the values obtained at screening/cycle 1.**

If a patient's BW exceeds that for the 98th centile for their age:

- Bevacizumab doses should be calculated with the weight for the 98th centile
- Irinotecan, temozolomide and topotecan doses should be calculated using the BSA obtained from the Mosteller formula, using the BW for the 98th centile and the patient's actual height.

In all cases, the dosing of overweight patients should be discussed and agreed with the Co-ordinating Sponsor before the administration of any IMP.

9.4 Treatment Schedule

The required evaluations at baseline (screening), on study, and at end of treatment are summarised in the Schedule of Assessments (Section 9.2) which should be consulted in the first instance. Other tests and/or increased frequency of examinations or clinical follow up may be needed for patient management based on the PI's clinical judgement, the results of which will be recorded on the CRF.

The on-study procedures will be different depending on the allocated arm and whether the cycle repeats every 21 or 28 days. The first day of a cycle is defined as the first day on which study treatment is administered. In the event of scheduling conflicts due to administrative reasons, dosing and study evaluations may take place on the designated day 1 (D1) ±3 days (see Schedule of Activities in the synopsis).

9.4.1 Day 1 of Cycle 1

- A physical examination (including weight, height, blood pressure, pulse and performance status) should be performed within 24 hours before dosing of the first cycle
- Patient's blood samples (haematology, biochemistry, clotting) should be drawn and reviewed up to 72 hours prior to dosing
- Urinalysis (early morning urine dipstick and/or protein/creatinine ratio) should be performed within 24 hours prior to each bevacizumab injection or prior to dosing in other arms

9.4.2 Day 1 of subsequent cycles

- A physical examination (including height, weight, blood pressure/pulse and performance status) should be performed within 24 hours before dosing

- Patient's lab tests (haematology, biochemistry, clotting) should be drawn and reviewed up to 72 hours prior to re-dosing
- Urinalysis (early morning urine dipstick and/or protein/creatinine ratio) should be performed within 24 hours prior to each bevacizumab injection or prior to dosing in other arms
- Tumour assessment scans will be performed every other cycle starting at end of cycle 2 (prior to cycle 3) as detailed in section 7.4.3. For patients who are having an MRI scan performed for tumour assessment purposes a functional imaging scan (MRI-derived functional imaging biomarkers of angiogenesis) will also be performed at this time
- Bilateral bone marrow aspirates and trephines assessed by local morphology will also be collected every other cycle starting at the end of cycle 2 (prior to cycle 3) as detailed in section 7.4.3
- Two blood samples for the molecular monitoring mRNA analyses and exploratory biomarkers should be collected before study drug administration at the end of cycle 2 and 4 (prior to starting cycle 3 and 5 respectively).

9.4.3 Post Cycle 6 (For patients continuing to Cycle 7-12)

A full assessment will be carried out Post Cycle 6 for patients continuing trial treatment to Cycles 7-12. The following will be performed before the start of the next cycle:

- A tumour scan (MRI/CT/MIBG/^{18}-FDG PET/CT) will also be performed +/- functional imaging scan and bone marrow sample, if previous involvement. If one modality shows progressive disease (MRI/CT, MIBG or bone marrow histology) it will not be necessary to perform all modalities
- Two blood samples for the molecular monitoring mRNA analyses and exploratory biomarkers should be collected before study drug administration
- An X-ray of the left hand/wrist

The following can be performed as part of the Pre-Cycle assessment for Cycle 7.

- A complete physical exam will include performance status, height (both sitting and standing), weight and blood pressure/pulse)
- All safety laboratory samples will be collected (haematology, blood biochemistry, clotting tests).
- Urinalysis (early morning urine dipstick and/or protein/creatinine ratio) should be performed
- A urine (preferred) or serum pregnancy test will be done on girls who are post-menarche

9.4.4 End of Treatment

An End of Treatment visit will take place approximately 28±7 days after the last dose of the last cycle of trial treatment or sooner if the patient develops PD or withdraws from the study. The following will be performed:

- A complete physical exam will include performance status, height (both sitting and standing), weight and blood pressure/pulse)
- All safety laboratory samples will be collected (haematology, blood biochemistry, clotting tests)
- Urinalysis (early morning urine dipstick and/or protein/creatinine ratio) should be performed
- A urine (preferred) or serum pregnancy test will be done on girls who are post-menarche
- A blood sample for exploratory biomarkers should be collected at the end of treatment and the point of disease progression
- An X-ray of the left hand/wrist
- A tumour scan (MRI/CT/MIBG/^{18}FDG PET/CT) will also be performed +/- functional imaging scan and bone marrow sample, if previous involvement. If one modality shows progressive disease (MRI/CT, MIBG or bone marrow histology) it will not be necessary to perform all modalities

9.4.5 Treatment Duration

Patients with a response (CR, PR) or stable disease (SD) while on the BEACON-Neuroblastoma Trial will receive 6 cycles of trial treatment. If the patient has achieved a satisfactory response (i.e. CR, PR

or SD) with acceptable toxicity, treatment can be extended beyond 6 cycles (up to 12 cycles) after discussion with the Coordinating Sponsor and the CI.

Patients receiving cycles 7 to 12 will have the same clinical and laboratory assessments at each cycle (physical examination, performance status, vital signs, full blood count, biochemistry, clotting and urine dipstick and/or protein/creatinine ratio). These patients will have a response assessment (CT/MRI, MIBG/^{18}-FDG PET/CT scan ± bone marrow aspirate/trephine) within one week of starting cycles 7, 10 and at end of treatment. No biomarker samples/scans are required.

9.5 Dose Modifications for the bevacizumab randomisation

Patients should be carefully monitored for toxicity. After the initial treatment cycle, dose reductions and/or administration delays/discontinuation will be decided using specific predefined rules to accommodate individual tolerance of treatment and maintain protocol dose intensity. All toxicities will be graded according to version 4.0 of the NCI CTCAE. Doses are to be adjusted based on the most severe toxicity that the patient experiences, related or possibly related to the study treatment between each cycle of treatment.

For haematological toxicities, the guiding principle is that the full doses of all drugs will be administered only if blood counts have recovered to ANC > 0.75 x 10^9/L (without G-CSF support for 72 hours) and platelet count > 75 x 10^9/L (without platelet transfusions for 72 hours) for patients without known bone marrow disease or to ANC > 0.5 x 10^9/L (without G-CSF support for 72 hours) and platelet count > 50 x 10^9/L (without platelet transfusions for 72 hours) for patients with bone marrow disease before the start of the next cycle.

For non-haematological toxicities, the guiding principle is that the full doses of all drugs will be administered only if toxicities are resolved to grade 2 or less prior to start of the cycle.

Patients should be assessed for the development of bevacizumab treatment emergent side effects prior to **each** bevacizumab administration.

Dose reductions for bevacizumab-related AEs are **NOT** allowed. Therefore, when indicated, the administration of bevacizumab should be either **delayed** or **discontinued**. A list of expected AEs can be found in the current version of the bevacizumab Summary of Product Characteristics (SPC) and Investigator Brochure (IB). The safety information in the bevacizumab IB will be updated as the toxicity profile of bevacizumab has been revised.

Although generally well tolerated, the use of bevacizumab is associated with a number of side effects that necessitate careful clinical monitoring. The most prevalent side effects include hypertension (most commonly), proteinuria, thrombotic events, both arterial and venous, and less frequently, bowel perforation and delayed wound healing.

Adverse events of special interest (AESIs) are adverse drug reactions that have been observed across clinical trials in which bevacizumab has been used either as monotherapy or in combination with chemotherapy. The following AESIs have also been specified in this protocol:
• Hypertension
• Proteinuria
• Wound healing complication
• Bleeding/haemorrhage
• Thromboembolic events (arterial and venous)
• Fistula
• Gastrointestinal perforation
• Congestive heart failure (CHF)

• Posterior Reversible Encephalopathy Syndrome (PRES)

It is hoped that such an approach will enable further characterisation of the clinical course and the management of these events in paediatric patients.

Adverse Events should be classified into whether at the physician's judgement these are likely to be related to bevacizumab toxicity or non-bevacizumab toxicity and managed accordingly following the steps in the diagram overleaf:

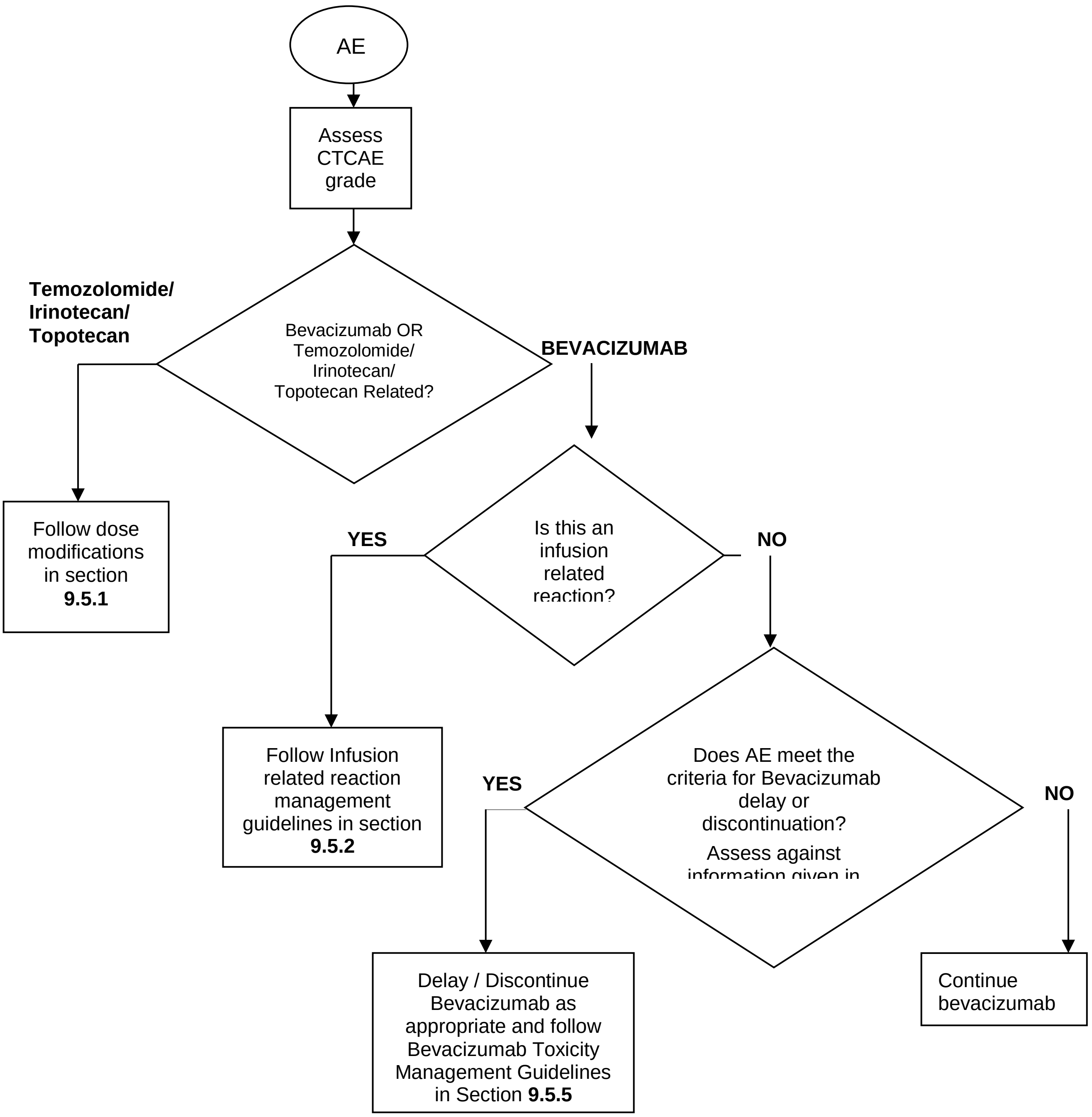

Figure 1: Classification of Adverse Events in the BEACON-Neuroblastoma Trial (bevacizumab randomisation)

9.5.1 Dose Modifications for AEs due to chemotherapy - for the bevacizumab randomisation

Note: Adverse Events should be classified according to whether, in the physician's judgement, these are likely to be related to Bevacizumab toxicity or non-Bevacizumab toxicity. These should be managed separately (Figure 1: Classification of Adverse Events in the BEACON-Neuroblastoma Trial), and any dose modification or discontinuation required to chemotherapy (temozolomide ± irinotecan or topotecan) deemed to last longer than one week should NOT affect the scheduling of Bevacizumab, even if this means that the two elements of the study treatment become unsynchronised.
In order to help synchronisation of chemotherapy and bevacizumab, if the delay is only expected for a week, all IMPs should be delayed and if the delay requires continuation longer than a week, the IMPs can be administered independently.
The maximum delay to the administration of chemotherapy due to adverse events is 43 days. The delay may be extended over 43 days only if the patient is showing a good response to the treatment and agreement is obtained from the Sponsor.

Table 6: Dose levels for dose adjustments

Drug	Starting dose	Reduction 20% Dose level - 1	Reduction 40% Dose level - 2
Arm T			
Temozolomide	200 mg/m^2/d for 5 days every 4 weeks	160 mg/m^2/d for 5 days every 4 weeks	120 mg/m^2/d for five days every 4 weeks
Arm BT			
Temozolomide	200 mg/m^2/d for 5 days every 4 weeks	160 mg/m^2/d for 5 days every 4 weeks	120 mg/m^2/d for 5 days every 4 weeks
Arm IT			
Temozolomide	100 mg/m^2/d for 5 days every 3 weeks	80 mg/m^2/d for 5 days every 3 weeks	60 mg/m^2/d for 5 days every 3 weeks
Irinotecan	50 mg/m^2/d for 5 days every 3 weeks	40 mg/m^2/d for 5 days every 3 weeks	30 mg/m^2/d for 5 days every 3 weeks
Arm BIT			
Temozolomide	100 mg/m^2/d for 5 days every 3 weeks	80 mg/m^2/d for 5 days every 3 weeks	60 mg/m^2/d for 5 days every 3 weeks
Irinotecan	50 mg/m^2/d for 5 days every 3 weeks	40 mg/m^2/d for 5 days every 3 weeks	30 mg/m^2/d for 5 days every 3 weeks
Arm TTo			
Temozolomide	150 mg/m^2/d for 5 days every 4 weeks	120 mg/m^2/d for 5 days every 4 weeks	90 mg/m^2/d for five days every 4 weeks
Topotecan	0.75 mg/m^2/d for 5 days every 4 weeks	0.5 mg/m^2/d for 5 days every 4 weeks	0.25 mg/m^2/d for 5 days every 4 weeks
Arm BTTo			
Temozolomide	150 mg/m^2/d for 5 days every 4 weeks	120 mg/m^2/d for 5 days every 4 weeks	90 mg/m^2/d for five days every 4 weeks
Topotecan	0.75 mg/m^2/d for 5 days every 4 weeks	0.5 mg/m^2/d for 5 days every 4 weeks	0.25 mg/m^2/d for 5 days every 4 weeks

Study Treatment – Dose Reduction or Discontinuation for AEs attributed to chemotherapy (NOT to bevacizumab)

Table 7 - Arm T - Dose reduction and discontinuation rules

Type of Toxicity	Dose modification at first occurrence	Dose modification at second occurrence
• ANC < 0.75 x 10^9/L or<br>• Platelet count < 75 x10^9/L<br>But recovered on day 28 after the start of a cycle	No dose modification	No dose modification
ANC < 0.75 x 10^9/L or<br>Platelet count < 75 x10^9/L<br>But recovered between day 29 to 35 after the start of a cycle	No dose modification	Decrease temozolomide to dose level -1
ANC < 0.75 x 10^9/L or<br>Platelet count < 75 x10^9/L<br>But recovered between day 36 to 42 after the start of a cycle	Decrease temozolomide to dose level -1	Decrease temozolomide to dose level –2
ANC < 0.75 x 10^9/L or<br>Platelet count < 75 x10^9/L<br>On day 43 after the start of the cycle	Consider discontinuation of study treatment*	Not applicable
Liver function: Elevation of AST/ALT grade ≥3 that recovers to grade ≤1 before day 28	No dose modification	No dose modification
Liver function: Elevation of AST/ALT grade ≥3 not recovered to grade ≤1 before day 28	Decrease temozolomide to dose level -1	Discontinue study treatment
Other grade ≥3 non haematological toxicity not recovered to grade ≤2 before day 28	Decrease temozolomide to dose level -1	Discontinue study treatment

Note: All platelets cut-off values require no platelet transfusions within 72 hours of starting the cycle. All neutrophil cut-off values require being off G-CSF for at least 72 hours. For those patients with known bone marrow involvement, the cut-off values required are ANC ≥0.5 x10^9/L and platelets ≥50 x10^9/L
*If not recovered 15 days after expected date of start (i.e. day 43 after start of cycle), refer to Sponsor to discuss individual case.

Table 8: Arm BT - Dose reduction and discontinuation rules

ype of Toxicity	Dose modification at first occurrence	Dose modification at second occurrence
ANC < 0.75 x 10^9/L or<br>Platelet count < 75 x10^9/L<br>But recovered on day 28 after the start of a cycle	No dose modification	No dose modification
ANC < 0.75 x 10^9/L or	No dose modification	Decrease temozolomide to dose level -1

Platelet count < 75 x10^9/L<br>But recovered between day 29 to 35 after the start of a cycle		
ANC < 0.75 x 10^9/L or<br>Platelet count < 75 x10^9/L<br>But recovered between day 36 to 42 after the start of a cycle	Decrease temozolomide to dose level -1	Decrease temozolomide to dose level –2
ANC < 0.75 x 10^9/L or<br>Platelet count < 75 x10^9/L<br>On day 43 after the start of the cycle	Consider discontinuation of study treatment*	Not applicable
Liver function: Elevation of AST/ALT grade ≥3 that recovers to grade ≤1 before day 28	No dose modification	No dose modification
Liver function: Elevation of AST/ALT grade ≥3 not recovered to grade ≤1 before day 28	Decrease temozolomide to dose level -1	Discontinue study treatment
Other grade ≥3 non haematological toxicity not recovered to grade ≤2 before day 28	Decrease temozolomide to dose level -1	Discontinue study treatment

Note: All platelets cut-off values require no platelet transfusions within 72 hours of starting the cycle. All neutrophil cut-off values require being off G-CSF for at least 72 hours. For those patients with known bone marrow involvement, the cut-off values required are ANC ≥0.5 x10^9/L and platelets ≥50 x10^9/L
*If not recovered 15 days after expected date of start (i.e. day 43 after start of cycle), refer to Sponsor to discuss individual case.

Table 9: Arm IT - Dose reduction and discontinuation rules

Type of Toxicity	Dose modification at first occurrence	Dose modification at second occurrence
ANC < 0.75 x10^9/L or<br>Platelet count < 75 x10^9/L<br>But recovered on day 21 after the start of a cycle	No dose modification	No dose modification
ANC < 0.75 x 10^9/L or<br>Platelet count < 75 x10^9/L<br>But recovered between day 22 to 28 after the start of a cycle	No dose modification	Decrease temozolomide to dose level -1
ANC < 0.75 x 10^9/L or<br>Platelet count < 75 x10^9/L<br>But recovered between day 29 to 35 after the start of a cycle	Decrease temozolomide to dose level -1<br>Decrease irinotecan to dose level-1	Decrease temozolomide to dose level –2<br>Decrease irinotecan to dose level -2
ANC < 0.75 x 10^9/L or<br>Platelet count < 75 x10^9/L<br>On day 36 after the start of the cycle	Consider discontinuation of study treatment*	Not applicable
Liver function: Elevation of AST/ALT grade ≥3 that recovers to grade ≤1 before day 21	No dose modification	No dose modification

Liver function: Elevation of AST/ALT grade ≥3 not recovered to grade ≤1 before day 21	Decrease temozolomide to dose level -1	Discontinue study treatment
Grade 3 and 4 diarrhoea >3 days despite maximum loperamide therapy	Decrease irinotecan dose to dose level -1<br>If the same level of toxicity persists > 2 weeks despite suitable symptomatic treatment, discontinue study treatment<br>If diarrhoea is ongoing on day 21, delay next cycle for up to 2 weeks until diarrhoea resolves to ≤ grade 1<br>If the diarrhoea does not resolve after a 2-week delay, the patient should discontinue study treatment	Decrease irinotecan dose level -2<br>If the same level of toxicity persists > 2 weeks despite suitable symptomatic treatment, discontinue study treatment<br>If diarrhoea is ongoing on day 21, delay next cycle for up to 2 weeks until diarrhoea resolves to ≤ grade 1<br>If the diarrhoea does not resolve after a 2-week delay, the patient should discontinue study treatment
Other grade ≥3 non haematological toxicity not recovered to grade ≤2 before day 21	Decrease both irinotecan and temozolomide to level -1	Discontinue study treatment

Note: All platelets cut-off values require no platelet transfusions within 72 hours of starting the cycle. All neutrophil cut-off values require being off G-CSF for at least 72 hours. For those patients with known bone marrow involvement, the cut-off values required are ANC ≥0.5 x10^9/L and platelets ≥50 x10^9/L
*If not recovered 15 days after expected date of start (i.e. day 36 after start of cycle), refer to Sponsor to discuss individual case.

Table 10: Arm BIT - Dose reduction and discontinuation rules

Type of Toxicity	Dose modification at first occurrence	Dose modification at second occurrence
ANC < 0.75 x 10^9/L or<br>Platelet count < 75 x10^9/L<br>But recovered on day 21 after the start of a cycle	No dose modification	No dose modification
ANC < 0.75 x 10^9/L or<br>Platelet count < 75 x10^9/L<br>But recovered between day 22 to 28 after the start of a cycle	No dose reduction	Decrease temozolomide to dose level -1
ANC < 0.75 x 10^9/L or<br>Platelet count < 75 x10^9/L<br>But recovered between day 29 to 35 after the start of a cycle	Decrease temozolomide to dose level -1<br>Decrease irinotecan to dose level-1	Decrease temozolomide to dose level –2<br>Decrease irinotecan to dose level -2
ANC < 0.75 x 10^9/L or<br>Platelet count < 75 x10^9/L<br>On day 36 after the start of the cycle	Consider discontinuation of study treatment*	Not applicable
Liver function: Elevation of AST/ALT grade ≥3 that	No dose modification	No dose modification

recovers to grade ≤1 before day 21		
Liver function: Elevation of AST/ALT grade ≥3 not recovered to grade ≤1 before day 21	Decrease temozolomide to dose level -1	Discontinue study treatment
Grade 3 and 4 diarrhoea >3 days despite maximum loperamide therapy	Decrease irinotecan dose to dose level -1<br>If the same level of toxicity persists > 2 weeks despite suitable symptomatic treatment, discontinue study treatment<br>If diarrhoea is ongoing on day 21, delay next cycle for up to 2 weeks until diarrhoea resolves to ≤ grade 1<br>If the diarrhoea does not resolve after a 2-week delay, the patient should discontinue study treatment	Decrease irinotecan dose level -2<br>If the same level of toxicity persists > 2 weeks despite suitable symptomatic treatment, discontinue study treatment<br>If diarrhoea is ongoing on day 21, delay next cycle for up to 2 weeks until diarrhoea resolves to ≤ grade 1<br>If the diarrhoea does not resolve after a 2-week delay, the patient should discontinue study treatment
Other grade ≥3 non haematological toxicity not recovered to grade ≤2 before day 21	Decrease both irinotecan and temozolomide to level -1	Discontinue study treatment

Note: All platelets cut-off values require no platelet transfusions within 72 hours of starting the cycle. All neutrophil cut-off values require being off G-CSF for at least 72 hours. For those patients with known bone marrow involvement, the cut-off values required are ANC ≥0.5 x10^9/L and platelets ≥50 x10^9/L

*If not recovered 15 days after expected date of start (i.e. day 36 after start of cycle), refer to Sponsor to discuss individual case

Table 11: Arm TTo - Dose reduction and discontinuation rules

Type of Toxicity	Dose modification at first occurrence	Dose modification at second occurrence
ANC < 0.75 x 10^9/L or<br>Platelet count < 75 x10^9/L<br>But recovered on day 28 after the start of a cycle	No dose modification	No dose modification
ANC < 0.75 x 10^9/L or<br>Platelet count < 75 x10^9/L<br>But recovered between day 29 to 35 after the start of a cycle	No dose reduction	Decrease temozolomide to dose level -1
ANC < 0.75 x 10^9/L or<br>Platelet count < 75 x10^9/L<br>But recovered between day 36 to 42 after the start of a cycle	Decrease temozolomide to dose level -1<br>Decrease topotecan to dose level-1	Decrease temozolomide to dose level –2<br>Decrease topotecan to dose level -2

ANC < 0.75 x 10^9/L or<br>Platelet count < 75 x10^9/L<br>On day 43 after the start of the cycle	Consider discontinuation of study treatment*	Not applicable
Liver function: Elevation of AST/ALT grade ≥3 that recovers to grade ≤1 before day 21	No dose modification	No dose modification
Liver function: Elevation of AST/ALT grade ≥3 not recovered to grade ≤1 before day 28	Decrease temozolomide to dose level -1	Discontinue study treatment
Other grade ≥3 non haematological toxicity not recovered to grade ≤2 before day 28	Decrease both topotecan and temozolomide to level -1	Discontinue study treatment

Note: All platelets cut-off values require no platelet transfusions within 72 hours of starting the cycle. All neutrophil cut-off values require being off G-CSF for at least 72 hours. For those patients with known bone marrow involvement, the cut-off values required are ANC ≥0.5 x10^9/L and platelets ≥50 x10^9/L

*If not recovered 15 days after expected date of start (i.e. day 43 after start of cycle), refer to Sponsor to discuss individual case

Table 12: Arm BTTo - Dose reduction and discontinuation rules

Type of Toxicity	Dose modification at first occurrence	Dose modification at second occurrence
ANC < 0.75 x 10^9/L or<br>Platelet count < 75 x10^9/L<br>But recovered on day 28 after the start of a cycle	No dose modification	No dose modification
ANC < 0.75 x 10^9/L or<br>Platelet count < 75 x10^9/L<br>But recovered between day 29 to 35 after the start of a cycle	No dose reduction	Decrease temozolomide to dose level -1
ANC < 0.75 x 10^9/L or<br>Platelet count < 75 x10^9/L<br>But recovered between day 36 to 42 after the start of a cycle	Decrease temozolomide to dose level -1<br>Decrease topotecan to dose level -1	Decrease temozolomide to dose level –2<br>Decrease topotecan to dose level -2
ANC < 0.75 x 10^9/L or<br>Platelet count < 75 x10^9/L<br>On day 43 after the start of the cycle	Consider discontinuation of study treatment*	Not applicable
Liver function: Elevation of AST/ALT grade ≥3 that recovers to grade ≤1 before day 28	No dose modification	No dose modification
Liver function: Elevation of AST/ALT grade ≥3 not recovered to grade ≤1 before day 28	Decrease temozolomide to dose level -1	Discontinue study treatment

Other grade ≥3 non haematological toxicity not recovered to grade ≤2 before day 28	Decrease both topotecan and temozolomide to level -1	Discontinue study treatment

Note: All platelets cut-off values require no platelet transfusions within 72 hours of starting the cycle. All neutrophil cut-off values require being off G-CSF for at least 72 hours. For those patients with known bone marrow involvement, the cut-off values required are ANC ≥0.5 x10^{9}/L and platelets ≥50 x10^{9}/L

*If not recovered 15 days after expected date of start (i.e. day 43 after start of cycle), refer to Sponsor to discuss individual case

9.5.2 Bevacizumab – Infusion-related Reaction/Infusional Site Extravasation Management Guidelines

Table 13: Bevacizumab - Infusion-related reaction/infusional site extravasation management guidelines

Grade	Management
Grade 1<br>*Infusion-related Reaction*<br>Mild transient reaction; infusion interruption not indicated; intervention not indicated<br>*Allergic reaction*<br>Transient flushing or rash; fever < 38°C; intervention not indicated	If a Grade 1 infusion-related or allergic reaction occurs during the infusion, no treatment is needed. Supervise the patient and complete bevacizumab infusion at a 50% rate. If no reactions occur, the next dose can be administered at a 75-100% rate. If reactions re-occur, challenge at a 75% rate and continue to use this rate if no reactions occur. If reactions re-occur, challenge at 75%, use the 50% rate for subsequent administrations.
Grade 2<br>*Infusion-related and Allergic Reaction* (e.g. Rash, flushing, urticaria; dyspnoea, drug fever ≥ 38 ºC)<br>Intervention or infusion interruption indicated but responds promptly to symptomatic treatment (e.g., antihistamines, NSAIDS, narcotics); prophylactic medications indicated for ≤24 hrs)	When a Grade 2 reaction occurs, stop the bevacizumab infusion. Manage the infusion reaction according to institutional guidelines.<br><br>After recovery, resume infusion at 50% of the previous infusion rate for 15 minutes. If no further symptoms occur, complete the infusion at the reduced rate. Pre-medication should be given with the next infusion, but the infusion time may not be reduced.<br><br>If a Grade 2 infusion-related adverse reaction occurs, all subsequent infusions should be administered over the shortest period that was well tolerated. For example:<br>If an infusion-related AE occurred after the first administration of bevacizumab, the subsequent (i.e., the second) infusion must be administered over a slower infusion rate. If the infusion is then well tolerated with pre-medication, all subsequent infusions can be delivered over this extended infusion time. A possible gradual increase (i.e. from 50% to 75% infusion rate) is possible, provided that pre-medication is used. If grade 1 or 2 reactions occur at the increased rate, infusion should be continued at the previous tolerated rate for the whole treatment.

	If Grade 2 reactions occur at 50% rate despite appropriate pre-medications, further reduction in the infusion rate should be discussed with the Coordinating Sponsor and the discontinuation of bevacizumab may be considered.
Grade 3<br>*Infusion-related and allergic reaction*<br>Prolonged (e.g., not rapidly responsive to symptomatic medication and/or brief interruption of infusion); recurrence of symptoms following initial improvement; hospitalisation indicated for clinical sequelae (e.g., renal impairment, pulmonary infiltrates)<br>Anaphylaxis Symptomatic bronchospasm, with or without urticaria; parenteral intervention indicated per institutional guidelines; allergy-related oedema/angioedema; hypotension	The bevacizumab infusion should be stopped and not restarted on that day. At the physician's discretion, bevacizumab may be permanently discontinued or re-instituted with pre-medication and at a rate half that when the reaction occurred.<br><br>If the reaction occurred during administration at a 75% rate, initially re-challenge at a slower infusion rate (half of the rate when the reaction occurred) and gradually increase to 75% of the normal rate. When bevacizumab is re-started, the patient should be monitored, per physician's usual practice, for duration comparable to the duration of the initial reaction.
Grade 4<br>*Infusion-related and allergic reaction*<br>Life-threatening consequences; urgent intervention indicated per institutional guidelines<br><br>*Anaphylaxis*<br>Life-threatening consequences; urgent intervention indicated per institutional guidelines	In the event of a grade 4 reaction occurring during the infusion of bevacizumab, it is suggested that the following steps be taken:<br>Stop the bevacizumab infusion.<br>Maintain an adequate airway.<br>Administer antihistamines, corticosteroids, epinephrine, or other medications as required.<br>Continue to observe the patient, document observations, and administer further treatment according to the individual clinical case and clinical judgment.<br>Permanently discontinue bevacizumab.
Infusion Site Extravasation	
When extravasation of bevacizumab occurs during an infusion, it is recommended to take the following actions:<br>Discontinue the bevacizumab infusion.<br>Treat the extravasation according to institutional guidelines for extravasation of a non-caustic/ non-vesicant agent.	

9.5.3 Bevacizumab - Treatment Delays

The decision whether to continue bevacizumab administration should be based on an individual patient's circumstances and the physician's judgment that continuation is in the patient's best interest. Bevacizumab administration should be delayed if any of the side effects, outlined in Section 9.5.3, has been observed. Once a patient has met the re-treatment criteria (refer to Table 16: Bevacizumab- toxicity management guidelines), bevacizumab administration should recommence.

Note: Adverse Events should be classified according to whether, in the physician's judgement, these are likely to be related to Bevacizumab toxicity or non-Bevacizumab toxicity. These should be managed separately (see Figure 1: Classification of Adverse Events in the BEACON-Neuroblastoma Trial (bevacizumab randomisation)), and any dose modification or discontinuation required to chemotherapy (irinotecan ± temozolomide) deemed to last longer than one week should NOT affect the scheduling of Bevacizumab, even if this means that the two elements of the study treatment become unsynchronised. In order to help synchronisation of chemotherapy and bevacizumab, if the delay is only expected for a

week, all IMPs should be delayed and if the delay requires continuation longer than a week, the IMPs can be administered independently.

Table 14: Adverse Events requiring bevacizumab treatment delay

Adverse Event	Severity/Intensity (CTCAE grade)
Hypertension	Grade 2-3
Wound healing complications	Any grade
Proteinuria	Grade 3
Venous thrombosis/embolism (including vascular access device)	Grade 3 and asymptomatic grade 4
Any other clinically significant (CTCAE Grade 3/4) AEs that, according to the physician's discretion, are not clearly associated with chemotherapy and could be related to Bevacizumab	Grade 3 or 4

Regardless of the reason for the delay in bevacizumab, patients must discontinue bevacizumab when the administration of bevacizumab had to be interrupted for more than 6 weeks.

9.5.4 Bevacizumab - Discontinuation

Treatment with bevacizumab should be discontinued after a patient has been diagnosed with tumour progression, tumour recurrence, or second primary non-neuroblastoma malignancy, or if administration has been interrupted for more than 6 weeks. Bevacizumab administration should be permanently discontinued if a patient develops any of the bevacizumab adverse reactions as outlined in Section 9.5.4.

Note: Adverse Events should be classified according to whether, in the physician's judgement, these are likely to be related to Bevacizumab toxicity or non-Bevacizumab toxicity. These should be managed separately (see Figure 1: Classification of Adverse Events in the BEACON-Neuroblastoma Trial (bevacizumab randomisation), and any dose modification or discontinuation required to chemotherapy (irinotecan ± temozolomide) deemed to last longer than one week should NOT affect the scheduling of Bevacizumab, even if this means that the two elements of the study treatment become unsynchronised. In order to help synchronisation of chemotherapy and bevacizumab, if the delay is only expected for a week, all IMPs should be delayed and if the delay requires continuation longer than a week, the IMPs can be administered independently.

Table 15: Adverse Events requiring discontinuation of bevacizumab

Adverse Event	Severity/Intensity (CTCAE grade)
Hypertension	Grade 4 (Hypertensive crisis)<br>Hypertensive encephalopathy<br>Medically significant hypertension not controlled with medication
Left ventricular systolic dysfunction	Grade 3 or 4
Heart failure	Any grade
Gastrointestinal (GI) perforation	Any grade
Tracheo-oesophageal fistula	Any grade
Any non−tracheo-oesophageal fistula	Grade 4
Recto-vaginal fistulae	Grade 3-5
Proctalgia	Grade 3-4
Haemorrhage:	

Non-pulmonary or non-CNS<br>Pulmonary or CNS	Grade 3-4<br>Grade 2/3/4
Proteinuria	Grade 4
Posterior reversible encephalopathy syndrome (PRES)	Any grade
Venous thrombosis/embolism	Grade 4
Any arterial thrombosis/embolism	Any grade
Myocardial infarction	Any grade
Cerebrovascular ischemia	Transient ischaemic attacks (TIAs)<br>Cerebrovascular accidents (stroke)`
Osteonecrosis	Any grade
Eye disorders	Grade 4
Necrotising fasciitis	Any grade
Weight decrease	Grade 1-3

9.5.5 Bevacizumab - Toxicity Management guidelines

Treatment-emergent toxicities must be managed according to national and international guidelines. Management guidelines of expected toxicities are provided in Table 16: Bevacizumab- toxicity management guidelines.

Table 16: Bevacizumab- toxicity management guidelines

<table>
<tr><th colspan="2">Proteinuria</th></tr>
<tr><td colspan="2">The screening dipstick urinalysis for proteinuria must be ≤1+ before bevacizumab is administered. Any patient showing ≥2+ on urinary dipstick must have a urine sample sent for calculation of urine protein/creatinine ratio.
Urine protein/creatinine ratio is calculated dividing the concentration of protein in a spot sample by the concentration of creatinine in that sample. Both have to be measured in the same unit (mmol/L or mg/dl). Normal values: < 0.2.
Pr/Cr ratio has been shown to reflect 24-hour urine protein excretion quite accurately particularly since the first morning specimen eliminates the possibility of postural proteinuria.
A referral to a nephrologist is recommended when a patient develops prolonged proteinuria.</td></tr>
<tr><td>Grade 2
2+ - 3+ on urine dipstick
OR
Urine Pr/Cr (protein/Creatinine) ratio 0.5–1.9</td><td rowspan="2">Perform an early morning (first sample) Pr/Cr ratio or 24-hour urinary collection

Delay bevacizumab if clinically significant proteinuria is present:
1. Pr/Cr ratio > 1.9 or
2. 24-hour urinary protein excretion ≥ 0.5

Resume bevacizumab when either the:
1. Pr/Cr ratio < 1.9 or
2. 24-hour urinary protein excretion < 0.5 g

Permanently discontinue bevacizumab if:
Bevacizumab had to be delayed for > 6 consecutive weeks</td></tr>
<tr><td>Grade 3
Urine P/C > 1.9</td></tr>
<tr><th colspan="2">Wound Complications (Non-Infectious) / Wound Dehiscence</th></tr>
<tr><td>Grade 1
Incisional separation of ≤ 25% of wound, no deeper than</td><td>Delay bevacizumab until the wound has satisfactorily healed</td></tr>
</table>

superficial fascia<br>**Grade 2**<br>*Incisional separation of > 25% of wound, local care indicated*<br>**Grade 3**<br>*Primary wound closure or revision by operative intervention is indicated*	

**Hypertension**	
Age and sex-appropriate systolic and/or diastolic blood pressure that is persistently above the 95th percentile (ULN) requires further evaluation. It is strongly recommended that patients who develop hypertension during the study be evaluated in conjunction with a (paediatric) specialist.<br>In children, blood pressure varies with the age and is closely related to height and weight. Variability in blood pressure in children of similar age and body build should be expected, and it is recommended that serial measurements obtained when a patient's blood pressure is assessed.<br>Exercise, excitement, coughing, crying, and struggling may raise the systolic pressure of infants and children as much as 40-50 mmHg greater than their usual level. Steroid usage (e.g., tumour-related intracranial pressure, allergic reaction, emesis, etc.) may also increase blood pressure and should be weaned off as soon as clinical situation permits. Guidelines to age-specific percentiles of blood pressure can be accessed at http://www.nhlbi.nih.gov/health/prof/heart/hbp/hbp_ped.pdf and in Appendix 8 – Blood Pressure Levels by Age and Height Percentile	
**Grade 2**<br>*Recurrent or persistent (≥ 24 hours) BP increase > ULN; monotherapy indicated*	**Delay bevacizumab administration**<br>Initiate anti-hypertensive therapy<br><br>Resume bevacizumab once systolic and/or diastolic BP for age and sex is below the 95th percentile
**Grade 3**<br>*Requiring more than one antihypertensive drug or more intensive therapy than previously*	
**Grade 4**<br>*Life-threatening consequences (e.g., malignant hypertension, transient or permanent neurologic deficit, hypertensive crisis); urgent intervention required*	**Permanently discontinue bevacizumab**
**Venous Thrombo-Embolism (including vascular access device)**	
R**Grade 3**<br>*Thrombosis (e.g. uncomplicated pulmonary embolism (venous), non-embolic cardiac mural (arterial) thrombosis), medical intervention indicated*	**Delay bevacizumab**<br>Bevacizumab may be resumed once the patient has been fully anticoagulated and if the patient has not experienced a Grade 3 or 4 haemorrhagic event<br>**Low-molecular-weight heparin** should be prescribed and the treatment monitored in compliance with the approved product labelling or according to local clinical practice guidelines Similarly, for patients on a **coumarin derivative** or **unfractionated heparin**, the INR and APPT, respectively, should be within therapeutic range.<br>**Permanently discontinue bevacizumab** if the VTE worsens or recurs after resuming therapy

9.6 Central Venous Access Device (CVAD)

Due to the increased risk of potentially severe wound healing complications in patients exposed to bevacizumab, treatment with bevacizumab must not start earlier than four weeks (28 days) after the last major surgery.

Patients receiving bevacizumab usually require the placement of a CVAD, which is considered a minor surgery on this study. CVAD should ideally be placed 7 days prior to the first bevacizumab administration; however, should this not be possible, a minimum 48-hour interval before the start of bevacizumab administration is also acceptable, provided that the wound has satisfactorily started to heal.

Taken all of the above into consideration, sites are encouraged to plan ahead of time the placement of the CVAD (i.e., booking of a surgical room, pre anaesthesiology visit, etc.) so that placement can occur while respecting the 48-hour interval before starting bevacizumab administration.

Of note, replacement of the CVAD (e.g., central line infection or malfunction) during bevacizumab treatment requires additional precautions. No antidote exists to bevacizumab and its relatively long half-life does not allow for pre-emptive interruption in case of an emergency. Thus, CVAD replacement in patients receiving bevacizumab should be performed by an experienced surgeon or interventional radiologist. Consideration may be given to the placement of a peripherally-inserted central (PICC) line. In all cases, careful examination of the wound healing process should be performed before the next planned dose of bevacizumab is administered, at least 48 hours after line replacement. Any suspicion of wound healing complication should mandate interrupting bevacizumab until complete wound healing is confirmed (for bevacizumab interruption beyond six weeks, please see section 9.5.3). Sutures should be left in place until the wound has satisfactorily healed.

For supportive treatment and concomitant medication please refer to Sections 12 and 13 respectively.

10 DINUTUXIMAB BETA AND TOPOTECAN RANDOMISATIONS

10.1 Eligibility for the dinutuximab beta randomisation

10.1.1 Inclusion criteria for the dinutuximab beta randomisation

Disease specific

- Histologically proven neuroblastoma as per International Neuroblastoma Staging System (INSS) definition[1]
- Relapsed or refractory neuroblastoma
    - Relapsed: any relapsed or progressed high-risk neuroblastoma
    - Refractory high risk disease: Lack of adequate response to frontline therapy that precludes the patient from proceeding to consolidation therapies (e.g. myeloablative chemotherapy)
- Measurable disease by cross sectional imaging (RECIST) or evaluable disease (uptake on MIBG scan with or without bone marrow histology). Patients with bone marrow detectable disease only (bone marrow aspirate or trephine) are NOT eligible for the study

General

- Age ≥1 to ≤21 years
- Informed consent from patient, parent or guardian

Performance status and organ function

- Performance status:
    - Lansky ≥ 50%, Karnofsky ≥ 50% or ECOG ≤3
      (Patients who are unable to walk because of paralysis, but who are able to sit upright unassisted in a wheelchair, will be considered ambulatory for the purpose of assessing performance score)
- Bone marrow function (within 72 hours of randomisation):
    - No bone marrow disease:
        - Platelets ≥75 x 10^9/L (unsupported for 72 hours)
        - ANC ≥ 0.75 x10^9/L (no G-CSF support for 72 hours)
        - Haemoglobin *≥ 8 g/dL* (transfusions allowed)
    - Bone marrow disease:
        - Platelets ≥ 50 x10^9/L (unsupported for 72 hours)
        - ANC ≥ 0.5 x 10^9/L (no G-CSF for 72 hours)
        - Haemoglobin *≥ 8 g/dL* (transfusions allowed)
- Renal function (within 7 days of randomisation):
    - Serum creatinine ≤ 1.5 ULN for age, if higher, a calculated GFR (radioisotope or 24 hour urine calculated creatinine clearance) must be ≥ 60 ml/min/1.73 m^2
- Liver function (within 72 hours of randomisation): AST or ALT *≤ 3.0 ULN* and total bilirubin ≤1.5 ULN. In case of liver metastases, AST or ALT ≤ 5 ULN and Total bilirubin ≤ 2.5 ULN
- Cardiac function, measured by echocardiogram within 4 weeks of randomisation or within 12 weeks if the patient has not received anthracyclines or cardiotoxics in between. Shortening fraction ≥ 29% on echocardiogram
- Adequate lung function; no dyspnoea at rest and pulse oximetry > 94% in room air
- Females of childbearing potential must have a negative serum or urine pregnancy test within 72 hours prior to initiation of treatment. Sexually active women of childbearing potential must agree to use acceptable and appropriate contraception during the study and for at least 6 months after the last study treatment administration. Sexually active males patients must agree to use condom during the study and for at least 6 months after the last study treatment administration.
- Availability and willingness to place a double central venous access if needed for trial treatment and supportive care in case of treatment with chemo-immunotherapy

Dinutuximab beta

10.1.2 Exclusion criteria for the dinutuximab beta randomisation

- Previous treatment with temozolomide
- Previous treatment with chemotherapy in combination with anti-GD2 directed therapy ("chemo-immunotherapy") with any anti-GD2 antibody. Prior treatment with anti-GD2 directed therapy alone with/without cytokines is allowed provided a 4 week wash-out period is met
- Known hypersensitivity to:
  - Any study drug or component of the formulation
  - Patients with mild previous hypersensitivity reactions to anti-GD2 antibodies may be included, but those with severe (or G4) hypersensitivity reactions to antiGD2 antibodies will be excluded
- Clinically significant neurological deficit, uncontrolled seizures or objective peripheral neuropathy (>grade 2). (Unresolved neurological deficits from previous spinal cord compression are acceptable)
- Uncontrolled infection
- Inadequate recovery from prior surgery with no ongoing ≥Grade 3 surgical complications. For core biopsies, no less than 24 hours; for open excisional biopsies, no less than 48 hours; for major surgery, no less than 2 weeks.
- Patient less than (at point of planned date of randomisation):
  - Two weeks from prior chemotherapy. One week from prior oral metronomic chemotherapy (i.e. oral etoposide or oral cyclophosphamide).
  - Six weeks from prior craniospinal radiotherapy or MIBG therapy and two weeks from radiotherapy to the tumour bed. No washout is required for palliative radiotherapy
  - Eight weeks from prior high dose chemotherapy with autologous haematopoietic stem cell rescue
  - Three months from prior allogeneic stem cell transplant, no ongoing treatment with immunosuppressive agents and no signs of ≥grade 2 acute graft versus host disease
  - 14 days or 5 half-lives (whichever occurs later) from last administration of an IMP in an IMP-trial.
  - 14 days or 5 half-lives (whichever occurs later) from last administration of any other biological/targeted anticancer agent
- Bleeding metastases (Patients with CNS metastases can be enrolled as long as the metastases are not bleeding)
- Pregnant or lactating patient
- Any uncontrolled medical condition that poses an additional risk to the patient
- Low probability of treatment compliance

10.2 Schedule of events for the dinutuximab beta and topotecan randomisations

Protocol Activity	Screen / Baseline	Pre Dosing (All Cycles)	Post Cycles 2, 4 (within 7 days of starting the next cycle)	Post Cycle 6 & End of Treatment[18] (28 days after Cycle 6 dose and Last Dose given)	Follow Up[19]
Informed Consent/Assent	X				
Medical History	X				
Physical Examination and Performance status (including pupil responses)[1]	X	X		X	X
Visual acuity [1]	X	Repeat if clinically indicated			
Weight and Height[2]	X	X		X	X
**Laboratory Tests**					
Haematology[3]	X	X		X	X
Blood Chemistry[3]	X	X		X	X
Pregnancy Test[4]	X			X	
Glomerular Filtration Rate (GFR[5])	X				
**Tumour Assessment**					
MRI or CT Brain[6]	X				
MRI (preferred) or CT scan of tumour[6]	X		X	X	
I[123]-mIBG[6] or [18]FDG PET/CT Scan	X		X	X	
Bilateral Bone Marrow Aspirate and Trephine[6]	X		X	X	
Miscellaneous:					
hocardiogram[7]	X				
Oxygen saturations[8]	X	X *For patient randomised in the dinutuximab beta arms only*			
Chest x-ray[9]	X				
Tanner staging[10]	X			X	X
Menstrual Status[10]	X			X	X
Adverse Events	**X** (assess throughout study)				

Dinutuximab beta

Concomitant Medications	**X** (assess throughout study)				
**Biomarkers: For all biomarker samples, please check the BEACON-Neuroblastoma Laboratory Manual**					
**Protocol Activity**	**Screen / Baseline**	**Pre Dosing (All Cycles)**	**Post Cycles 2, 4 (within 7 days of starting the next cycle)**	**Post Cycle 6 & End of Treatment[18] (28 days after Cycle 6 dose and Last Dose given)**	**Follow Up[19]**
Blood for analysis of mRNA RT-qPCR (TH, PHOX2B, DCX)[11]	**X**		**X**	**X**	
Blood for $F_c$gamma and KIR receptor genotype - *all patients* [12]	**X**				
Bone marrow aspirate for analysis of mRNA RT-qPCR (TH, PHOX2B, DCX)[11]	**X**				
Blood sample for exploratory biomarkers[13]	**X**		**X**	**X**	
Whole blood sample for DNA[14]	**X**				
Immunophenotyping [11] - *For patient randomised in the dinutuximab beta arms only*[15]		**Day 1 and 8[20] of cycle 1, 3 and 5**		**X**	
Antibody Dependent Cellular Cytotoxicity (ADCC) *For patient randomised in the dinutuximab beta arms only*		**Day 1 and 8[20] of cycle 1 only**			
Dinituximab beta PK and ADA (Dinituximab beta arm only)[16]	**X**	**X**	**X**	**X**	
Tumour Molecular Profiling (Formalin Fixed Paraffin Embedded or Frozen tissue) [17]	**X**				

Note: Not all sample collections will be ongoing at any one time. See BEACON-Neuroblastoma Lab Manual for current sample collections.

Dinutuximab beta

1. Performance status will be reported using the Lansky scale for 1-12 year olds and using the Karnofsky/ECOG scale for older patients. Performance status and physical examination (including BP, HR and Temperature) will be performed at screening/baseline and within 24 hours prior to each cycle in all treatment arms. Both examinations will also be performed at the end of treatment visit, and at each follow up visit (see footnote 17). Menstrual status (regularity of menstruation) in females of child bearing potential should be assessed at screening, end of treatment and during follow up. A clinical assessment of vision and pupil responses is mandated at baseline. A clinical assessment of pupil responses should be conducted before starting each cycle. If there are any concerns about vision or pupil responses then during treatment or follow up, a referral to an ophthalmologist should be considered.
2. Patient's weight will be measured at screening, on day 1 of each cycle prior to dosing and at the end of treatment. Body height (standing or lying length where age-appropriate) will be measured at screening and at the end of treatment. After end of treatment, weight and height will be performed at each follow up visit (see footnote 17).
3. Haematology and biochemistry blood tests must be done at screening and within 72 hours prior to cycle 1 dosing. Unless required by the investigator, safety tests will not be repeated at cycle 1, day 1 prior to dosing. Beginning in cycle 2 and continuing for cycles 3, 4, 5, and 6, pre-dose activities can be done within 72 h pre-dose. It is recommended that blood counts are monitored weekly for all patients. Additional safety assessments may be done according to institutional standard of care. These tests must also be performed at end of treatment. Haematology includes - haemoglobin (Hb), white blood cells (WBC) with differential count, neutrophils lymphocytes and platelets. Biochemistry includes - sodium, potassium, calcium, urea, creatinine, total protein, albumin, bilirubin, alkaline phosphatase (ALK PHOS), GGT, LDH, ALT or AST.
4. For girls who are post-menarchal, a urine (preferred) or serum pregnancy test will be done at screening (within 72 hours of dosing), after 6 cycles and at the end of treatment. If the results are inconclusive, a repeat test must be performed using a urine sample. In addition, a pregnancy test will be done whenever one menstrual cycle is missing during treatment or a potential pregnancy is suspected. Girls who become pregnant while on study will be discontinued immediately and the outcome of the pregnancy will be followed. Pregnancy tests may be repeated during study if required by hospital regulations and/or Research Ethics Committees (RECs).
5. Calculated GFR (radioisotope or 24 hour urine calculated creatinine clearance) should be carried out at screening within 7 days of eligibility assessment in patients with a level of serum creatinine > 1.5 ULN for age. An estimated GFR is sufficient using the Schwartz formula. Only if this is deranged, the test has to be repeated.
6. Tumour assessments: (Must comply with RECIST 1.1) The minimum requirement is a cross sectional image of site of measurable disease by MRI (preferred) or CT, performed within 4 weeks prior to receiving the first dose of trial treatment. A scan including the brain (either CT or MRI) must also be performed at screening to assess patient's eligibility with regard to the presence of bleeding brain metastases. At baseline, MIBG scans (or 18-FDG PET/CT scan if MIBG negative disease) and bilateral bone marrow aspirates and trephine are to be done within 4 weeks prior to receiving the first dose of trial treatment. Bone marrow assessments will be done in all patients at screening and will only be repeated after 2, 4 and 6 courses if positive at screening or clinically indicated (until they become negative). Patients with negative bone marrow assessments at screening will not have repeated BM samples. To ensure comparability, the same scan, equipment, method and technique used during baseline should be consistently used throughout the study. Additional investigations of possible metastatic sites should be done upon presentation of signs and symptoms. Tumour lesions previously irradiated will be considered measurable only if progressing. Scans should be completed within 7 days prior to cycles 3 and 5; following cycle 6; and at end of treatment if the patient discontinues treatment early. Response assessment will be performed with the same modalities used at screening (MRI/CT/MIBG/bone marrow histology as appropriate). Tumour assessments do not have to be repeated where the following cycle is delayed due to toxicity.
7. An echocardiogram should be performed within 4 weeks of randomisation. If the patient has had an echocardiogram within 12 weeks of eligibility assessment and has not received anthracyclines or cardiotoxics then this echocardiogram can be used for screening and does not need to be repeated for this study.
8. Sites should ensure that oxygen saturation is ≥ 94% each cycle in order to proceed administering dinutuximab beta with chemotherapy
9. Chest x-ray to be repeated prior to subsequent courses if any respiratory signs or symptoms develop and deemed clinically relevant
10. Tanner staging and menstrual status (if applicable) should be completed at screening within 4 weeks of commencing trial treatment. For patients undergoing follow up visits following end of treatment, Tanner staging and menstrual status should be collected annually.
11. **Please refer to current BEACON-Neuroblastoma Laboratory Manual before taking samples**. Blood samples will be obtained at baseline, post Cycles 2, 4 and 6 for the molecular monitoring analysis of mRNA by qRT-PCR for TH, PHOX2B and DCX in all patients. Bone Marrow samples (right and left) for the measurement of circulating neuroblastoma mRNA should also be taken from patients at screening.
12. Mandatory in all patients. Please refer to **current BEACON-Neuroblastoma Laboratory Manual** for details.
13. Blood samples for the evaluation of exploratory biomarkers will be collected at baseline, post cycle 2, post cycle 4 and post cycle 6 (samples can be collected the same day as starting the next cycle, but have to be collected before study drug administration). Samples will be taken at EOT if the patient discontinues treatment early. Samples will be taken from all patients.
14. A blood sample for constitutional DNA analysis will also be performed at baseline. Samples for constitutional DNA analyses are optional.
15. Performed in local labs where feasible. Day 1 and 8 of cycles 1, 3 and 5. Please refer to **current BEACON-Neuroblastoma Laboratory Manual** for suggested flow cytometry panel.

16. Baseline, immediately prior to starting next cycle (trough) and end of infusion (peak) in all cycles only in dinutuximab beta patients.
17. Tumour molecular profiling: The evaluation of neuroblastoma related biomarkers will be performed on samples of tumour tissue collected at the time of diagnosis or during frontline therapy and then the point of relapse if a biopsy/surgery is performed for clinical reasons (this sample is mandatory for study entry, if no tumour is available this will be discussed with the Coordinating Sponsor and the Chief Investigator **before** study entry). Archival tumour tissue either as paraffin embedded or frozen material will be collected.
18. Response assessment of the Primary Endpoint takes place Post Cycle 6. Patients completing treatment at cycle 6 will have an End of Treatment visit approximately 28 +7 days after the Cycle 6 dose. Patients may continue to receive **chemotherapy only** trial treatment following Cycle 6, following discussion with the Coordinating Sponsor and CI. Patients receiving cycles 7-12 will have the same clinical and laboratory assessments at each cycle and have a response assessment within 1 week of starting cycles 7 and 10. NB- The Post cycle 6 assessments may be used as the Pre Cycle 7 tests. Patients who discontinue treatment early (prior to cycle 6) will have an End of Treatment visit approximately 28 days after the last dose given.
19. A follow up visit will be scheduled 90 ± 15 days after the last dose of trial treatment, unless the patient withdraws consent or receives another treatment. Patients that develop relapse or start further therapy will not undergo further follow up visits but will still have survival and disease status data collected for them at the time points for follow up visits. For those patients that remain relapse-free and do not start any further therapy, follow up visits will be done 3-monthly for one year and 6-monthly for up to 5 years. Additional follow up visits will be scheduled, as needed to monitor any sustained unresolved, treatment emergent adverse events.
20. Day 8 samples to be taken prior to completion of dinutuximab beta infusion.

10.3 Trial therapy (dinutuximab beta and topotecan randomisations)

The bevacizumab randomisation was completed following the recruitment of 160 patients, on 07 February 2019. On 01 August 2019 dinutuximab beta was added to two backbone chemotherapy regimens (T, TTo), thereby creating 4 treatment arms: temozolomide (Arm T), dinutuximab beta + temozolomide (Arm dBT), temozolomide + topotecan (Arm TTo) and dinutuximab beta + temozolomide + topotecan (Arm dBTTo).
However, following review and discussion of the results of the bevacizumab and irinotecan randomisations for EFS and OS in the BEACON-Neuroblastoma trial by the TMG, it was concluded that T alone is inferior. Therefore, from 28 January 2020, patients will be randomised to one of the following two arms:

TTo: Temozolomide + Topotecan

dBTTo: Dinutuximab beta + Temozolomide + Topotecan

Also, it was recommended that patients who were randomised to T or dBT and were receiving these regimens as part of the trial at the point of implementation, should be made aware of the TMG recommendation to switch to TTo (for those randomised to T) or to dBTTo (for those randomised to dBT).

Figure 2 Dinutuximab beta amendment trial therapy overview

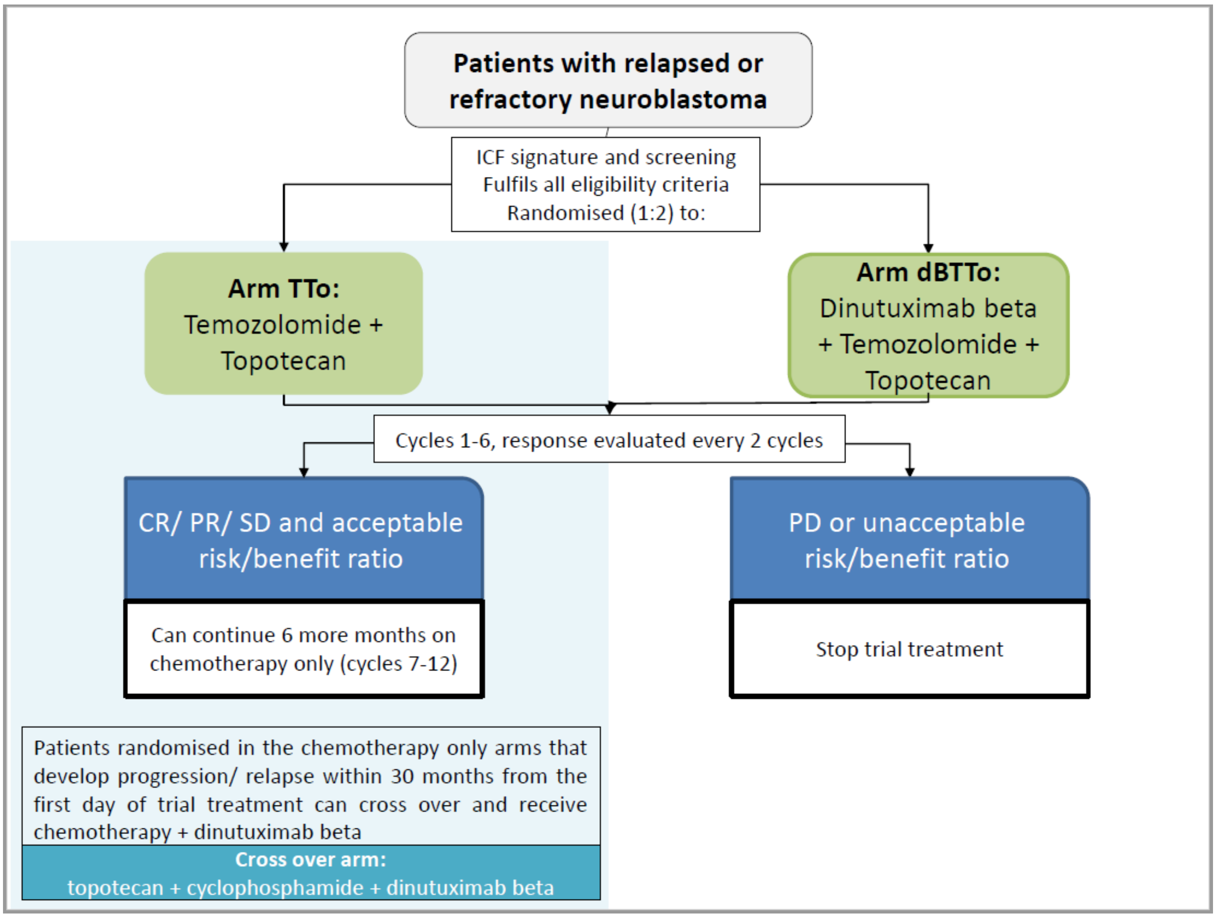

Six cycles of treatment will be given. Subsequent cycles should start every 4 weeks provided adequate recovery from prior toxicities.

After six cycles, patients without evidence of progressive disease and acceptable toxicity profile can continue trial treatment upon agreement of the sponsor and the CI. These patients may receive up to six more courses of the chemotherapy regimen they were assigned at randomisation without dinutuximab beta.

Patients who are randomised to chemotherapy alone and who experience progression or relapse within 30 months from starting trial treatment and are fulfilling specific criteria detailed in Section 10.4.6 will be allowed to cross-over to a chemotherapy (topotecan + cyclophosphamide) plus dinutuximab beta containing regimen for 6 cycles after approval from the sponsor and the CI. Refer to Section 10.4.6 for details of the cross-over aspect of the trial.

10.3.1 Dinutuximab beta and topotecan trial treatment

Trial drugs will be administered to patients within this trial according to the treatment arm that the patient is allocated to:

Table 17: Treatment Schedule in the BEACON-Neuroblastoma - dinutuximab beta randomisation

For each treatment arm a cycle consists of:

**Arm T –closed to new randomisations**		
Days 1 – 5	Temozolomide 200 mg/m^2/day PO	Every 4 weeks
**Arm dBT–closed to new randomisations**		
Day 1 – 7	Dinutuximab beta 10 mg/m^2/day 24h iv infusion	Every 4 weeks
Days 1 – 5	Temozolomide 200 mg/m^2/day PO	Every 4 weeks
**Arm TTo**		
Days 1 – 5	Temozolomide 150 mg/m^2/day PO	Every 4 weeks
Days 1 – 5	Topotecan 0.75 mg/m^2/day IV	Every 4 weeks
**Arm dBTTo**		
Day 1 – 7	Dinutuximab beta 10 mg/m^2/day 24h iv infusion	Every 4 weeks
Days 1 – 5	Temozolomide 150 mg/m^2/day PO	Every 4 weeks
Days 1 – 5	Topotecan 0.75 mg/m^2/day IV	Every 4 weeks

Dinutuximab beta: If allocated to arms dBT or dBTTo the dose of dinutuximab beta will be 10 mg/m^2/day given through a 24-hour infusion for 7 days (days 1-7).

Patients should remain admitted as inpatients during the first course of dinutuximab beta with chemotherapy. For subsequent courses, institutional practice can be applied, and patients can be discharged on ambulatory pumps if clinically well.

Temozolomide: The dose of temozolomide will be 150 mg/m^2/day when administered in combination with Topotecan or 200 mg/m^2/day when administered alone for five days orally depending on the allocated arm. Where possible, doses will be rounded to the nearest 5mg as per the table in **Appendix 7 – Temozolomide Dosing**.

Topotecan: The dose of topotecan will be 0.75 mg/m^2/day for five days. Topotecan will be given intravenously over 30mins on days 1-5, at least one hour following the administration of temozolomide.

The administered dose of chemotherapy may be rounded up or down to the nearest dose that can be accurately measured.

NOTE: Although the toxicity profiles of dinutuximab beta and temozolomide ± topotecan are different, all drugs should be administered together. If there are delays to the chemotherapy due to myelotoxicity, the administration of dinutuximab beta should also be delayed.

The patient's height and weight (BW) must be measured prior to each cycle of treatment.

Temozolomide, topotecan and dinutuximab beta are prescribed per body surface area (BSA) except for those with low weight (see below). **The same dose should be given to the patient every cycle, using the doses calculated using the screening or Cycle 1 BW and BSA, UNLESS the calculated dose(s) have changed by ≥10% from the values obtained at screening/cycle 1.**

Dosing for low weight patients

For patients with BSA ≤ 0.5 m^2, temozolomide will be dosed per kg (see Appendix 7 – Temozolomide Dosing). Topotecan dosing will be 0.025mg/kg/day IV as a 30 minute infusion for 5 days for patients with low weight (<12kg). Dinutuximab beta will be dosed on BSA in all patients.

Dosing for patients with obesity/overweight

If a patient's BW exceeds that for the 98th centile for their age:

- Dinutuximab beta, temozolomide and topotecan doses should be calculated using the BSA obtained from the Mosteller formula, using the BW for the 98th centile and the patient's actual height.

In all cases, the dosing of overweight patients should be discussed and agreed with the Sponsor and Clinical Coordinator before the administration of any IMP.

10.4 Treatment Schedule

The required evaluations at baseline (screening), on study, and at end of treatment are summarised in the Schedule of Activities (See 10.2) which should be consulted in the first instance. Other tests and/or increased frequency of examinations or clinical follow up may be needed for patient management based on the PI's clinical judgement, the results of which will be recorded on the CRF.

The first day of a cycle is defined as the first day on which study treatment is administered. In the event of scheduling conflicts due to administrative reasons, dosing and study evaluations may take place on the designated day 1 (D1) ±3 days (see Schedule of Activities in the synopsis).

10.4.1 Day 1 of Cycle 1

- A physical examination (including weight, height, blood pressure, pulse, performance status and pupil reflexes) should be performed within 24 hours before dosing of the first cycle
- A clinical assessment of vision and pupil responses is mandated at baseline. Where clinically relevant during treatment or follow up, a referral to an ophthalmologist should be considered.
- Oxygen saturation must be performed within 24 hours before dosing of the first cycle. It must be ≥94% to proceed administering dinutuximab beta with chemotherapy.
- Patient's blood samples (haematology, biochemistry) should be drawn and reviewed up to 72 hours prior to dosing

10.4.2 Day 1 of subsequent cycles

- A physical examination (including height, weight, blood pressure/pulse, performance status and pupil reflexes) should be performed within 24 hours before dosing

- Oxygen saturation must be performed within 24 hours before dosing of each cycle. It must be ≥94% to proceed administering dinutuximab beta with chemotherapy. Patient's lab tests (haematology, biochemistry) should be drawn and reviewed up to 72 hours prior to re-dosing. Full blood count, creatinine, electrolytes and liver function (ALT, AST, bilirubin) should be checked weekly
- Tumour assessment scans will be performed every other cycle starting at end of cycle 2, 4, and 6 (prior to cycle 3) as detailed in section 7.4.3.
- For patients with positive bone marrow involvement by local morphology (aspirates or biopsies), bilateral bone marrow aspirates and trephines assessed by local morphology will also be collected every other cycle starting at the end of cycle 2 (prior to cycle 3) as detailed in Section 10.2

Criteria to start a new cycle: Before initiating a new treatment cycle adequate bone marrow, renal and liver functions as defined by the following should be established:

- The patient has not met any of the criteria for stopping protocol therapy because of disease progression (See Section 13.1). Toxicity induced from previous cycle recovered to ≤ Grade 2
- As a general principle, the same criteria used at study entry for haematology, renal and liver function should be used to start subsequent cycles. Section 10.5 provides guidance on dose modifications for chemotherapy and dinutuximab beta. Administration of dinutuximab beta should be started in an inpatient setting but can be continued in an ambulatory / outpatient setting if well tolerated and only oral supportive care /analgesia is needed. Details regarding treatment infusion can be found in the pharmacy manual.

10.4.3 Post Cycle 6 (For patients continuing to Cycle 7-12 on chemotherapy only)

A full assessment will be carried out Post Cycle 6 for patients continuing trial treatment to Cycles 7-12. The following will be performed before the start of the next cycle:

- A tumour scan (MRI/CT/MIBG/18-FDG PET/CT) will also be performed +/- functional imaging scan and bone marrow sample, if previous involvement. If one modality shows progressive disease (MRI/CT, MIBG or bone marrow histology) it will not be necessary to perform all modalities
- Two blood samples for the molecular monitoring mRNA analyses and exploratory biomarkers should be collected before study drug administration

The following can be performed as part of the Pre-Cycle assessment for Cycle 7.

- A complete physical exam will include performance status, height (both sitting and standing), weight and blood pressure/pulse)
- All safety laboratory samples will be collected (haematology, blood biochemistry).
- A urine (preferred) or serum pregnancy test will be done on girls who are post-menarche

10.4.4 End of Treatment

An End of Treatment visit will take place approximately 28±7 days after the last dose of the last cycle of trial treatment or sooner if the patient develops PD or withdraws from the study. The following will be performed:

- A complete physical exam will include performance status, height, weight, visual acuity, pupil response and blood pressure/pulse)
- All safety laboratory samples will be collected (haematology, biochemistry)
- A urine (preferred) or serum pregnancy test will be done on girls who are post-menarche
- A blood sample for exploratory biomarkers should be collected at the end of treatment and the point of disease progression
- A tumour scan (MRI/CT/MIBG/^{18}FDG PET/CT) will also be performed +/- functional imaging scan and bone marrow sample, if previous involvement. If one modality shows progressive disease (MRI/CT, MIBG or bone marrow histology) it will not be necessary to perform all modalities

- Tanner staging

10.4.5 Treatment Duration

Patients with a response (CR, PR) or stable disease (SD) and acceptable risk/benefit ratio after completing 6 cycles of trial treatment, can continue chemotherapy only (without dinutuximab beta) trial treatment for up to an additional 6 cycles following discussion with the Sponsor and Chief Investigator.

Patients receiving cycles 7 to 12 will have the same clinical and laboratory assessments at each cycle (physical examination, performance status, vital signs, full blood count, biochemistry, clotting and urine dipstick and/or protein/creatinine ratio). These patients will have a response assessment (CT/MRI, MIBG/18-FDG PET/CT scan ± bone marrow aspirate/trephine) within one week of starting cycles 7, 10 and at end of treatment. No biomarker samples/scans are required.

10.4.6 Cross-over

Patients who are randomised to chemotherapy alone arms (T or TTo) on the dinutuximab beta randomization and who experience progression or relapse within 30 months of starting trial treatment will be allowed to cross-over to a chemotherapy plus dinutuximab beta containing regimen for 6 cycles, provided the following conditions are fulfilled:

- Progression/relapse is confirmed by the CI/Clinical Coordinator upon review of the most recent and previous response assessment CRF (unequivocal evidence of PD by RECIST or INRC must be shown).
- It is the first progression/relapse after the dinutuximab beta randomisation. No previous therapies for the first progression/relapse after the dinutuximab beta randomisation are allowed other than emergency chemotherapy.
- Progression/relapse occurs from the first scheduled trial response assessment (after 2 cycles) onwards.
- Patients should have received at least 80% of their protocol defined treatment.
- Patients that experienced a response or stable disease and received consolidation or maintenance-type therapies after completing six courses of trial treatment (e.g. MIBG therapy) are eligible for the cross-over provided adequate wash-out periods are fulfilled (as per the eligibility criteria for dinutuximab beta randomisation).

The regimen recommended for the combination will be topotecan-cyclophosphamide (London et al. 2010):

Table 18: Cross-over treatment overview

**Cross-over to topotecan-cyclophosphamide-dinutuximab beta for 6 cycles**		
Days 1 – 5	Topotecan 0.75 mg/m^2/d iv, infused over 30 minutes	Every 4 weeks
Days 1 – 5	Cyclophosphamide 250 mg/m^2/day iv<br>Infused over 60 minutes, with 4 hours hydration (starting 2 hours before the start of cyclophosphamide) at 125 ml/m^2/hour as per institutional practice	Every 4 weeks
Day 1 – 7	Dinutuximab beta 10 mg/m^2/day 24h iv infusion	Every 4 weeks

For patients with weight ≤12 kg, cyclophosphamide will be dosed at 8.3 mg/kg/day and topotecan at 0.025mg/kg/day. Patients should be closely monitored for safety only and should try to adhere to the relevant activities as outlined in the schedule of activities (Section 10.2). Disease status should be assessed according to local practice. Dinutuximab beta will be dosed on BSA in all patients. Any

toxicities resulting from the topotecan plus cyclophosphamide treatment regimen **(including the occurrence of haemorrhagic cystitis)** should be managed in accordance to local toxicity management guidelines.

10.5 Dose Modifications – dinutuximab beta and topotecan randomisations

Patients should be carefully monitored for toxicity. After the initial treatment cycle, dose reductions and/or administration delays/discontinuation will be decided using specific predefined rules to accommodate individual tolerance of treatment and maintain protocol dose intensity. All toxicities will be graded according to version 4 of the NCI CTCAE. Doses are to be adjusted based on the most severe toxicity that the patient experiences, related or possibly related to the study treatment between each cycle of treatment.

The maximum delay to the administration of chemotherapy/ dinutuximab beta due to adverse events is 2 weeks beyond due start date (i.e. 43 days from start of previous cycle). The delay may only be extended over 43 days following agreement from the Sponsor.

As a general principle, the same criteria used at study entry for haematology, renal and liver function should be used to start subsequent cycles. Full doses of all drugs will be administered only if toxicities are resolved to grade 2 or less prior to start of the cycle. This section provides guidance on dose modifications for toxicities related to chemotherapy and dinutuximab beta.

Adverse events should be classified into whether at the Investigator's judgment these are likely to be related to dinutuximab beta toxicity, chemotherapy toxicity, both or not related and managed accordingly following the advice given in the following sections.

10.5.1 Dose modifications for dinutuximab beta specific toxicities

Table 19: Dose modification for dinutuximab beta specific toxicities

Adverse event	Dose Modification of dinutuximab beta
Anaphylaxis	• *≤ Grade 2; maximise anti-histamines and supportive care; consider reducing to 50% rate if worsening symptoms*<br>• *Grade 3:* Interrupt infusion and give supportive measures. Resume at 50% rate when resolves to ≤ Grade 2<br>• ***Recurrent Grade 3 or any Grade 4:*** **permanently discontinue dinutuximab beta**
Capillary leak syndrome	• *≤ Grade 2:* decrease infusion to 50%. Resume to 100% if resolves with supportive measures.<br>• *Grade 3:* Interrupt infusion and give supportive measures. Resume at 50% rate when resolves to ≤ Grade 2<br>• ***Recurrent Grade 3 or any Grade 4:*** **permanently discontinue dinutuximab beta**
Fever	• If persistently 40ºC despite maximal supportive measures then pause dinutuximab beta infusion and resume once toxicity has resolved to ≤Grade 2 fever.

Dinutuximab beta

Adverse event	Dose Modification of dinutuximab beta
Hypotension	• ≤ *Grade 2:* Slow infusion to 50% rate. Resume 100% rate once resolves.<br>• ≥ *Grade 3:* Interrupt infusion. If hypotension resolves or improves to ≤ Grade 2 with fluid boluses (e.g. 20 ml/kg sodium chloride 0.9% fluid challenge over 20-30 minutes), dinutuximab beta may be resumed at 50% rate. If this tolerated then increase to 100% rate following day as tolerated. If hypotension ≥ grade 3 recurs with 50% rate of dinutuximab beta, then discontinue dinutuximab beta and consider restarting the infusion the following day.<br>If hypotension not responsive to 20 ml/kg fluid challenge then discontinue dinutuximab beta infusion, support blood pressure with IV fluids, and vaso-pressors if necessary. Dinutuximab beta infusion may be restarted at 50% once patient stable without blood pressure support, and then increased to 100% rate if tolerated. **If patient requires ventilator support, patient should permanently discontinue dinutuximab beta.**
Neuropathy	• *Grade 1:* Continue infusion and close observation<br>• *Grade 2*: Pause dinutuximab beta. Restart at 50% if resolves to Grade 1 or less. If not resolved within 48 hrs then omit from current cycle. **If not resolved to Grade 1 by time next cycle due then permanently discontinue:**<br>• **≥ Grade 3: Permanently discontinue dinutuximab beta Consider high dose systemic steroids / IVIG (Discuss with the sponsor and CI).**
Pain	• ≥ *Grade 3* despite analgesia: pause dinutuximab beta infusion, resume at 50% rate once pain controlled. Increase to 100% rate as tolerated.
Visual acuity or ocular toxicity	• *Dilated pupils with sluggish responses:* interrupt infusion. Resume at 50% rate once resolves<br>• Impaired visual accommodation, correctable with eye glasses does NOT need dose modification, provided that these toxicities are judged to be tolerable by the responsible clinician, as well as the patient and family.<br>• **≥ *Grade 3:* permanently discontinue dinutuximab beta**<br>** Please note that accommodation disorders and opthalmoplegia have been described as common Adverse Events and do not require any dose modifications*
Hyponatraemia	• ≤ *Grade 2:* supportive measures. Continue 100% infusion rate<br>• *Grade 3:* Continue dinutuximab beta at 100% rate unless symptomatic hyponatraemia in which sodium < 125 mmol/L for more than 48 hours despite corrective measures<br>• ***Grade 4* (< 120 mmol/l despite appropriate corrective measures)*:* Permanently discontinue dinutuximab beta**
Dyspnoea	• ≤ *Grade 2:* Oxygen supplementation of needed to maintain oxygen saturations > 90%. Regular salbutamol +/- epinephrine nebulisers if bronchospasm or stridor. If saturations not maintained then stop dinutuximab beta infusion; restart at 50% rate if symptoms resolve (saturations > 90% in air) and then increased to 100% if tolerated.<br>• *Grade 3:* Stop dinutuximab infusion. If symptoms resolve with supportive measures as above, and saturations > 90% in air, then restart dinutuximab beta at 50% and then increase to 100% as tolerated

Dinutuximab beta

Adverse event	Dose Modification of dinutuximab beta
	• *Grade 4:* **Permanently discontinue dinutuximab beta**
Cardiac toxicity	• ≥ ***Grade 3:*** **Permanently discontinue dinutuximab beta**
GI symptoms (diarrhoea / vomiting)	• ≤ *Grade 3:* no dose reduction unless considered intolerable<br>• *Grade 4;* Pause dinutuximab beta infusion, restart infusion when symptoms settle.
Infection	• *In the case of any ≥ G3 infection occurring during dinutuximab beta infusion, dinutuximab beta should be omitted until infection is controlled. In the case of uncomplicated bacteraemia or febrile neutropenia in clinically stable children it may be then be restarted, but for any other ≥ G3 infections it should be aborted until the next cycle. Temperature alone, in the absence of other signs of infection, should not be considered a reason to stop dinutuximab beta*

10.5.2 Dose modifications for haematological toxicity

While it is known that dinutuximab beta alone can cause some degree of myelosuppression, if haematological toxicity appears in this study, it will be most likely related to chemotherapy. Hence, dose modifications for haematological toxicity include recommendations to reduce doses of chemotherapy only.

Table 20: Dose levels for dose adjustments in the dinutuximab beta randomisation

Drug	Starting dose	Reduction 20%<br>Dose level – 1	Reduction 40%<br>Dose level – 2
Arms T and dBT			
Temozolomide	200 mg/m^2/d for 5 days every 4 weeks	160 mg/m^2/d for 5 days every 4 weeks	120 mg/m^2/d for five days every 4 weeks
Arms TTo and dBTTo			
Temozolomide	150 mg/m^2/d for 5 days every 4 weeks	120 mg/m^2/d for 5 days every 4 weeks	90 mg/m^2/d for five days every 4 weeks
Topotecan	0.75 mg/m^2/d for 5 days every 4 weeks	0.6 mg/m^2/d for 5 days every 4 weeks	0.48 mg/m^2/d for 5 days every 4 weeks

Table 21: Dose reduction and discontinuation rules for AEs attributed to chemotherapy – All arms as applicable

Type of Toxicity	Dose modification at first occurrence	Dose modification at second occurrence
ANC < 0.75 x 10^9/L or<br>Platelet count < 75 x10^9/L<br>But recovered on day 28 after the start of a cycle	No dose modification	No dose modification

ANC < 0.75 x 10^9/L or<br>Platelet count < 75 x10^9/L<br>But recovered between day 29 to 35 after the start of a cycle	No dose reduction	Decrease temozolomide to dose level -1
ANC < 0.75 x 10^9/L or<br>Platelet count < 75 x10^9/L<br>But recovered between day 36 to 42 after the start of a cycle	Decrease temozolomide to dose level -1<br>Decrease topotecan to dose level -1	Decrease temozolomide to dose level –2<br>Decrease topotecan to dose level -2
ANC < 0.75 x 10^9/L or<br>Platelet count < 75 x10^9/L<br>On day 43 after the start of the cycle	Consider discontinuation of study treatment*	Not applicable

Note: All platelets cut-off values require no platelet transfusions within 72 hours of starting the cycle. All neutrophil cut-off values require being off G-CSF for at least 72 hours. For those patients with known bone marrow involvement, the cut-off values required are ANC ≥0.5 x10^9/L and platelets ≥50 x10^9/L

*If not recovered 15 days after expected date of start (i.e. day 43 after start of cycle), refer to Sponsor to discuss individual case

Haematological recovery thresholds needed to administer dinutuximab beta are lower than those needed for chemotherapy (neutrophils ≥ 0.5 x 10^9/l and platelets ≥ 20 x10^9/l in accordance with the SmPC). In case of hematological toxicity dinutuximab beta should be delayed until the whole course can be given concurrently with chemotherapy.

These dose modifications are provided as recommendations. In case an alternative management strategy is thought to be beneficial for the patient, the case will be discussed with the sponsor.

10.5.3 Dose modifications for hepatic toxicity

Table 22: Dose modifications for hepatic toxicity in the dinutuximab beta randomisation

	Dinutuximab beta		Topotecan		Temozolomide	
	1st occurrence	2nd occurrence	1st occurrence	2nd occurrence	1st occurrence	2nd occurrence
**Elevated ALT, AST, ALP, GGT, Bilirubin**<br>**≤ Grade 2**	No dose modification	No dose modification	No dose modification	No dose modification	No dose modification	No dose modification
**Elevated ALT, AST, ALP, GGT, Bilirubin**<br>**Grade 3 that recovers to grade ≤1 before day 35**	No dose modification	No dose modification	No dose modification	No dose modification	No dose modification	No dose modification
**Elevated ALT, AST, ALP, GGT, Bilirubin**	No dose modification	No dose modification	No dose modification	Discontinue study treatment	Decrease temozolomide to dose level -1	Discontinue study treatment

**Grade 3 not recovered to grade ≤1 before day 35**						
**Elevated ALT, AST, ALP, GGT, Bilirubin**<br>**Grade 4**	Reduce to 50% for cycle; can be increased to 100% for subsequent cycles providing improved to ≤ Grade 2	Reduce to 50% for cycle; can be increased to 100% for subsequent cycles providing improved to ≤ Grade 2	No dose modification	Discontinue study treatment	Decrease temozolomide to dose level -1	Discontinue study treatment

11 TREATMENT COMPLIANCE

Bevacizumab, dinutuximab beta, irinotecan and topotecan and cyclophosphamide will be administered intravenously and temozolomide will be given orally. Information regarding the dates for all drugs and doses of treatment administered will be recorded in the patient's medical records.

Patients and/or parents must be instructed to return any unused capsules of temozolomide if dispensed for home use and a pill count will be performed. Returns must be documented on the pharmacy drug accountability log. Sites should follow their local practice for destruction of unused capsules.

12 SUPPORTIVE TREATMENT

12.1 Nausea and Vomiting

Anti-emetics may be used, at the investigator's discretion, for the prevention and/or treatment of nausea and vomiting. Local anti-emetic policies for chemotherapy should be followed, when necessary.

Patients receiving dinutuximab beta could also receive prophylactic anti-emetics, for example for anticipated potential of intravenous morphine to cause nausea as per institutional policies.

12.2 Growth Factors

Primary prophylactic use of growth factors will not be permitted. Therapeutic usage or secondary prophylactic use of G-CSF should be discussed with the Sponsor on an individual case basis.

12.3 Fever and neutropenia

Antibiotics should be administered on the basis of institutional policy.

12.4 Blood products

Therapeutic use of blood products will be permitted. Patients should not be transfused platelets within the 72 hours prior to starting each cycle.

12.5 Pneumocystis jirovecii pneumonia (PJP) prophylaxis

PCP prophylaxis is mandatory for patients receiving temozolomide. Drugs and schedules for PJP prophylaxis should be according to institutional policies.

12.6 Management of side effects caused by non-selective NSAIDs as cyclooxygenase (COX) type I and II inhibitors

For patients receiving dinutuximab beta. Due to the potential side effects of non-selective NSAIDs as cyclooxygenase (COX) type I and II inhibitors and its effects on platelet aggregation (increased haemorrhagic risk, gastrointestinal mucosal injury): proton pump inhibitors (PPIs) or H2-receptor antagonists according to institutional use need to be considered.

12.7 Supportive care during Dinutuximab beta infusion

12.7.1 Pain Management

It is anticipated that patients receiving dinutuximab beta by the continuous infusion schedule will be able to be managed largely on an outpatient patient basis, with oral opioid or non-opioid medication providing adequate pain control. However, since neuropathic pain is an anticipated side effect even in a prolonged continuous infusion setting, all patients should receive premedication with gabapentin from at least 3 days prior to the start of the dinutuximab beta, as well as intravenous morphine prior to and during antibody infusions as required

Concomitant standard pain management should be established with or without IV opioid analgesia and should follow standard WHO guidelines including medications as follows:

- Gabapentin: Prior to receiving dinutuximab beta, the patient should be primed with oral gabapentin, starting 3 days prior to the start of the dinutuximab beta infusion. The recommended oral dose of gabapentin is 10 mg/kg/dose once daily on day 1, increasing to 10 mg/kg/dose twice daily on day 2 and 10 mg/kg three times a day thereafter. Gabapentin may either be stopped at the end of each continuous antibody infusion (and restarted 3 days prior to subsequent cycle) or continued throughout cycles and stopped after the end of the last infusion, according to local guidelines.

  Maximum single dose for Gabapentin as per institutional guidelines.

- Intravenous morphine: An intravenous opioid (e.g. morphine infusion 0.03 mg/kg/hr or equivalent) should be commenced 1-2 hour prior to starting the first dinutuximab beta infusion (Cycle 1). Thereafter, it is recommended to administer a continuous morphine (or alternative intravenous opioid at equivalent dose) infusion rate of up to 0.03 mg/kg/hour on the first day, with additional boluses as required (in accordance with local guidelines). Ideally intravenous morphine can be weaned off on a daily basis over the first 5 days (e.g. to 0.02 mg/kg/hour to 0.01 mg/kg/h to 0.005 mg/kg/hour). Boluses can then be given as required either self-administered or nurse administered at 0.02 mg/kg/dose. If continuous opioid is required for more than 5 days then the dose should be weaned at the end of treatment by 20% each day. It is expected that the IV opioid can be rapidly tapered off, depending on the individual patient's pain tolerance.

  Subsequent cycles may be started with intravenous morphine as above, depending on the amount of opioid required in the previous cycle, until a safe and well tolerated out-patient pain management regime is in place for the individual patient.

- Oral and transdermal opioids: e.g. oral morphine can be administered at a dose of 0.2 - 0.4 mg/kg every 4-6 hours. It is not advised to use long acting morphine in this situation as it takes 48 hours to stabilise the dose and probably is not useful. Oral tramadol may be considered once oral morphine is sufficient at lower doses to control pain satisfactorily. Alternatively transdermal application of fentanyl patches may be used in centres where fentanyl patches are regularly used for pain control. The equivalent transdermal fentanyl dose rate in µg/hr will be calculated from the current use of IV morphine according to the manufacturer's guidelines. This allows for pain management during continuous infusion of dinutuximab beta in an outpatient setting.

- Non-steroidal anti-inflammatory drugs (NSAIDs), used as per local guidelines, dosing and availability as a fixed regimen during antibody treatment. Check section 12.6 for prevention of toxicities derived from the use of COX I and II inhibitors. :

  - Paracetamol: Recommended dosages as per institutional guidelines

  - Ibuprofen: This is the most widely used NSAID in paediatrics. It is approved from age 6 months onwards and has a longer duration of action (6-8 hours). Recommended dosages as per institutional guidelines. Ibuprofen should be used according to local clinical practice. Caution must be taken if platelet count < 50 x 10^9/l due to increased risk of bleeding.

Dinutuximab beta

- Metamizole: Because of its spasmolytic properties metamizole is particularly suitable for visceral pain or colicky pain. More useful than repeated short infusions is a long-term infusion with a dosage of 2.5 to 3.0 mg/kg/h, always with close monitoring of blood pressure values. Metamizole is approved for use from the age of three months onwards. A risk assessment for agranulocytosis in children receiving metamizole therapy is not possible at present.

  Recommended dosage of Metamizole: 10 mg/kg PO every 6 hours as needed or long-term infusion with a dosage of 2.5 to 3.0 mg/kg/h. However, in some European countries, the use of Metamizole is not permitted in paediatric patients and this national guidance needs to be respected.

- Other NSAIDs, e.g. Indomethacin or Ketoralac, may be used as per institutional guidelines, as an alternative to ibuprofen if fever not controlled.

➢ Hyoscine-butyl-bromide or hyoscyamine may be used for children for abdominal pain not responding to morphine, as per institutional guidelines

12.7.2 Prevention of dinutuximab beta related infusion reactions

For anticipated potential of dinutuximab beta for allergic reactions, anti-histamines should be prescribed as per institutional guidelines. For example: diphenhydramine, hydroxyzine, cetirizine, or chlorphenamine. Corticosteroids should not be used as premedication for dinutuximab beta or as anti-emetics as they may reduce the efficacy of dinutuximab beta. Corticosteroids use should be limited to severe allergic toxicities that do not respond to stopping dinutuximab beta and antihistamines.

13 CONCOMITANT MEDICATION

Patients must be instructed not to take any additional medications, including over-the-counter (OTC) products and herbal remedies, during the study without prior consultation with their doctor (local PI). Palliative and supportive care for disease-related symptoms should be offered to all patients when appropriate. Relevant concomitant medications and blood products, as well as interventions (e.g., analgesic use, paracentesis) received by patients from screening until the end of the AE reporting period should be documented in the patient's medical notes.

No chemotherapy, hormonal anticancer therapy, or experimental anticancer medications other than those that are study-related will be permitted while the patient is receiving study treatment. In case of disease progression requiring withdrawal from study treatment, no further trial medications will be given and investigators should give whatever anti-tumour therapy is considered appropriate.

Caution must be taken for patients receiving bevacizumab if intravenous bisphosphonates are given simultaneously or sequentially.

Systemic (oral or intravenous) corticosteroids should be avoided except if needed as an emergency (e.g. anaphylaxis) but topical applications, inhaled sprays, eye drops or local injection (e.g., intraocular) of corticosteroids are allowed.

Treatment-related adverse events related to concomitant medication should be treated according to local practice.

Palliative radiotherapy to non-target lesions or to symptomatic target lesions in patients that have other target lesions that allow evaluation of response will be permitted.

All vaccinations (including influenza) should be avoided during administration of dinutuximab beta until 10 weeks after the last treatment course, due to immune stimulation through dinutuximab beta and possible risk for rare neurological toxicities.

14 ASSESSMENTS

All randomised patients receiving a single dose or more of study medications will be considered evaluable for toxicity. Safety endpoints include adverse events, clinical examination (including height, weight, blood pressure and pulse rate) and laboratory tests (haematology, chemistry, clotting). Safety laboratory tests should include full blood count and differential, biochemistry with sodium, potassium, calcium, urea, creatinine, total protein, albumin, bilirubin, alkaline phosphatase (ALK PHOS), LDH, ALT or AST. For the bevacizumab randomisation, INR and APTT also need to be measured, in addition to urine assessment for the detection of proteinuria. For the dinutuximab beta randomisation, chest x-ray has to be performed at screening and repeated if clinically indicated.

All study related procedures must be performed at the study site or at accredited centres/laboratories to which the study site refers patients for laboratory tests or imaging if these cannot be routinely performed at the study site.

In the case of additional investigations being performed at the patient's local hospital and not as part of study related procedures, it is the Investigator's responsibility to ensure he/she receives and reviews the results. The results must be recorded on the CRF as required, and the reports from the other hospitals must be available for source data verification. Laboratory reference ranges, including effective

dates, and evidence of laboratory accreditation must be obtained from all laboratories used. It is the Principal Investigators' responsibility at each site to obtain these.

14.1 Response assessment

Responses will be evaluated using the Response Evaluation Criteria in Solid Tumours (see Appendix 4 – RECIST Criteria 1.1) for those patients with measurable disease on cross-sectional imaging. All patients who have measurable disease will be considered evaluable for anti-tumour activity using standard RECIST criteria.

Response will be determined for patients with evaluable disease (only MIBG avid disease) using a semi-quantitative score (see Appendix 6 - CURIE & SIOPEN scoring methods for neuroblastoma) and the new International Neuroblastoma Response Criteria (Appendix 5 – Tumor Response at Metastatic Soft Tissue and Bone Sites (Park et al. 2017).

Patients who fail treatment early, prior to response evaluation, will be considered to be non-responders. Changes in tumour size will be categorised as Complete Response (CR), Partial Response (PR), Stable Disease (SD), Progressive Disease (PD – including the appearance of new lesions) or Non Evaluable (NE). For patients where disease is only evaluable by MIBG, with or without bone marrow disease, response will be described using INRC criteria. The Curie/SIOPEN score (see Appendix 6 - CURIE & SIOPEN scoring methods for neuroblastoma will be used to report MIBG scans at screening and after 2, 4 and 6 courses of treatment. Patients with MIBG negative scans but lesions on 18FDG-PET/CT scans will be evaluated with the same imaging modality. For evaluation of bone marrow involvement, bilateral iliac crest bone marrow aspirates and trephines will be performed at screening and repeated after 2, 4 and 6 courses if the prior assessment showed positivity or if clinical suspicion of BM involvement arises. CR in BM will be defined when 2 aspirates and 2 trephines are negative in a previously involved BM. PD will be defined when previously normal BM (at 4 sites) becomes involved (any of the 4 sites). The recently published international guidelines to evaluate response in the bone marrow component will be used at the time of the analysis (Burchill et al. 2017). As an additional measure of quality control, an independent blinded radiologist and nuclear medicine physician will review all CT/MRI and MIBG scans of patients who respond (CR or PR), along with a random sample of at least 20% of the non-responders. For the assessment of the primary endpoint, local assessment of RECIST or INRC will be used.

15 BIOMARKERS

15.1 Blood sampling safety

The amount of blood taken for biomarker studies will not exceed 3 ml/kg (below 5% of total blood volume) which has been established as a safe and ethically appropriate volume for children older than 12 months. Please refer to Laboratory Manual.

European Medicines Agency (EMA) recommendations for trial related blood loss (including any losses in the manoeuvre) in paediatric populations are that no more than 3% of total blood volume should be taken during a four week period and not more than 1% of total blood volume at a single time-point. At a total blood volume estimated at 80 to 90ml/kg body weight, this equates to 2.4mL to 2.7mL blood per kg body weight during a four week period, or 0.8mL to 0.9mL blood per kg at any one time.

These recommended volumes must not be exceeded for any patient. Please use the information below and in to ensure that the recommended volumes are adhered to.

Table 23: EMA Recommendations for Trial related Blood Loss

Weight (kg)	Total blood volume (mL)	1% blood volume (mL)*	3% total blood volume (mL)*
5	425	4	12
10	850	8	24
15	1275	12	36
20	1700	17	51
25	2125	21	63
30	2550	25	75
35	2975	29	87
40	3400	34	102
45	3825	38	114
50	4250	42	126
55	4675	46	138
60	5100	51	103
65	5525	55	165
70	5950	59	177
75	6375	63	186
80	6800	68	204

* Per individual, the trial-related blood loss (including any losses in the manoeuvre) should not exceed 3 % of the total blood volume during a period of four weeks and should not exceed 1 % at any single time.

Time-points for the biomarker assays have been aligned in order to minimise invasiveness and reduce the volume of dead space blood that is removed from the patient. Where possible, blood for haematology and biochemistry analysis should be taken at the same times as the assay sample points for the same reasons. Investigators must seek advice from the Coordinating Sponsor if there is a concern regarding the volume of study related blood loss for a particular patient.

The table below demonstrates the blood volumes required for the dinutuximab beta randomisation immune monitoring.

Table 24: BEACON dinutuximab beta amendment: Blood volumes for immune monitoring

Assay	Volume per assay	Day 1 Cycle 1	Day 8 Cycle 1	Day 1 Cycles 2, 4 & 6	Day 8 Cycles 2, 4 & 6	Day 1 Cycles 3 & 5	Day 8 Cycles 3 & 5
FcR & KIR genotype	2ml	x					
Dinutuximab beta PK & ADA	2ml	x	x	x	x	x	x
ADCC	6 ml *(12 ml)	x	x				
Immuno-phenotyping	2 ml	x	x			x	x
Total volume at time point		12 ml *(18ml)	10 ml *(16 ml)	2ml	2ml	4ml	4ml
Total volume in 4 week cycle		22ml *(34ml)		4 ml		8 ml	

* 12 ml if WBC < 2 x 10^9/l

15.2 MRI-derived functional imaging biomarkers of angiogenesis

Functional imaging scans will be performed in patients in all arms of the trial with measurable disease with the criteria:

- tumour measuring at least 2 cm in the minimum axial diameter at screening assessment
- tumour location not prone to motion artefacts (e.g. avoiding diaphragmatic surface, para-cardiac region and adjacent to major arteries)

Patients with metallic implants near the tumour or contraindications to MR imaging will be not eligible for functional imaging evaluation. Scanning will be performed at baseline; post 2, 4 and 6 cycles of trial treatment and at EOT if patient discontinues trial treatment early. Dynamic contrast enhanced sequences will be added to the conventional MRI procedure when the patient is having MRI as part of their disease assessment for those cases with measurable disease by RECIST criteria. The addition of this sequence will increase scanner time by 15 minutes and it is believed that this will not pose an additional risk to the patients. Functional imaging will be limited to specialised centres with the capability to perform these MR methods in children. Data will be collected and analysed centrally.

15.3 Molecular monitoring mRNA

Please refer to the current BEACON-Neuroblastoma Laboratory Manual before taking samples. Blood (1 x 2ml) and bone marrow (BM) aspirate (2 x 0.5ml, one from each side, not pooled) samples will be collected at baseline, and at post cycles 2, 4 and 6 of trial treatment. For those patients receiving bevacizumab, at least 48 hours **must** elapse from any bone marrow sampling and any bevacizumab infusion. Samples will be stored in PAXgene™ blood RNA tubes at -80°C until being transported in batches to St James University Hospital, Leeds for storage until analysis. If there are surplus sample to the study requirements, these will be registered in the Children's Cancer and Leukaemia Group (CCLG) Biobank and made available for additional future research.

15.4 Neuroblastoma exploratory biomarker analyses

Please refer to the current BEACON-Neuroblastoma Laboratory Manual for further details. Collected samples include:

- Archival tumour: an archived formalin fixed tumour tissue embedded in paraffin (FFPE) block will be submitted for each patient if available (unstained slides or fresh frozen tissue might be acceptable). Any tumour blocks not utilised will be returned to the site. In the event that the entire tumour block cannot be obtained, a partial block may be submitted. Patients where archival tumour is not available must be discussed with the Coordinating Sponsor/CI before starting study medications.
- Blood sample for neuroblastoma exploratory biomarker analyses: a blood sample should be taken at baseline (screening), post cycle 2 (prior to commencing cycle 3), at post cycle 4 (prior to commencing cycle 5) and at either post cycle 6 or EOT if patient discontinues trial treatment early . A 5ml blood sample is preferred at each time point, however a minimum of 2.5ml is acceptable.
- Blood sample for constitutional DNA analysis: a 2ml blood sample will also be taken at baseline for constitutional DNA analysis for those patients that provide additional informed consent. Samples for constitutional DNA analyses are optional. This will be used to confirm the somatic nature of the genetic alterations detected in the tumour DNA.

Exploratory biomarkers will include analyses performed in blood and tumour samples related to the biology of neuroblastoma and identifying potential markers of prognostic or response to new therapies. These may include, but are not limited to:

- Immunohistochemistry in archival tumour samples
- Tumour molecular profiling - gene expression and DNA sequencing will be performed to explore possible associations between candidate molecular predictors and clinical outcome.
- Mutant circulating DNA - mutant circulating DNA for driver mutations will be assessed as a potential surrogate marker for tumour burden or treatment efficacy.
- *Dinutuximab beta pharmacokinetics (PK) and Anti-Drug Antibody (ADA) levels* - baseline, peak and trough Dinutuximab beta levels will be measured during chemo-immunotherapy. Human Anti-chimera Antibodies (HACA) will be measured prior to each cycle of chemo-immunotherapy, using established ELISA methodology (Siebert, Eger, et al. 2016), to assess for the development of ADA.
- *Immunophenotyping and ADCC* - The effects of chemo-immunotherapy on the number of circulating (peripheral blood) immune cells will be assessed by immunophenotyping. Functional activity of immune cells will be measured using established Antibody Dependent Cellular Cytotoxicity (ADCC) assays (Siebert et al. 2014), to quantify anti-GD2-mediated immune effector response.
- *Fcgamma receptor and KIR/KIR ligand* - Previous studies have correlated Fcgamma receptor (FcgR) genotype and KIR/KIR receptor repertoire with response to Dinutuximab beta, with more favourable response in patients with high affinity FcgR2A and FcgR3A and activating KIR (e.g. KIR 2DS2)(Siebert, Jensen, et al. 2016). PCR for FCGR2A high- and low-affinity alleles (131H and 131R) and FCGR3A high- and low-affinity alleles (158V and 158F), respectively, and genotyping of KIR/KIR ligands, will therefore be carried out on all patients using established methodology. Genotypes will be correlated with outcome and with immune effector function (ADCC).

Exploratory biomarker analyses on blood samples will include identifying potential markers of prognosis or response to new therapies, and the biology of neuroblastoma. This will help better understand the pathogenesis, course and outcome of childhood neuroblastoma.

After the described analyses, surplus material from tumour samples, extracted DNA or RNA will be stored at The Institute of Cancer Research (ICR) tissue bank, Sutton, UK, for up to 10 years after the end of the study at which time they will be destroyed. If required by regulatory authorities, however, specimens may be maintained for a longer period.

15.5 Sample Collection

For details on sampling procedures, sample storage, and shipment refer to the Laboratory Manual. All samples (except the blood sample for constitutional DNA) are part of the study and therefore are mandated. Not all sample collections will be ongoing at any one time. See BEACON-Neuroblastoma Lab Manual for current sample collections.

15.5.1 Peripheral blood samples

15.5.1.1 Molecular monitoring mRNA

Peripheral blood samples (2 ml) for measurement of circulating neuroblastoma mRNA should be taken from all patients at the following time points:

- baseline
- post cycle 2 (before commencing cycle 3)
- post cycle 4 (before commencing cycle 5)
- post cycle 6 (before commencing cycle 7)
- EOT if the patient discontinues trial treatment early

Immediately after collection, samples should be transferred into PAXgene™ blood RNA tubes.

15.5.1.2 Neuroblastoma exploratory biomarkers

Please refer to the current BEACON-Neuroblastoma Laboratory Manual for further details.

A blood sample (5ml/sample preferred, but at least 2.5ml/sample) for Neuroblastoma exploratory biomarkers will be taken from all patients at the following time points:

- baseline
- post cycle 2 (before commencing cycle 3)
- post cycle 4 (before commencing cycle 5)
- post cycle 6 (before commencing cycle 7)
- EOT if the patient discontinues trial treatment early

15.5.1.3 Constitutional DNA analysis biomarker

A 2ml blood sample will be taken at baseline for constitutional DNA analysis for those patients that provide additional informed consent.

For details on sampling procedures, sample storage, and shipment, please refer to the Laboratory Manual.

15.5.2 Bone Marrow Samples

15.5.2.1 Molecular monitoring mRNA

Please refer to current BEACON-Neuroblastoma Laboratory Manual before taking samples. Bone marrow aspirates (right and left iliac crest (2 x 0.5ml, one from each side, not pooled)) for the measurement of circulating neuroblastoma mRNA should be taken from all patients at baseline.

15.5.3 Archival tumour samples

15.5.3.1 Neuroblastoma exploratory biomarkers

Patients or their parents will be asked to give consent to retrieve any previously stored tumour sample (which could be at different hospitals) and any future samples collected during the course of the study treatment or follow up. Tumour tissue obtained at the time of relapse is especially valuable and should be obtained if ethically and clinically indicated. If a biopsy was performed or, if it is not possible to send

FFPE tumour tissue blocks, unstained, uncovered slides must be sent for analyses instead (submission of fresh frozen tumour tissue can be acceptable as well). After completion of biomarker analysis, any tumour blocks not utilised will be returned to sites where requested. Surplus material, extracted DNA or RNA will be stored at The Institute of Cancer Research (ICR) tissue bank, Sutton, UK. Please refer to the Laboratory Manual for further details.

16 PATIENT FOLLOW UP

A follow up visit will be scheduled 90 ± 15 days after the last dose of trial treatment, unless the patient withdraws consent or receives another treatment. Patients that develop relapse or start further therapy will not undergo further follow up visits, however, a Follow Up Form should still be completed and returned at the required time points detailing the patient's current disease and survival status. For those patients that remain relapse-free and do not start any further therapy, follow up visits will be done 3-monthly for one year and 6-monthly for up to 5 years. Additional follow up visits will be scheduled as needed, to monitor any sustained unresolved, treatment emergent adverse events. The first date of new anti-cancer treatment will be recorded.

For those patients that complete six courses of treatment and have not developed PD, further therapy up to 12 cycles could be considered by the local investigator after discussion with the Coordinating Sponsor and the CI. Patients who are randomised to receive dinutuximab beta and who continue past 6 courses of trial treatment will be allowed to continue for an additional 6 courses on chemotherapy **only.**

- In the event of progression/relapse, the type of progression/relapse will be recorded (local/metastatic/combined) as well as the new anticancer treatment that is started.
- Record vital signs, physical exam, and collect blood samples for the safety lab tests (full blood count and differential, biochemistry and clotting), including urinalysis, at each follow up visit.

16.1 Patient Withdrawal

Patients may withdraw from study treatment at any time at their own request or at the request of their parents (if patient under 16 years of age), or they may stop study treatment at any time at the discretion of the treating Investigator or CI for safety, behavioural, or administrative reasons. If a patient does not return for a scheduled visit, every effort should be made to contact the patient. In any circumstance, every effort should be made to document patient outcome where possible.

The Investigator should:

- Enquire about the reason for withdrawal
- Request the patient return for a final visit, if applicable
- Follow up with the patient regarding any unresolved adverse events
- Perform a physical exam on the patient (including height, weight and blood pressure/pulse)
- Arrange for a tumour assessment and all safety labs (full blood count, biochemistry and clotting bloods) to be collected

Patients who withdraw from trial treatment will continue to be followed up as per the protocol for at least 5 years after registration.

If the patient withdraws from study treatment, and also withdraws consent please refer to section 16.1.1.1 below.

16.1.1.1 Withdrawal of Consent

Patients/parents/legal guardians may withdraw consent at any time during the trial. The details of the withdrawal should be clearly documented and communicated to the BEACON-Neuroblastoma Trial Office.

There are three types of withdrawal as detailed below:

- Patient or their parent(s)/legal guardian would like to withdraw the patient from the trial, but is willing for the patient to be followed-up according to the trial schedule (follow-up data can be collected and used in the trial analysis)
- Patient or their parent(s)/legal guardian does not wish for the patient to attend trial follow-up visits but is willing for the patient to be followed-up at standard clinic visits (follow-up data can be collected at standard clinic visits and used in the trial analysis)
- Patient or their parent(s)/legal guardian is not willing for the patient to be followed up for trial purposes at any further visits (any data collected prior to the withdrawal of consent can be used in the trial analysis)

The following should be clearly documented in the medical notes:

- The date the patient or their parent(s)/legal guardian withdraw consent.
- The reason, if given (e.g. toxicity to drug).
- Type of withdrawal

If a patient withdraws from the trial, please complete and return a Treatment Discontinuation Form and a Withdrawal of Consent Form if applicable.

17 PHARMACEUTICAL INFORMATION

17.1 Definition of Investigational Medicinal Product

In this clinical trial bevacizumab, dinutuximab beta, irinotecan, temozolomide, topotecan and cyclophosphamide are all considered to be Investigational Medicinal Products.

17.2 Bevacizumab

17.2.1 Bevacizumab - Drug Supply

Bevacizumab will be supplied by F. Hoffmann-La Roche Ltd and distributed directly to sites by F. Hoffmann-La Roche Ltd.

17.2.2 Bevacizumab - Ordering

For the drug ordering process, please see the BEACON-Neuroblastoma Pharmacy Manual.

17.2.3 Bevacizumab - Formulation, Packaging and Labelling

Bevacizumab, a recombinant humanised monoclonal antibody (rhuMAb VEGF), is produced by recombinant DNA technology in a Chinese hamster ovary mammalian cell expression system in a nutrient medium containing the antibiotic gentamycin and is purified by a process that includes specific viral inactivation and removal steps. Gentamycin is detectable in the final product at 0.35 ppm. Bevacizumab consists of 214 amino acids and has a molecular weight of approximately 149,000 Daltons. Bevacizumab is supplied as a clear to slightly opalescent, colourless to pale brown, sterile liquid for intravenous (IV) infusion in single-use vials that are preservative free. Bevacizumab will be supplied as a 25-mg/mL concentrate for solution for infusion in glass vials. The formulation contains sodium phosphate, trehalose, polysorbate 20, and Sterile Water for Injection (SWFI) in addition to the active ingredient, bevacizumab. The study drug will be labelled in the local language according to the regulatory requirements in each country, as well as in accordance with Good Clinical Practice (GCP). **Commercial supply of bevacizumab SHOULD NOT be used in any study patient.** Vials are shipped in cooled validated containers. Upon receipt of the study drug, vials are to be refrigerated at +2°C − +8°C and should remain refrigerated until just prior to use. DO NOT FREEZE. DO NOT SHAKE. Protect from light. Temperature logs must be maintained (in accordance with local pharmacy practice) to ensure

proper storage conditions. Do not use medication beyond the expiration date stamped on the vial. If a temperature deviation from the allowed 2°C−8°C is found either during shipment or storage, please contact the Coordinating Sponsor to determine whether drug is still suitable for use. VIALS ARE FOR SINGLE USE ONLY. Vials used for one patient must not be used for any other patient. Vials must not be used after the re-test date shown on the pack.

17.2.4 Bevacizumab - Preparation and Dispensing

Bevacizumab should be diluted prior to infusion according to locally validated practice and/or procedures for the aseptic preparation, with particular reference to the preparation of agents that are to be administered as Investigational Medicinal Products (IMPs) in a clinical trial.

Such practice and/or procedures should also reflect the published data on the compatibility of bevacizumab with the intended container, the chemical stability of bevacizumab with the proposed diluent for infusion at the intended final concentrations of the prepared infusion and the appropriate storage conditions consequent on the choices made. A note should be made in the Pharmacy File which documents:

- The relevant practices/procedures with version control, updated as appropriate during the duration of the trial,
- The method of preparation of the infusion with respect to the container, diluent, concentration and any other relevant information.
- The storage conditions applied to the prepared infusion.

17.2.5 Compatibility information

The following is provided for information. Sites may use this information in order to prepare and/or dispense bevacizumab but may alternatively use other locally validated information, as above. The practice adopted should be documented in the Pharmacy File.

Bevacizumab does not contain any antimicrobial preservative. It is compatible with sodium chloride 0.9% solution for IV infusion in a concentration range of 1.4 - 16.5mg/ml. The preferred final volume of the infusion is 100ml. but the final volume should be changed as necessary according to the required dose to ensure that the concentration is within the range stated. After reconstitution, bevacizumab is chemically and physically stable in sodium chloride 0.9% for 48 hours at 2° - 30°C. No incompatibility between bevacizumab and polyvinylchloride or polyolefin containers has been observed. Bevacizumab is incompatible with glucose solutions. It is recommended that bevacizumab be administered within 8 hours after preparation; if this is not feasible, in-use storage times and conditions are the responsibility of the user and would normally not be longer than 24 hours at 2° - 8°C, unless dilution has taken place in controlled and validated aseptic conditions.

17.2.6 Bevacizumab - Administration

Bevacizumab should be administered as a continuous IV infusion using a rate–regulating device. It should not be administered as a bolus. The first bevacizumab infusion should be delivered over a minimum of 90 ± 15 minutes. If no adverse reactions occur, the second dose should be administered over a minimum of 60 ± 15 minutes. If no adverse reactions occur after the second administration, all subsequent doses should be administered over a minimum of 30 ± 15 minutes. Refer to Table 13: Bevacizumab - Infusion-related reaction/infusional site extravasation management guidelinesfor management guidelines in the event that an infusion-related reaction, allergic reaction, or infusional site extravasation occur. To ensure the complete delivery of bevacizumab, the IV infusion line must be flushed with sodium chloride 0.9%. It is recommended to complete the IV infusion with an additional 50ml of 0.9% sodium chloride for injection. **Note**: The additional volume used to flush the IV infusion line is not included in the recommended infusion times.

17.2.7 Bevacizumab – Accountability

F. Hoffmann-La Roche Ltd will provide bevacizumab once site agreements have been signed and institutional approval has been received by the BEACON-Neuroblastoma Trial Office (UK). Upon receipt the bevacizumab needs to be unpackaged and stored at 2-8°C.

The Investigator, or an approved representative (e.g. pharmacist), will ensure that all study bevacizumab is stored in a secured area, under recommended storage conditions and in accordance with applicable regulatory requirements.

To ensure adequate records, all study drugs must be accounted for on the drug accountability inventory forms, as instructed by the University of Birmingham. Used or partially used vials can be destroyed at the site and accurate records must be kept and made available to BEACON-Neuroblastoma trial personnel on request (see 17.2.8). Unless otherwise authorised by BEACON-Neuroblastoma trial personnel, at the end of the clinical study all bevacizumab supplies unallocated or unused by the patients must be destroyed according to the centres own practice and accountability logs completed accordingly.

IMP traceability must be employed at sites for irinotecan, temozolomide and topotecan starting at the point of dispensing to the patient. Full details are provided in the BEACON-Neuroblastoma Pharmacy Manual.

17.2.8 Bevacizumab - Destruction

Local or institutional regulations may require that, for safety reasons, partially used IMP be immediately destroyed. This is allowed on the condition that source document verification has been performed on the remaining inventory and reconciled with the documentation of the quantity shipped, dispensed, returned, and destroyed. Written documentation of any IMP that has been destroyed must be available for monitoring inspection and contain the following:

- Identity (batch numbers or patient numbers) of IMP destroyed
- Quantity of IMP destroyed
- Date of destruction
- Method of destruction
- Name and signature of responsible person (or company) who destroyed IMP

17.3 Cyclophosphamide

Cyclophosphamide will be obtained from hospital stock. Details regarding formulation, packing and labelling, preparation, dispensing and administration can be found in the BEACON - dinutuximab beta amendment pharmacy manual.

17.4 Dinutuximab beta

Dinutuximab beta will be supplied by EUSA Pharma free of charge for this trial. Details regarding f, packing and labelling, preparation, dispensing, administration, destruction and accountability can be found in the BEACON - dinutuximab beta amendment pharmacy manual.

17.5 Irinotecan

17.5.1 Irinotecan - Drug Supply

Irinotecan will be obtained from hospital stocks.

17.5.2 Irinotecan - Formulation, Packaging and Labelling

The intravenous formulation of irinotecan (Irinotecan hydrochloride trihydrate) is available commercially in 20 mg/ml vials.

17.5.3 Irinotecan - Preparation and Dispensing

The total administered dose of chemotherapy may be rounded up or down to the nearest dose that can be accurately measured. Irinotecan should be diluted prior to infusion according to locally validated practice and/or procedures for the aseptic preparation of chemotherapy agents, with particular reference to the preparation of chemotherapy agents that are to be administered as Investigational Medicinal Products in a clinical trial.

Such practice and/or procedures should also reflect the published data on the compatibility of irinotecan with the intended container, the chemical stability of irinotecan with the proposed diluent for infusion at the intended final concentrations of the prepared infusion and the appropriate storage conditions consequent on the choices made. A note should be made in the Pharmacy File which documents:

- The relevant practices/procedures with version control, updated as appropriate during the duration of the trial,
- The method of preparation of the infusion with respect to the container, diluent, concentration and any other relevant information.
- The storage conditions applied to the prepared infusion.

17.5.4 Compatibility information

The following is provided for information. Sites may use this information in order to prepare and/or dispense irinotecan but may alternatively use other locally validated information, as above. The practice adopted should be documented in the Pharmacy File.

Irinotecan is compatible with glucose 5% and sodium chloride 0.9% solutions for IV infusion in a concentration range of 0.12 - 2.8 mg/ml. After reconstitution, irinotecan is physically and chemically stable in the stated solutions for infusion for up to 28 days when stored in LDPE or PVC containers at 5ºC or at 30ºC and protected from light.

17.5.5 Irinotecan - Administration

For intravenous administration only. Infuse over 60 minutes.

17.6 Temozolomide

17.6.1 Temozolomide – Drug Supply

Temozolomide will be obtained from hospital stocks.

17.6.2 Temozolomide - Formulation, Packaging and Labelling

Temozolomide is available commercially as capsules for oral administration. Temozolomide contains lactose. Each capsule will contain 5, 20, 100, 140, 180 or 250 mg of the active ingredient temozolomide. Temozolomide must be stored protected from light. Do not store above 30°C. If a capsule becomes damaged, avoid contact of powder contents with skin or mucous membrane. If contact does occur, wash the affected area.

17.6.3 Temozolomide - Administration

The total administered dose of temozolomide should be rounded to the nearest 5mg as per the guidelines in Appendix 7 – Temozolomide Dosing. Capsules should be taken orally and not chewed. Keep capsules out of reach and sight of children, preferably in a locked cupboard. Capsules should ideally be administered on an empty stomach, with a minimum 2 hour fasting period pre-dose and for up to 1 hour post administration. If patients are unable to swallow capsules whole, the capsules may either be left to soften in fruit juice/apple sauce or opened and the contents mixed with fruit juice or apple sauce. Local guidelines already in place may be used provided they follow the principles set out below):

1. If prescribed, give a dose of anti-sickness medicine 30 minutes before each dose of temozolomide.
2. Working over a medicine cup, hold the capsule by each end gently twisting and pulling the capsule open before emptying the contents into the medicine cup. **Take care not to inhale any powder.**

3. Repeat step 2 for each capsule.
4. Carefully mix the powder into the juice using an oral syringe and then draw up all the liquid into the oral syringe.
5. Place the syringe into the patient's mouth, preferably inside a cheek and slowly push the plunger. If preferred the patient can drink the liquid directly from the medicine cup.
6. If you are using apple sauce, mix the powder with the sauce thoroughly and feed it to the patient using the medicine spoon.
7. Add some more fruit juice or apple sauce to the medicine cup and mix. Ensure that all the fruit juice or the apple sauce used to mix the powder is given to ensure the entire dose has been given. Repeat if any powder is still visible in the medicine cup.

An information sheet for parents has been produced detailing the above steps if temozolomide is to be taken at home.

If the patient vomits within 20 minutes after taking the dose, and the capsules are visible in the vomit, the dose should be re-administered and a note should be made in the patient's notes and on the accountability log.

17.7 Topotecan

17.7.1 Topotecan - Drug Supply

Topotecan will be obtained from hospital stocks.

17.7.2 Topotecan - Formulation, Packaging and Labelling

The intravenous formulation of topotecan (topotecan hydrochloride) is available commercially in 1mg and 4mg vials, as either a powder for reconstitution or a solution containing 1mg. in 1ml.

17.7.3 Topotecan - Preparation and Dispensing

The total administered dose of chemotherapy may be rounded up or down to the nearest dose that can be accurately measured. Topotecan should be reconstituted and further diluted prior to infusion according to locally validated practice and/or procedures for the aseptic preparation of chemotherapy agents, with particular reference to the preparation of chemotherapy agents that are to be administered as Investigational Medicinal Products in a clinical trial.

Such practice and/or procedures should also reflect the published data on the compatibility of topotecan with the intended container, the chemical stability of topotecan with the proposed diluent for infusion at the intended final concentrations of the prepared infusion and the appropriate storage conditions consequent on the choices made. A note should be made in the Pharmacy File which documents:

- The relevant practices/procedures with version control, updated as appropriate during the duration of the trial,
- The method of preparation of the infusion with respect to the container, diluent, concentration and any other relevant information.
- The storage conditions applied to the prepared infusion.

17.7.4 Topotecan - Compatibility information

The following is provided for information. Sites may use this information in order to prepare and/or dispense topotecan but may alternatively use other locally validated information, as above. The practice adopted should be documented in the Pharmacy File.

Topotecan is compatible with glucose 5% and sodium chloride 0.9% solutions for IV infusion in a concentration range of 0.025 – 0.05 mg/ml (25 – 50 microgram/ml). After reconstitution, the topotecan infusion must be completed within 12 hours if stored at room temperature or 24 hours if stored between 2 - 8°C.

17.7.5 Topotecan - Administration

For intravenous administration only. Infuse over 30 minutes.

18 ADVERSE EVENT REPORTING

The collection and reporting of Adverse Events (AEs) will be in accordance with the Medicines for Human Use Clinical Trials Regulations 2004 and its subsequent amendments, or the equivalent appropriate legislation in the participating member state. Definitions of different types of AE are listed in Appendix 2 - Definition of Adverse Events. The Investigator should assess the seriousness and causality (relatedness) of all AEs experienced by the patient (this should be documented in the source data) with reference to the Investigator Brochure or Summary of Product Characteristics as appropriate.

18.1 Reporting Requirements

18.1.1 Adverse Events (AE)

All medical occurrences which meet the definition of an AE (see Appendix 2 - Definition of Adverse Events) should be recorded.

Please note this does not include abnormal laboratory findings. An abnormal laboratory value is only considered to be an AE if the abnormality:

- Results in patient early discontinuation from the study treatment
- Requires treatment, modification or interruption of dose, or any other therapeutic intervention, or is judged to be of significant clinical importance

If a laboratory abnormality is one component of a diagnosis or syndrome, then only the diagnosis or syndrome should be recorded.

18.1.2 AESIs of Bevacizumab

AE of Special Interest (AESIs) are adverse drug reactions observed across clinical trials in patients who have received bevacizumab in combination with different chemotherapy regimens. The AESI defined in this protocol include:

- Hypertension
- Proteinuria
- Wound healing complication
- Bleeding/haemorrhage
- Venous thromboembolic events
- Arterial thromboembolic events
- Fistula
- Gastrointestinal perforation
- Congestive heart failure
- Posterior Reversible Encephalopathy Syndrome (PRES)

The purpose for specifying these AESI is to enable further characterisation of the clinical course and the management of these events. An AESI may or may not be the consequence of treatment with bevacizumab.

18.1.3 Serious Adverse Advents (SAE)

Investigators should report AEs that meet the definition of an SAE (see Appendix 2 - Definition of Adverse Events)

18.1.3.1 Events that do not require reporting on a Serious Adverse Event Form

The following events should not be reported on an SAE Form:

- Progression or death as a result of the patient's cancer, as this information is captured elsewhere on the CRF
- Hospitalisations for:
  - Protocol defined treatment
  - Pre-planned elective procedures unless the condition worsens
  - Treatment for progression of the patient's cancer

The following events should be **reported on an Expected SAR Form** rather than an SAE Form:

- Admissions to control symptoms of vomiting unless the condition is life threatening or proves fatal
- Admissions for supportive treatment (e.g. transfusions or treatment of non-complicated febrile neutropenia) during an episode of myelosuppression unless this proves fatal or requires admission to a high dependency or intensive care facility

SAR Forms should be completed promptly on resolution of the event and returned as follows:

UK and Ireland

- In the post: **BEACON-Neuroblastoma Trial Office, CRCTU, University of Birmingham, Vincent Drive, Edgbaston, Birmingham, B15 2TT United Kingdom**

Other participating countries:

- In the post: National Co-Sponsor

18.1.3.2 Monitoring pregnancies for potential Serious Adverse Events

It is important to monitor the outcome of pregnancies of patients in order to provide SAE data on congenital anomalies or birth defects. In the event that a patient or their partner becomes pregnant during the SAE reporting period please complete a Pregnancy Notification Form (providing the patient's details) and return to the BEACON-Neuroblastoma Trial Office as soon as possible. If it is the patient who is pregnant provide outcome data on a follow-up Pregnancy Notification Form. Where the patient's partner is pregnant, consent must first be obtained and the patient should be given a Pregnancy Release of Information Form give to their partner. If the partner is happy to provide information on the outcome of their pregnancy they should sign the Pregnancy Release of Information Form. Once consent has been obtained, details of the outcome of the pregnancy must be provided on a follow-up Pregnancy Notification Form. If appropriate, also complete an SAE Form as detailed below.

18.1.4 Reporting period

Details of all reportable AEs (except those listed in 18.1.1 and 18.1.3.118.1.3.1) will be documented and reported from the date of commencement of protocol defined treatment until 30 days after the administration of the last treatment.

SAEs that are judged to be at least possibly related to the IMPs must still be reported in an expedited manner irrespective of how long after IMP administration the reaction occurred.

18.2 Reporting Procedure

18.2.1 Site

18.2.1.1 Adverse Events

AEs should be reported on an AE Form (and where applicable on a paper SAE Form). An AE Form should be completed for each visit.

AEs will be reviewed using the Common Terminology Criteria for Adverse Events (CTCAE), version 4.0 (see Appendix 3 - Common Toxicity Criteria Grading). Any AEs experienced by the patient but not included in the CTCAE should be graded by an Investigator and recorded on the AE Form using a scale

of (1) mild, (2) moderate or (3) severe. For each sign/symptom, the highest grade observed since the last visit should be recorded.

An abnormal laboratory value should only be reported if it:

- Results in patient early discontinuation from the study treatment
- Requires treatment, modification or interruption of dose, or any other therapeutic intervention, or is judged to be of significant clinical importance

If a laboratory abnormality is one component of a diagnosis or syndrome, then only the diagnosis or syndrome should be recorded.

18.2.1.2 Serious Adverse Events

For more detailed instructions on SAE reporting refer to the SAE & Expected SAR Form Completion Guidelines contained in the Investigator Site File (ISF).

AEs defined as serious and which require reporting as an SAE (excluding events listed in section 18.1.3.1) should be reported on an SAE Form. When completing the form, the Investigator will be asked to define the causality and the severity of the AE which should be documented using the CTCAE version 4.0.

SAEs that are judged to be at least possibly related to the IMP(s) must still be reported in an expedited manner irrespective of how long after IMP administration the reaction occurred.

On becoming aware that a patient has experienced an SAE, the Investigator (or delegate) must complete, date and sign an SAE Form. The form should be faxed together with a SAE Fax Cover Sheet to the BEACON-Neuroblastoma Trial Office using one of the numbers listed below as soon as possible and no later than 24 hours after first becoming aware of the event:

To report an SAE, fax the SAE Form with an SAE Fax Cover Sheet to:
BEACON-Neuroblastoma Trial Office
🖷 +44 (0)121 414 9520 or +44 (0)121 414 3700

If reporting by fax is not possible, please send to:

Reg@trials.bham.ac.uk
Include "BEACON_Neuroblastoma SAE" in the subject line

On receipt the BEACON-Neuroblastoma Trial Office will allocate each SAE a unique reference number. This number will be transcribed onto the SAE Fax Cover Sheet which will then be faxed back to the site as proof of receipt. If confirmation of receipt is not received within 1 working day please contact the Trial Office. The SAE reference number should be quoted on all correspondence and follow-up reports regarding the SAE. The SAE Fax Cover Sheet completed by the Trial Office should be filed with the SAE Form in the ISF.

For SAE Forms completed by someone other than the Investigator, the Investigator will be required to countersign the original SAE Form to confirm agreement with the causality and severity assessments. The form should then be returned to the Trial Office by fax and the original copy kept in the ISF. Investigators should also report SAEs to their own Trust in accordance with local practice.

18.2.1.3 Provision of follow-up information

Patients should be followed up until resolution or stabilisation of the event. Follow-up information should be provided on a new SAE Form (refer to the SAE & Expected SAR Form Completion Guidelines for further information).

18.2.2 Trial Office

On receipt of an SAE Form, seriousness and causality will be determined independently by a Clinical Coordinator. An SAE judged by the Investigator or Clinical Coordinator to have a reasonable causal relationship with the trial medication will be regarded as a Serious Adverse Reaction (SAR). The Clinical Coordinator will also assess all SARs for expectedness. If the event meets the definition of a SAR that is unexpected (i.e. is not defined in the Reference Safety Information (taken from the Summary of Product Characteristics) for any patient group) it will be classified as a Suspected Unexpected Serious Adverse Reaction (SUSAR).

18.2.3 Reporting to the Competent Authority and main Research Ethics Committee

18.2.3.1 Suspected Unexpected Serious Adverse Reactions (SUSAR)

The Trial Office will report a minimal data set of all individual events categorised as a fatal or life threatening SUSAR to National Competent Authorities and main National Ethics Committee within 7 days. Detailed follow-up information will be provided within an additional 8 days. All other events categorised as SUSARs will be reported within 15 days.

18.2.3.2 Serious Adverse Reactions (SAR)

The UK Coordinating Centre will include details of all SAEs, SARs (including SUSARs) in a Development Safety Update Report (DSUR) produced annually from the date of the first Clinical Trial Authorisation received for the trial to the submission of the End of Trial Declaration. The National Coordinating Centres will be responsible for forwarding this report to the relevant Competent Authority and REC. The UK Coordinating Centre will report to the MHRA and UK REC.

Adverse Events (AE)

Details of all AEs will be reported to the Competent Authorities on request.

18.2.3.3 Other safety issues identified during the course of the trial

The Competent Authorities and National Ethics Committees will be notified immediately if a significant safety issue is identified during the course of the trial.

18.2.4 Investigators

Details of all SUSARs and any other safety issue which arises during the course of the trial will be reported to Principal Investigators. A copy of any such correspondence should be filed in the ISF.

18.2.5 Data Monitoring Committee

The independent Data Monitoring Committee (DMC) will review all SAEs. All SUSARs and SARs that are life-threatening or result in death will be reviewed.

18.2.6 Manufacturer of Investigational Medicinal Product

CRCTU will report all SAEs for patients randomised to a bevacizumab arm to F. Hoffmann-La Roche Ltd, manufacturer of bevacizumab in accordance with the Safety Data Exchange agreement.

19 DATA HANDLING AND RECORD KEEPING

19.1 Data Collection

This trial will use an electronic remote data capture (eRDC) system which will be used for completion of CRFs. Access to the eRDC system will be granted to individuals via the BEACON-Neuroblastoma Trial Office. Paper CRFs may be used initially before the eRDC is live.

SAE reporting will be entirely paper-based throughout the course of the trial.

CRFs must be completed (and for the paper SAE Form signed/dated and faxed to the Trial Office) by the Investigator or an authorised member of the site research team (as delegated on the Site Signature and Delegation Log) within the timeframe specified by the Coordinating Sponsor. The exception is the SAE Form which must be co-signed by the Investigator (this must be done as soon as possible but can be done after the form has already been faxed to the Trial Office so as not to delay initial reporting). See Adverse Event reporting section 18.2 for further details.

Data reported on each form should be consistent with the source data. If information is not known, this must be clearly indicated on the form. All missing and ambiguous data will be clarified. All sections are to be completed before being submitted (with the exception of the SAE Form).

In all cases it remains the responsibility of the Investigator to ensure that the CRF has been completed correctly and that the data are accurate.

CRFs may be amended by the Trial Office, as appropriate, throughout the duration of the trial. Whilst this will not constitute a protocol amendment, sites will be notified of new versions of the form when they are available in the eRDC system, and in the case of the SAE Fform, new versions of the form must be implemented by participating sites immediately on receipt.

19.2 Archiving

It is the responsibility of the Principal Investigator to ensure all essential trial documentation and source records (e.g. signed Informed Consent Forms, Investigator Site Files, Pharmacy Files, patients' hospital notes, copies of CRFs etc.) at their site are securely retained for at least 15 years after the end of the trial. Do not destroy any documents without prior approval from the CRCTU Document Storage Manager.

20 QUALITY MANAGEMENT

The trial is being conducted under the auspices of the Cancer Research UK Clinical Trials Unit (CRCTU) according to the current guidelines for Good Clinical Practice (GCP). UK and Irish participating sites will be monitored by CRCTU staff and other European Sites will be monitored by their respective National Co–Sponsor Centres to confirm compliance with the protocol and the protection of patients' rights as detailed in the Declaration of Helsinki.

20.1 Site Set-up and Initiation

All sites will be required to sign appropriate contracts with their National Co-Sponsor Centre prior to participation. In addition all participating Investigators will be asked to sign the necessary agreements and supply a current CV with evidence of recent GCP training to the National Co-Sponsor Centre. All members of the site research team will also be required to sign a Site Signature and Delegation Log which lists the range of duties that have been delegated to them for the trial. This should be counter-

signed by the Principal Investigator and returned to the National Co-Sponsor Centre. Prior to commencing recruitment all sites will undergo a process of initiation. Key members of the site research team will be required to attend either a meeting or a teleconference covering aspects of the trial design, protocol procedures, Adverse Event reporting, collection and reporting of data, and record keeping. Sites will be provided with an Investigator Site File and a Pharmacy File containing essential documentation, instructions, and other documentation required for the conduct of the trial. The National Co-Sponsor Centre must be informed immediately of any change in the site research team.

20.2 On-site Monitoring

Monitoring will be carried out as required following a risk assessment and as documented in the BEACON-Neuroblastoma Quality Management Plan (QMP) and the National Co-Sponsor Monitoring Plan for each respective country. It is the responsibility of participating National Co-Sponsor Centres to ensure that the level and process of monitoring described in the National Co-Sponsor Monitoring Plan for that country is in accordance with their national regulations and to inform the Coordinating Sponsor of any issues.

Additional on-site monitoring visits may be triggered for example by poor CRF completion rates, poor data quality, low or high SAE reporting rates, excessive number of patient withdrawals or deviations. If a monitoring visit is required the National Co-Sponsor Centre (or equivalent) will contact the site to arrange a date for the proposed visit and will provide the site with written confirmation. Investigators will allow the BEACON-Neuroblastoma trial staff access to source documents as requested.

20.3 Central Monitoring

Where a patient has given explicit consent and national regulations permit, sites will be instructed to send in copies of signed Informed Consent Forms to National Co-Sponsor Centres for in-house review.

Trial staff from the Coordinating Sponsor will be in regular contact with the National Co-Sponsor Centre/site research team to check on progress and address any queries that they may have. Trials staff will check incoming CRFs for compliance with the protocol, data consistency, missing data and timing.

CRFs will be initially checked by the National Co-Sponsor Centre and Data Clarification Forms requesting missing data or clarification of inconsistencies or discrepancies will be sent electronically to sites via the eRDC system.

National Co-Sponsor Centres/Sites may be suspended from further recruitment in the event of serious and persistent non-compliance with the protocol and/or GCP. Any major problems identified during monitoring may be reported to BEACON-Neuroblastoma Trial Management Group and the relevant regulatory bodies. This includes reporting serious breaches of GCP and/or the trial protocol to the appropriate regulatory bodies.

20.4 Audit and Inspection

The Investigator will permit trial-related monitoring, audits, ethical review, and regulatory inspection(s) at their site, providing direct access to source data/documents.

National Co-Sponsor Centres and research sites are also requested to notify the BEACON-Neuroblastoma Trial Office of any inspections by the Competent Authorities.

20.5 Notification of Serious Breaches

This trial is sponsored by the University of Birmingham, in the UK. In accordance with Regulation 29A of the Medicines for Human Use (Clinical Trials) Regulations 2004 and its amendments, the

Coordinating Sponsor of the trial is responsible for notifying the licensing authority in writing of any serious breach of:

- The conditions and principles of GCP in connection with that trial or;
- The protocol relating to that trial, within 7 days of becoming aware of that breach

For the purposes of this regulation, a “serious breach” is a breach which is likely to effect to a significant degree:

- The safety or physical or mental integrity of the patients in the trial; or
- The scientific value of the trial

National Co-Sponsor Centres/Sites are therefore requested to notify the BEACON-Neuroblastoma Trial Office of a suspected trial-related serious breach of GCP and/or the trial protocol. Where the Trial Office is investigating whether or not a serious breach has occurred, National Co-Sponsor Centres and research sites are also requested to co-operate with the BEACON-Neuroblastoma Trial Office in providing sufficient information to report the breach to the Competent Authority where required and in undertaking any corrective and/or preventive action.

21 END OF TRIAL DEFINITION

The end of trial will be the date of the last participant last visit. The Coordinating Sponsor will notify the Competent Authority and Research Ethics Committee that the trial has ended and will provide them with a summary of the clinical trial report within 6 months of the end of trial.
The trial results will be included on the clinical trial registry (ISRCTN) and on the Clinical Trials Information System. A lay summary will be included on the UK Health Research Authority website, the Clinical Trials Information System, and the trial website and the Cancer Research UK website.

22 STATISTICAL CONSIDERATIONS

22.1 Trial Design

BEACON-Neuroblastoma was a 3x2 factorial randomised Phase II trial. Patients were randomised to bevacizumab or not, to irinotecan or topotecan or neither, with a backbone of temozolomide. There were 6 arms:
1) Temozolomide only (T)
2) Temozolomide + Bevacizumab (BT)
3) Irinotecan + Temozolomide (IT)
4) Irinotecan + Temozolomide + Bevacizumab (BIT)
5) Temozolomide + Topotecan (TTo)
6) Temozolomide + Topotecan + Bevacizumab (BTTo)

The bevacizumab randomisation (part 1) was initially a Phase II randomised trial (Jung design (Jung 2008)) to evaluate whether bevacizumab was sufficiently active, as assessed by objective response rate (CR + PR), to warrant further investigation in a larger Phase III trial with EFS and OS as the main endpoints. This objective has now been achieved, so the randomisation (part 2) continued with PFS as the primary endpoint, but still as a Phase II comparison, i.e. using a relaxed alpha. The irinotecan and topotecan randomisations will use a probability-based Bayesian approach for the primary presentation of the data.

Following the recruitment of 160 patients the chemotherapy randomisation will continue, the bevacizumab randomisation will be closed and an additional randomisation to test the addition of dinutuximab beta will be opened. The trial will utilise a 2x2 factorial design; the design will assume no

interaction between the two chemotherapy regimens and dinutuximab beta, though the presence of interactions will be explored at the analysis stage.

The new four arms will be as follows:

1) Temozolomide only (T)
2) Dinutuximab beta + Temozolomide- (dBT)
3) Temozolomide + Topotecan (TTo)
4) Temozolomide + Topotecan + Dinutuximab beta (dBTTo)

22.2 Definition of Outcome Measures

22.2.1 Primary

- Best response is defined as Complete Response or Partial Response at any time during the first 6 cycles of trial treatment. Response is measured using the RECIST 1.1 criteria (Appendix 4 – RECIST Criteria 1.1) for patients with measurable disease or the new INRC criteria for patients with evaluable disease (see Appendix 5 – Tumor Response at Metastatic Soft Tissue and Bone Sites (Park et al. 2017)).
- For the Bevacizumab randomisation part 2 only: PFS is defined as the time from randomisation until first event (progression, recurrence following response or death without progression or recurrence). For those patients who do not experience event during the course of the trial, PFS times will be censored at the date of their last available trial assessment.

22.2.2 Secondary

- Adverse Events (AEs) will be recorded according to the NCI Common Toxicity Criteria for Adverse Events (CTCAE) version 4.0 (Appendix 3 - Common Toxicity Criteria Grading).
- PFS is defined as the time from randomisation until first event (progression, recurrence following response or death without progression or recurrence). For those patients who do not experience event during the course of the trial, progression-free survival times will be censored at the date of their last available trial assessment.
- EFS is defined as the time from randomisation until first event (progression, recurrence following response, second malignancy or death without progression or recurrence). For those patients who do not experience an event during the course of the trial, EFS times will be censored at the date of their last available trial assessment.
- Overall survival (OS) is defined as the time from randomisation until death from any cause. Patients who do not die during the course of the trial will be censored at the date of their last available trial assessment.

22.2.3 Exploratory/Tertiary

Exploratory/tertiary endpoints are described in section 2.2.

22.3 Sample Size

Bevacizumab Randomisation

For the bevacizumab randomisation (part 1), a 15% increase in response rate with bevacizumab would be considered sufficiently promising to warrant further investigation. Assuming a baseline response rate of 25% without bevacizumab (i.e. 40% with bevacizumab), using a two stage Minimax Jung design (Jung 2008), with α and β both set to 0.2 (a relaxed α has been used as this is a Phase II trial with the aim of obtaining sufficient evidence of activity, not definitive results), 53 patients per arm in total are required. Stage one requires 42 patients per arm; if there are ≥2 more responses in the bevacizumab arm compared to the no bevacizumab arm, the trial will proceed to the second stage. At the end of stage 2, with 53 patients per arm, if there are ≥4 more responses in the bevacizumab arm compared to the no

bevacizumab arm, bevacizumab will be considered sufficiently active to warrant further investigation. This cut point will be considered as a guideline and other factors – such as toxicity – will be taken into account before making the clinical decision on whether to proceed to a Phase III trial. The baseline response rate of 25% without bevacizumab is an estimate; the actual observed response rate may be different, leading to some change – possibly an increase – in the number of patients and responses needed, so we will aim to recruit 160 patients in total to allow for this eventuality. The differential needed to go to phase III trial will be adjusted as appropriate.

For the bevacizumab randomisation (part 2), assuming 40% PFS at 1 year in the control arm, with 160 patients and 80 events in total there will be 80% power to detect a difference of 15% at 1p=0.15 (calculated using the *stpower logrank* command Stata). If there were a large treatment effect, it is possible that this randomisation might demonstrate clear evidence of benefit for bevacizumab; following Rubinstein [68], a p-value of p<0.005 may be needed in a Phase II trial to warrant such a conclusion

Irinotecan and Topotecan Randomisations

For the irinotecan and topotecan randomisations, a probability-based Bayesian approach has been adopted. Despite the lack of evidence, the temozolomide + irinotecan combination is widely considered, especially in the USA, to be standard therapy for relapsed/refractory neuroblastoma. Hence, demonstrating that the response rate with temozolomide + irinotecan is not likely to be more than 15% better may be insufficient to change practice. Using a conventional randomised Phase II design – e.g. Jung – with a smaller than 15% difference would require a much larger number of patients and would not be feasible within the proposed timescale. Similar considerations apply to the topotecan question, where promising single-arm Phase II data exist. Hence, based on the posterior distributions derived from the observed response rates, probabilities that the response rates with irinotecan (i.e. TI and BTI arms) and with topotecan (i.e. TTo and BTTo arms) are greater than the response rates with no irinotecan or no topotecan (i.e. T and TB arms) will be given for a range of differences. Some scenarios are provided in the tables below (assuming 25% response rate with no irinotecan and with no topotecan, with 60 patients per arm for the irinotecan versus no irinotecan comparison and 40 patients per arm for the topotecan versus no topotecan comparison). A risk ratio (RR) > 1.0 indicates a higher response rate in the experimental arm, i.e. risk ratio of positive event in the experimental arm compared to positive event in the control arm. Absolute differences in response rate of 0%, 5%, 10% and 15% correspond to RRs of 1.00, 1.2, 1.4 and 1.6 respectively. Ln(RR) is assumed to be distributed normally with variance derived by delta method (Whitehead 2003). Similar consideration applies to the topotecan randomisation.

For the irinotecan and topotecan randomisations, Table 25 below shows scenarios with 60 patients per arm. For example, if a risk ratio of 1.0 is observed (i.e. no observed difference between the two arms), the probability of the true risk ratio being more than 1.2 will be 28%. With a risk ratio of 1.4 in favour of the topotecan arm, there will be an 88% chance of the experimental arm being better than the control arm (i.e. probability of true risk ratio > 1.0 = 88%).

Table 25: Scenario probabilities based on probability-based Bayesian approach (n=60 per arm)

Control Rate	Difference	Experimental Rate	# Events	RR	Pr (true RR>1.0| data) (%)	Pr (true RR>1.2| data) (%)	Pr (true RR>1.4| data) (%)	Pr (true RR>1.6| data) (%)
0.25	0	0.25	30	1.0	50	28	14	7
0.25	0.05	0.3	33	1.2	73	50	30	17
0.25	0.1	0.35	36	1.4	88	71	50	32
0.25	0.15	0.4	39	1.6	96	85	69	50

The irinotecan and topotecan randomisations are essentially free questions that will provide some preliminary unbiased randomised evidence on whether irinotecan and topotecan are useful agents in relapsed/refractory neuroblastoma. Randomisation of about 60 patients per arm will be much more informative than the current non-randomised data, and will allow a decision to be made as to whether irinotecan and/or topotecan are worthy of further investigation or whether they should be dropped as treatments for relapsed/refractory neuroblastoma.

Dinutuximab beta randomisation

An additional 64 patients will be recruited from the point at which the amendment is opened and will be randomised 2:1 to receive dinutuximab beta or not. Assuming a control arm response rate of 25% and experimental arm response rate of 45%, with 64 patients in total there will be 80% power at 1p=0.23. The calculation was performed using Sample Size Tables for Clinical Studies software. The calculation assumes no drop outs. The dinutuximab beta question would not be taken any further if at the final analysis 1p>0.23 and it would be concluded that dinutuximab beta was not active when added to chemotherapy.

22.4 Interim and Main Analyses of Outcome Measures

Detailed methodology for summary and statistical analyses of the data collected in this trial will be documented in a Statistical Analysis Plan, which will be dated and maintained by the Coordinating Sponsor. This document may modify the plans outlined in the protocol; however, any major modifications of the primary endpoint definition and/or its analysis will also be reflected in a protocol amendment.

22.4.1 Planned Interim Analyses

The trial will have one formal interim analysis for bevacizumab question to determine whether to continue recruitment for bevacizumab randomisation (part 1). The interim analysis will be presented in confidence to an independent Data Monitoring Committee (DMC). The DMC will meet at least annually to assess safety of patients during the recruitment phase of the study. The first stage of the trial will recruit 42 patients into each arm of the bevacizumab randomisation and have been assessed for best response. The results of stage 1 will be presented to the independent DMC by the trial statistician. Based on the results seen in stage 1, the trial may continue to recruit up to 60 patients in each arm of the bevacizumab randomisation.

22.4.2 Main Analysis

First main analysis will be performed for primary and secondary outcomes when all patients have completed treatment and been assessed for response. Subsequent analyses will evaluate long term outcome.

Primary analysis of the primary outcome measure will not be adjusted for the other treatment allocation. Secondary analysis of the primary outcome measure and secondary outcome measures analyses will be analysed as a factorial design. That is, all patients allocated to bevacizumab will be compared to all patients allocated to no bevacizumab, with stratification by irinotecan and topotecan allocation; similarly, all patients allocated to irinotecan will be compared to all patients allocated to no irinotecan, with stratification by bevacizumab allocation and the topotecan question will be stratified for bevacizumab allocation; all patients allocated to dinutuximab beta will be compared to all patients allocated to no dinutuximab beta, with stratification by topotecan allocation. This analysis is considered appropriate since there is no reason to anticipate interactions between bevacizumab and irinotecan or topotecan or between dinutuximab beta and topotecan as the agents have different mechanisms of action; however, interactions will be explored, although there will be limited power to detect these. Analyses adjusted for the stratification factors will also be performed.

The final analysis of the primary endpoint, response (based on the local response assessment), will be performed once all patients have completed treatment and been assessed for best response. Patients who are not assessable for response – e.g. because of early stopping of treatment or death – will be assumed to be non-responders.

All analyses will be intention to treat, with all patients analysed in the arm to which they were randomised. Baseline patient features will be tabulated. Analyses will be primarily descriptive.

Bevacizumab randomisation (part 1): For the primary endpoint of best response, the number of responders and proportion will be presented. The risk ratio with confidence intervals will be calculated. Exploratory generalised linear model will be performed and p-values will be given for information.

Bevacizumab randomisation (part 2): For the primary endpoint of PFS, Kaplan-Meier survival curves will be produced and hazard ratios, with confidence intervals (CIs), will be calculated using Cox regression models.

Irinotecan and topotecan randomisations: For comparison of the groups, posterior probability distributions will be plotted of the observed risk ratio for response – see example plots below. Non-informative priors will be used so the posterior distribution simply reflects the actual data – i.e. Pr (true, parameter|data). Conventional secondary analyses, as per the Bevacizumab randomisation, will also be performed.

Figure 3: Probability plots for irinotecan and topotecan questions

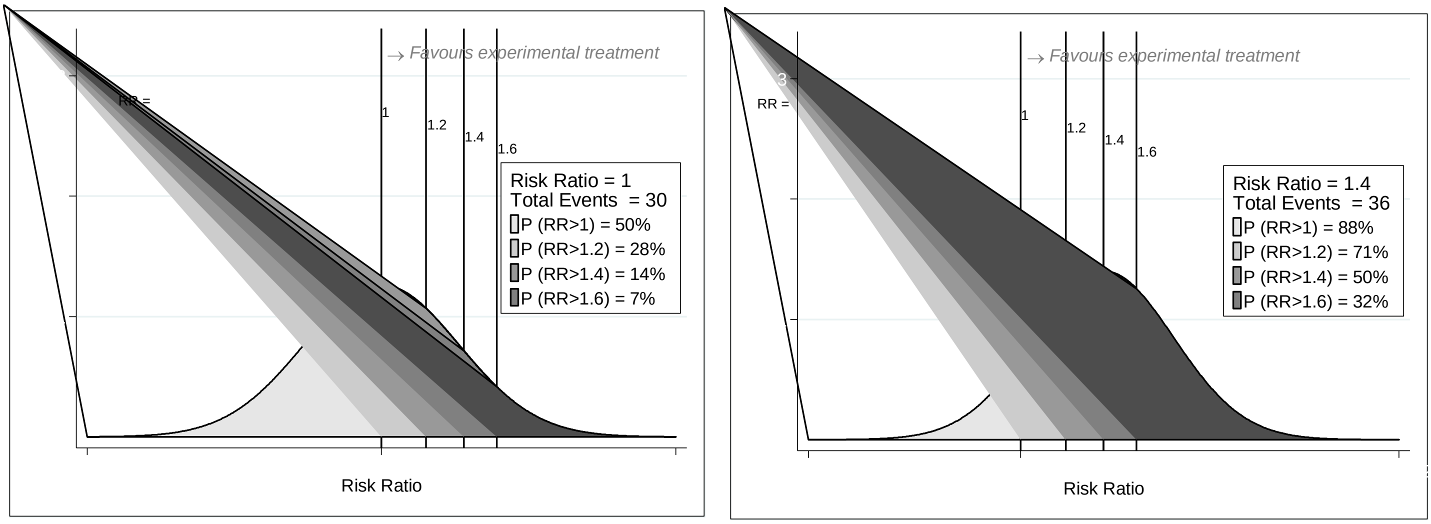

Risk Ratio = 1.00; Total Events = 30 **Risk Ratio = 1.4; Total Events = 36**

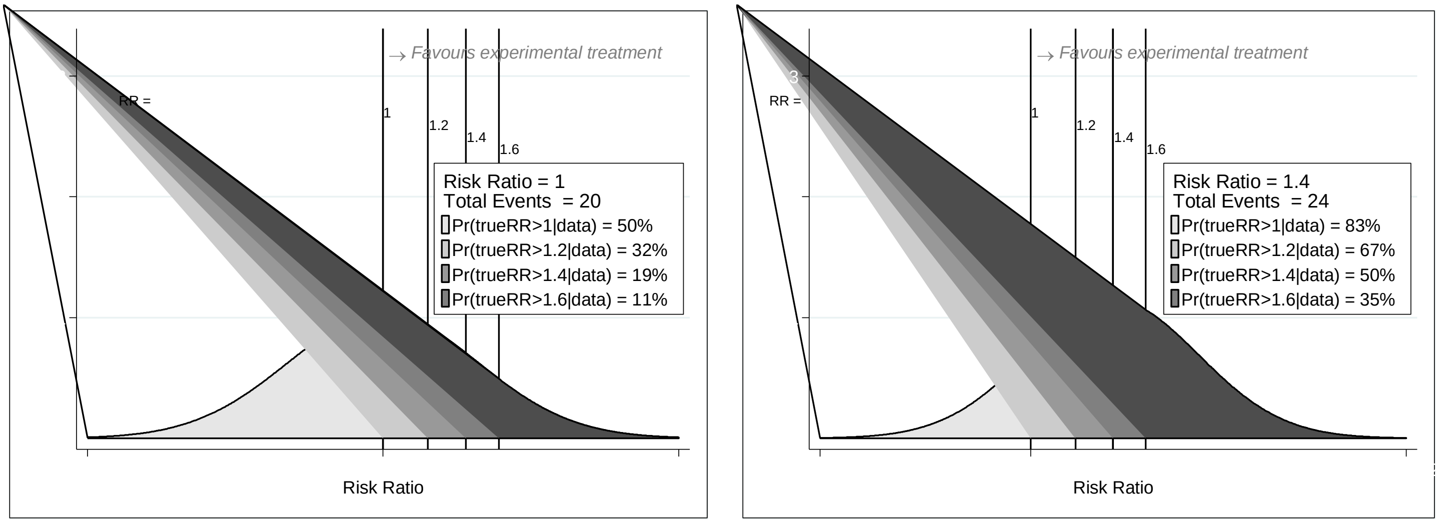

In the first plot, if no difference is observed between the arms (i.e. RR=1.00), there is only a 7% chance that the true difference in response rate in favour of irinotecan is >15% and an 14% chance that it is >10%; in this case, it might be concluded that there is relatively little chance of irinotecan being an effective agent. In the second plot, if a 10% difference is observed (i.e. RR=1.4), there is an 88% chance that the true response rate is better with irinotecan and a 50% chance that it is more than 10% better; in this case, it might be concluded that irinotecan is worthy of further investigation in a larger trial. The interpretation of the plots for the topotecan question will be similar.

Dinutuximab beta randomisation: chi squared test will be used to compare the treatments. Other analyses will be as per bevacizumab randomisation (part 1).

Analysis of Secondary and Tertiary Endpoints

Safety

Safety data will be summarised by arm for all treated patients using appropriate tabulations and descriptive statistics. Exploratory standard statistical tests will be performed and p-values will be given for information. All patients who receive at least one dose of study medications will be considered evaluable for safety analyses. The analysis of safety will extend through 90 days after the last administration of a study drug.

Survival Outcomes (PFS, EFS and OS)

For time-to-event data (PFS, EFS and OS) Kaplan-Meier life tables will be produced and hazard ratios, with confidence intervals (CIs), will be calculated. Exploratory log-rank test for time-to-event data will be performed and p-values will be given for information. Probability plots for the hazard ratios, as per the irinotecan randomisation, will also be presented for information.

Biomarker Analysis

Analysis of laboratory data will be performed as specified in the analysis plan.

22.5 Stopping Guidelines

The independent DMC will review the safety data at regular intervals and will make recommendations to the Trial Steering Committee (TSC) if they have concerns regarding any of the arms. The bevacizumab randomisation is designed as a two stage trial with a stopping criterion for lack of activity.

23 TRIAL ORGANISATIONAL STRUCTURE

23.1 Coordinating Sponsor

The University of Birmingham is the Coordinating Sponsor for this study.

23.2 Co-Sponsor Centres

The trial is being conducted under the auspices of the Cancer Research UK Clinical Trials Unit (CRCTU), University of Birmingham, UK.

The Coordinating Investigator in each country, known as the National Coordinating Investigator, on behalf of the Coordinating Sponsor, is responsible for the application for an Ethics Committee/Institutional Review Board approval according to national and institutional guidelines. Furthermore, the National Co-Sponsor/Coordinating Investigator will provide all authorised institutions of the participating countries with all documents required for an IEC/IRB approval according to local law

and regulation. The authorised institutions will provide all further documents required by national law and for application at the responsible Ethics Committee.

Strict adherence to all specifications laid down in this protocol is required for all aspects of the trial conduct; the investigator may not modify or alter the procedures described in this protocol.

23.3 Relationship of trial committees

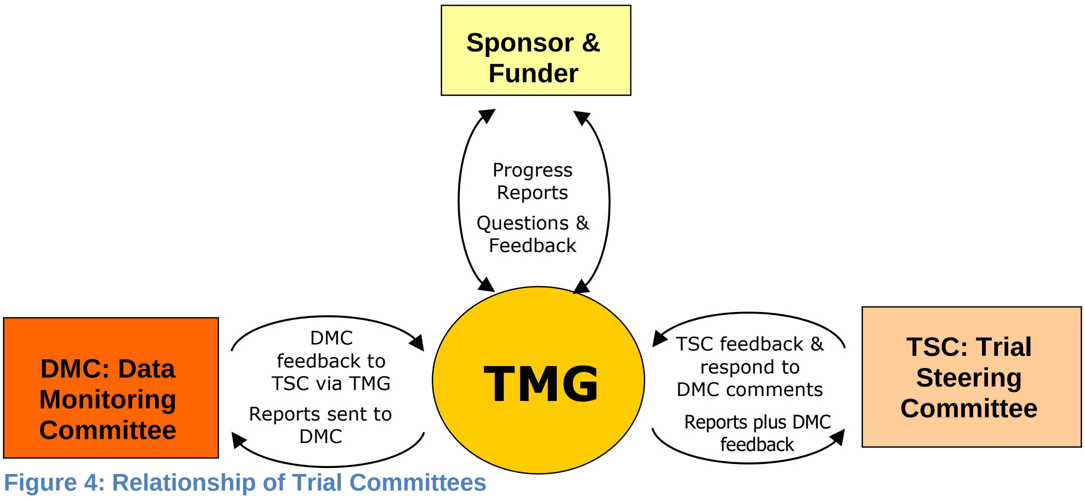

Figure 4: Relationship of Trial Committees

23.4 Trial Management Group

The Trial Management Group (TMG) will consist, as a minimum, of the Chief Investigator, Co-investigators and the trial team at the CRCTU. The Trial Management team will be responsible for the clinical set-up, ongoing management, promotion of the study and interpretation of the results. The Trial Management Group will meet approximately every 6 months.

23.5 Trial Steering Committee

The Trial Steering Committee (TSC) will consist of the an Independent Chairman, the Chief Investigator, other National Coordinating Investigators, members of the TMG as appropriate and two additional independent members. The TSC will provide overall supervision of the trial in particular trial progress, adherence to the protocol, patient safety and consideration of new information. The TSC will hold teleconferences or meet at least every 6-12 months. An emergency teleconference / meeting may be convened if a major safety issue is identified. Using feedback from the Data Monitoring Committee (DMC), the TSC may consider discontinuing the trial if the recruitment rate or data quality are unacceptable or if any issues arise that may compromise patient safety.

23.6 Data Monitoring Committee

Analyses will be supplied in confidence at least annually to an independent Data Monitoring Committee (DMC), which will be asked to give advice on whether the accumulated data from the trial, together with the results from other relevant research, justifies the continuing recruitment and treatment of patients. The DMC will operate in accordance with a trial specific charter based upon the template created by the Damocles Group. The DMC may meet prior to the trial opening, prior to the TSC and annually thereafter during the recruitment and treatment phases of the trial. The DMC will also review the data after 42 patients have been enrolled into a bevacizumab or non-bevacizumab receiving arm of the study (for whom primary outcome is available). Additional meetings may be called if recruitment is much faster than anticipated and the DMC may, at their discretion, request to meet more frequently or continue to meet following completion of recruitment. An emergency meeting may also be convened if a major safety issue is identified. The DMC will report directly to the Trial Steering Committee who may decide to convey the findings of the DMC to the National Co-Sponsor Centres, funders, and Sponsor as appropriate. The DMC may consider recommending the discontinuation of the trial if the recruitment rate or data quality are unacceptable or if any issues are identified which may compromise patient safety. Though unlikely in this early phase study, it is possible that the trial might also stop early if the interim

analyses showed differences in activity between treatments that were deemed to be convincing to the clinical community.

23.7 Finance

This is an academic-initiated and academic-led trial.

Bevacizumab is provided free of charge by F. Hoffmann-La Roche Ltd. F. Hoffmann-La Roche Ltd will also be performing biomarker analyses at their own costs. Irinotecan, temozolomide and topotecan will not be provided free of charge by the Sponsor and are available commercially.

Dinutuximab beta is provided free of charge by EUSA Pharma, including labelling and distribution. Temozolomide and topotecan will not be provided free of charge by the Sponsor and are available commercially.

Professor Andrew Peet holds a Programme Grant which will provide funding towards performing functional imaging and the central analysis of results. Other outstanding functional imaging costs are being covered by Imagine for Margo.

It is the responsibility of the National Co-Sponsor to obtain sufficient funding to run the trial within their respective nation state. The National Co-Sponsor has the obligation to provide administration for the region as set out in appendix 1 for the conduct of the trial in accordance with the terms of the protocol.

In the UK, the BEACON-Neuroblastoma trial is funded by a Cancer Research UK (CRUK) (UK) through a Clinical Trials Advisory and Awards Committee (CTAAC) grant. Imagine for Margo and Solving Kids Cancer are providing financial support for the running costs of the trial and additional funds are sought locally within each country.

23.8 NIHR CRN Portfolio

The BEACON-Neuroblastoma trial is a National Institute for Health Research (NIHR) Clinical Research Network (CRN) Portfolio study.

24 ETHICAL CONSIDERATIONS

The accepted basis for the conduct of clinical trials in humans is founded on the protection of human rights and the dignity of human beings with regard to the application of biology and medicine and requires the **compliance with the principles of Good Clinical Practice (GCP) and detailed guidelines** in line with those principles (Directive 2001/20/EC (2) and Directive 2005/28/EC (1)).

GCP (Good Clinical Practice) is a set of internationally recognised ethical and scientific quality requirements which must be observed for designing, conducting, recording and reporting clinical trials that involve the participation of human subjects. Compliance with good clinical practice provides assurance that the rights, safety and well-being of trial patients are protected, and that the results of the clinical trials are credible (Article 1 (2) of Directive 2001/20/EC).

The Coordinating Sponsor and Investigators shall consider all relevant guidance with respect to commencing and conducting a clinical trial (Article 4 of the Directive 2005/28/EC) and take into account the consensus on harmonisation for GCP.

The conduct of this trial shall be based on the following **international ethical and statutory source:**

the 18th World Medical Association **Declaration of Helsinki**, Finland, June 1964, amended at the 48th World Medical Association General Assembly, Somerset West, Republic of South Africa, October 1996 (website: http://www.wma.net/en/30publications/10policies/b3/index.html).

If the region has adopted the Convention for the protection of Human Rights and dignity of the human being with regard to the application of biology and medicine: **Convention on Human Rights and Biomedicine** (CETS No.: 164)

(Council of Europe – Ratification signed in the following countries: Bosnia and Herzegovina, Bulgaria, Croatia, Cyprus, Czech Republic, Denmark, Estonia, Finland, France, Georgia, Greece, Hungary, Iceland, Italy, Latvia, Lithuania, Luxembourg, Moldova, Montenegro, Netherlands, Norway, Portugal, Romania, San Marino, Serbia, Slovakia, Slovenia, Spain, Sweden, Switzerland, The Former Yugoslav Republic of Macedonia, Turkey, and Ukraine).

Directive 2001/20/EC OF THE EUROPEAN PARLIAMENT AND OF THE COUNCIL of 4 April 2001 on the approximation of the laws, regulations and administrative provisions of the Member States relating to the implementation of good clinical practice in the conduct of clinical trials on medicinal products for human use (Official Journal L21, 01/05/2001 P. 0034 – 0044) and detailed guidance.

Directive 2005/28/EC of 8 April 2005 laying down principles and detailed guidelines for good clinical practice as regards investigational medicinal products for human use, as well as the requirements for authorisation of the manufacturing or importation of such products (Official Journal L 91, 09/04/2005 P. 0013 – 0019).

Directive 95/46/EC of the European Parliament and of the Council of 24 October 1995 on the protection of individuals with regard to the processing of personal data and on the free movement of such data (Official Journal L 281 , 23/11/1995 P. 0031 – 0050).

In the UK, **t**he trial will be conducted in accordance with the **Research Governance Framework** for Health and Social Care, the applicable UK Statutory Instruments, (which include the **Medicines for Human Use Clinical Trials 2004** and subsequent amendments, the **Data Protection Act 1998** and **Human Tissue Act 2008**.

Scientific guidelines relating to the quality, safety and efficacy of medicinal products for human use, as agreed upon by the CHMP and published by the Agency, as well as the other pharmaceutical Community guidelines published by the Commission in the different volumes of the rules governing medicinal products in the European Community (as stated in the Directive 2005/28/EC (9)).

Before patients can be registered on to the study, each site must obtain all necessary regulatory approvals in accordance with their national laws. It is the Principal Investigators responsibility to ensure any subsequent amendments gain the necessary approval. This does not affect the clinicians' responsibility to take immediate action if thought necessary to protect the health and interest of individual patients.

Sites will not be permitted to register patients until written confirmation of the institutional approval has been received by the BEACON-Neuroblastoma Trial Office and the National Co-Sponsor.

25 CONFIDENTIALITY AND DATA PROTECTION

Personal data recorded on all documents will be regarded as strictly confidential and will be handled and stored in accordance with the appropriate laws in each participating nation state.

Patients will be identified using only their unique trial number, initials and date of birth on the CRFs and on correspondence between the BEACON-Neuroblastoma Trial Office, National Co-Sponsor and participating sites.

The National Co-Sponsor may receive from other parties and the Coordinating Sponsor's business information, trade secrets and/or confidential or proprietary information belonging to other parties. All such information which is designated as **confidential information** shall not be used for any purpose other than participation in the clinical trial and performance of obligations under this contract.

The National Co-Sponsor shall not copy or disclose any confidential information to a third party with the exception of employees, subcontracted partners, and healthcare professionals that require access to this information to perform their transferred obligations.

The restrictions shall not apply to:

Information that was already in the possession of the Coordinating Sponsor in receipt of the confidential information concerned before disclosure (except as a result of a breach of this agreement); information obtained independently from a third party source that was free to disclose the same; information that is in the public domain (except as a result of a breach of this or any other agreement); information that is required to be disclosed by law or any governmental or regulatory authority.

Where local laws permit, parents/patients are asked to give permission for the National Co-Sponsor to be sent a copy of their signed Informed Consent Form which will not be anonymised. This will be used to perform in-house monitoring of the consent process.

Similarly, **for UK patients only**, their full name, date of birth, National Health Service (NHS) number, or in Scotland the Community Health Index (CHI), address and hospital number will be collected at trial entry to allow tracing through the Cancer Registries and the NHS Information Centre for Health and Social Care (service provided by the Office of National Statistics) and to assist with long-term follow-up via other health care professionals (the patient's General Practitioner).

The Investigator must maintain documents not for submission to the Trial Office (e.g. Patient Identification Logs) in strict confidence. In the case of specific issues and/or queries from the regulatory authorities, it will be necessary to have access to the complete trial records, provided that patient confidentiality is protected.

26 INSURANCE AND INDEMNITY

The National Co-Sponsor Centres are responsible for obtaining insurance to set up and run the BEACON-Neuroblastoma trial in their respective countries and for ensuring that sites in their country are adequately covered.

University of Birmingham employees are indemnified by the University insurers for negligent harm caused by the design or coordination of the clinical trials they undertake whilst in the University's employment. The University of Birmingham cannot offer indemnity for non-negligent harm. The University of Birmingham is independent of any pharmaceutical company, and as such it is not covered by the Association of the British Pharmaceutical Industry (ABPI) guidelines for patient compensation.

In the UK, in terms of liability at a site, NHS Trust hospitals have a duty to care for patients treated, whether or not the patient is taking part in a clinical trial. Compensation is therefore available via NHS indemnity in the event of clinical negligence having being proven.

27 PUBLICATION POLICY

Results of this trial will be submitted for publication in peer reviewed journals. Manuscripts will be prepared by the Trial Management Group (TMG) and authorship will be determined by mutual agreement, taking account of the contribution made by each investigator/site.

Any publications and presentations prepared by Investigators must be reviewed by the TMG. Manuscripts must be submitted to the TMG in a timely fashion and in advance of being submitted for publication, to allow time for review and resolution of any outstanding issues. Authors must acknowledge that the trial was performed with the support of the University of Birmingham. Intellectual property rights will be addressed in the corresponding contracts between Coordinating Sponsor and National Co-Sponsor Centres/sites.

28 REFERENCE LIST

Ady, N., J. M. Zucker, B. Asselain, V. Edeline, F. Bonnin, J. Michon, R. Gongora, and L. Manil. 1995. 'A new 123I-MIBG whole body scan scoring method--application to the prediction of the response of metastases to induction chemotherapy in stage IV neuroblastoma', *Eur J Cancer*, 31A: 256-61.

Bagatell, R, L.M. Wagner, S. L. Cohn, J. M. Maris, C. P. Reynolds, and C. F. Stewart. 2009. 'Irinotecan plus temozolomide in children with recurrent or refractory neuroblastoma: A phase II Children's Oncology Group study.', *J Clin Oncol*, 27: abstract 10011.

Bagatell, R., W. B. London, L. M. Wagner, S. D. Voss, C. F. Stewart, J. M. Maris, C. Kretschmar, and S. L. Cohn. 2011. 'Phase II study of irinotecan and temozolomide in children with relapsed or refractory neuroblastoma: a Children's Oncology Group study', *J Clin Oncol*, 29: 208-13.

Beaudry, P., M. Nilsson, M. Rioth, D. Prox, D. Poon, L. Xu, P. Zweidler-Mckay, A. Ryan, J. Folkman, S. Ryeom, and J. Heymach. 2008. 'Potent antitumor effects of ZD6474 on neuroblastoma via dual targeting of tumor cells and tumor endothelium', *Mol Cancer Ther*, 7: 418-24.

Benesch, M., M. Windelberg, W. Sauseng, V. Witt, G. Fleischhack, H. Lackner, H. Gadner, U. Bode, and C. Urban. 2008. 'Compassionate use of bevacizumab (Avastin) in children and young adults with refractory or recurrent solid tumors', *Ann Oncol*, 19: 807-13.

Berthold, F., J. Boos, S. Burdach, R. Erttmann, G. Henze, J. Hermann, T. Klingebiel, B. Kremens, F. H. Schilling, M. Schrappe, T. Simon, and B. Hero. 2005. 'Myeloablative megatherapy with autologous stem-cell rescue versus oral maintenance chemotherapy as consolidation treatment in patients with high-risk neuroblastoma: a randomised controlled trial', *Lancet Oncol*, 6: 649-58.

Brisse, H. J., M. B. McCarville, C. Granata, K. B. Krug, S. L. Wootton-Gorges, K. Kanegawa, F. Giammarile, M. Schmidt, B. L. Shulkin, K. K. Matthay, V. J. Lewington, S. Sarnacki, B. Hero, M. Kaneko, W. B. London, A. D. Pearson, S. L. Cohn, and T. Monclair. 2011. 'Guidelines for imaging and staging of neuroblastic tumors: consensus report from the International Neuroblastoma Risk Group Project', *Radiology*, 261: 243-57.

Brodeur, G. M., J. Pritchard, F. Berthold, N. L. Carlsen, V. Castel, R. P. Castelberry, B. De Bernardi, A. E. Evans, M. Favrot, F. Hedborg, and et al. 1993. 'Revisions of the international criteria for neuroblastoma diagnosis, staging, and response to treatment', *J Clin Oncol*, 11: 1466-77.

Brodeur, G. M., R. C. Seeger, A. Barrett, F. Berthold, R. P. Castleberry, G. D'Angio, B. De Bernardi, A. E. Evans, M. Favrot, A. I. Freeman, and et al. 1988. 'International criteria for diagnosis, staging, and response to treatment in patients with neuroblastoma', *J Clin Oncol*, 6: 1874-81.

Broniscer, A., J. N. Baker, M. Tagen, A. Onar-Thomas, R. J. Gilbertson, A. M. Davidoff, A. S. Pai Panandiker, W. Leung, T. K. Chin, C. F. Stewart, M. Kocak, C. Rowland, T. E. Merchant, S. C. Kaste, and A. Gajjar. 2010. 'Phase I study of vandetanib during and after radiotherapy in children with diffuse intrinsic pontine glioma', *J Clin Oncol*, 28: 4762-8.

Burchill, S. A., I. J. Lewis, K. R. Abrams, R. Riley, J. Imeson, A. D. Pearson, R. Pinkerton, and P. Selby. 2001. 'Circulating neuroblastoma cells detected by reverse transcriptase polymerase chain reaction for tyrosine hydroxylase mRNA are an independent poor prognostic indicator in stage 4 neuroblastoma in children over 1 year', *J Clin Oncol*, 19: 1795-801.

Burchill, Susan A., Klaus Beiske, Hiroyuki Shimada, Peter F. Ambros, Robert Seeger, Godelieve A.M. Tytgat, Penelope R. Brock, Michelle Haber, Julie R. Park, and Frank Berthold. 2017. 'Recommendations for the standardization of bone marrow disease assessment and reporting in children with neuroblastoma on behalf of the International Neuroblastoma Response Criteria Bone Marrow Working Group', 123: 1095-105.

Canete, A., S. Navarro, J. Bermudez, A. Pellin, V. Castel, and A. Llombart-Bosch. 2000. 'Angiogenesis in neuroblastoma: relationship to survival and other prognostic factors in a cohort of neuroblastoma patients', *J Clin Oncol*, 18: 27-34.

Chanthery, Y. H., W. C. Gustafson, M. Itsara, A. Persson, C. S. Hackett, M. Grimmer, E. Charron, S. Yakovenko, G. Kim, K. K. Matthay, and W. A. Weiss. 2012. 'Paracrine signaling through MYCN enhances tumor-vascular interactions in neuroblastoma', *Sci Transl Med*, 4: 115ra3.

Chisholm, Julia C., Johannes H. M. Merks, Michela Casanova, Gianni Bisogno, Daniel Orbach, Jean-Claude Gentet, Anne-Sophie Thomassin-Defachelles, Pascal Chastagner, Stephen Lowis, Milind Ronghe, Kieran McHugh, Rick R. van Rijn, Magalie Hilton, Jeanette Bachir, Sabine

Fürst-Recktenwald, Birgit Geoerger, and Odile Oberlin. 2017. 'Open-label, multicentre, randomised, phase II study of the EpSSG and the ITCC evaluating the addition of bevacizumab to chemotherapy in childhood and adolescent patients with metastatic soft tissue sarcoma (the BERNIE study)', *European Journal of Cancer*, 83: 177-84.

Di Giannatale, A., K. McHugh, N. Dias, A. Devos, B. Geoerger, T. Jaspan, R. Calmon, F. Courbon, K. Malekzadeh, I. Aerts, A. Verschuur, M. Casanova, F. Fouyssac, H. Rubie, N. Corradini, Y. Perel, C. M. Zwaan, R. Riccardi, M. C. Le Deley, and H. Caron. 2012. 'Phase II study of temozolomide in combination with topotecan (TOTEM) in relapsed or refractory neuroblastoma and other pediatric solid malignancies: A European ITCC study.', *J Clin Oncol*, Abstract 9517.

Dickson, P. V., J. B. Hamner, T. L. Sims, C. H. Fraga, C. Y. Ng, S. Rajasekeran, N. L. Hagedorn, M. B. McCarville, C. F. Stewart, and A. M. Davidoff. 2007. 'Bevacizumab-induced transient remodeling of the vasculature in neuroblastoma xenografts results in improved delivery and efficacy of systemically administered chemotherapy', *Clin Cancer Res*, 13: 3942-50.

'Dinutuximab beta Apeiron Assessment report'. 2017. European Medicines Agency. http://www.ema.europa.eu/docs/en_GB/document_library/EPAR_-_Public_assessment_report/human/003918/WC500227726.pdf.

Eisenhauer, E. A., P. Therasse, J. Bogaerts, L. H. Schwartz, D. Sargent, R. Ford, J. Dancey, S. Arbuck, S. Gwyther, M. Mooney, L. Rubinstein, L. Shankar, L. Dodd, R. Kaplan, D. Lacombe, and J. Verweij. 2009. 'New response evaluation criteria in solid tumours: revised RECIST guideline (version 1.1)', *Eur J Cancer*, 45: 228-47.

EMA, European Medicines Agency. 2008. 'Ethical considerations for clinical trials on medicinal products conducted with the pediatric population. Recommendations of the ad hoc group for the development of implementing

guidelines for Directive 2001/20/EC relating to good clinical practice in the conduct of clinical trials on medicinal products for human use', *Available at: ftp://ftp.cordis.europa.eu/pub/fp7/docs/ethical-considerations-paediatrics_en.pdf [last accessed 20 Oct 2011]*.

Emens, L. A., and G. Middleton. 2015. 'The interplay of immunotherapy and chemotherapy: harnessing potential synergies', *Cancer Immunol Res*, 3: 436-43.

Erbe, A. K., W. Wang, L. Carmichael, K. Kim, E. A. Mendonca, Y. Song, D. Hess, P. K. Reville, W. B. London, A. Naranjo, J. A. Hank, M. B. Diccianni, R. A. Reisfeld, S. D. Gillies, K. K. Matthay, S. L. Cohn, M. D. Hogarty, J. M. Maris, J. R. Park, M. F. Ozkaynak, A. L. Gilman, A. L. Yu, and P. M. Sondel. 2018. 'Neuroblastoma Patients' KIR and KIR-Ligand Genotypes Influence Clinical Outcome for Dinutuximab-based Immunotherapy: A Report from the Children's Oncology Group', *Clin Cancer Res*, 24: 189-96.

Federico, S. M., M. B. McCarville, B. L. Shulkin, P. M. Sondel, J. A. Hank, P. Hutson, M. Meagher, A. Shafer, C. Y. Ng, W. Leung, W. E. Janssen, J. Wu, S. Mao, R. C. Brennan, V. M. Santana, A. S. Pappo, and W. L. Furman. 2017. 'A Pilot Trial of Humanized Anti-GD2 Monoclonal Antibody (hu14.18K322A) with Chemotherapy and Natural Killer Cells in Children with Recurrent/Refractory Neuroblastoma', *Clin Cancer Res*, 23: 6441-49.

Fox, E., R. Aplenc, R. Bagatell, M. K. Chuk, E. Dombi, W. Goodspeed, A. Goodwin, M. Kromplewski, N. Jayaprakash, M. Marotti, K. H. Brown, B. Wenrich, P. C. Adamson, B. C. Widemann, and F. M. Balis. 2010. 'A phase 1 trial and pharmacokinetic study of cediranib, an orally bioavailable pan-vascular endothelial growth factor receptor inhibitor, in children and adolescents with refractory solid tumors', *J Clin Oncol*, 28: 5174-81.

Friedman, H. S., M. D. Prados, P. Y. Wen, T. Mikkelsen, D. Schiff, L. E. Abrey, W. K. Yung, N. Paleologos, M. K. Nicholas, R. Jensen, J. Vredenburgh, J. Huang, M. Zheng, and T. Cloughesy. 2009. 'Bevacizumab alone and in combination with irinotecan in recurrent glioblastoma', *J Clin Oncol*, 27: 4733-40.

Furman WL, Federico SM, McCarville MB, et al. . 2016. 'Improved clinical responses with the concomitant use of an anti-GD2 monoclonal antibody and chemotherapy in newly diagnosed children with high-risk (HR) neuroblastoma (NB): Preliminary results of a phase II study.', *Journal of Clinical Oncology*, 34: 10501.

Garaventa, A., R. Luksch, S. Biasotti, G. Severi, M. R. Pizzitola, E. Viscardi, A. Prete, S. Mastrangelo, M. Podda, R. Haupt, and B. De Bernardi. 2003. 'A phase II study of topotecan with vincristine and doxorubicin in children with recurrent/refractory neuroblastoma', *Cancer*, 98: 2488-94.

Garaventa, A., S. Parodi, B. De Bernardi, D. Dau, C. Manzitti, M. Conte, F. Casale, E. Viscardi, M. Bianchi, P. D'Angelo, G. A. Zanazzo, R. Luksch, C. Favre, A. Tamburini, and R. Haupt. 2009. 'Outcome of children with neuroblastoma after progression or relapse. A retrospective study of the Italian neuroblastoma registry', *Eur J Cancer*, 45: 2835-42.

Garrido-Laguna, I., M. Hidalgo, and R. Kurzrock. 2011. 'The inverted pyramid of biomarker-driven trials', *Nat Rev Clin Oncol*, 8: 562-6.

Gatta, G., G. Zigon, R. Capocaccia, J. W. Coebergh, E. Desandes, P. Kaatsch, G. Pastore, R. Peris-Bonet, and C. A. Stiller. 2009. 'Survival of European children and young adults with cancer diagnosed 1995-2002', *Eur J Cancer*, 45: 992-1005.

Glade Bender, J. L., P. C. Adamson, J. M. Reid, L. Xu, S. Baruchel, Y. Shaked, R. S. Kerbel, E. M. Cooney-Qualter, D. Stempak, H. X. Chen, M. D. Nelson, M. D. Krailo, A. M. Ingle, S. M. Blaney, J. J. Kandel, and D. J. Yamashiro. 2008. 'Phase I trial and pharmacokinetic study of bevacizumab in pediatric patients with refractory solid tumors: a Children's Oncology Group Study', *J Clin Oncol*, 26: 399-405.

Glade Bender, J. L., A. Lee, J. M. Reid, S. Baruchel, T. Roberts, S. D. Voss, B. Wu, C. H. Ahern, A. M. Ingle, P. Harris, B. J. Weigel, and S. M. Blaney. 2013. 'Phase I pharmacokinetic and pharmacodynamic study of pazopanib in children with soft tissue sarcoma and other refractory solid tumors: a children's oncology group phase I consortium report', *J Clin Oncol*, 31: 3034-43.

Grill, Jacques, Maura Massimino, Eric Bouffet, Amedeo A. Azizi, Geoffrey McCowage, Adela Cañete, Frank Saran, Marie-Cécile Le Deley, Pascale Varlet, Paul S. Morgan, Tim Jaspan, Chris Jones, Felice Giangaspero, Helen Smith, Josep Garcia, Markus C. Elze, Raphaël F. Rousseau, Lauren Abrey, Darren Hargrave, and Gilles Vassal. 2018. 'Phase II, Open-Label, Randomized, Multicenter Trial (HERBY) of Bevacizumab in Pediatric Patients With Newly Diagnosed High-Grade Glioma', 36: 951-58.

Gururangan, S., S. N. Chi, T. Young Poussaint, A. Onar-Thomas, R. J. Gilbertson, S. Vajapeyam, H. S. Friedman, R. J. Packer, B. N. Rood, J. M. Boyett, and L. E. Kun. 2010. 'Lack of efficacy of bevacizumab plus irinotecan in children with recurrent malignant glioma and diffuse brainstem glioma: a Pediatric Brain Tumor Consortium study', *J Clin Oncol*, 28: 3069-75.

Hiddemann, W., M. Kneba, M. Dreyling, N. Schmitz, E. Lengfelder, R. Schmits, M. Reiser, B. Metzner, H. Harder, S. Hegewisch-Becker, T. Fischer, M. Kropff, H. E. Reis, M. Freund, B. Wormann, R. Fuchs, M. Planker, J. Schimke, H. Eimermacher, L. Trumper, A. Aldaoud, R. Parwaresch, and M. Unterhalt. 2005. 'Frontline therapy with rituximab added to the combination of cyclophosphamide, doxorubicin, vincristine, and prednisone (CHOP) significantly improves the outcome for patients with advanced-stage follicular lymphoma compared with therapy with CHOP alone: results of a prospective randomized study of the German Low-Grade Lymphoma Study Group', *Blood*, 106: 3725-32.

Jakovljevic, G., S. Culic, J. Stepan, A. Bonevski, and S. Seiwerth. 2009. 'Vascular endothelial growth factor in children with neuroblastoma: a retrospective analysis', *J Exp Clin Cancer Res*, 28: 143.

Jung, S. H. 2008. 'Randomized phase II trials with a prospective control', *Stat Med*, 27: 568-83.

Kim, E. S., S. Z. Soffer, J. Huang, K. W. McCrudden, A. Yokoi, C. A. Manley, W. Middlesworth, J. J. Kandel, and D. J. Yamashiro. 2002. 'Distinct response of experimental neuroblastoma to combination antiangiogenic strategies', *J Pediatr Surg*, 37: 518-22.

Kowalczyk, A., M. Gil, I. Horwacik, Z. Odrowaz, D. Kozbor, and H. Rokita. 2009. 'The GD2-specific 14G2a monoclonal antibody induces apoptosis and enhances cytotoxicity of chemotherapeutic drugs in IMR-32 human neuroblastoma cells', *Cancer Lett*, 281: 171-82.

Kushner, B. H., K. Kramer, S. Modak, and N. K. Cheung. 2006. 'Irinotecan plus temozolomide for relapsed or refractory neuroblastoma', *J Clin Oncol*, 24: 5271-6.

Ladenstein R, Gray J, Valteau Couanet D, et al. . 2015. "Short and Long term infusion of ch14.18/CHO immunotherapy: Toxicity profiles and outcomes in 530 High risk neuroblastoma patients in 2 SIOPEN trials." In *47th Congress of the International Society of Paediatric Oncology (SIOP)* Cape Town.

Ladenstein, R., U. Poetschger, R. Luksch, P. Brock, V. Castel, I. Yaniv, V. Papadakis, G. Laureys, J. Malis, W. Balwierz, E. Ruud, P. Kogner, H. Schroder, A. Forjaz de Lacerda, M. Beck Popovic, P. Bician, M. Garami, T. Trahair, A.D. Pearson, and D. Valteau-Couanet. 2011. 'Busulphan-

melphalan as a myeloablative therapy (MAT) for high-risk neuroblastoma: Results from the HR-NBL1/SIOPEN trial.', *J Clin Oncol 29: 2011 (suppl: abstr 2)*.

Ladenstein, R., U. Potschger, O. Hartman, A. D. Pearson, T. Klingebiel, V. Castel, I. Yaniv, T. Demirer, and G. Dini. 2008. '28 years of high-dose therapy and SCT for neuroblastoma in Europe: lessons from more than 4000 procedures', *Bone Marrow Transplant*, 41 Suppl 2: S118-27.

Ladenstein, R., D. Valteau-Couanet, P. Brock, I. Yaniv, V. Castel, G. Laureys, J. Malis, V. Papadakis, A. Lacerda, E. Ruud, P. Kogner, M. Garami, W. Balwierz, H. Schroeder, M. Beck-Popovic, G. Schreier, D. Machin, U. Potschger, and A. Pearson. 2010. 'Randomized Trial of prophylactic granulocyte colony-stimulating factor during rapid COJEC induction in pediatric patients with high-risk neuroblastoma: the European HR-NBL1/SIOPEN study', *J Clin Oncol*, 28: 3516-24.

Lewington, V., Z. Bar Sever, and T. Lynch. 2009. 'Development of a semi-quantitative I-123 mIBG reporting method in high risk neuroblastoma. ', *J Nucl Med. 2009; 50 (Supplement 2):1379*.

Liu, G., H. S. Rugo, G. Wilding, T. M. McShane, J. L. Evelhoch, C. Ng, E. Jackson, F. Kelcz, B. M. Yeh, F. T. Lee, Jr., C. Charnsangavej, J. W. Park, E. A. Ashton, H. M. Steinfeldt, Y. K. Pithavala, S. D. Reich, and R. S. Herbst. 2005. 'Dynamic contrast-enhanced magnetic resonance imaging as a pharmacodynamic measure of response after acute dosing of AG-013736, an oral angiogenesis inhibitor, in patients with advanced solid tumors: results from a phase I study', *J Clin Oncol*, 23: 5464-73.

Lode NH, Valteau-Couanet D, et al. 2016. "Phase II clinical trial with long-term infusion of anti-GD2 antibody ch14.18/CHO in combination with interleukin-2 (IL2) in patients with high risk neuroblastoma [Abstract number: 10562]." In *American Society of Clinical Oncology Annual Meeting*. Chicago, Illinois.

London, W. B., V. Castel, T. Monclair, P. F. Ambros, A. D. Pearson, S. L. Cohn, F. Berthold, A. Nakagawara, R. L. Ladenstein, T. Iehara, and K. K. Matthay. 2011. 'Clinical and biologic features predictive of survival after relapse of neuroblastoma: a report from the International Neuroblastoma Risk Group project', *J Clin Oncol*, 29: 3286-92.

London, W. B., C. N. Frantz, L. A. Campbell, R. C. Seeger, B. A. Brumback, S. L. Cohn, K. K. Matthay, R. P. Castleberry, and L. Diller. 2010. 'Phase II randomized comparison of topotecan plus cyclophosphamide versus topotecan alone in children with recurrent or refractory neuroblastoma: a Children's Oncology Group study', *J Clin Oncol*, 28: 3808-15.

Maris, J. M. 2010. 'Recent advances in neuroblastoma', *N Engl J Med*, 362: 2202-11.

Maris, J. M., J. Courtright, P. J. Houghton, C. L. Morton, R. Gorlick, E. A. Kolb, R. Lock, M. Tajbakhsh, C. P. Reynolds, S. T. Keir, J. Wu, and M. A. Smith. 2008. 'Initial testing of the VEGFR inhibitor AZD2171 by the pediatric preclinical testing program', *Pediatr Blood Cancer*, 50: 581-7.

Maris, J. M., M. D. Hogarty, R. Bagatell, and S. L. Cohn. 2007. 'Neuroblastoma', *Lancet*, 369: 2106-20.

Mascarenhas, L., E. R. Lyden, P. P. Breitfeld, D. O. Walterhouse, S. S. Donaldson, C. N. Paidas, D. M. Parham, J. R. Anderson, W. H. Meyer, and D. S. Hawkins. 2010. 'Randomized phase II window trial of two schedules of irinotecan with vincristine in patients with first relapse or progression of rhabdomyosarcoma: a report from the Children's Oncology Group', *J Clin Oncol*, 28: 4658-63.

Matthay, K. K., H. Brisse, D. Couanet, J. Couturier, J. Benard, V. Mosseri, V. Edeline, J. Lumbroso, D. Valteau-Couanet, and J. Michon. 2003. 'Central nervous system metastases in neuroblastoma: radiologic, clinical, and biologic features in 23 patients', *Cancer*, 98: 155-65.

Matthay, K. K., V. Edeline, J. Lumbroso, M. L. Tanguy, B. Asselain, J. M. Zucker, D. Valteau-Couanet, O. Hartmann, and J. Michon. 2003. 'Correlation of early metastatic response by 123I-metaiodobenzylguanidine scintigraphy with overall response and event-free survival in stage IV neuroblastoma', *J Clin Oncol*, 21: 2486-91.

Matthay, K. K., C. P. Reynolds, R. C. Seeger, H. Shimada, E. S. Adkins, D. Haas-Kogan, R. B. Gerbing, W. B. London, and J. G. Villablanca. 2009. 'Long-term results for children with high-risk neuroblastoma treated on a randomized trial of myeloablative therapy followed by 13-cis-retinoic acid: a children's oncology group study', *J Clin Oncol*, 27: 1007-13.

Matthay, K. K., B. Shulkin, R. Ladenstein, J. Michon, F. Giammarile, V. Lewington, A. D. Pearson, and S. L. Cohn. 2010. 'Criteria for evaluation of disease extent by (123)I-metaiodobenzylguanidine scans in neuroblastoma: a report for the International Neuroblastoma Risk Group (INRG) Task Force', *Br J Cancer*, 102: 1319-26.

Matthay, K. K., J. G. Villablanca, R. C. Seeger, D. O. Stram, R. E. Harris, N. K. Ramsay, P. Swift, H. Shimada, C. T. Black, G. M. Brodeur, R. B. Gerbing, and C. P. Reynolds. 1999. 'Treatment of high-risk neuroblastoma with intensive chemotherapy, radiotherapy, autologous bone marrow transplantation, and 13-cis-retinoic acid. Children's Cancer Group', *N Engl J Med*, 341: 1165-73.

Messina, J. A., S. C. Cheng, B. L. Franc, M. Charron, B. Shulkin, B. To, J. M. Maris, G. Yanik, R. A. Hawkins, and K. K. Matthay. 2006. 'Evaluation of semi-quantitative scoring system for metaiodobenzylguanidine (mIBG) scans in patients with relapsed neuroblastoma', *Pediatr Blood Cancer*, 47: 865-74.

Modak, Shakeel, Brian H. Kushner, Ellen Basu, Stephen S. Roberts, and Nai-Kong V. Cheung. 2017. 'Combination of bevacizumab, irinotecan, and temozolomide for refractory or relapsed neuroblastoma: Results of a phase II study', 64: e26448.

Mody R, Naranjo A, et al. . 2018. 'Phase II trial of irinotecan/ temozolomide/ dinutuximab/ granulocyte macrophage colony stimulating factor (I/T/DIN/GMCSF) in children with relapsed/ refractory neuroblastoma (NBL): A report from the Children’s Oncology Group. ', *Journal of Clinical Oncology*, 36.

Mody, R., A. Naranjo, C. Van Ryn, A. L. Yu, W. B. London, B. L. Shulkin, M. T. Parisi, S. E. Servaes, M. B. Diccianni, P. M. Sondel, J. G. Bender, J. M. Maris, J. R. Park, and R. Bagatell. 2017. 'Irinotecan-temozolomide with temsirolimus or dinutuximab in children with refractory or relapsed neuroblastoma (COG ANBL1221): an open-label, randomised, phase 2 trial', *Lancet Oncol*, 18: 946-57.

Moreno L, Moroz V, Owens C, et al. 2019. "Bevacizumab for children with relapsed & refractory high-risk neuroblastomoa (RR-HRNB): Results of the BEACON-neuroblastoma randomized phase II trial – A European ITCC-SIOPEN trial." In *European Society for Medical Oncology (ESMO) Congress 2019;*. Barcelona, Spain.

Moreno, Lucas, Veronica Moroz, Cormac Owens, Jennifer Laidler, Dominique Valteau-Couanet, Marion Gambart, Victoria Castel, Natasha Van Eijkelenburg, Aurora Castellano, Karsten Nysom, Nicolas U. Gerber, Genevieve Laureys, Ruth Lydia Ladenstein, Guy Makin, Sucheta Vaidya, Estelle Thebaud, Pamela Kearns, Andrew DJ Pearson, and Keith Wheatley. 2019. 'Temozolomide versus irinotecan-temozolomide for children with relapsed and refractory high risk neuroblastoma (RR-HRNB): Results of the BEACON-Neuroblastoma randomized phase 2 trial—A European Innovative Therapies for Children with Cancer (ITCC) - International Society of Pediatric Oncology Europe Neuroblastoma Group (SIOPEN) trial', *Journal of Clinical Oncology*, 37: 10001-01.

Navid, Fariba, Victor M. Santana, Michael Neel, M. Beth McCarville, Barry L. Shulkin, Jianrong Wu, Catherine A. Billups, Shenghua Mao, Vinay M. Daryani, Clinton F. Stewart, Michelle Kunkel, Wendene Smith, Deborah Ward, Alberto S. Pappo, Armita Bahrami, David M. Loeb, Jennifer Reikes Willert, Bhaskar N. Rao, and Najat C. Daw. 2017. 'A phase II trial evaluating the feasibility of adding bevacizumab to standard osteosarcoma therapy', 141: 1469-77.

Naylor, S. 2003. 'Biomarkers: current perspectives and future prospects', *Expert Rev Mol Diagn*, 3: 525-9.

Park, Julie R., Rochelle Bagatell, Susan L. Cohn, Andrew D. Pearson, Judith G. Villablanca, Frank Berthold, Susan Burchill, Ariane Boubaker, Kieran McHugh, Jed G. Nuchtern, Wendy B. London, Nita L. Seibel, O. Wolf Lindwasser, John M. Maris, Penelope Brock, Gudrun Schleiermacher, Ruth Ladenstein, Katherine K. Matthay, and Dominique Valteau-Couanet. 2017. 'Revisions to the International Neuroblastoma Response Criteria: A Consensus Statement From the National Cancer Institute Clinical Trials Planning Meeting', 35: 2580-87.

Patterson, D. M., D. Gao, D. N. Trahan, B. A. Johnson, A. Ludwig, E. Barbieri, Z. Chen, J. Diaz-Miron, L. Vassilev, J. M. Shohet, and E. S. Kim. 2011a. 'Effect of MDM2 and vascular endothelial growth factor inhibition on tumor angiogenesis and metastasis in neuroblastoma', *Angiogenesis*, 14: 255-66.

———. 2011b. 'Effect of MDM2 and vascular endothelial growth factor inhibition on tumor angiogenesis and metastasis in neuroblastoma', *Angiogenesis*.

Pearson, A. D., C. R. Pinkerton, I. J. Lewis, J. Imeson, C. Ellershaw, D. Machin, Group European Neuroblastoma Study, Cancer Children's, and Group Leukaemia. 2008. 'High-dose rapid and standard induction chemotherapy for patients aged over 1 year with stage 4 neuroblastoma: a randomised trial', *Lancet Oncol*, 9: 247-56.

Pritchard, J., S. J. Cotterill, S. M. Germond, J. Imeson, J. de Kraker, and D. R. Jones. 2005. 'High dose melphalan in the treatment of advanced neuroblastoma: results of a randomised trial (ENSG-1) by the European Neuroblastoma Study Group', *Pediatr Blood Cancer*, 44: 348-57.

Roche. 'Bevacizumab Summary of Product Characteristics', Accessed 15 February 2011. http://www.medicines.org.uk/EMC/medicine/15748/SPC/Avastin+25mg+ml+concentrate+for+solution+for+infusion/.

Rosen, M. A., and M. D. Schnall. 2007. 'Dynamic contrast-enhanced magnetic resonance imaging for assessing tumor vascularity and vascular effects of targeted therapies in renal cell carcinoma', *Clin Cancer Res*, 13: 770s-76s.

Rossler, J., Y. Monnet, F. Farace, P. Opolon, E. Daudigeos-Dubus, A. Bourredjem, G. Vassal, and B. Geoerger. 2011. 'The selective VEGFR1-3 inhibitor axitinib (AG-013736) shows antitumor activity in human neuroblastoma xenografts', *Int J Cancer*, 128: 2748-58.

Rossler, J., M. Taylor, B. Geoerger, F. Farace, J. Lagodny, R. Peschka-Suss, C. M. Niemeyer, and G. Vassal. 2008. 'Angiogenesis as a target in neuroblastoma', *Eur J Cancer*, 44: 1645-56.

Rubie, H., J. Chisholm, A. S. Defachelles, B. Morland, C. Munzer, D. Valteau-Couanet, V. Mosseri, C. Bergeron, C. Weston, C. Coze, A. Auvrignon, L. Djafari, R. Hobson, C. Baunin, F. Dickinson, H. Brisse, K. McHugh, L. Biassoni, F. Giammarile, and G. Vassal. 2006. 'Phase II study of temozolomide in relapsed or refractory high-risk neuroblastoma: a joint Societe Francaise des Cancers de l'Enfant and United Kingdom Children Cancer Study Group-New Agents Group Study', *J Clin Oncol*, 24: 5259-64.

Sawyers, C. L. 2008. 'The cancer biomarker problem', *Nature*, 452: 548-52.

Segerstrom, L., D. Fuchs, U. Backman, K. Holmquist, R. Christofferson, and F. Azarbayjani. 2006. 'The anti-VEGF antibody bevacizumab potently reduces the growth rate of high-risk neuroblastoma xenografts', *Pediatr Res*, 60: 576-81.

Siebert, N., C. Eger, D. Seidel, M. Juttner, M. Zumpe, D. Wegner, S. Kietz, K. Ehlert, G. J. Veal, W. Siegmund, M. Weiss, H. Loibner, R. Ladenstein, and H. N. Lode. 2016. 'Pharmacokinetics and pharmacodynamics of ch14.18/CHO in relapsed/refractory high-risk neuroblastoma patients treated by long-term infusion in combination with IL-2', *MAbs*, 8: 604-16.

Siebert, Nikolai, Christian Jensen, Sascha Troschke-Meurer, Maxi Zumpe, Madlen Jüttner, Karoline Ehlert, Silke Kietz, Ina Müller, and Holger N. Lode. 2016. 'Neuroblastoma patients with high-affinity FCGR2A, -3A and stimulatory KIR 2DS2 treated by long-term infusion of anti-GD2 antibody ch14.18/CHO show higher ADCC levels and improved event-free survival', *OncoImmunology*, 5: e1235108.

Siebert, Nikolai, Diana Seidel, Christin Eger, Madlen Jüttner, and Holger N. Lode. 2014. 'Functional bioassays for immune monitoring of high-risk neuroblastoma patients treated with ch14.18/CHO anti-GD2 antibody', *PloS one*, 9: e107692-e92.

Simon, T., B. Hero, A. Faldum, R. Handgretinger, M. Schrappe, T. Klingebiel, and F. Berthold. 2011. 'Long term outcome of high-risk neuroblastoma patients after immunotherapy with antibody ch14.18 or oral metronomic chemotherapy', *BMC Cancer*, 11: 21.

Simon, T., A. Langler, F. Berthold, T. Klingebiel, and B. Hero. 2007. 'Topotecan and etoposide in the treatment of relapsed high-risk neuroblastoma: results of a phase 2 trial', *J Pediatr Hematol Oncol*, 29: 101-6.

Simon, T., A. Langler, U. Harnischmacher, M. C. Fruhwald, N. Jorch, A. Claviez, F. Berthold, and B. Hero. 2007. 'Topotecan, cyclophosphamide, and etoposide (TCE) in the treatment of high-risk neuroblastoma. Results of a phase-II trial', *J Cancer Res Clin Oncol*, 133: 653-61.

Sims, T. L., R. F. Williams, C. Y. Ng, S. F. Rosati, Y. Spence, and A. M. Davidoff. 2008. 'Bevacizumab suppresses neuroblastoma progression in the setting of minimal disease', *Surgery*, 144: 269-75.

Slamon DJ, Leyland-Jones B, et al. . 2001. 'Use of chemotherapy plus a monoclonal antibody against HER2 for metastatic breast cancer that overexpresses HER2', *New England Journal of Medicine*, 15: 783 - 92.

Tan, D. S., G. V. Thomas, M. D. Garrett, U. Banerji, J. S. de Bono, S. B. Kaye, and P. Workman. 2009. 'Biomarker-driven early clinical trials in oncology: a paradigm shift in drug development', *Cancer J*, 15: 406-20.

Vassal, G., F. Giammarile, M. Brooks, B. Geoerger, D. Couanet, J. Michon, E. Stockdale, M. Schell, A. Geoffray, J. C. Gentet, F. Pichon, H. Rubie, L. Cisar, S. Assadourian, and B. Morland. 2008.

'A phase II study of irinotecan in children with relapsed or refractory neuroblastoma: a European cooperation of the Societe Francaise d'Oncologie Pediatrique (SFOP) and the United Kingdom Children Cancer Study Group (UKCCSG)', *Eur J Cancer*, 44: 2453-60.

Viprey, V. F., M. V. Corrias, B. Kagedal, S. Oltra, K. Swerts, A. Vicha, R. Ladenstein, and S. A. Burchill. 2007. 'Standardisation of operating procedures for the detection of minimal disease by QRT-PCR in children with neuroblastoma: quality assurance on behalf of SIOPEN-R-NET', *Eur J Cancer*, 43: 341-50.

Whitehead, A. 2003. *Meta-analysis of controlled clinical trials* (John Wiley and Sons).

Yang, R. K., and P. M. Sondel. 2010. 'Anti-GD2 Strategy in the Treatment of Neuroblastoma', *Drugs Future*, 35: 665.

Yanik, G., M.T. Parisi, A. Naranjo, K. K. Matthay, W. B. London, P.W. McGrady, S. Kreissman, and B. Shulkin. 2010. 'MIBG scoring as a prognostic indicator in patients with stage IV neuroblastoma: A COG study', *J Clin Oncol 28:15s, (suppl; abstr 9516)*.

Yap, T. A., S. K. Sandhu, P. Workman, and J. S. de Bono. 2010. 'Envisioning the future of early anticancer drug development', *Nat Rev Cancer*, 10: 514-23.

Yu, A. L., A. L. Gilman, M. F. Ozkaynak, W. B. London, S. G. Kreissman, H. X. Chen, M. Smith, B. Anderson, J. G. Villablanca, K. K. Matthay, H. Shimada, S. A. Grupp, R. Seeger, C. P. Reynolds, A. Buxton, R. A. Reisfeld, S. D. Gillies, S. L. Cohn, J. M. Maris, P. M. Sondel, and Group Children's Oncology. 2010. 'Anti-GD2 antibody with GM-CSF, interleukin-2, and isotretinoin for neuroblastoma', *N Engl J Med*, 363: 1324-34.

Zeng, Y., S. Fest, R. Kunert, H. Katinger, V. Pistoia, J. Michon, G. Lewis, R. Ladenstein, and H. N. Lode. 2005. 'Anti-neuroblastoma effect of ch14.18 antibody produced in CHO cells is mediated by NK-cells in mice', *Mol Immunol*, 42: 1311-9.

Zhen, Z., X. Sun, Y. He, Y. Cai, J. Wang, and Z. Guan. 2010. 'The sequence of drug administration influences the antitumor effects of bevacizumab and cyclophosphamide in a neuroblastoma model', *Med Oncol*.

APPENDIX 1 – WMA DECLARATION OF HELSINKI

WORLD MEDICAL ASSOCIATION DECLARATION OF HELSINKI
Recommendations guiding physicians
in biomedical research involving human subjects
Adopted by the 18th World Medical Assembly
Helsinki, Finland, June 1964
and amended by the
29th World Medical Assembly, Tokyo, Japan, October 1975
35th World Medical Assembly, Venice, Italy, October 1983
41st World Medical Assembly, Hong Kong, September 1989
and the
48th General Assembly, Somerset West, Republic of South Africa, October 1996

INTRODUCTION

It is the mission of the physician to safeguard the health of the people. His or her knowledge and conscience are dedicated to the fulfilment of this mission.

The Declaration of Geneva of the World Medical Association binds the physician with the words, "The Health of my patient will be my first consideration," and the International Code of Medical Ethics declares that, "A physician shall act only in the patient's interest when providing medical care which might have the effect of weakening the physical and mental condition of the patient."

The purpose of biomedical research involving human subjects must be to improve diagnostic, therapeutic and prophylactic procedures and the understanding of the aetiology and pathogenesis of disease.

In current medical practice most diagnostic, therapeutic or prophylactic procedures involve hazards. This applies especially to biomedical research.

Medical progress is based on research which ultimately must rest in part on experimentation involving human subjects.

In the field of biomedical research a fundamental distinction must be recognized between medical research in which the aim is essentially diagnostic or therapeutic for a patient, and medical research, the essential object of which is purely scientific and without implying direct diagnostic or therapeutic value to the person subjected to the research.

Special caution must be exercised in the conduct of research which may affect the environment, and the welfare of animals used for research must be respected.

Because it is essential that the results of laboratory experiments be applied to human beings to further scientific knowledge and to help suffering humanity, the World Medical Association has prepared the following recommendations as a guide to every physician in biomedical research involving human subjects. They should be kept under review in the future. It must be stressed that the standards as drafted are only a guide to physicians all over the world. Physicians are not relieved from criminal, civil and ethical responsibilities under the laws of their own countries.

I. BASIC PRINCIPLES

1. Biomedical research involving human subjects must conform to generally accepted scientific principles and should be based on adequately performed laboratory and animal experimentation and on a thorough knowledge of the scientific literature.

2. The design and performance of each experimental procedure involving human subjects should be clearly formulated in an experimental protocol which should be transmitted for consideration, comment and guidance to a specially appointed committee independent of the investigator and

the sponsor provided that this independent committee is in conformity with the laws and regulations of the country in which the research experiment is performed.

3. Biomedical research involving human subjects should be conducted only by scientifically qualified persons and under the supervision of a clinically competent medical person. The responsibility for the human subject must always rest with a medically qualified person and never rest on the subject of the research, even though the subject has given his or her consent.

4. 4. Biomedical research involving human subjects cannot legitimately be carried out unless the importance of the objective is in proportion to the inherent risk to the subject.

5. Every biomedical research project involving human subjects should be preceded by careful assessment of predictable risks in comparison with foreseeable benefits to the subject or to others. Concern for the interests of the subject must always prevail over the interests of science and society.

6. The right of the research subject to safeguard his or her integrity must always be respected. Every precaution should be taken to respect the privacy of the subject and to minimize the impact of the study on the subject's physical and mental integrity and on the personality of the subject.

7. Physicians should abstain from engaging in research projects involving human subjects unless they are satisfied that the hazards involved are believed to be predictable. Physicians should cease any investigation if the hazards are found to outweigh the potential benefits.

8. In publication of the results of his or her research, the physician is obliged to preserve the accuracy of the results. Reports of experimentation not in accordance with the principles laid down in this Declaration should not be accepted for publication.

9. In any research on human beings, each potential subject must be adequately informed of the aims, methods, anticipated benefits and potential hazards of the study and the discomfort it may entail. He or she should be informed that he or she is at liberty to abstain from participation in the study and that he or she is free to withdraw his or her consent to participation at any time. The physician should then obtain the subject's freely-given informed consent, preferably in writing.

10. When obtaining informed consent for the research project the physician should be particularly cautious if the subject is in a dependent relationship to him or her or may consent under duress. In that case the informed consent should be obtained by a physician who is not engaged in the investigation and who is completely independent of this official relationship.

11. In case of legal incompetence, informed consent should be obtained from the legal guardian in accordance with national legislation. Where physical or mental incapacity makes it impossible to obtain informed consent, or when the subject is a minor, permission from the responsible relative replaces that of the subject in accordance with national legislation. Whenever the minor child is in fact able to give a consent, the minor's consent must be obtained in addition to the consent of the minor's legal guardian.

12. The research protocol should always contain a statement of the ethical considerations involved and should indicate that the principles enunciated in the present Declaration are complied with.

II. MEDICAL RESEARCH COMBINED WITH PROFESSIONAL CARE (Clinical Research)

1. In the treatment of the sick person, the physician must be free to use a new diagnostic and therapeutic measure, if in his or her judgement it offers hope of saving life, re-establishing health or alleviating suffering.

2. The potential benefits, hazards and discomfort of a new method should be weighed against the advantages of the best current diagnostic and therapeutic methods.

3. In any medical study, every patient - including those of a control group, if any - should be assured of the best proven diagnostic and therapeutic method. This does not exclude the use of inert placebo in studies where no proven diagnostic or therapeutic method exists.

4. The refusal of the patient to participate in a study must never interfere with the physician-patient relationship.

5. If the physician considers it essential not to obtain informed consent, the specific reasons for this proposal should be stated in the experimental protocol for transmission to the independent committee (I, 2).

6. The physician can combine medical research with professional care, the objective being the acquisition of new medical knowledge, only to the extent that medical research is justified by its potential diagnostic or therapeutic value for the patient.

III. NON-THERAPEUTIC BIOMEDICAL RESEARCH INVOLVING HUMAN SUBJECTS (Non-Clinical Biomedical Research)

1. In the purely scientific application of medical research carried out on a human being, it is the duty of the physician to remain the protector of the life and health of that person on whom biomedical research is being carried out.

2. The subject should be volunteers - either healthy persons or patients for whom the experimental design is not related to the patient's illness.

3. The investigator or the investigating team should discontinue the research if in his/her or their judgement it may, if continued, be harmful to the individual.

4. In research on man, the interest of science and society should never take precedence over considerations related to the wellbeing of the subject.

APPENDIX 2 - DEFINITION OF ADVERSE EVENTS

Adverse Event (AE)

Any untoward medical occurrence in a patient or clinical trial subject administered a medicinal product and which does not necessarily have a causal relationship with this treatment.

Comment:

An AE can therefore be any unfavourable and unintended sign (including abnormal laboratory findings), symptom or disease temporally associated with the use of an investigational medicinal product, whether or not related to the investigational medicinal product.

Adverse Reaction (AR)

All untoward and unintended responses to an IMP related to any dose administered.

Comment:

An AE judged by either the reporting Investigator or Sponsor as having causal relationship to the IMP qualifies as an AR. The expression reasonable causal relationship means to convey in general that there is evidence or argument to suggest a causal relationship.

Serious Adverse Event (SAE)

Any untoward medical occurrence or effect that at any dose:

- Results in death
- Is life-threatening*
- Requires hospitalisation** or prolongation of existing inpatients' hospitalisation
- Results in persistent or significant disability or incapacity
- Is a congenital anomaly/birth defect
- Or is otherwise considered medically significant by the Investigator***

Comments:

The term severe is often used to describe the intensity (severity) of a specific event. This is not the same as serious, which is based on patients/event outcome or action criteria.

* Life threatening in the definition of an SAE refers to an event in which the patient was at risk of death at the time of the event; it does not refer to an event that hypothetically might have caused death if it were more severe.

**Hospitalisation is defined as an unplanned, formal inpatient admission, even if the hospitalisation is a precautionary measure for continued observation. Thus, hospitalisation for protocol treatment (e.g. line insertion), elective procedures (unless brought forward because of worsening symptoms), or for social reasons (e.g. respite care), are not regarded as an SAE.

*** Medical judgment should be exercised in deciding whether an AE is serious in other situations. Important AEs that are not immediately life threatening or do not result in death or hospitalisation but may jeopardise the subject or may require intervention to prevent one of the other outcomes listed in the definition above, should be considered serious.

Serious Adverse Reaction (SAR)

An Adverse Reaction which also meets the definition of a Serious Adverse Event.

Suspected Unexpected Serious Adverse Reaction (SUSAR)

A SAR that is unexpected i.e. the nature, or severity of the event is not consistent with the applicable product information.

A SUSAR should meet the definition of an AR, UAR and SAR.

Unexpected Adverse Reaction (UAR)

An AR, the nature or severity of which is not consistent with the applicable product information (e.g. Investigator Brochure for an unapproved IMP or (compendium of) Summary of Product Characteristics (SPC) for a licensed product).

When the outcome of an AR is not consistent with the applicable product information the AR should be considered unexpected.

APPENDIX 3 - COMMON TOXICITY CRITERIA GRADING

Toxicities will be recorded according to the Common Terminology Criteria for Adverse Events (CTCAE), version 4.0. The full CTCAE document is available on the National Cancer Institute (NCI) website, the following address was correct when this version of the protocol was approved:

http://ctep.cancer.gov/protocolDevelopment/electronic_applications/ctc.htm

APPENDIX 4 – RECIST CRITERIA 1.1

The following contains excerpts from the RECIST criteria.
For more information regarding RECIST and a full copy of criteria, go to http://www.eortc.org (Eisenhauer et al. 2009)

Definitions for the modified response evaluation criteria in solid tumours

Measurable lesions

Tumour lesions: Must be accurately measured in at least one dimension (longest diameter in the plane of measurement is to be recorded) with a minimum size of:

- 10mm by CT scan (CT scan slice thickness no greater than 5 mm)
- 10mm calliper measurement by clinical exam (lesions which cannot be accurately measured with callipers should be recorded as non-measurable)
- 20mm by chest X-ray

Malignant lymph nodes: To be considered pathologically enlarged *and* measurable, a lymph node must be≥15mm in *short* axis when assessed by CT scan (CT scan slice thickness recommended to be no greater than 5mm). At baseline and in follow up, only the *short* axis will be measured and followed.

Non-measurable lesions

All other lesions, including small lesions (longest diameter < 10mm or pathological lymph nodes with P10 to < 15mm short axis), as well as truly non-measurable lesions. Lesions considered truly non-measurable include: leptomeningeal disease, ascites, pleural or pericardial effusion, inflammatory breast disease, lymphangitic involvement of skin or lung, abdominal masses/abdominal organomegaly, identified by physical exam that is not measurable by reproducible imaging techniques.

Target lesions

When more than one measurable lesion is present at baseline all lesions up to a maximum of five lesions total (and a maximum of two lesions per organ) representative of all involved organs should be identified as target lesions and will be recorded and measured at baseline (this means in instances where patients have only one or two organ sites involved a maximum of two and four lesions respectively will be recorded). Target lesions should be selected on the basis of their size (lesions with the longest diameter), be representative of all involved organs, but in addition should be those that lend themselves to reproducible repeated measurements. It may be the case that, on occasion, the largest lesion does not lend itself to reproducible measurement in which circumstance the next largest lesion which can be measured reproducibly should be selected.

Non-target lesions

All other lesions (or sites of disease) including pathological lymph nodes should be identified as non-target lesions and should also be recorded at baseline. Measurements are not required and these lesions should be followed as ‘present’, ‘absent’, or in rare cases ‘unequivocal progression’ (more details to follow). In addition, it is possible to record multiple non target lesions involving the same organ as a single item on the case record form (e.g. ‘multiple enlarged pelvic lymph nodes’ or ‘multiple liver metastases’).

Evaluation of target lesions

- Complete Response (CR): Disappearance of all target lesions. Any pathological lymph nodes (whether target or non-target) must have reduction in short axis to <10 mm.
- Partial Response (PR): At least a 30% decrease in the sum of diameters of target lesions, taking as reference the baseline sum diameters.
- Progressive Disease (PD): At least a 20% increase in the sum of diameters of target lesions, taking as reference the smallest sum on study (this includes the baseline sum if that is the smallest on study). In addition to the relative increase of 20%, the sum must also demonstrate an absolute increase of at least 5 mm. (Note: the appearance of one or more new lesions is also considered progression).
- Stable Disease (SD): Neither sufficient shrinkage to qualify for PR nor sufficient increase to qualify for PD, taking as reference the smallest sum diameters while on study.

Evaluation of non-target lesions

While some non-target lesions may actually be measurable, they need not be measured and instead should be assessed only qualitatively at the time points specified in the protocol.

- Complete Response (CR): Disappearance of all non-target lesions and normalisation of tumour marker level. All lymph nodes must be non-pathological in size (< 10mm short axis).
- Non-CR/Non-PD: Persistence of one or more non-target lesion(s) and/or maintenance of tumour marker level above the normal limits.
- Progressive Disease (PD): Unequivocal progression (see comments below) of existing non-target lesions. (Note: the appearance of one or more new lesions is also considered progression).

Evaluation of best overall response

Best response determination in trials where confirmation of complete or partial response IS NOT required: Best response in these trials is defined as the best response across all time points (for example, a patient who has SD at first assessment, PR at second assessment, and PD on last assessment has a best overall response of PR). When SD is believed to be best response, it must also meet the protocol specified minimum time from baseline.

If the minimum time is not met when SD is otherwise the best time point response, the patient's best response depends on the subsequent assessments. For example, a patient who has SD at first assessment, PD at second and does not meet minimum duration for SD, will have a best response of PD. The same patient lost to follow-up after the first SD assessment would be considered unevaluable.

Time point response: patients with target (+/- non target) disease:

Target lesions	***Non-target lesions***	***New lesions***	***Overall response***
CR	CR	No	CR
CR	Non CR/non PD	No	PR
CR	Not evaluated	No	PR
PR	Non PD or not all evaluated	No	PR
SD	Non PD or not all evaluated	No	SD
Not all evaluated	Non-PD	No	NE
PD	Any	Yes or No	PD
Any	PD	Yes or No	PD
Any	Any	Yes	PD

Time point response: patients with non-target disease only:

Non target lesions	***New lesions***	***Overall response***
CR	No	CR
Non CR/non PD	No	Non CR/non PD
Not all evaluated	No	NE
Unequivocal PD	Yes or no	PD
Any	Yes	PD

CR (complete response), PD (progressive disease) and NE (unevaluable). A ‘non CR/non PD’ is preferred over ‘stable disease’ for non-target disease since SD is increasingly used as endpoint for assessment of efficacy in some trials so to assign this category when no lesions can be measured is not advised.

APPENDIX 5 – TUMOR RESPONSE AT METASTATIC SOFT TISSUE AND BONE SITES (PARK ET AL. 2017)

RESPONSE	ANATOMICAL IMAGING + MIBG (FDG-PET)
**CR**	Resolution of all sites of disease, defined as:<br>Non-primary target and non-target lesions measure < 10 mm<br>AND<br>Lymph nodes identified as target lesions decrease to a short axis < 10 mm<br>AND<br>MIBG uptake or FDG-PET uptake (for MIBG-nonavid tumors) of non-primary lesions resolves completely
**PR**	≥30% decrease in sum of diameters* of non-primary lesions compared with baseline AND all of the following:<br>• Non-target lesions may be stable or smaller in size AND<br>• No new lesions AND<br>• ≥ 50% reduction in MIBG absolute bone score (relative MIBG<br>• bone score ≥ 0.1 to ≥ 0.5) or ≥ 50% reduction in number of FDG-PET–avid bone lesions‡§
**PD**	Any of the following<br>• Any new soft tissue lesion by CT/MRI that is also MIBG avid or FDG-PET avid<br>• Any new soft tissue lesion seen on anatomic imaging that is biopsied and confirmed to be neuroblastoma or ganglioneuroblastoma<br>• Any new bone site that is MIBG avid<br>• A new bone site that is FDG-PET avid (for MIBG-nonavid tumors) AND has CT/MRI findings consistent with tumor OR has been confirmed histologically to be neuroblastoma or ganglioneuroblastoma<br>• >20% increase in longest diameter taking as reference the smallest sum on study (this includes the baseline sum if that is the smallest on study) AND a minimum absolute increase of 5mm in sum of diameters of target soft tissue lesions<br>• Relative MIBG score ≥1.2
**SD**	Neither sufficient shrinkage for PR nor sufficient increase for PD of non-primary lesions

CR: Complete Response
PR: Partial Response
PD: Progressive Disease
SD: Stable Disease

May 2017

APPENDIX 6 - CURIE & SIOPEN SCORING METHODS FOR NEUROBLASTOMA

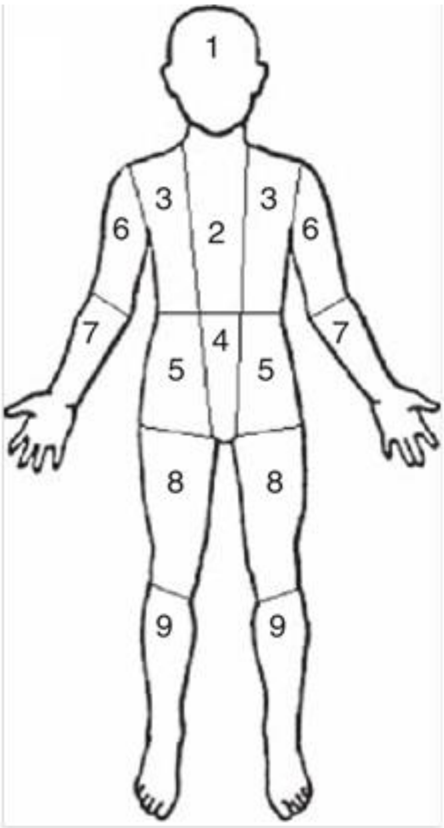

Figure 5: The CURIE Method

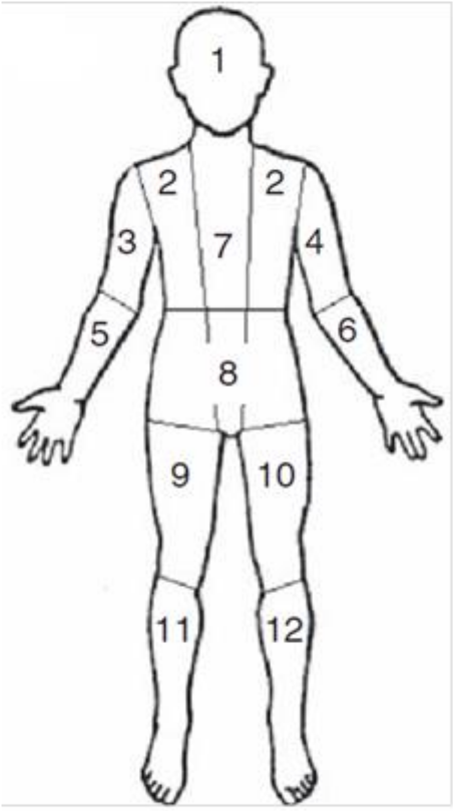

Figure 6: The SIOPEN Method

The CURIE method: This method divides the skeleton into nine segments to view osteomedullary involvement, and adds a tenth sector that counts any soft tissue involvement to the score. The extension score for this method is graded as: 0. no sites per segment; 1. one site per segment; 2. more than one site per segment; and 3. diffuse involvement (>50% of the segment). The intensity score is graded as: 0. for no uptake; 1. for doubtful uptake; 2. for definite uptake less than liver; and 3. for intense uptake greater than that of liver. Thus, the maximum score for either extension or intensity would be 30. The Curie score has been shown to have a good inter-observer concordance of 92 and 95% in two independent studies (Matthay, Edeline, et al. 2003; Messina et al. 2006). It has been validated in France and is now widely used in North America for the New Approaches to Neuroblastoma Therapy consortium and the Children's Oncology Group (COG)(Ady et al. 1995).

The SIOPEN method: the skeletal distribution of MIBG is recorded in 12 anatomical body segments as follows: skull, thoracic cage, proximal right upper limb, distal right upper limb, proximal left upper limb, distal left upper limb, spine, pelvis, proximal right lower limb, distal right lower limb, proximal left lower limb and distal left lower limb. The extent and pattern of skeletal MIBG involvement is scored using a 0–6 scale to discriminate between focal discrete lesions and patterns of more diffuse infiltration. Each segment is scored as 0. no involvement; 1. one discrete lesion; 2. two discrete lesions; 3. three discrete lesions; 4. > 3 discrete foci or a single diffuse lesion involving < 50% of a bone; 5. diffuse involvement of > 50 to 95% of an entire bone; 6, diffuse involvement of the entire bone, with a maximum score of 72.

The SIOPEN score is the current method being used in Europe for the prospective phase 3 neuroblastoma trial. (Brisse et al. 2011; Matthay et al. 2010)

APPENDIX 7 – TEMOZOLOMIDE DOSING

These tables are to be followed where possible. In other circumstances, please round to nearest practical dose:

	Temozolomide 100mg/m²/day	
**BSA (m²)**	**Calculated Dose (mg)**	**Administered Dose (mg)**
.20 – .50	3.35 mg/kg/day	3.35 mg/kg/day
.51	51	50
.52 – .53	52 – 53	50
.54 - .56	54 – 56	55
.57 - .58	57 – 58	55
.59 - .61	59 – 61	60
.62 – .63	62 – 63	60
.64 - .66	64 – 66	65
.67 – .68	67 – 68	65
.69 - .71	69 – 71	70
.72 – .73	72 – 73	70
.74 - .76	74 – 76	75
.77 – .78	77 – 78	75
.79 - .81	79 – 81	80
.82 – .83	82 – 83	80
.84 - .86	84 – 86	85
.87 – .88	87 – 88	85
.89 - .91	89 – 91	90
.92 - .93	92 – 93	90
.94 - .96	94 – 96	95
.97 - .99	97 – 99	95

	Temozolomide 150mg/m²/day	
BSA (m²)	Calculated Dose (mg)	Administered Dose (mg)
.20 – .50	5.0 mg/kg/day	5.0 mg/kg/day
.51	77	75
.52 – .54	78 – 81	80
.55 - .58	83 – 87	85
.59 - .61	89 – 92	90
.62 - .64	93 – 96	95
.65 – .68	98 – 102	100
.69 - .71	104 – 107	105
.72 – .74	108 – 111	110
.75 - .78	113 – 117	115
.79 – .81	119 – 122	120
.82 - .84	123 – 126	125
.85 – .88	128 – 132	130
.89 - .91	134 – 137	135
.92 – .94	138 – 141	140
.95 - .98	143 – 147	145
.99	149	150

NB- Temozolomide only arm closed new randomisations on 28 Jan 2020.

	Temozolomide 200mg/m²/day	
**BSA (m²)**	**Calculated Dose (mg)**	**Administered Dose (mg)**
.20 – .50	6.7 mg/kg/day	6.7 mg/kg/day
.51	102	100
.52 – .53	104 – 106	105
.54 - .56	108 – 112	110
.57 - .58	114 – 116	115
.59 - .61	118 – 122	120
.62 – .63	124 – 126	125
.64 - .66	128 – 132	130
.67 – .68	134 – 136	135
.69 - .71	138 – 142	140
.72 – .73	144 – 146	145
.74 - .76	148 – 152	150
.77 – .78	154 – 156	155
.79 - .81	158 – 162	160
.82 – .83	164 – 166	165
.84 - .86	168 – 172	170
.87 – .88	174 – 176	175
.89 - .91	178 – 182	180
.92 - .93	184 – 186	185
.94 - .96	188 – 192	190
.97 - .99	194 – 196	195

APPENDIX 8 – BLOOD PRESSURE LEVELS BY AGE AND HEIGHT PERCENTILE FOR CHILDREN AND ADOLESCENTS

The following tables have been taken from The Fourth Report on the Diagnosis, Evaluation, and Treatment of High Blood Pressure in Children and Adolescents published by the US department of Health and Human Services, 2005
http://www.nhlbi.nih.gov/health/prof/heart/hbp/hbp_ped.pdf

Using the BP Tables

1. Use the standard height charts in Appendix 9 – Height for Age Chart - Girls or Appendix 10 – Height for Age Chart - Boys to determine the height percentile
2. Measure and record the child's SBP and DBP
3. Use the correct gender table for SBP and DBP
4. Find the child's age in the left side of the table. Follow the age row horizontally across the table to the intersection of the line for the height percentile (vertical column).
5. There, find the 50th, 90th, 95th and 99th percentiles for SBP in the left columns and for DBP in the right columns
    - BP less than the 90th percentile is normal
    - BP between the 90th and 95th percentile is pre hypertension. In adolescents, BP equal to or exceeding 120/80mmHg is pre hypertension, even if this figure is less than the 90th percentile
    - BP greater than the 95th percentile may be hypertension.
6. If the BP is greater than the 90th percentile, the BP should be repeated twice at the same office visit, and an average SBP and DBP should be used. If the BP is greater than the 95th percentile, BP should be staged. If stage I (95th percentile to the 99th percentile plus 5mmHg), BP measurements should be repeated on two more occasions.

Blood Pressure Levels for Girls by Age and Height Percentile*

Age (Year)	BP Percentile ↓	Systolic BP (mmHg) ← Percentile of Height → 5th	10th	25th	50th	75th	90th	95th	Diastolic BP (mmHg) ← Percentile of Height → 5th	10th	25th	50th	75th	90th	95th
1	50th	83	84	85	86	88	89	90	38	39	39	40	41	41	42
	90th	97	97	98	100	101	102	103	52	53	53	54	55	55	56
	95th	100	101	102	104	105	106	107	56	57	57	58	59	59	60
	99th	108	108	109	111	112	113	114	64	64	65	65	66	67	67
2	50th	85	85	87	88	89	91	91	43	44	44	45	46	46	47
	90th	98	99	100	101	103	104	105	57	58	58	59	60	61	61
	95th	102	103	104	105	107	108	109	61	62	62	63	64	65	65
	99th	109	110	111	112	114	115	116	69	69	70	70	71	72	72
3	50th	86	87	88	89	91	92	93	47	48	48	49	50	50	51
	90th	100	100	102	103	104	106	106	61	62	62	63	64	64	65
	95th	104	104	105	107	108	109	110	65	66	66	67	68	68	69
	99th	111	111	113	114	115	116	117	73	73	74	74	75	76	76
4	50th	88	88	90	91	92	94	94	50	50	51	52	52	53	54
	90th	101	102	103	104	106	107	108	64	64	65	66	67	67	68
	95th	105	106	107	108	110	111	112	68	68	69	70	71	71	72
	99th	112	113	114	115	117	118	119	76	76	76	77	78	79	79
5	50th	89	90	91	93	94	95	96	52	53	53	54	55	55	56
	90th	103	103	105	106	107	109	109	66	67	67	68	69	69	70
	95th	107	107	108	110	111	112	113	70	71	71	72	73	73	74
	99th	114	114	116	117	118	120	120	78	78	79	79	80	81	81
6	50th	91	92	93	94	96	97	98	54	54	55	56	56	57	58
	90th	104	105	106	108	109	110	111	68	68	69	70	70	71	72
	95th	108	109	110	111	113	114	115	72	72	73	74	74	75	76
	99th	115	116	117	119	120	121	122	80	80	80	81	82	83	83
7	50th	93	93	95	96	97	99	99	55	56	56	57	58	58	59
	90th	106	107	108	109	111	112	113	69	70	70	71	72	72	73
	95th	110	111	112	113	115	116	116	73	74	74	75	76	76	77
	99th	117	118	119	120	122	123	124	81	81	82	82	83	84	84
8	50th	95	95	96	98	99	100	101	57	57	57	58	59	60	60
	90th	108	109	110	111	113	114	114	71	71	71	72	73	74	74
	95th	112	112	114	115	116	118	118	75	75	75	76	77	78	78
	99th	119	120	121	122	123	125	125	82	82	83	83	84	85	86
9	50th	96	97	98	100	101	102	103	58	58	58	59	60	61	61
	90th	110	110	112	113	114	116	116	72	72	72	73	74	75	75
	95th	114	114	115	117	118	119	120	76	76	76	77	78	79	79
	99th	121	121	123	124	125	127	127	83	83	84	84	85	86	87
10	50th	98	99	100	102	103	104	105	59	59	59	60	61	62	62
	90th	112	112	114	115	116	118	118	73	73	73	74	75	76	76
	95th	116	116	117	119	120	121	122	77	77	77	78	79	80	80
	99th	123	123	125	126	127	129	129	84	84	85	86	86	87	88

Age (Year)	BP Percentile ↓	Systolic BP (mmHg) ← Percentile of Height →							Diastolic BP (mmHg) ← Percentile of Height →						
		5th	10th	25th	50th	75th	90th	95th	5th	10th	25th	50th	75th	90th	95th
11	50th	100	101	102	103	105	106	107	60	60	60	61	62	63	63
	90th	114	114	116	117	118	119	120	74	74	74	75	76	77	77
	95th	118	118	119	121	122	123	124	78	78	78	79	80	81	81
	99th	125	125	126	128	129	130	131	85	85	86	87	87	88	89
12	50th	102	103	104	105	107	108	109	61	61	61	62	63	64	64
	90th	116	116	117	119	120	121	122	75	75	75	76	77	78	78
	95th	119	120	121	123	124	125	126	79	79	79	80	81	82	82
	99th	127	127	128	130	131	132	133	86	86	87	88	88	89	90
13	50th	104	105	106	107	109	110	110	62	62	62	63	64	65	65
	90th	117	118	119	121	122	123	124	76	76	76	77	78	79	79
	95th	121	122	123	124	126	127	128	80	80	80	81	82	83	83
	99th	128	129	130	132	133	134	135	87	87	88	89	89	90	91
14	50th	106	106	107	109	110	111	112	63	63	63	64	65	66	66
	90th	119	120	121	122	124	125	125	77	77	77	78	79	80	80
	95th	123	123	125	126	127	129	129	81	81	81	82	83	84	84
	99th	130	131	132	133	135	136	136	88	88	89	90	90	91	92
15	50th	107	108	109	110	111	113	113	64	64	64	65	66	67	67
	90th	120	121	122	123	125	126	127	78	78	78	79	80	81	81
	95th	124	125	126	127	129	130	131	82	82	82	83	84	85	85
	99th	131	132	133	134	136	137	138	89	89	90	91	91	92	93
16	50th	108	108	110	111	112	114	114	64	64	65	66	66	67	68
	90th	121	122	123	124	126	127	128	78	78	79	80	81	81	82
	95th	125	126	127	128	130	131	132	82	82	83	84	85	85	86
	99th	132	133	134	135	137	138	139	90	90	90	91	92	93	93
17	50th	108	109	110	111	113	114	115	64	65	65	66	67	67	68
	90th	122	122	123	125	126	127	128	78	79	79	80	81	81	82
	95th	125	126	127	129	130	131	132	82	83	83	84	85	85	86
	99th	133	133	134	136	137	138	139	90	90	91	91	92	93	93

BP, blood pressure

* The 90th percentile is 1.28 SD, 95th percentile is 1.645 SD, and the 99th percentile is 2.326 SD over the mean. For research purposes, the standard deviations in appendix table B–1 allow one to compute BP Z-scores and percentiles for girls with height percentiles given in table 4 (i.e., the 5th, 10th, 25th, 50th, 75th, 90th, and 95th percentiles). These height percentiles must be converted to height Z-scores given by (5% = -1.645; 10% = -1.28; 25% = -0.68; 50% = 0; 75% = 0.68; 90% = 1.28; 95% = 1.645) and then computed according to the methodology in steps 2–4 described in appendix B. For children with height percentiles other than these, follow steps 1–4 as described in appendix B.

Blood Pressure Levels for Boys by Age and Height Percentile*

Age (Year)	BP Percentile ↓	Systolic BP (mmHg) ← Percentile of Height →							Diastolic BP (mmHg) ← Percentile of Height →						
		5th	10th	25th	50th	75th	90th	95th	5th	10th	25th	50th	75th	90th	95th
1	50th	80	81	83	85	87	88	89	34	35	36	37	38	39	39
	90th	94	95	97	99	100	102	103	49	50	51	52	53	53	54
	95th	98	99	101	103	104	106	106	54	54	55	56	57	58	58
	99th	105	106	108	110	112	113	114	61	62	63	64	65	66	66
2	50th	84	85	87	88	90	92	92	39	40	41	42	43	44	44
	90th	97	99	100	102	104	105	106	54	55	56	57	58	58	59
	95th	101	102	104	106	108	109	110	59	59	60	61	62	63	63
	99th	109	110	111	113	115	117	117	66	67	68	69	70	71	71
3	50th	86	87	89	91	93	94	95	44	44	45	46	47	48	48
	90th	100	101	103	105	107	108	109	59	59	60	61	62	63	63
	95th	104	105	107	109	110	112	113	63	63	64	65	66	67	67
	99th	111	112	114	116	118	119	120	71	71	72	73	74	75	75
4	50th	88	89	91	93	95	96	97	47	48	49	50	51	51	52
	90th	102	103	105	107	109	110	111	62	63	64	65	66	66	67
	95th	106	107	109	111	112	114	115	66	67	68	69	70	71	71
	99th	113	114	116	118	120	121	122	74	75	76	77	78	78	79
5	50th	90	91	93	95	96	98	98	50	51	52	53	54	55	55
	90th	104	105	106	108	110	111	112	65	66	67	68	69	69	70
	95th	108	109	110	112	114	115	116	69	70	71	72	73	74	74
	99th	115	116	118	120	121	123	123	77	78	79	80	81	81	82
6	50th	91	92	94	96	98	99	100	53	53	54	55	56	57	57
	90th	105	106	108	110	111	113	113	68	68	69	70	71	72	72
	95th	109	110	112	114	115	117	117	72	72	73	74	75	76	76
	99th	116	117	119	121	123	124	125	80	80	81	82	83	84	84
7	50th	92	94	95	97	99	100	101	55	55	56	57	58	59	59
	90th	106	107	109	111	113	114	115	70	70	71	72	73	74	74
	95th	110	111	113	115	117	118	119	74	74	75	76	77	78	78
	99th	117	118	120	122	124	125	126	82	82	83	84	85	86	86
8	50th	94	95	97	99	100	102	102	56	57	58	59	60	60	61
	90th	107	109	110	112	114	115	116	71	72	72	73	74	75	76
	95th	111	112	114	116	118	119	120	75	76	77	78	79	79	80
	99th	119	120	122	123	125	127	127	83	84	85	86	87	87	88
9	50th	95	96	98	100	102	103	104	57	58	59	60	61	61	62
	90th	109	110	112	114	115	117	118	72	73	74	75	76	76	77
	95th	113	114	116	118	119	121	121	76	77	78	79	80	81	81
	99th	120	121	123	125	127	128	129	84	85	86	87	88	88	89
10	50th	97	98	100	102	103	105	106	58	59	60	61	61	62	63
	90th	111	112	114	115	117	119	119	73	73	74	75	76	77	78
	95th	115	116	117	119	121	122	123	77	78	79	80	81	81	82
	99th	122	123	125	127	128	130	130	85	86	86	88	88	89	90

Age (Year)	BP Percentile ↓	Systolic BP (mmHg) ← Percentile of Height →							Diastolic BP (mmHg) ← Percentile of Height →						
		5th	10th	25th	50th	75th	90th	95th	5th	10th	25th	50th	75th	90th	95th
11	50th	99	100	102	104	105	107	107	59	59	60	61	62	63	63
	90th	113	114	115	117	119	120	121	74	74	75	76	77	78	78
	95th	117	118	119	121	123	124	125	78	78	79	80	81	82	82
	99th	124	125	127	129	130	132	132	86	86	87	88	89	90	90
12	50th	101	102	104	106	108	109	110	59	60	61	62	63	63	64
	90th	115	116	118	120	121	123	123	74	75	75	76	77	78	79
	95th	119	120	122	123	125	127	127	78	79	80	81	82	82	83
	99th	126	127	129	131	133	134	135	86	87	88	89	90	90	91
13	50th	104	105	106	108	110	111	112	60	60	61	62	63	64	64
	90th	117	118	120	122	124	125	126	75	75	76	77	78	79	79
	95th	121	122	124	126	128	129	130	79	79	80	81	82	83	83
	99th	128	130	131	133	135	136	137	87	87	88	89	90	91	91
14	50th	106	107	109	111	113	114	115	60	61	62	63	64	65	65
	90th	120	121	123	125	126	128	128	75	76	77	78	79	79	80
	95th	124	125	127	128	130	132	132	80	80	81	82	83	84	84
	99th	131	132	134	136	138	139	140	87	88	89	90	91	92	92
15	50th	109	110	112	113	115	117	117	61	62	63	64	65	66	66
	90th	122	124	125	127	129	130	131	76	77	78	79	80	80	81
	95th	126	127	129	131	133	134	135	81	81	82	83	84	85	85
	99th	134	135	136	138	140	142	142	88	89	90	91	92	93	93
16	50th	111	112	114	116	118	119	120	63	63	64	65	66	67	67
	90th	125	126	128	130	131	133	134	78	78	79	80	81	82	82
	95th	129	130	132	134	135	137	137	82	83	83	84	85	86	87
	99th	136	137	139	141	143	144	145	90	90	91	92	93	94	94
17	50th	114	115	116	118	120	121	122	65	66	66	67	68	69	70
	90th	127	128	130	132	134	135	136	80	80	81	82	83	84	84
	95th	131	132	134	136	138	139	140	84	85	86	87	87	88	89
	99th	139	140	141	143	145	146	147	92	93	93	94	95	96	97

BP, blood pressure

* The 90th percentile is 1.28 SD, 95th percentile is 1.645 SD, and the 99th percentile is 2.326 SD over the mean. For research purposes, the standard deviations in appendix table B–1 allow one to compute BP Z-scores and percentiles for boys with height percentiles given in table 3 (i.e., the 5th, 10th, 25th, 50th, 75th, 90th, and 95th percentiles). These height percentiles must be converted to height Z-scores given by (5% = -1.645; 10% = -1.28; 25% = -0.68; 50% = 0; 75% = 0.68; 90% = 1.28; 95% = 1.645) and then computed according to the methodology in steps 2–4 described in appendix B. For children with height percentiles other than these, follow steps 1–4 as described in appendix B.

APPENDIX 9 – HEIGHT FOR AGE CHART - GIRLS

Length-for-age GIRLS

Birth to 2 years (percentiles)

						Percentiles (length in cm)										
Year: Month	Month	L	M	S	SD	1st	3rd	5th	15th	25th	50th	75th	85th	95th	97th	99th
0: 0	0	1	49.1477	0.03790	1.8627	44.8	45.6	46.1	47.2	47.9	49.1	50.4	51.1	52.2	52.7	53.5
0: 1	1	1	53.6872	0.03640	1.9542	49.1	50.0	50.5	51.7	52.4	53.7	55.0	55.7	56.9	57.4	58.2
0: 2	2	1	57.0673	0.03568	2.0362	52.3	53.2	53.7	55.0	55.7	57.1	58.4	59.2	60.4	60.9	61.8
0: 3	3	1	59.8029	0.03520	2.1051	54.9	55.8	56.3	57.6	58.4	59.8	61.2	62.0	63.3	63.8	64.7
0: 4	4	1	62.0899	0.03486	2.1645	57.1	58.0	58.5	59.8	60.6	62.1	63.5	64.3	65.7	66.2	67.1
0: 5	5	1	64.0301	0.03463	2.2174	58.9	59.9	60.4	61.7	62.5	64.0	65.5	66.3	67.7	68.2	69.2
0: 6	6	1	65.7311	0.03448	2.2664	60.5	61.5	62.0	63.4	64.2	65.7	67.3	68.1	69.5	70.0	71.0
0: 7	7	1	67.2873	0.03441	2.3154	61.9	62.9	63.5	64.9	65.7	67.3	68.8	69.7	71.1	71.6	72.7
0: 8	8	1	68.7498	0.03440	2.3650	63.2	64.3	64.9	66.3	67.2	68.7	70.3	71.2	72.6	73.2	74.3
0: 9	9	1	70.1435	0.03444	2.4157	64.5	65.6	66.2	67.6	68.5	70.1	71.8	72.6	74.1	74.7	75.8
0:10	10	1	71.4818	0.03452	2.4676	65.7	66.8	67.4	68.9	69.8	71.5	73.1	74.0	75.5	76.1	77.2
0:11	11	1	72.7710	0.03464	2.5208	66.9	68.0	68.6	70.2	71.1	72.8	74.5	75.4	76.9	77.5	78.6
1: 0	12	1	74.0150	0.03479	2.5750	68.0	69.2	69.8	71.3	72.3	74.0	75.8	76.7	78.3	78.9	80.0
1: 1	13	1	75.2176	0.03496	2.6296	69.1	70.3	70.9	72.5	73.4	75.2	77.0	77.9	79.5	80.2	81.3
1: 2	14	1	76.3817	0.03514	2.6841	70.1	71.3	72.0	73.6	74.6	76.4	78.2	79.2	80.8	81.4	82.6
1: 3	15	1	77.5099	0.03534	2.7392	71.1	72.4	73.0	74.7	75.7	77.5	79.4	80.3	82.0	82.7	83.9
1: 4	16	1	78.6055	0.03555	2.7944	72.1	73.3	74.0	75.7	76.7	78.6	80.5	81.5	83.2	83.9	85.1
1: 5	17	1	79.6710	0.03576	2.8490	73.0	74.3	75.0	76.7	77.7	79.7	81.6	82.6	84.4	85.0	86.3
1: 6	18	1	80.7079	0.03598	2.9039	74.0	75.2	75.9	77.7	78.7	80.7	82.7	83.7	85.5	86.2	87.5
1: 7	19	1	81.7182	0.03620	2.9582	74.8	76.2	76.9	78.7	79.7	81.7	83.7	84.8	86.6	87.3	88.6
1: 8	20	1	82.7036	0.03643	3.0129	75.7	77.0	77.7	79.6	80.7	82.7	84.7	85.8	87.7	88.4	89.7
1: 9	21	1	83.6654	0.03666	3.0672	76.5	77.9	78.6	80.5	81.6	83.7	85.7	86.8	88.7	89.4	90.8
1:10	22	1	84.6040	0.03688	3.1202	77.3	78.7	79.5	81.4	82.5	84.6	86.7	87.8	89.7	90.5	91.9
1:11	23	1	85.5202	0.03711	3.1737	78.1	79.6	80.3	82.2	83.4	85.5	87.7	88.8	90.7	91.5	92.9
2: 0	24	1	86.4153	0.03734	3.2267	78.9	80.3	81.1	83.1	84.2	86.4	88.6	89.8	91.7	92.5	93.9

WHO Child Growth Standards

Height-for-age GIRLS

2 to 5 years (percentiles)

						Percentiles (height in cm)										
Year: Month	Month	L	M	S	SD	1st	3rd	5th	15th	25th	50th	75th	85th	95th	97th	99th
2: 0	24	1	85.7153	0.03764	3.2267	78.2	79.6	80.4	82.4	83.5	85.7	87.9	89.1	91.0	91.8	93.2
2: 1	25	1	86.5904	0.03786	3.2783	79.0	80.4	81.2	83.2	84.4	86.6	88.8	90.0	92.0	92.8	94.2
2: 2	26	1	87.4462	0.03808	3.3300	79.7	81.2	82.0	84.0	85.2	87.4	89.7	90.9	92.9	93.7	95.2
2: 3	27	1	88.2830	0.03830	3.3812	80.4	81.9	82.7	84.8	86.0	88.3	90.6	91.8	93.8	94.6	96.1
2: 4	28	1	89.1004	0.03851	3.4313	81.1	82.6	83.5	85.5	86.8	89.1	91.4	92.7	94.7	95.6	97.1
2: 5	29	1	89.8991	0.03872	3.4809	81.8	83.4	84.2	86.3	87.6	89.9	92.2	93.5	95.6	96.4	98.0
2: 6	30	1	90.6797	0.03893	3.5302	82.5	84.0	84.9	87.0	88.3	90.7	93.1	94.3	96.5	97.3	98.9
2: 7	31	1	91.4430	0.03913	3.5782	83.1	84.7	85.6	87.7	89.0	91.4	93.9	95.2	97.3	98.2	99.8
2: 8	32	1	92.1906	0.03933	3.6259	83.8	85.4	86.2	88.4	89.7	92.2	94.6	95.9	98.2	99.0	100.6
2: 9	33	1	92.9239	0.03952	3.6724	84.4	86.0	86.9	89.1	90.4	92.9	95.4	96.7	99.0	99.8	101.5
2:10	34	1	93.6444	0.03971	3.7186	85.0	86.7	87.5	89.8	91.1	93.6	96.2	97.5	99.8	100.6	102.3
2:11	35	1	94.3533	0.03989	3.7638	85.6	87.3	88.2	90.5	91.8	94.4	96.9	98.3	100.5	101.4	103.1
3: 0	36	1	95.0515	0.04006	3.8078	86.2	87.9	88.8	91.1	92.5	95.1	97.6	99.0	101.3	102.2	103.9
3: 1	37	1	95.7399	0.04024	3.8526	86.8	88.5	89.4	91.7	93.1	95.7	98.3	99.7	102.1	103.0	104.7
3: 2	38	1	96.4187	0.04041	3.8963	87.4	89.1	90.0	92.4	93.8	96.4	99.0	100.5	102.8	103.7	105.5
3: 3	39	1	97.0885	0.04057	3.9389	87.9	89.7	90.6	93.0	94.4	97.1	99.7	101.2	103.6	104.5	106.3
3: 4	40	1	97.7493	0.04073	3.9813	88.5	90.3	91.2	93.6	95.1	97.7	100.4	101.9	104.3	105.2	107.0
3: 5	41	1	98.4015	0.04089	4.0236	89.0	90.8	91.8	94.2	95.7	98.4	101.1	102.6	105.0	106.0	107.8
3: 6	42	1	99.0448	0.04105	4.0658	89.6	91.4	92.4	94.8	96.3	99.0	101.8	103.3	105.7	106.7	108.5
3: 7	43	1	99.6795	0.04120	4.1068	90.1	92.0	92.9	95.4	96.9	99.7	102.4	103.9	106.4	107.4	109.2
3: 8	44	1	100.3058	0.04135	4.1476	90.7	92.5	93.5	96.0	97.5	100.3	103.1	104.6	107.1	108.1	110.0
3: 9	45	1	100.9238	0.04150	4.1883	91.2	93.0	94.0	96.6	98.1	100.9	103.7	105.3	107.8	108.8	110.7
3:10	46	1	101.5337	0.04164	4.2279	91.7	93.6	94.6	97.2	98.7	101.5	104.4	105.9	108.5	109.5	111.4
3:11	47	1	102.1360	0.04179	4.2683	92.2	94.1	95.1	97.7	99.3	102.1	105.0	106.6	109.2	110.2	112.1
4: 0	48	1	102.7312	0.04193	4.3075	92.7	94.6	95.6	98.3	99.8	102.7	105.6	107.2	109.8	110.8	112.8

WHO Child Growth Standards

Height-for-age GIRLS

2 to 5 years (percentiles)

World Health Organization

Year: Month	Month	L	M	S	SD	Percentiles (height in cm)										
						1st	3rd	5th	15th	25th	50th	75th	85th	95th	97th	99th
4: 1	49	1	103.3197	0.04206	4.3456	93.2	95.1	96.2	98.8	100.4	103.3	106.3	107.8	110.5	111.5	113.4
4: 2	50	1	103.9021	0.04220	4.3847	93.7	95.7	96.7	99.4	100.9	103.9	106.9	108.4	111.1	112.1	114.1
4: 3	51	1	104.4786	0.04233	4.4226	94.2	96.2	97.2	99.9	101.5	104.5	107.5	109.1	111.8	112.8	114.8
4: 4	52	1	105.0494	0.04246	4.4604	94.7	96.7	97.7	100.4	102.0	105.0	108.1	109.7	112.4	113.4	115.4
4: 5	53	1	105.6148	0.04259	4.4981	95.2	97.2	98.2	101.0	102.6	105.6	108.6	110.3	113.0	114.1	116.1
4: 6	54	1	106.1748	0.04272	4.5358	95.6	97.6	98.7	101.5	103.1	106.2	109.2	110.9	113.6	114.7	116.7
4: 7	55	1	106.7295	0.04285	4.5734	96.1	98.1	99.2	102.0	103.6	106.7	109.8	111.5	114.3	115.3	117.4
4: 8	56	1	107.2788	0.04298	4.6108	96.6	98.6	99.7	102.5	104.2	107.3	110.4	112.1	114.9	116.0	118.0
4: 9	57	1	107.8227	0.04310	4.6472	97.0	99.1	100.2	103.0	104.7	107.8	111.0	112.6	115.5	116.6	118.6
4:10	58	1	108.3613	0.04322	4.6834	97.5	99.6	100.7	103.5	105.2	108.4	111.5	113.2	116.1	117.2	119.3
4:11	59	1	108.8948	0.04334	4.7195	97.9	100.0	101.1	104.0	105.7	108.9	112.1	113.8	116.7	117.8	119.9
5: 0	60	1	109.4233	0.04347	4.7566	98.4	100.5	101.6	104.5	106.2	109.4	112.6	114.4	117.2	118.4	120.5

WHO Child Growth Standards

Height-for-age GIRLS

5 to 19 years (percentiles)

World Health Organization

Year: Month	Month	L	M	S	SD	Percentiles (height in cm)										
						1st	3rd	5th	15th	25th	50th	75th	85th	95th	97th	99th
5: 1	61	1	109.6016	0.04355	4.7731	98.5	100.6	101.8	104.7	106.4	109.6	112.8	114.5	117.5	118.6	120.7
5: 2	62	1	110.1258	0.04364	4.8059	98.9	101.1	102.2	105.1	106.9	110.1	113.4	115.1	118.0	119.2	121.3
5: 3	63	1	110.6451	0.04373	4.8385	99.4	101.5	102.7	105.6	107.4	110.6	113.9	115.7	118.6	119.7	121.9
5: 4	64	1	111.1596	0.04382	4.8710	99.8	102.0	103.1	106.1	107.9	111.2	114.4	116.2	119.2	120.3	122.5
5: 5	65	1	111.6696	0.04390	4.9023	100.3	102.4	103.6	106.6	108.4	111.7	115.0	116.8	119.7	120.9	123.1
5: 6	66	1	112.1753	0.04399	4.9346	100.7	102.9	104.1	107.1	108.8	112.2	115.5	117.3	120.3	121.5	123.7
5: 7	67	1	112.6767	0.04407	4.9657	101.1	103.3	104.5	107.5	109.3	112.7	116.0	117.8	120.8	122.0	124.2
5: 8	68	1	113.1740	0.04415	4.9966	101.6	103.8	105.0	108.0	109.8	113.2	116.5	118.4	121.4	122.6	124.8
5: 9	69	1	113.6672	0.04423	5.0275	102.0	104.2	105.4	108.5	110.3	113.7	117.1	118.9	121.9	123.1	125.4
5:10	70	1	114.1565	0.04431	5.0583	102.4	104.6	105.8	108.9	110.7	114.2	117.6	119.4	122.5	123.7	125.9
5:11	71	1	114.6421	0.04439	5.0890	102.8	105.1	106.3	109.4	111.2	114.6	118.1	119.9	123.0	124.2	126.5
6: 0	72	1	115.1244	0.04447	5.1196	103.2	105.5	106.7	109.8	111.7	115.1	118.6	120.4	123.5	124.8	127.0
6: 1	73	1	115.6039	0.04454	5.1490	103.6	105.9	107.1	110.3	112.1	115.6	119.1	120.9	124.1	125.3	127.6
6: 2	74	1	116.0812	0.04461	5.1784	104.0	106.3	107.6	110.7	112.6	116.1	119.6	121.4	124.6	125.8	128.1
6: 3	75	1	116.5568	0.04469	5.2089	104.4	106.8	108.0	111.2	113.0	116.6	120.1	122.0	125.1	126.4	128.7
6: 4	76	1	117.0311	0.04475	5.2371	104.8	107.2	108.4	111.6	113.5	117.0	120.6	122.5	125.6	126.9	129.2
6: 5	77	1	117.5044	0.04482	5.2665	105.3	107.6	108.8	112.0	114.0	117.5	121.1	123.0	126.2	127.4	129.8
6: 6	78	1	117.9769	0.04489	5.2960	105.7	108.0	109.3	112.5	114.4	118.0	121.5	123.5	126.7	127.9	130.3
6: 7	79	1	118.4489	0.04495	5.3243	106.1	108.4	109.7	112.9	114.9	118.4	122.0	124.0	127.2	128.5	130.8
6: 8	80	1	118.9208	0.04502	5.3538	106.5	108.9	110.1	113.4	115.3	118.9	122.5	124.5	127.7	129.0	131.4
6: 9	81	1	119.3926	0.04508	5.3822	106.9	109.3	110.5	113.8	115.8	119.4	123.0	125.0	128.2	129.5	131.9
6:10	82	1	119.8648	0.04514	5.4107	107.3	109.7	111.0	114.3	116.2	119.9	123.5	125.5	128.8	130.0	132.5
6:11	83	1	120.3374	0.04520	5.4393	107.7	110.1	111.4	114.7	116.7	120.3	124.0	126.0	129.3	130.6	133.0
7: 0	84	1	120.8105	0.04525	5.4667	108.1	110.5	111.8	115.1	117.1	120.8	124.5	126.5	129.8	131.1	133.5
7: 1	85	1	121.2843	0.04531	5.4954	108.5	110.9	112.2	115.6	117.6	121.3	125.0	127.0	130.3	131.6	134.1
7: 2	86	1	121.7587	0.04536	5.5230	108.9	111.4	112.7	116.0	118.0	121.8	125.5	127.5	130.8	132.1	134.6

2007 WHO Reference

Height-for-age GIRLS

5 to 19 years (percentiles)

World Health Organization

Year: Month	Month	L	M	S	SD	Percentiles (height in cm) 1st	3rd	5th	15th	25th	50th	75th	85th	95th	97th	99th
7: 3	87	1	122.2338	0.04542	5.5519	109.3	111.8	113.1	116.5	118.5	122.2	126.0	128.0	131.4	132.7	135.1
7: 4	88	1	122.7098	0.04547	5.5796	109.7	112.2	113.5	116.9	118.9	122.7	126.5	128.5	131.9	133.2	135.7
7: 5	89	1	123.1868	0.04551	5.6062	110.1	112.6	114.0	117.4	119.4	123.2	127.0	129.0	132.4	133.7	136.2
7: 6	90	1	123.6646	0.04556	5.6342	110.6	113.1	114.4	117.8	119.9	123.7	127.5	129.5	132.9	134.3	136.8
7: 7	91	1	124.1435	0.04561	5.6622	111.0	113.5	114.8	118.3	120.3	124.1	128.0	130.0	133.5	134.8	137.3
7: 8	92	1	124.6234	0.04565	5.6891	111.4	113.9	115.3	118.7	120.8	124.6	128.5	130.5	134.0	135.3	137.9
7: 9	93	1	125.1045	0.04569	5.7160	111.8	114.4	115.7	119.2	121.2	125.1	129.0	131.0	134.5	135.9	138.4
7:10	94	1	125.5869	0.04573	5.7431	112.2	114.8	116.1	119.6	121.7	125.6	129.5	131.5	135.0	136.4	138.9
7:11	95	1	126.0706	0.04577	5.7703	112.6	115.2	116.6	120.1	122.2	126.1	130.0	132.1	135.6	136.9	139.5
8: 0	96	1	126.5558	0.04581	5.7975	113.1	115.7	117.0	120.5	122.6	126.6	130.5	132.6	136.1	137.5	140.0
8: 1	97	1	127.0424	0.04585	5.8249	113.5	116.1	117.5	121.0	123.1	127.0	131.0	133.1	136.6	138.0	140.6
8: 2	98	1	127.5304	0.04588	5.8511	113.9	116.5	117.9	121.5	123.6	127.5	131.5	133.6	137.2	138.5	141.1
8: 3	99	1	128.0199	0.04591	5.8774	114.3	117.0	118.4	121.9	124.1	128.0	132.0	134.1	137.7	139.1	141.7
8: 4	100	1	128.5109	0.04594	5.9038	114.8	117.4	118.8	122.4	124.5	128.5	132.5	134.6	138.2	139.6	142.2
8: 5	101	1	129.0035	0.04597	5.9303	115.2	117.9	119.2	122.9	125.0	129.0	133.0	135.2	138.8	140.2	142.8
8: 6	102	1	129.4975	0.04600	5.9569	115.6	118.3	119.7	123.3	125.5	129.5	133.5	135.7	139.3	140.7	143.4
8: 7	103	1	129.9932	0.04602	5.9823	116.1	118.7	120.2	123.8	126.0	130.0	134.0	136.2	139.8	141.2	143.9
8: 8	104	1	130.4904	0.04604	6.0078	116.5	119.2	120.6	124.3	126.4	130.5	134.5	136.7	140.4	141.8	144.5
8: 9	105	1	130.9891	0.04607	6.0347	117.0	119.6	121.1	124.7	126.9	131.0	135.1	137.2	140.9	142.3	145.0
8:10	106	1	131.4895	0.04608	6.0590	117.4	120.1	121.5	125.2	127.4	131.5	135.6	137.8	141.5	142.9	145.6
8:11	107	1	131.9912	0.04610	6.0848	117.8	120.5	122.0	125.7	127.9	132.0	136.1	138.3	142.0	143.4	146.1
9: 0	108	1	132.4944	0.04612	6.1106	118.3	121.0	122.4	126.2	128.4	132.5	136.6	138.8	142.5	144.0	146.7
9: 1	109	1	132.9989	0.04613	6.1352	118.7	121.5	122.9	126.6	128.9	133.0	137.1	139.4	143.1	144.5	147.3
9: 2	110	1	133.5046	0.04614	6.1599	119.2	121.9	123.4	127.1	129.4	133.5	137.7	139.9	143.6	145.1	147.8
9: 3	111	1	134.0118	0.04615	6.1846	119.6	122.4	123.8	127.6	129.8	134.0	138.2	140.4	144.2	145.6	148.4

2007 WHO Reference

Height-for-age GIRLS

5 to 19 years (percentiles)

World Health Organization

Year: Month	Month	L	M	S	SD	Percentiles (height in cm) 1st	3rd	5th	15th	25th	50th	75th	85th	95th	97th	99th
9: 4	112	1	134.5202	0.04616	6.2095	120.1	122.8	124.3	128.1	130.3	134.5	138.7	141.0	144.7	146.2	149.0
9: 5	113	1	135.0299	0.04616	6.2330	120.5	123.3	124.8	128.6	130.8	135.0	139.2	141.5	145.3	146.8	149.5
9: 6	114	1	135.5410	0.04617	6.2579	121.0	123.8	125.2	129.1	131.3	135.5	139.8	142.0	145.8	147.3	150.1
9: 7	115	1	136.0533	0.04617	6.2816	121.4	124.2	125.7	129.5	131.8	136.1	140.3	142.6	146.4	147.9	150.7
9: 8	116	1	136.5670	0.04616	6.3039	121.9	124.7	126.2	130.0	132.3	136.6	140.8	143.1	146.9	148.4	151.2
9: 9	117	1	137.0821	0.04616	6.3277	122.4	125.2	126.7	130.5	132.8	137.1	141.4	143.6	147.5	149.0	151.8
9:10	118	1	137.5987	0.04616	6.3516	122.8	125.7	127.2	131.0	133.3	137.6	141.9	144.2	148.0	149.5	152.4
9:11	119	1	138.1167	0.04615	6.3741	123.3	126.1	127.6	131.5	133.8	138.1	142.4	144.7	148.6	150.1	152.9
10: 0	120	1	138.6363	0.04614	6.3967	123.8	126.6	128.1	132.0	134.3	138.6	143.0	145.3	149.2	150.7	153.5
10: 1	121	1	139.1575	0.04612	6.4179	124.2	127.1	128.6	132.5	134.8	139.2	143.5	145.8	149.7	151.2	154.1
10: 2	122	1	139.6803	0.04611	6.4407	124.7	127.6	129.1	133.0	135.3	139.7	144.0	146.4	150.3	151.8	154.7
10: 3	123	1	140.2049	0.04609	6.4620	125.2	128.1	129.6	133.5	135.8	140.2	144.6	146.9	150.8	152.4	155.2
10: 4	124	1	140.7313	0.04607	6.4835	125.6	128.5	130.1	134.0	136.4	140.7	145.1	147.5	151.4	152.9	155.8
10: 5	125	1	141.2594	0.04605	6.5050	126.1	129.0	130.6	134.5	136.9	141.3	145.6	148.0	152.0	153.5	156.4
10: 6	126	1	141.7892	0.04603	6.5266	126.6	129.5	131.1	135.0	137.4	141.8	146.2	148.6	152.5	154.1	157.0
10: 7	127	1	142.3206	0.04600	6.5467	127.1	130.0	131.6	135.5	137.9	142.3	146.7	149.1	153.1	154.6	157.6
10: 8	128	1	142.8534	0.04597	6.5670	127.6	130.5	132.1	136.0	138.4	142.9	147.3	149.7	153.7	155.2	158.1
10: 9	129	1	143.3874	0.04594	6.5872	128.1	131.0	132.6	136.6	138.9	143.4	147.8	150.2	154.2	155.8	158.7
10:10	130	1	143.9222	0.04591	6.6075	128.6	131.5	133.1	137.1	139.5	143.9	148.4	150.8	154.8	156.3	159.3
10:11	131	1	144.4575	0.04588	6.6277	129.0	132.0	133.6	137.6	140.0	144.5	148.9	151.3	155.4	156.9	159.9
11: 0	132	1	144.9929	0.04584	6.6465	129.5	132.5	134.1	138.1	140.5	145.0	149.5	151.9	155.9	157.5	160.5
11: 1	133	1	145.5280	0.04580	6.6652	130.0	133.0	134.6	138.6	141.0	145.5	150.0	152.4	156.5	158.1	161.0
11: 2	134	1	146.0622	0.04576	6.6838	130.5	133.5	135.1	139.1	141.6	146.1	150.6	153.0	157.1	158.6	161.6
11: 3	135	1	146.5951	0.04571	6.7009	131.0	134.0	135.6	139.7	142.1	146.6	151.1	153.5	157.6	159.2	162.2

2007 WHO Reference

Height-for-age GIRLS

5 to 19 years (percentiles)

World Health Organization

						Percentiles (height in cm)										
Year: Month	Month	L	M	S	SD	1st	3rd	5th	15th	25th	50th	75th	85th	95th	97th	99th
11: 4	136	1	147.1262	0.04567	6.7193	131.5	134.5	136.1	140.2	142.6	147.1	151.7	154.1	158.2	159.8	162.8
11: 5	137	1	147.6548	0.04562	6.7360	132.0	135.0	136.6	140.7	143.1	147.7	152.2	154.6	158.7	160.3	163.3
11: 6	138	1	148.1804	0.04557	6.7526	132.5	135.5	137.1	141.2	143.6	148.2	152.7	155.2	159.3	160.9	163.9
11: 7	139	1	148.7023	0.04552	6.7689	133.0	136.0	137.6	141.7	144.1	148.7	153.3	155.7	159.8	161.4	164.4
11: 8	140	1	149.2197	0.04546	6.7835	133.4	136.5	138.1	142.2	144.6	149.2	153.8	156.3	160.4	162.0	165.0
11: 9	141	1	149.7322	0.04541	6.7993	133.9	136.9	138.5	142.7	145.1	149.7	154.3	156.8	160.9	162.5	165.6
11:10	142	1	150.2390	0.04535	6.8133	134.4	137.4	139.0	143.2	145.6	150.2	154.8	157.3	161.4	163.1	166.1
11:11	143	1	150.7394	0.04529	6.8270	134.9	137.9	139.5	143.7	146.1	150.7	155.3	157.8	162.0	163.6	166.6
12: 0	144	1	151.2327	0.04523	6.8403	135.3	138.4	140.0	144.1	146.6	151.2	155.8	158.3	162.5	164.1	167.1
12: 1	145	1	151.7182	0.04516	6.8516	135.8	138.8	140.4	144.6	147.1	151.7	156.3	158.8	163.0	164.6	167.7
12: 2	146	1	152.1951	0.04510	6.8640	136.2	139.3	140.9	145.1	147.6	152.2	156.8	159.3	163.5	165.1	168.2
12: 3	147	1	152.6628	0.04503	6.8744	136.7	139.7	141.4	145.5	148.0	152.7	157.3	159.8	164.0	165.6	168.7
12: 4	148	1	153.1206	0.04497	6.8858	137.1	140.2	141.8	146.0	148.5	153.1	157.8	160.3	164.4	166.1	169.1
12: 5	149	1	153.5678	0.04490	6.8952	137.5	140.6	142.2	146.4	148.9	153.6	158.2	160.7	164.9	166.5	169.6
12: 6	150	1	154.0041	0.04483	6.9040	137.9	141.0	142.6	146.8	149.3	154.0	158.7	161.2	165.4	167.0	170.1
12: 7	151	1	154.4290	0.04476	6.9122	138.3	141.4	143.1	147.3	149.8	154.4	159.1	161.6	165.8	167.4	170.5
12: 8	152	1	154.8423	0.04468	6.9184	138.7	141.8	143.5	147.7	150.2	154.8	159.5	162.0	166.2	167.9	170.9
12: 9	153	1	155.2437	0.04461	6.9254	139.1	142.2	143.9	148.1	150.6	155.2	159.9	162.4	166.6	168.3	171.4
12:10	154	1	155.6330	0.04454	6.9319	139.5	142.6	144.2	148.4	151.0	155.6	160.3	162.8	167.0	168.7	171.8
12:11	155	1	156.0101	0.04446	6.9362	139.9	143.0	144.6	148.8	151.3	156.0	160.7	163.2	167.4	169.1	172.1
13: 0	156	1	156.3748	0.04439	6.9415	140.2	143.3	145.0	149.2	151.7	156.4	161.1	163.6	167.8	169.4	172.5
13: 1	157	1	156.7269	0.04431	6.9446	140.6	143.7	145.3	149.5	152.0	156.7	161.4	163.9	168.2	169.8	172.9
13: 2	158	1	157.0666	0.04423	6.9471	140.9	144.0	145.6	149.9	152.4	157.1	161.8	164.3	168.5	170.1	173.2
13: 3	159	1	157.3936	0.04415	6.9489	141.2	144.3	146.0	150.2	152.7	157.4	162.1	164.6	168.8	170.5	173.6

2007 WHO Reference

Height-for-age GIRLS

5 to 19 years (percentiles)

World Health Organization

						Percentiles (height in cm)										
Year: Month	Month	L	M	S	SD	1st	3rd	5th	15th	25th	50th	75th	85th	95th	97th	99th
13: 4	160	1	157.7082	0.04408	6.9518	141.5	144.6	146.3	150.5	153.0	157.7	162.4	164.9	169.1	170.8	173.9
13: 5	161	1	158.0102	0.04400	6.9524	141.8	144.9	146.6	150.8	153.3	158.0	162.7	165.2	169.4	171.1	174.2
13: 6	162	1	158.2997	0.04392	6.9525	142.1	145.2	146.9	151.1	153.6	158.3	163.0	165.5	169.7	171.4	174.5
13: 7	163	1	158.5771	0.04384	6.9520	142.4	145.5	147.1	151.4	153.9	158.6	163.3	165.8	170.0	171.7	174.8
13: 8	164	1	158.8425	0.04376	6.9509	142.7	145.8	147.4	151.6	154.2	158.8	163.5	166.0	170.3	171.9	175.0
13: 9	165	1	159.0961	0.04369	6.9509	142.9	146.0	147.7	151.9	154.4	159.1	163.8	166.3	170.5	172.2	175.3
13:10	166	1	159.3382	0.04361	6.9487	143.2	146.3	147.9	152.1	154.7	159.3	164.0	166.5	170.8	172.4	175.5
13:11	167	1	159.5691	0.04353	6.9460	143.4	146.5	148.1	152.4	154.9	159.6	164.3	166.8	171.0	172.6	175.7
14: 0	168	1	159.7890	0.04345	6.9428	143.6	146.7	148.4	152.6	155.1	159.8	164.5	167.0	171.2	172.8	175.9
14: 1	169	1	159.9983	0.04337	6.9391	143.9	146.9	148.6	152.8	155.3	160.0	164.7	167.2	171.4	173.0	176.1
14: 2	170	1	160.1971	0.04330	6.9365	144.1	147.2	148.8	153.0	155.5	160.2	164.9	167.4	171.6	173.2	176.3
14: 3	171	1	160.3857	0.04322	6.9319	144.3	147.3	149.0	153.2	155.7	160.4	165.1	167.6	171.8	173.4	176.5
14: 4	172	1	160.5643	0.04314	6.9267	144.5	147.5	149.2	153.4	155.9	160.6	165.2	167.7	172.0	173.6	176.7
14: 5	173	1	160.7332	0.04307	6.9228	144.6	147.7	149.3	153.6	156.1	160.7	165.4	167.9	172.1	173.8	176.8
14: 6	174	1	160.8927	0.04299	6.9168	144.8	147.9	149.5	153.7	156.2	160.9	165.6	168.1	172.3	173.9	177.0
14: 7	175	1	161.0430	0.04292	6.9120	145.0	148.0	149.7	153.9	156.4	161.0	165.7	168.2	172.4	174.0	177.1
14: 8	176	1	161.1845	0.04284	6.9051	145.1	148.2	149.8	154.0	156.5	161.2	165.8	168.3	172.5	174.2	177.2
14: 9	177	1	161.3176	0.04277	6.8996	145.3	148.3	150.0	154.2	156.7	161.3	166.0	168.5	172.7	174.3	177.4
14:10	178	1	161.4425	0.04270	6.8936	145.4	148.5	150.1	154.3	156.8	161.4	166.1	168.6	172.8	174.4	177.5
14:11	179	1	161.5596	0.04263	6.8873	145.5	148.6	150.2	154.4	156.9	161.6	166.2	168.7	172.9	174.5	177.6
15: 0	180	1	161.6692	0.04255	6.8790	145.7	148.7	150.4	154.5	157.0	161.7	166.3	168.8	173.0	174.6	177.7
15: 1	181	1	161.7717	0.04248	6.8721	145.8	148.8	150.5	154.6	157.1	161.8	166.4	168.9	173.1	174.7	177.8
15: 2	182	1	161.8673	0.04241	6.8648	145.9	149.0	150.6	154.8	157.2	161.9	166.5	169.0	173.2	174.8	177.8
15: 3	183	1	161.9564	0.04235	6.8589	146.0	149.1	150.7	154.8	157.3	162.0	166.6	169.1	173.2	174.9	177.9

2007 WHO Reference

Height-for-age GIRLS

5 to 19 years (percentiles)

World Health Organization

Year: Month	Month	L	M	S	SD	Percentiles (height in cm) 1st	3rd	5th	15th	25th	50th	75th	85th	95th	97th	99th
15: 4	184	1	162.0393	0.04228	6.8510	146.1	149.2	150.8	154.9	157.4	162.0	166.7	169.1	173.3	174.9	178.0
15: 5	185	1	162.1164	0.04221	6.8429	146.2	149.2	150.9	155.0	157.5	162.1	166.7	169.2	173.4	175.0	178.0
15: 6	186	1	162.1880	0.04214	6.8346	146.3	149.3	150.9	155.1	157.6	162.2	166.8	169.3	173.4	175.0	178.1
15: 7	187	1	162.2542	0.04208	6.8277	146.4	149.4	151.0	155.2	157.6	162.3	166.9	169.3	173.5	175.1	178.1
15: 8	188	1	162.3154	0.04201	6.8189	146.5	149.5	151.1	155.2	157.7	162.3	166.9	169.4	173.5	175.1	178.2
15: 9	189	1	162.3719	0.04195	6.8115	146.5	149.6	151.2	155.3	157.8	162.4	167.0	169.4	173.6	175.2	178.2
15:10	190	1	162.4239	0.04189	6.8039	146.6	149.6	151.2	155.4	157.8	162.4	167.0	169.5	173.6	175.2	178.3
15:11	191	1	162.4717	0.04182	6.7946	146.7	149.7	151.3	155.4	157.9	162.5	167.1	169.5	173.6	175.3	178.3
16: 0	192	1	162.5156	0.04176	6.7867	146.7	149.8	151.4	155.5	157.9	162.5	167.1	169.6	173.7	175.3	178.3
16: 1	193	1	162.5560	0.04170	6.7786	146.8	149.8	151.4	155.5	158.0	162.6	167.1	169.6	173.7	175.3	178.3
16: 2	194	1	162.5933	0.04164	6.7704	146.8	149.9	151.5	155.6	158.0	162.6	167.2	169.6	173.7	175.3	178.3
16: 3	195	1	162.6276	0.04158	6.7621	146.9	149.9	151.5	155.6	158.1	162.6	167.2	169.6	173.8	175.3	178.4
16: 4	196	1	162.6594	0.04152	6.7536	146.9	150.0	151.6	155.7	158.1	162.7	167.2	169.7	173.8	175.4	178.4
16: 5	197	1	162.6890	0.04147	6.7467	147.0	150.0	151.6	155.7	158.1	162.7	167.2	169.7	173.8	175.4	178.4
16: 6	198	1	162.7165	0.04141	6.7381	147.0	150.0	151.6	155.7	158.2	162.7	167.3	169.7	173.8	175.4	178.4
16: 7	199	1	162.7425	0.04136	6.7310	147.1	150.1	151.7	155.8	158.2	162.7	167.3	169.7	173.8	175.4	178.4
16: 8	200	1	162.7670	0.04130	6.7223	147.1	150.1	151.7	155.8	158.2	162.8	167.3	169.7	173.8	175.4	178.4
16: 9	201	1	162.7904	0.04125	6.7151	147.2	150.2	151.7	155.8	158.3	162.8	167.3	169.8	173.8	175.4	178.4
16:10	202	1	162.8126	0.04119	6.7063	147.2	150.2	151.8	155.9	158.3	162.8	167.3	169.8	173.8	175.4	178.4
16:11	203	1	162.8340	0.04114	6.6990	147.3	150.2	151.8	155.9	158.3	162.8	167.4	169.8	173.9	175.4	178.4
17: 0	204	1	162.8545	0.04109	6.6917	147.3	150.3	151.8	155.9	158.3	162.9	167.4	169.8	173.9	175.4	178.4
17: 1	205	1	162.8743	0.04104	6.6844	147.3	150.3	151.9	155.9	158.4	162.9	167.4	169.8	173.9	175.4	178.4
17: 2	206	1	162.8935	0.04099	6.6770	147.4	150.3	151.9	156.0	158.4	162.9	167.4	169.8	173.9	175.5	178.4
17: 3	207	1	162.9120	0.04094	6.6696	147.4	150.4	151.9	156.0	158.4	162.9	167.4	169.8	173.9	175.5	178.4

2007 WHO Reference

Height-for-age GIRLS

5 to 19 years (percentiles)

World Health Organization

Year: Month	Month	L	M	S	SD	Percentiles (height in cm) 1st	3rd	5th	15th	25th	50th	75th	85th	95th	97th	99th
17: 4	208	1	162.9300	0.04089	6.6622	147.4	150.4	152.0	156.0	158.4	162.9	167.4	169.8	173.9	175.5	178.4
17: 5	209	1	162.9476	0.04084	6.6548	147.5	150.4	152.0	156.1	158.5	162.9	167.4	169.8	173.9	175.5	178.4
17: 6	210	1	162.9649	0.04080	6.6490	147.5	150.5	152.0	156.1	158.5	163.0	167.5	169.9	173.9	175.5	178.4
17: 7	211	1	162.9817	0.04075	6.6415	147.5	150.5	152.1	156.1	158.5	163.0	167.5	169.9	173.9	175.5	178.4
17: 8	212	1	162.9983	0.04071	6.6357	147.6	150.5	152.1	156.1	158.5	163.0	167.5	169.9	173.9	175.5	178.4
17: 9	213	1	163.0144	0.04066	6.6282	147.6	150.5	152.1	156.1	158.5	163.0	167.5	169.9	173.9	175.5	178.4
17:10	214	1	163.0300	0.04062	6.6223	147.6	150.6	152.1	156.2	158.6	163.0	167.5	169.9	173.9	175.5	178.4
17:11	215	1	163.0451	0.04058	6.6164	147.7	150.6	152.2	156.2	158.6	163.0	167.5	169.9	173.9	175.5	178.4
18: 0	216	1	163.0595	0.04053	6.6088	147.7	150.6	152.2	156.2	158.6	163.1	167.5	169.9	173.9	175.5	178.4
18: 1	217	1	163.0733	0.04049	6.6028	147.7	150.7	152.2	156.2	158.6	163.1	167.5	169.9	173.9	175.5	178.4
18: 2	218	1	163.0862	0.04045	6.5968	147.7	150.7	152.2	156.2	158.6	163.1	167.5	169.9	173.9	175.5	178.4
18: 3	219	1	163.0982	0.04041	6.5908	147.8	150.7	152.3	156.3	158.7	163.1	167.5	169.9	173.9	175.5	178.4
18: 4	220	1	163.1092	0.04037	6.5847	147.8	150.7	152.3	156.3	158.7	163.1	167.6	169.9	173.9	175.5	178.4
18: 5	221	1	163.1192	0.04034	6.5802	147.8	150.7	152.3	156.3	158.7	163.1	167.6	169.9	173.9	175.5	178.4
18: 6	222	1	163.1279	0.04030	6.5741	147.8	150.8	152.3	156.3	158.7	163.1	167.6	169.9	173.9	175.5	178.4
18: 7	223	1	163.1355	0.04026	6.5678	147.9	150.8	152.3	156.3	158.7	163.1	167.6	169.9	173.9	175.5	178.4
18: 8	224	1	163.1418	0.04023	6.5632	147.9	150.8	152.3	156.3	158.7	163.1	167.6	169.9	173.9	175.5	178.4
18: 9	225	1	163.1469	0.04019	6.5569	147.9	150.8	152.4	156.4	158.7	163.1	167.6	169.9	173.9	175.5	178.4
18:10	226	1	163.1508	0.04016	6.5521	147.9	150.8	152.4	156.4	158.7	163.2	167.6	169.9	173.9	175.5	178.4
18:11	227	1	163.1534	0.04012	6.5457	147.9	150.8	152.4	156.4	158.7	163.2	167.6	169.9	173.9	175.5	178.4
19: 0	228	1	163.1548	0.04009	6.5409	147.9	150.9	152.4	156.4	158.7	163.2	167.6	169.9	173.9	175.5	178.4

2007 WHO Reference

APPENDIX 10 – HEIGHT FOR AGE CHART - BOYS

Length-for-age BOYS

Birth to 2 years (percentiles)

World Health Organization

						Percentiles (length in cm)										
Year: Month	Month	L	M	S	SD	1st	3rd	5th	15th	25th	50th	75th	85th	95th	97th	99th
0: 0	0	1	49.8842	0.03795	1.8931	45.5	46.3	46.8	47.9	48.6	49.9	51.2	51.8	53.0	53.4	54.3
0: 1	1	1	54.7244	0.03557	1.9465	50.2	51.1	51.5	52.7	53.4	54.7	56.0	56.7	57.9	58.4	59.3
0: 2	2	1	58.4249	0.03424	2.0005	53.8	54.7	55.1	56.4	57.1	58.4	59.8	60.5	61.7	62.2	63.1
0: 3	3	1	61.4292	0.03328	2.0444	56.7	57.6	58.1	59.3	60.1	61.4	62.8	63.5	64.8	65.3	66.2
0: 4	4	1	63.8860	0.03257	2.0808	59.0	60.0	60.5	61.7	62.5	63.9	65.3	66.0	67.3	67.8	68.7
0: 5	5	1	65.9026	0.03204	2.1115	61.0	61.9	62.4	63.7	64.5	65.9	67.3	68.1	69.4	69.9	70.8
0: 6	6	1	67.6236	0.03165	2.1403	62.6	63.6	64.1	65.4	66.2	67.6	69.1	69.8	71.1	71.6	72.6
0: 7	7	1	69.1645	0.03139	2.1711	64.1	65.1	65.6	66.9	67.7	69.2	70.6	71.4	72.7	73.2	74.2
0: 8	8	1	70.5994	0.03124	2.2055	65.5	66.5	67.0	68.3	69.1	70.6	72.1	72.9	74.2	74.7	75.7
0: 9	9	1	71.9687	0.03117	2.2433	66.8	67.7	68.3	69.6	70.5	72.0	73.5	74.3	75.7	76.2	77.2
0:10	10	1	73.2812	0.03118	2.2849	68.0	69.0	69.5	70.9	71.7	73.3	74.8	75.6	77.0	77.6	78.6
0:11	11	1	74.5388	0.03125	2.3293	69.1	70.2	70.7	72.1	73.0	74.5	76.1	77.0	78.4	78.9	80.0
1: 0	12	1	75.7488	0.03137	2.3762	70.2	71.3	71.8	73.3	74.1	75.7	77.4	78.2	79.7	80.2	81.3
1: 1	13	1	76.9186	0.03154	2.4260	71.3	72.4	72.9	74.4	75.3	76.9	78.6	79.4	80.9	81.5	82.6
1: 2	14	1	78.0497	0.03174	2.4773	72.3	73.4	74.0	75.5	76.4	78.0	79.7	80.6	82.1	82.7	83.8
1: 3	15	1	79.1458	0.03197	2.5303	73.3	74.4	75.0	76.5	77.4	79.1	80.9	81.8	83.3	83.9	85.0
1: 4	16	1	80.2113	0.03222	2.5844	74.2	75.4	76.0	77.5	78.5	80.2	82.0	82.9	84.5	85.1	86.2
1: 5	17	1	81.2487	0.03250	2.6406	75.1	76.3	76.9	78.5	79.5	81.2	83.0	84.0	85.6	86.2	87.4
1: 6	18	1	82.2587	0.03279	2.6973	76.0	77.2	77.8	79.5	80.4	82.3	84.1	85.1	86.7	87.3	88.5
1: 7	19	1	83.2418	0.03310	2.7553	76.8	78.1	78.7	80.4	81.4	83.2	85.1	86.1	87.8	88.4	89.7
1: 8	20	1	84.1996	0.03342	2.8140	77.7	78.9	79.6	81.3	82.3	84.2	86.1	87.1	88.8	89.5	90.7
1: 9	21	1	85.1348	0.03376	2.8742	78.4	79.7	80.4	82.2	83.2	85.1	87.1	88.1	89.9	90.5	91.8
1:10	22	1	86.0477	0.03410	2.9342	79.2	80.5	81.2	83.0	84.1	86.0	88.0	89.1	90.9	91.6	92.9
1:11	23	1	86.9410	0.03445	2.9951	80.0	81.3	82.0	83.8	84.9	86.9	89.0	90.0	91.9	92.6	93.9
2: 0	24	1	87.8161	0.03479	3.0551	80.7	82.1	82.8	84.6	85.8	87.8	89.9	91.0	92.8	93.6	94.9

WHO Child Growth Standards

Height-for-age BOYS

2 to 5 years (percentiles)

World Health Organization

						Percentiles (height in cm)										
Year: Month	Month	L	M	S	SD	1st	3rd	5th	15th	25th	50th	75th	85th	95th	97th	99th
2: 0	24	1	87.1161	0.03507	3.0551	80.0	81.4	82.1	83.9	85.1	87.1	89.2	90.3	92.1	92.9	94.2
2: 1	25	1	87.9720	0.03542	3.1160	80.7	82.1	82.8	84.7	85.9	88.0	90.1	91.2	93.1	93.8	95.2
2: 2	26	1	88.8065	0.03576	3.1757	81.4	82.8	83.6	85.5	86.7	88.8	90.9	92.1	94.0	94.8	96.2
2: 3	27	1	89.6197	0.03610	3.2353	82.1	83.5	84.3	86.3	87.4	89.6	91.8	93.0	94.9	95.7	97.1
2: 4	28	1	90.4120	0.03642	3.2928	82.8	84.2	85.0	87.0	88.2	90.4	92.6	93.8	95.8	96.6	98.1
2: 5	29	1	91.1828	0.03674	3.3501	83.4	84.9	85.7	87.7	88.9	91.2	93.4	94.7	96.7	97.5	99.0
2: 6	30	1	91.9327	0.03704	3.4052	84.0	85.5	86.3	88.4	89.6	91.9	94.2	95.5	97.5	98.3	99.9
2: 7	31	1	92.6631	0.03733	3.4591	84.6	86.2	87.0	89.1	90.3	92.7	95.0	96.2	98.4	99.2	100.7
2: 8	32	1	93.3753	0.03761	3.5118	85.2	86.8	87.6	89.7	91.0	93.4	95.7	97.0	99.2	100.0	101.5
2: 9	33	1	94.0711	0.03787	3.5625	85.8	87.4	88.2	90.4	91.7	94.1	96.5	97.8	99.9	100.8	102.4
2:10	34	1	94.7532	0.03812	3.6120	86.4	88.0	88.8	91.0	92.3	94.8	97.2	98.5	100.7	101.5	103.2
2:11	35	1	95.4236	0.03836	3.6604	86.9	88.5	89.4	91.6	93.0	95.4	97.9	99.2	101.4	102.3	103.9
3: 0	36	1	96.0835	0.03858	3.7069	87.5	89.1	90.0	92.2	93.6	96.1	98.6	99.9	102.2	103.1	104.7
3: 1	37	1	96.7337	0.03879	3.7523	88.0	89.7	90.6	92.8	94.2	96.7	99.3	100.6	102.9	103.8	105.5
3: 2	38	1	97.3749	0.03900	3.7976	88.5	90.2	91.1	93.4	94.8	97.4	99.9	101.3	103.6	104.5	106.2
3: 3	39	1	98.0073	0.03919	3.8409	89.1	90.8	91.7	94.0	95.4	98.0	100.6	102.0	104.3	105.2	106.9
3: 4	40	1	98.6310	0.03937	3.8831	89.6	91.3	92.2	94.6	96.0	98.6	101.3	102.7	105.0	105.9	107.7
3: 5	41	1	99.2459	0.03954	3.9242	90.1	91.9	92.8	95.2	96.6	99.2	101.9	103.3	105.7	106.6	108.4
3: 6	42	1	99.8515	0.03971	3.9651	90.6	92.4	93.3	95.7	97.2	99.9	102.5	104.0	106.4	107.3	109.1
3: 7	43	1	100.4485	0.03986	4.0039	91.1	92.9	93.9	96.3	97.7	100.4	103.1	104.6	107.0	108.0	109.8
3: 8	44	1	101.0374	0.04002	4.0435	91.6	93.4	94.4	96.8	98.3	101.0	103.8	105.2	107.7	108.6	110.4
3: 9	45	1	101.6186	0.04016	4.0810	92.1	93.9	94.9	97.4	98.9	101.6	104.4	105.8	108.3	109.3	111.1
3:10	46	1	102.1933	0.04031	4.1194	92.6	94.4	95.4	97.9	99.4	102.2	105.0	106.5	109.0	109.9	111.8
3:11	47	1	102.7625	0.04045	4.1567	93.1	94.9	95.9	98.5	100.0	102.8	105.6	107.1	109.6	110.6	112.4
4: 0	48	1	103.3273	0.04059	4.1941	93.6	95.4	96.4	99.0	100.5	103.3	106.2	107.7	110.2	111.2	113.1

WHO Child Growth Standards

Height-for-age BOYS

2 to 5 years (percentiles)

World Health Organization

Year: Month	Month	L	M	S	SD	Percentiles (height in cm) 1st	3rd	5th	15th	25th	50th	75th	85th	95th	97th	99th
4: 1	49	1	103.8886	0.04073	4.2314	94.0	95.9	96.9	99.5	101.0	103.9	106.7	108.3	110.8	111.8	113.7
4: 2	50	1	104.4473	0.04086	4.2677	94.5	96.4	97.4	100.0	101.6	104.4	107.3	108.9	111.5	112.5	114.4
4: 3	51	1	105.0041	0.04100	4.3052	95.0	96.9	97.9	100.5	102.1	105.0	107.9	109.5	112.1	113.1	115.0
4: 4	52	1	105.5596	0.04113	4.3417	95.5	97.4	98.4	101.1	102.6	105.6	108.5	110.1	112.7	113.7	115.7
4: 5	53	1	106.1138	0.04126	4.3783	95.9	97.9	98.9	101.6	103.2	106.1	109.1	110.7	113.3	114.3	116.3
4: 6	54	1	106.6668	0.04139	4.4149	96.4	98.4	99.4	102.1	103.7	106.7	109.6	111.2	113.9	115.0	116.9
4: 7	55	1	107.2188	0.04152	4.4517	96.9	98.8	99.9	102.6	104.2	107.2	110.2	111.8	114.5	115.6	117.6
4: 8	56	1	107.7697	0.04165	4.4886	97.3	99.3	100.4	103.1	104.7	107.8	110.8	112.4	115.2	116.2	118.2
4: 9	57	1	108.3198	0.04177	4.5245	97.8	99.8	100.9	103.6	105.3	108.3	111.4	113.0	115.8	116.8	118.8
4:10	58	1	108.8689	0.04190	4.5616	98.3	100.3	101.4	104.1	105.8	108.9	111.9	113.6	116.4	117.4	119.5
4:11	59	1	109.4170	0.04202	4.5977	98.7	100.8	101.9	104.7	106.3	109.4	112.5	114.2	117.0	118.1	120.1
5: 0	60	1	109.9638	0.04214	4.6339	99.2	101.2	102.3	105.2	106.8	110.0	113.1	114.8	117.6	118.7	120.7

WHO Child Growth Standards

Height-for-age BOYS

5 to 19 years (percentiles)

World Health Organization

Year: Month	Month	L	M	S	SD	Percentiles (height in cm) 1st	3rd	5th	15th	25th	50th	75th	85th	95th	97th	99th
5: 1	61	1	110.2647	0.04164	4.5914	99.6	101.6	102.7	105.5	107.2	110.3	113.4	115.0	117.8	118.9	120.9
5: 2	62	1	110.8006	0.04172	4.6226	100.0	102.1	103.2	106.0	107.7	110.8	113.9	115.6	118.4	119.5	121.6
5: 3	63	1	111.3338	0.04180	4.6538	100.5	102.6	103.7	106.5	108.2	111.3	114.5	116.2	119.0	120.1	122.2
5: 4	64	1	111.8636	0.04187	4.6837	101.0	103.1	104.2	107.0	108.7	111.9	115.0	116.7	119.6	120.7	122.8
5: 5	65	1	112.3895	0.04195	4.7147	101.4	103.5	104.6	107.5	109.2	112.4	115.6	117.3	120.1	121.3	123.4
5: 6	66	1	112.9110	0.04203	4.7456	101.9	104.0	105.1	108.0	109.7	112.9	116.1	117.8	120.7	121.8	124.0
5: 7	67	1	113.4280	0.04211	4.7765	102.3	104.4	105.6	108.5	110.2	113.4	116.7	118.4	121.3	122.4	124.5
5: 8	68	1	113.9410	0.04218	4.8060	102.8	104.9	106.0	109.0	110.7	113.9	117.2	118.9	121.8	123.0	125.1
5: 9	69	1	114.4500	0.04226	4.8367	103.2	105.4	106.5	109.4	111.2	114.5	117.7	119.5	122.4	123.5	125.7
5:10	70	1	114.9547	0.04234	4.8672	103.6	105.8	106.9	109.9	111.7	115.0	118.2	120.0	123.0	124.1	126.3
5:11	71	1	115.4549	0.04241	4.8964	104.1	106.2	107.4	110.4	112.2	115.5	118.8	120.5	123.5	124.7	126.8
6: 0	72	1	115.9509	0.04249	4.9268	104.5	106.7	107.8	110.8	112.6	116.0	119.3	121.1	124.1	125.2	127.4
6: 1	73	1	116.4432	0.04257	4.9570	104.9	107.1	108.3	111.3	113.1	116.4	119.8	121.6	124.6	125.8	128.0
6: 2	74	1	116.9325	0.04264	4.9860	105.3	107.6	108.7	111.8	113.6	116.9	120.3	122.1	125.1	126.3	128.5
6: 3	75	1	117.4196	0.04272	5.0162	105.8	108.0	109.2	112.2	114.0	117.4	120.8	122.6	125.7	126.9	129.1
6: 4	76	1	117.9046	0.04280	5.0463	106.2	108.4	109.6	112.7	114.5	117.9	121.3	123.1	126.2	127.4	129.6
6: 5	77	1	118.3880	0.04287	5.0753	106.6	108.8	110.0	113.1	115.0	118.4	121.8	123.6	126.7	127.9	130.2
6: 6	78	1	118.8700	0.04295	5.1055	107.0	109.3	110.5	113.6	115.4	118.9	122.3	124.2	127.3	128.5	130.7
6: 7	79	1	119.3508	0.04303	5.1357	107.4	109.7	110.9	114.0	115.9	119.4	122.8	124.7	127.8	129.0	131.3
6: 8	80	1	119.8303	0.04311	5.1659	107.8	110.1	111.3	114.5	116.3	119.8	123.3	125.2	128.3	129.5	131.8
6: 9	81	1	120.3085	0.04318	5.1949	108.2	110.5	111.8	114.9	116.8	120.3	123.8	125.7	128.9	130.1	132.4
6:10	82	1	120.7853	0.04326	5.2252	108.6	111.0	112.2	115.4	117.3	120.8	124.3	126.2	129.4	130.6	132.9
6:11	83	1	121.2604	0.04334	5.2554	109.0	111.4	112.6	115.8	117.7	121.3	124.8	126.7	129.9	131.1	133.5
7: 0	84	1	121.7338	0.04342	5.2857	109.4	111.8	113.0	116.3	118.2	121.7	125.3	127.2	130.4	131.7	134.0
7: 1	85	1	122.2053	0.04350	5.3159	109.8	112.2	113.5	116.7	118.6	122.2	125.8	127.7	130.9	132.2	134.6
7: 2	86	1	122.6750	0.04358	5.3462	110.2	112.6	113.9	117.1	119.1	122.7	126.3	128.2	131.5	132.7	135.1

2007 WHO Reference

Height-for-age BOYS

5 to 19 years (percentiles)

World Health Organization

Year: Month	Month	L	M	S	SD	Percentiles (height in cm) 1st	3rd	5th	15th	25th	50th	75th	85th	95th	97th	99th
7: 3	87	1	123.1429	0.04366	5.3764	110.6	113.0	114.3	117.6	119.5	123.1	126.8	128.7	132.0	133.3	135.7
7: 4	88	1	123.6092	0.04374	5.4067	111.0	113.4	114.7	118.0	120.0	123.6	127.3	129.2	132.5	133.8	136.2
7: 5	89	1	124.0736	0.04382	5.4369	111.4	113.8	115.1	118.4	120.4	124.1	127.7	129.7	133.0	134.3	136.7
7: 6	90	1	124.5361	0.04390	5.4671	111.8	114.3	115.5	118.9	120.8	124.5	128.2	130.2	133.5	134.8	137.3
7: 7	91	1	124.9964	0.04398	5.4973	112.2	114.7	116.0	119.3	121.3	125.0	128.7	130.7	134.0	135.3	137.8
7: 8	92	1	125.4545	0.04406	5.5275	112.6	115.1	116.4	119.7	121.7	125.5	129.2	131.2	134.5	135.9	138.3
7: 9	93	1	125.9104	0.04414	5.5577	113.0	115.5	116.8	120.2	122.2	125.9	129.7	131.7	135.1	136.4	138.8
7:10	94	1	126.3640	0.04422	5.5878	113.4	115.9	117.2	120.6	122.6	126.4	130.1	132.2	135.6	136.9	139.4
7:11	95	1	126.8156	0.04430	5.6179	113.7	116.2	117.6	121.0	123.0	126.8	130.6	132.6	136.1	137.4	139.9
8: 0	96	1	127.2651	0.04438	5.6480	114.1	116.6	118.0	121.4	123.5	127.3	131.1	133.1	136.6	137.9	140.4
8: 1	97	1	127.7129	0.04446	5.6781	114.5	117.0	118.4	121.8	123.9	127.7	131.5	133.6	137.1	138.4	140.9
8: 2	98	1	128.1590	0.04454	5.7082	114.9	117.4	118.8	122.2	124.3	128.2	132.0	134.1	137.5	138.9	141.4
8: 3	99	1	128.6034	0.04462	5.7383	115.3	117.8	119.2	122.7	124.7	128.6	132.5	134.6	138.0	139.4	142.0
8: 4	100	1	129.0466	0.04470	5.7684	115.6	118.2	119.6	123.1	125.2	129.0	132.9	135.0	138.5	139.9	142.5
8: 5	101	1	129.4887	0.04478	5.7985	116.0	118.6	120.0	123.5	125.6	129.5	133.4	135.5	139.0	140.4	143.0
8: 6	102	1	129.9300	0.04487	5.8300	116.4	119.0	120.3	123.9	126.0	129.9	133.9	136.0	139.5	140.9	143.5
8: 7	103	1	130.3705	0.04495	5.8602	116.7	119.3	120.7	124.3	126.4	130.4	134.3	136.4	140.0	141.4	144.0
8: 8	104	1	130.8103	0.04503	5.8904	117.1	119.7	121.1	124.7	126.8	130.8	134.8	136.9	140.5	141.9	144.5
8: 9	105	1	131.2495	0.04511	5.9207	117.5	120.1	121.5	125.1	127.3	131.3	135.2	137.4	141.0	142.4	145.0
8:10	106	1	131.6884	0.04519	5.9510	117.8	120.5	121.9	125.5	127.7	131.7	135.7	137.9	141.5	142.9	145.5
8:11	107	1	132.1269	0.04527	5.9814	118.2	120.9	122.3	125.9	128.1	132.1	136.2	138.3	142.0	143.4	146.0
9: 0	108	1	132.5652	0.04535	6.0118	118.6	121.3	122.7	126.3	128.5	132.6	136.6	138.8	142.5	143.9	146.6
9: 1	109	1	133.0031	0.04543	6.0423	118.9	121.6	123.1	126.7	128.9	133.0	137.1	139.3	142.9	144.4	147.1
9: 2	110	1	133.4404	0.04551	6.0729	119.3	122.0	123.5	127.1	129.3	133.4	137.5	139.7	143.4	144.9	147.6
9: 3	111	1	133.8770	0.04559	6.1035	119.7	122.4	123.8	127.6	129.8	133.9	138.0	140.2	143.9	145.4	148.1

2007 WHO Reference

Height-for-age BOYS

5 to 19 years (percentiles)

World Health Organization

Year: Month	Month	L	M	S	SD	Percentiles (height in cm) 1st	3rd	5th	15th	25th	50th	75th	85th	95th	97th	99th
9: 4	112	1	134.3130	0.04566	6.1327	120.0	122.8	124.2	128.0	130.2	134.3	138.4	140.7	144.4	145.8	148.6
9: 5	113	1	134.7483	0.04574	6.1634	120.4	123.2	124.6	128.4	130.6	134.7	138.9	141.1	144.9	146.3	149.1
9: 6	114	1	135.1829	0.04582	6.1941	120.8	123.5	125.0	128.8	131.0	135.2	139.4	141.6	145.4	146.8	149.6
9: 7	115	1	135.6168	0.04589	6.2235	121.1	123.9	125.4	129.2	131.4	135.6	139.8	142.1	145.9	147.3	150.1
9: 8	116	1	136.0501	0.04597	6.2542	121.5	124.3	125.8	129.6	131.8	136.1	140.3	142.5	146.3	147.8	150.6
9: 9	117	1	136.4829	0.04604	6.2837	121.9	124.7	126.1	130.0	132.2	136.5	140.7	143.0	146.8	148.3	151.1
9:10	118	1	136.9153	0.04612	6.3145	122.2	125.0	126.5	130.4	132.7	136.9	141.2	143.5	147.3	148.8	151.6
9:11	119	1	137.3474	0.04619	6.3441	122.6	125.4	126.9	130.8	133.1	137.3	141.6	143.9	147.8	149.3	152.1
10: 0	120	1	137.7795	0.04626	6.3737	123.0	125.8	127.3	131.2	133.5	137.8	142.1	144.4	148.3	149.8	152.6
10: 1	121	1	138.2119	0.04633	6.4034	123.3	126.2	127.7	131.6	133.9	138.2	142.5	144.8	148.7	150.3	153.1
10: 2	122	1	138.6452	0.04640	6.4331	123.7	126.5	128.1	132.0	134.3	138.6	143.0	145.3	149.2	150.7	153.6
10: 3	123	1	139.0797	0.04647	6.4630	124.0	126.9	128.4	132.4	134.7	139.1	143.4	145.8	149.7	151.2	154.1
10: 4	124	1	139.5158	0.04654	6.4931	124.4	127.3	128.8	132.8	135.1	139.5	143.9	146.2	150.2	151.7	154.6
10: 5	125	1	139.9540	0.04661	6.5233	124.8	127.7	129.2	133.2	135.6	140.0	144.4	146.7	150.7	152.2	155.1
10: 6	126	1	140.3948	0.04667	6.5522	125.2	128.1	129.6	133.6	136.0	140.4	144.8	147.2	151.2	152.7	155.6
10: 7	127	1	140.8387	0.04674	6.5828	125.5	128.5	130.0	134.0	136.4	140.8	145.3	147.7	151.7	153.2	156.2
10: 8	128	1	141.2859	0.04680	6.6122	125.9	128.9	130.4	134.4	136.8	141.3	145.7	148.1	152.2	153.7	156.7
10: 9	129	1	141.7368	0.04686	6.6418	126.3	129.2	130.8	134.9	137.3	141.7	146.2	148.6	152.7	154.2	157.2
10:10	130	1	142.1916	0.04692	6.6716	126.7	129.6	131.2	135.3	137.7	142.2	146.7	149.1	153.2	154.7	157.7
10:11	131	1	142.6501	0.04698	6.7017	127.1	130.0	131.6	135.7	138.1	142.7	147.2	149.6	153.7	155.3	158.2
11: 0	132	1	143.1126	0.04703	6.7306	127.5	130.5	132.0	136.1	138.6	143.1	147.7	150.1	154.2	155.8	158.8
11: 1	133	1	143.5795	0.04709	6.7612	127.9	130.9	132.5	136.6	139.0	143.6	148.1	150.6	154.7	156.3	159.3
11: 2	134	1	144.0511	0.04714	6.7906	128.3	131.3	132.9	137.0	139.5	144.1	148.6	151.1	155.2	156.8	159.8
11: 3	135	1	144.5276	0.04719	6.8203	128.7	131.7	133.3	137.5	139.9	144.5	149.1	151.6	155.7	157.4	160.4

2007 WHO Reference

Height-for-age BOYS

5 to 19 years (percentiles)

World Health Organization

						Percentiles (height in cm)										
Year: Month	Month	L	M	S	SD	1st	3rd	5th	15th	25th	50th	75th	85th	95th	97th	99th
11: 4	136	1	145.0093	0.04723	6.8488	129.1	132.1	133.7	137.9	140.4	145.0	149.6	152.1	156.3	157.9	160.9
11: 5	137	1	145.4964	0.04728	6.8791	129.5	132.6	134.2	138.4	140.9	145.5	150.1	152.6	156.8	158.4	161.5
11: 6	138	1	145.9891	0.04732	6.9082	129.9	133.0	134.6	138.8	141.3	146.0	150.6	153.1	157.4	159.0	162.1
11: 7	139	1	146.4878	0.04736	6.9377	130.3	133.4	135.1	139.3	141.8	146.5	151.2	153.7	157.9	159.5	162.6
11: 8	140	1	146.9927	0.04740	6.9675	130.8	133.9	135.5	139.8	142.3	147.0	151.7	154.2	158.5	160.1	163.2
11: 9	141	1	147.5041	0.04744	6.9976	131.2	134.3	136.0	140.3	142.8	147.5	152.2	154.8	159.0	160.7	163.8
11:10	142	1	148.0224	0.04747	7.0266	131.7	134.8	136.5	140.7	143.3	148.0	152.8	155.3	159.6	161.2	164.4
11:11	143	1	148.5478	0.04750	7.0560	132.1	135.3	136.9	141.2	143.8	148.5	153.3	155.9	160.2	161.8	165.0
12: 0	144	1	149.0807	0.04753	7.0858	132.6	135.8	137.4	141.7	144.3	149.1	153.9	156.4	160.7	162.4	165.6
12: 1	145	1	149.6212	0.04755	7.1145	133.1	136.2	137.9	142.2	144.8	149.6	154.4	157.0	161.3	163.0	166.2
12: 2	146	1	150.1694	0.04758	7.1451	133.5	136.7	138.4	142.8	145.4	150.2	155.0	157.6	161.9	163.6	166.8
12: 3	147	1	150.7256	0.04759	7.1730	134.0	137.2	138.9	143.3	145.9	150.7	155.6	158.2	162.5	164.2	167.4
12: 4	148	1	151.2899	0.04761	7.2029	134.5	137.7	139.4	143.8	146.4	151.3	156.1	158.8	163.1	164.8	168.0
12: 5	149	1	151.8623	0.04762	7.2317	135.0	138.3	140.0	144.4	147.0	151.9	156.7	159.4	163.8	165.5	168.7
12: 6	150	1	152.4425	0.04763	7.2608	135.6	138.8	140.5	144.9	147.5	152.4	157.3	160.0	164.4	166.1	169.3
12: 7	151	1	153.0298	0.04763	7.2888	136.1	139.3	141.0	145.5	148.1	153.0	157.9	160.6	165.0	166.7	170.0
12: 8	152	1	153.6234	0.04764	7.3186	136.6	139.9	141.6	146.0	148.7	153.6	158.6	161.2	165.7	167.4	170.6
12: 9	153	1	154.2223	0.04763	7.3456	137.1	140.4	142.1	146.6	149.3	154.2	159.2	161.8	166.3	168.0	171.3
12:10	154	1	154.8258	0.04763	7.3744	137.7	141.0	142.7	147.2	149.9	154.8	159.8	162.5	167.0	168.7	172.0
12:11	155	1	155.4329	0.04762	7.4017	138.2	141.5	143.3	147.8	150.4	155.4	160.4	163.1	167.6	169.4	172.7
13: 0	156	1	156.0426	0.04760	7.4276	138.8	142.1	143.8	148.3	151.0	156.0	161.1	163.7	168.3	170.0	173.3
13: 1	157	1	156.6539	0.04758	7.4536	139.3	142.6	144.4	148.9	151.6	156.7	161.7	164.4	168.9	170.7	174.0
13: 2	158	1	157.2660	0.04756	7.4796	139.9	143.2	145.0	149.5	152.2	157.3	162.3	165.0	169.6	171.3	174.7
13: 3	159	1	157.8775	0.04754	7.5055	140.4	143.8	145.5	150.1	152.8	157.9	162.9	165.7	170.2	172.0	175.3

2007 WHO Reference

Height-for-age BOYS

5 to 19 years (percentiles)

World Health Organization

						Percentiles (height in cm)										
Year: Month	Month	L	M	S	SD	1st	3rd	5th	15th	25th	50th	75th	85th	95th	97th	99th
13: 4	160	1	158.4871	0.04751	7.5297	141.0	144.3	146.1	150.7	153.4	158.5	163.6	166.3	170.9	172.6	176.0
13: 5	161	1	159.0937	0.04747	7.5522	141.5	144.9	146.7	151.3	154.0	159.1	164.2	166.9	171.5	173.3	176.7
13: 6	162	1	159.6962	0.04744	7.5760	142.1	145.4	147.2	151.8	154.6	159.7	164.8	167.5	172.2	173.9	177.3
13: 7	163	1	160.2939	0.04740	7.5979	142.6	146.0	147.8	152.4	155.2	160.3	165.4	168.2	172.8	174.6	178.0
13: 8	164	1	160.8861	0.04735	7.6180	143.2	146.6	148.4	153.0	155.7	160.9	166.0	168.8	173.4	175.2	178.6
13: 9	165	1	161.4720	0.04730	7.6376	143.7	147.1	148.9	153.6	156.3	161.5	166.6	169.4	174.0	175.8	179.2
13:10	166	1	162.0505	0.04725	7.6569	144.2	147.6	149.5	154.1	156.9	162.1	167.2	170.0	174.6	176.5	179.9
13:11	167	1	162.6207	0.04720	7.6757	144.8	148.2	150.0	154.7	157.4	162.6	167.8	170.6	175.2	177.1	180.5
14: 0	168	1	163.1816	0.04714	7.6924	145.3	148.7	150.5	155.2	158.0	163.2	168.4	171.2	175.8	177.6	181.1
14: 1	169	1	163.7321	0.04707	7.7069	145.8	149.2	151.1	155.7	158.5	163.7	168.9	171.7	176.4	178.2	181.7
14: 2	170	1	164.2717	0.04701	7.7224	146.3	149.7	151.6	156.3	159.1	164.3	169.5	172.3	177.0	178.8	182.2
14: 3	171	1	164.7994	0.04694	7.7357	146.8	150.3	152.1	156.8	159.6	164.8	170.0	172.8	177.5	179.3	182.8
14: 4	172	1	165.3145	0.04687	7.7483	147.3	150.7	152.6	157.3	160.1	165.3	170.5	173.3	178.1	179.9	183.3
14: 5	173	1	165.8165	0.04679	7.7586	147.8	151.2	153.1	157.8	160.6	165.8	171.1	173.9	178.6	180.4	183.9
14: 6	174	1	166.3050	0.04671	7.7681	148.2	151.7	153.5	158.3	161.1	166.3	171.5	174.4	179.1	180.9	184.4
14: 7	175	1	166.7799	0.04663	7.7769	148.7	152.2	154.0	158.7	161.5	166.8	172.0	174.8	179.6	181.4	184.9
14: 8	176	1	167.2415	0.04655	7.7851	149.1	152.6	154.4	159.2	162.0	167.2	172.5	175.3	180.0	181.9	185.4
14: 9	177	1	167.6899	0.04646	7.7909	149.6	153.0	154.9	159.6	162.4	167.7	172.9	175.8	180.5	182.3	185.8
14:10	178	1	168.1255	0.04637	7.7960	150.0	153.5	155.3	160.0	162.9	168.1	173.4	176.2	180.9	182.8	186.3
14:11	179	1	168.5482	0.04628	7.8004	150.4	153.9	155.7	160.5	163.3	168.5	173.8	176.6	181.4	183.2	186.7
15: 0	180	1	168.9580	0.04619	7.8042	150.8	154.3	156.1	160.9	163.7	169.0	174.2	177.0	181.8	183.6	187.1
15: 1	181	1	169.3549	0.04609	7.8056	151.2	154.7	156.5	161.3	164.1	169.4	174.6	177.4	182.2	184.0	187.5
15: 2	182	1	169.7389	0.04599	7.8063	151.6	155.1	156.9	161.6	164.5	169.7	175.0	177.8	182.6	184.4	187.9
15: 3	183	1	170.1099	0.04589	7.8063	152.0	155.4	157.3	162.0	164.8	170.1	175.4	178.2	183.0	184.8	188.3

2007 WHO Reference

Height-for-age BOYS

5 to 19 years (percentiles)

World Health Organization

Year: Month	Month	L	M	S	SD	Percentiles (height in cm) 1st	3rd	5th	15th	25th	50th	75th	85th	95th	97th	99th
15: 4	184	1	170.4680	0.04579	7.8057	152.3	155.8	157.6	162.4	165.2	170.5	175.7	178.6	183.3	185.1	188.6
15: 5	185	1	170.8136	0.04569	7.8045	152.7	156.1	158.0	162.7	165.6	170.8	176.1	178.9	183.7	185.5	189.0
15: 6	186	1	171.1468	0.04559	7.8026	153.0	156.5	158.3	163.1	165.9	171.1	176.4	179.2	184.0	185.8	189.3
15: 7	187	1	171.4680	0.04548	7.7984	153.3	156.8	158.6	163.4	166.2	171.5	176.7	179.6	184.3	186.1	189.6
15: 8	188	1	171.7773	0.04538	7.7953	153.6	157.1	159.0	163.7	166.5	171.8	177.0	179.9	184.6	186.4	189.9
15: 9	189	1	172.0748	0.04527	7.7898	154.0	157.4	159.3	164.0	166.8	172.1	177.3	180.1	184.9	186.7	190.2
15:10	190	1	172.3606	0.04516	7.7838	154.3	157.7	159.6	164.3	167.1	172.4	177.6	180.4	185.2	187.0	190.5
15:11	191	1	172.6345	0.04506	7.7789	154.5	158.0	159.8	164.6	167.4	172.6	177.9	180.7	185.4	187.3	190.7
16: 0	192	1	172.8967	0.04495	7.7717	154.8	158.3	160.1	164.8	167.7	172.9	178.1	181.0	185.7	187.5	191.0
16: 1	193	1	173.1470	0.04484	7.7639	155.1	158.5	160.4	165.1	167.9	173.1	178.4	181.2	185.9	187.7	191.2
16: 2	194	1	173.3856	0.04473	7.7555	155.3	158.8	160.6	165.3	168.2	173.4	178.6	181.4	186.1	188.0	191.4
16: 3	195	1	173.6126	0.04462	7.7466	155.6	159.0	160.9	165.6	168.4	173.6	178.8	181.6	186.4	188.2	191.6
16: 4	196	1	173.8280	0.04451	7.7371	155.8	159.3	161.1	165.8	168.6	173.8	179.0	181.8	186.6	188.4	191.8
16: 5	197	1	174.0321	0.04440	7.7270	156.1	159.5	161.3	166.0	168.8	174.0	179.2	182.0	186.7	188.6	192.0
16: 6	198	1	174.2251	0.04429	7.7164	156.3	159.7	161.5	166.2	169.0	174.2	179.4	182.2	186.9	188.7	192.2
16: 7	199	1	174.4071	0.04418	7.7053	156.5	159.9	161.7	166.4	169.2	174.4	179.6	182.4	187.1	188.9	192.3
16: 8	200	1	174.5784	0.04407	7.6937	156.7	160.1	161.9	166.6	169.4	174.6	179.8	182.6	187.2	189.0	192.5
16: 9	201	1	174.7392	0.04396	7.6815	156.9	160.3	162.1	166.8	169.6	174.7	179.9	182.7	187.4	189.2	192.6
16:10	202	1	174.8896	0.04385	7.6689	157.0	160.5	162.3	166.9	169.7	174.9	180.1	182.8	187.5	189.3	192.7
16:11	203	1	175.0301	0.04375	7.6576	157.2	160.6	162.4	167.1	169.9	175.0	180.2	183.0	187.6	189.4	192.8
17: 0	204	1	175.1609	0.04364	7.6440	157.4	160.8	162.6	167.2	170.0	175.2	180.3	183.1	187.7	189.5	192.9
17: 1	205	1	175.2824	0.04353	7.6300	157.5	160.9	162.7	167.4	170.1	175.3	180.4	183.2	187.8	189.6	193.0
17: 2	206	1	175.3951	0.04343	7.6174	157.7	161.1	162.9	167.5	170.3	175.4	180.5	183.3	187.9	189.7	193.1
17: 3	207	1	175.4995	0.04332	7.6026	157.8	161.2	163.0	167.6	170.4	175.5	180.6	183.4	188.0	189.8	193.2

2007 WHO Reference

Height-for-age BOYS

5 to 19 years (percentiles)

World Health Organization

Year: Month	Month	L	M	S	SD	Percentiles (height in cm) 1st	3rd	5th	15th	25th	50th	75th	85th	95th	97th	99th
17: 4	208	1	175.5959	0.04322	7.5893	157.9	161.3	163.1	167.7	170.5	175.6	180.7	183.5	188.1	189.9	193.3
17: 5	209	1	175.6850	0.04311	7.5738	158.1	161.4	163.2	167.8	170.6	175.7	180.8	183.5	188.1	189.9	193.3
17: 6	210	1	175.7672	0.04301	7.5597	158.2	161.5	163.3	167.9	170.7	175.8	180.9	183.6	188.2	190.0	193.4
17: 7	211	1	175.8432	0.04291	7.5454	158.3	161.7	163.4	168.0	170.8	175.8	180.9	183.7	188.3	190.0	193.4
17: 8	212	1	175.9133	0.04281	7.5308	158.4	161.7	163.5	168.1	170.8	175.9	181.0	183.7	188.3	190.1	193.4
17: 9	213	1	175.9781	0.04271	7.5160	158.5	161.8	163.6	168.2	170.9	176.0	181.0	183.8	188.3	190.1	193.5
17:10	214	1	176.0380	0.04261	7.5010	158.6	161.9	163.7	168.3	171.0	176.0	181.1	183.8	188.4	190.1	193.5
17:11	215	1	176.0935	0.04251	7.4857	158.7	162.0	163.8	168.3	171.0	176.1	181.1	183.9	188.4	190.2	193.5
18: 0	216	1	176.1449	0.04241	7.4703	158.8	162.1	163.9	168.4	171.1	176.1	181.2	183.9	188.4	190.2	193.5
18: 1	217	1	176.1925	0.04232	7.4565	158.8	162.2	163.9	168.5	171.2	176.2	181.2	183.9	188.5	190.2	193.5
18: 2	218	1	176.2368	0.04222	7.4407	158.9	162.2	164.0	168.5	171.2	176.2	181.3	183.9	188.5	190.2	193.5
18: 3	219	1	176.2779	0.04213	7.4266	159.0	162.3	164.1	168.6	171.3	176.3	181.3	184.0	188.5	190.2	193.6
18: 4	220	1	176.3162	0.04204	7.4123	159.1	162.4	164.1	168.6	171.3	176.3	181.3	184.0	188.5	190.3	193.6
18: 5	221	1	176.3518	0.04195	7.3980	159.1	162.4	164.2	168.7	171.4	176.4	181.3	184.0	188.5	190.3	193.6
18: 6	222	1	176.3851	0.04185	7.3817	159.2	162.5	164.2	168.7	171.4	176.4	181.4	184.0	188.5	190.3	193.6
18: 7	223	1	176.4162	0.04177	7.3689	159.3	162.6	164.3	168.8	171.4	176.4	181.4	184.1	188.5	190.3	193.6
18: 8	224	1	176.4453	0.04168	7.3542	159.3	162.6	164.3	168.8	171.5	176.4	181.4	184.1	188.5	190.3	193.6
18: 9	225	1	176.4724	0.04159	7.3395	159.4	162.7	164.4	168.9	171.5	176.5	181.4	184.1	188.5	190.3	193.5
18:10	226	1	176.4976	0.04150	7.3247	159.5	162.7	164.5	168.9	171.6	176.5	181.4	184.1	188.5	190.3	193.5
18:11	227	1	176.5211	0.04142	7.3115	159.5	162.8	164.5	168.9	171.6	176.5	181.5	184.1	188.5	190.3	193.5
19: 0	228	1	176.5432	0.04134	7.2983	159.6	162.8	164.5	169.0	171.6	176.5	181.5	184.1	188.5	190.3	193.5

2007 WHO Reference

APPENDIX 11 – LANSKY AND KARNOFSKY/ECOG SCALES

LANSKY PLAY SCALE

100 %	Fully active, normal
90%	Minor restrictions in strenuous physical activity
80%	Active, but tired more quickly
70%	Greater restriction of play *and* less time spent in play activity
60%	Up and around, but active play minimal; keeps busy by being involved in quieter activities
50%	Lying around much of the day, but gets dressed; no active playing participates in all quiet play and activities
40%	Mainly in bed; participates in quiet activities
30%	Bedbound; needing assistance even for quiet play
20%	Sleeping often; play entirely limited to very passive activities
10 %	Doesn’t play; does not get out of bed
0%	Unresponsive

KARNOFSKY AND ECOG SCALES

Karnofsky Index	Description	ECOG Scale
**Able to carry on normal activity; no special care is needed.**		
100	Normal, no complaints, no evidence of disease.	0
90	Able to carry on normal activity, minor symptoms or signs of disease.	1
80	Normal activity with effort, some signs or symptoms of disease.	
**Unable to work, able to live at home and care for most personal needs, varying amount of assistance needed.**		
70	Cares for self, unable to carry on normal activity or to do work.	2
60	Requires occasional assistance from others, but able to care for most needs	3
50	Requires considerable assistance from others and frequent medical care.	
**Unable to care for self, requires institutional or hospital care or equivalent, disease may be rapidly progressing.**		
40	Disabled, requires special care and assistance.	4
30	Severely disabled, hospitalisation indicated, death not imminent.	
20	Very sick, hospitalisation necessary, active supportive treatment necessary.	
10	Moribund, fatal processes progressing rapidly.	
0	Dead	5

ECOG: Eastern Cooperative Group

APPENDIX 12 – TANNER STAGING

Record the appropriate stage using the numbers 1, 2, 3, 4 or 5.

The assessment should include the male genital, public hair and female breast.

Male puberty stage:

Stages	Description
Stage 1	Preadolescent. Testes, scrotum, and penis are about the same size and proportion as those in early childhood.
Stage 2	Scrotum and testes have enlarged, and there is a change in the texture of scrotal skin and some reddening of scrotal skin.
Stage 3	Growth of the penis has occurred, at first mainly in length but with some increase in breadth. There has been further growth of the testes and the scrotum.
Stage 4	The penis is further enlarged in length and breadth, with development of glans. The testes and the scrotum are further enlarged. There is also further darkening of scrotal skin.
Stage 5	Genitalia are adult in size shape. No further enlargement takes place after stage 5 is reached.

Female puberty stage:

Stages	Description
Stage 1	Preadolescent; only papillae are elevated.
Stage 2	Breast bud and papilla are elevated and a small amount is present; areola diameter is enlarged.
Stage 3	Further enlargement of breast mound, increased palpable glandular tissue.
Stage 4	Areola and papilla are elevated to form a second mound above the level of the rest of the breast.
Stage 5	Adult mature breast; recession of areola to the mound of breast tissue, rounding of the breast mound, and projection of only the papilla are evident.

APPENDIX 13 – Clinical studies of anti-GD2 therapies in combination with chemotherapy

Clinical studies using anti-GD2 therapies in combination with chemotherapy (refer to Section 1.1.1)

Agent (reference)	Results
Hu14.18K322A in combination with topotecan/cyclophosphamide, irinotecan/temozolomide and ifosfamide/carboplatin/etoposide in relapsed & refractory patients. St. Jude pilot trial<br>(A Pilot Trial of Humanized Anti-GD2 Monoclonal Antibody (hu14.18K322A) with Chemotherapy and Natural Killer Cells in Children with Recurrent/Refractory Neuroblastoma. Federico SM et al. Clin Cancer Res. 2017 Nov 1;23(21):6441-6449. doi: 10.1158/1078-0432.CCR-17-0379. Epub 2017 Sep 22)	n=13. Response rate 61.5%, 1-year PFS 77%
Hu14.18K322A in combination with topotecan/cyclophosphamide, cyclophosphamide/doxorubicin/vincristine and cisplatin/etoposide in children as induction for newly diagnosed high risk neuroblastoma. St.Jude Pilot trial<br>(Improved clinical responses with the concomitant use of an anti-GD2 monoclonal antibody and chemotherapy in newly diagnosed children with high-risk (HR) neuroblastoma (NB): Preliminary results of a phase II study. Wayne Lee Furman et al. S. Pappo. Journal of Clinical Oncology 2016 34:15_suppl, 10501-10501)	n=20 Response rate 80%.
Dinutuximab plus GM-CSF with irinotecan-temozolomide. COG randomised phase 2 trial.<br>(Irinotecan–temozolomide with temsirolimus or dinutuximab in children with refractory or relapsed neuroblastoma (COG ANBL1221): an open-label, randomised, phase 2 trial. Lancet oncology [1470-2045] Mody, R (2017) 18 : 7 p.946)	n=34 (17 patients treated with dinutuximab). Median progression-free survival was 0.25 years for arm A [irinotecan-temozolomide-temsirolimus] compared to 2.14 years for arm B, [irinotecan-temozolomide-dinutuximab],
Dinutuximab beta plus German induction chemotherapy (GPOH) in relapsed&refractory high risk neuroblastoma. German pilot study, (Lode H, Abstract 61, Advances in Neuroblastoma Research 2018)	n=16. Best objective response rate 50%
